# Revision of the family Chalcididae (Hymenoptera, Chalcidoidea) from Vietnam, with the description of 13 new species

**DOI:** 10.3897/zookeys.576.8177

**Published:** 2016-04-04

**Authors:** T. C. Narendran, Cornelis van Achterberg

**Affiliations:** 1 Deceased, formerly Department of Zoology, University of Calicut, Kerala, 673635, India; 2 Department of Terrestrial Zoology, Naturalis Biodiversity Center, Postbus 9517, 2300 RA Leiden, The Netherlands

**Keywords:** Chalcididae, revision, new species, keys, distribution, Vietnam, Oriental, new records

## Abstract

A total of 16 genera and 68 species of Chalcididae of Vietnam are taxonomically treated. Thirteen new species are described; the remaining 55 species are keyed, redescribed or provided with a diagnosis. Among these 37 species and eleven genera are recorded for the first time from Vietnam. The thirteen new species are: *Antrocephalus neogalleriae* Narendran & van Achterberg, **sp. n.**; *Brachymeria neowiebesina* Narendran & van Achterberg, **sp. n.**, *Brachymeria semirusula* Narendran & van Achterberg, **sp. n.**, *Dirhinus neoclaviger* Narendran & van Achterberg, **sp. n.**, *Epitranus narendrani* van Achterberg, **sp. n.**, *Epitranus neonigriceps* Narendran & van Achterberg, **sp. n.**, *Heydoniella vietnamensis* Narendran & van Achterberg, **sp. n.**, *Megachalcis vietnamicus* Narendran & van Achterberg, **sp. n.**, *Notaspidium vietnamicum* Narendran & van Achterberg, **sp. n.**, *Oxycoryphe neotenax* Narendran & van Achterberg, **sp. n.**, *Sthulapada neopadata* Narendran & van Achterberg, **sp. n.**, *Sthulapada vietnamensis* Narendran & van Achterberg, **sp. n.**, and *Tanycoryphus masii* Narendran & van Achterberg, **sp. n.**

The newly recorded genera are: *Antrocephalus* Kirby, 1883; *Haltichella* Spinola, 1811; *Heydoniella* Narendran, 2003; *Hockeria* Walker, 1834; *Kriechbaumerella* Dalla Torre, 1894; *Notaspidium* Dalla Torre, 1897; *Oxycoryphe* Kriechbaumer, 1894; *Psilochalcis* Kieffer, 1904; *Sthulapada* Narendran, 1989; *Tanycorphus* Cameron, 1905, and *Trigonura* Sichel, 1865.

The following known species are recorded for the first time from Vietnam: *Antrocephalus decipiens* (Masi, 1929); *Antrocephalus lugubris* (Masi, 1932); *Antrocephalus maculipennis* (Cameron, 1905); *Antrocephalus nasutus* (Holmgren, 1869); *Antrocephalus sepyra* (Walker, 1846); *Antrocephalus validicornis* (Holmgren, 1868); *Brachymeria alternipes* (Walker, 1871); *Brachymeria aurea* (Girault, 1915); *Brachymeria coxodentata* Joseph, Narendran & Joy, 1972; *Brachymeria euploeae* (Westwood, 1837); *Brachymeria hime* Habu, 1960; *Brachymeria jambolana* Gahan, 1942; *Brachymeria kamijoi* Habu, 1960; *Brachymeria lugubris* (Walker, 1871); *Brachymeria marmonti* (Girault, 1924); *Brachymeria minuta* (Linnaeus, 1767); *Brachymeria scutellocarinata* Joseph, Narendran & Joy, 1972; *Brachymeria shansiensis* Habu, 1961; *Brachymeria taiwana* (Matsumura, 1910); *Dirhinus anthracia* Walker, 1846; *Dirhinus claviger* Bouček & Narendran, 1981; *Epitranus albipennis* Walker, 1874; *Epitranus ater* Bouček, 1982; *Epitranus gauldi* Bouček, 1982; *Epitranus oxytelus* Bouček, 1982; *Epitranus ramnathi* (Mani & Dubey, 1973); *Haltichella delhensis* Roy & Farooqi, 1984; *Haltichella nipponensis* Habu, 1960; *Hockeria bangalorica* Narendran, 1989; *Hockeria guptai* Narendran, 1989; *Kriechbaumerella ayyari* (Gahan, 1919); *Kriechbaumerella cordigaster* Roy & Farooqi, 1984; *Kriechbaumerella destructor* (Waterston, 1922); *Kriechbaumerella nepalensis* Narendran, 1989; *Oxycoryphe scutellatus* Narendran, 1989; *Psilochalcis carinigena* (Cameron, 1907), and *Trigonura luzonensis* Narendran, 1987. *Brachymeria calopeplae* Joseph, Narendran & Joy, 1972, is treated as a valid species.

## Introduction

The family Chalcididae Latreille, 1817 (Hymenoptera: Chalcidoidea) is one of the most interesting and difficult hymenopteran families to study taxonomically. They show morphological resemblances among genera and species – a phenomenon widely found in other Chalcidoidea families as well - and it is not easy to separate them at species level and often at generic level. Though many species of Chalcididae look very much alike, they differ widely in habits. Members of this family comprise medium to large chalcids which vary from 1.5 to 15 mm in length. Members of this family comprise some of the largest specimens of the chalcidoid families. Chalcidids can be recognized by the swollen hind femur, the indistinct prepectus, the sharp occipital carina bordering the gena posteriorly, the strong punctation of the mesosoma, the side of the scapula separating the pronotum from the tegula, the shallow femoral depression (“facies femoralis” of Bouček (1952)) of the mesopleuron and the tarsi with five segments. Colour of chalcidids is often black or black with yellow or white or red markings. Body usually is without metallic refringence (in *Notaspidium* some species are with metallic refringence; Figs 169–172). In *Dirhinus* frontal horns are present. In *Epitranus* the clypeus is dorsally concave, pilose, and its distal margin is vaguely tri-lobed. In *Smicromorpha* the antennal flagellum has less than 11 segments; the metasoma is unusually narrow and tail-like and is a parasitoid of ants. There are about 90 valid genera and approximately 1500 valid species so far described from the world (Noyes, 2011). From Vietnam 18 species of Chalcididae are known of which 10 are listed in the chalcidoid database: (www.nhm.ac.uk/research-curation/research/projects/chalcidoids/database/) and with this paper a total of 68 species belonging to 16 genera (37 species and 11 genera new for Vietnam) are known.

## Historical résumé

Linnaeus (1767) was the first to describe species of Chalcididae (sensu stricto), viz., *Sphex sispes* (now *Chalcis sispes*) and *Vespa minuta* (= *Brachymeria minuta*) 249 years ago. Two decades later Fabricius (1787) coined the name “*Chalcis*” from which the name of the present superfamily Chalcidoidea is derived. Later Latreille (1817) formally established the name *Chalcidites* which was later amended to *Chalcididae*. Walker (1862) was the first to establish the name *Chalcididae* in the presence sense. Ashmead (1896) raised it to superfamily level which was later changed to Chalcidoidea. Some of the prominent early workers of Chalcididae include Westwood (1832–1839), Dalla Torre (1897), Spinola (1811), Dalman (1818), Foerster (1859), Klug (1834), and Kirby (1883). In 1904 Ashmead published his paper on the classification of Chalcidoidea in which he dealt with the chalcidid genera. Since then our knowledge on the taxonomy of the family has been greatly enhanced by the studies of Cameron (1903–1913), Crawford (1910, 1913), Waterston (1914, 1922), Girault (1910–1939), Gahan (1919–1946), Masi (1916–1951) and many others. Some of the prominent contributions to the taxonomy of Chalcididae in recent years include Bouček (1952, 1988a+b), Bouček and Delvare (1992), Steffan (1949–1957), Burks (1936, 1960), Nikol’skaya (1952, 1960), Mani (1989), Habu (1960–1963), Joseph, Narendran & Joy (1973), Naumann (1986), Narendran (1989) and Wijesekara (1997). In the Oriental region the only taxonomic revision of the family is that of Narendran published in 1989.

## Classification

The classification of Chalcididae follows Bouček (1988a) by using five subfamilies. They are: 1) Chalcidinae (with tribes Chalcidini, Brachymeriini, Cratocentrini and Phasgonophorini); 2) Haltichellinae (with tribes Haltichellini, Hybothoracini and Tropimeridini); 3) Dirhininae (with tribes Dirhinini and Aplorhinini); 4) Epitraninae (no tribes) and 5) Smicromorphinae (no tribes).

Until recently (Riek 1970), the family Leucospidae was included under the Chalcididae as a subfamily. However, Bouček and all other recent workers considered Leucospidae as a separate family.

## Phylogeny

Up to recently it was generally accepted that the family Chalcididae (sensu stricto) is a monophyletic lineage within the superfamily Chalcidoidea largely based on four morphological synapomorphies (Wijesekara 1997; Gibson 1999). Studies of Wijesekara (1997) states that there are four unambiguous characters supporting the monophyly of Chalcididae excluding Leucospidae, viz., 1) derived shape of labrum; 2) exposed and straight bases of mandibles; 3) convergence of parascutal and axillar carinae and 4) the presence of a genal or occipital carina. Leucospidae is the sister group of Chalcididae as supported by two synapomorphies: 1) the enlarged and ventrally toothed hind femur and 2) the elongated transversely orientated propodeal spiracles. According to the molecular analyses of Munro et al. (2011) Chalcididae and Chalcidinae were not monophyletic in any of their analyses and Leucospidae were not included in one of the monophyletic groups found in the Chalcididae. Cratocentrini (*Cratocentrus* and *Acanthochalcis*) were excluded from the Chalcididae. The subfamilies Epitraninae, Dirhininae and Haltichellinae were all monophyletic with strong support as the tribes Brachymeriini, Chalcidini and Phasgonophorini of the subfamily Chalcidinae (Munro et al. 2011).

## Distribution

The family Chalcididae is worldwide in distribution. Of the 38 genera and 447 species known from Oriental region (Noyes 2011) only five genera and ten species are so far reported from Vietnam, but additional species are listed in Narendran (1989). In this paper we record 68 species (including thirteen new species) belonging to 16 genera. Among the Vietnamese Chalcididae the most diverse genus is found to be *Brachymeria* Westwood, 1829, with 25 species followed by *Antrocephalus* Kirby, 1883, with seven species.

## Biology

While the majority of the species of Chalcididae are primary solitary endoparasitoids, some are secondary parasitoids or gregarious parasitoids. Chalcidids attack a wide range of hosts. The most targeted hosts are Lepidoptera followed by Diptera, Coleoptera and Hymenoptera. Some species attack Neuroptera, Orthoptera, and Strepsiptera. So far no host is recorded from Vietnam, except of *Smicromorpha masneri* Darling, 2009.

## Material and methods

The majority of material for this study of Chalcididae was collected by the second author (CvA) from different localities in Vietnam. Some other specimens collected from Vietnam were sent to the first author (TCN) from BPBM during 1980–1989. The material collected by CvA is mainly from Malaise traps, with some specimens taken by sweep netting. In the laboratory the material were studied using a Leica stereozoom binocular by TCN. The colour photographs are made with an Olympus SZX12 motorized stereomicroscope with AnalySIS Extended Focal Imaging Software by CvA. All primary types of the new species are deposited in RMNH (Leiden) and duplicates in IEBR (Hanoi).

### Depositories

BMNH
Natural History Museum, London, England, U.K. 


BPBM
 Bernice P. Bishop Museum, Honolulu, Hawaii, U.S.A. 


CNC
 Canadian National Collection, Ottawa, Canada 


DEI
 Senckenberg Deutsches Entomologisches Institute, Müncheberg, Germany 


DZCU
 Department of Zoology, University of Calicut, Kerala, India 


EIHU
 Entomological Laboratory, Hokkaido University, Japan 


FRID
Forest Research Institute, New Forest, Dehra Dun, India 


HDOU
 Hope Department, Oxford University, England, U.K. 


IEBR
 Institute of Ecology & Biological Resources, Vietnam Academy of Science & Technology, Hanoi, Vietnam 


INPC
National Pusa Collections, Indian Agriculture Research Institute, Haryana, New Delhi, India 


ITZA
 former Zoological Museum, University of Amsterdam (ZMA), Amsterdam, now in RMNH



JXAU
Jiangxi Agricultural University, Nanchang, China 


KYUN
 Entomological Laboratory, Faculty of Agriculture, Kyushu University, Fukuoka, Japan 


LUZN
Zoological Museum, Lund University, Sweden 


NHMV
Naturhistorisches Museum, Wien, Austria 


NHRM
Naturhistoriska Riksmuseet, Stockholm, Sweden 


NZSI
 National collections of Zoological Survey of India, Kolkotha, India 


QMB
Queensland Museum, Brisbane, Australia 


RMNH
Naturalis Biodiversity Center, Leiden, Netherlands (including collections from the Entomological Institute, Wageningen, National Museum of Natural History Leiden and Zoological Museum, Amsterdam) 


ROMT
Royal Ontario Museum, Toronto, Canada 


SAMA
 South Australian Museum, Adelaide, South Australia 


SFTD
WB Biologie, Sektion Forstwirtschaft, Thanrandt, Dresden, Germany 


USNM
 U. S. National Museum of Natural History, Smithsonian Institution, Washington D.C., U.S.A. 


ZDAMU
 Zoology Department of Aligarh Muslim University, Aligarh, India 


ZMB
Zoological Museum, Humboldt Universität, Berlin, Germany 


ZMCU
Zoological Museum, Copenhagen, Denmark 


### Terms and measurements

The terminology used is mainly that of Bouček (1952, 1988b) and Narendran (1989); for details see following figures. The general abbreviations of the terms are as follows:

AOL
 distance between anterior ocellus and posterior ocellus (Fig. 2) 


F1–F7
 ﻿ first to seventh funicular segments (IV-X in Fig. 3) 


**Figures 1–3.**
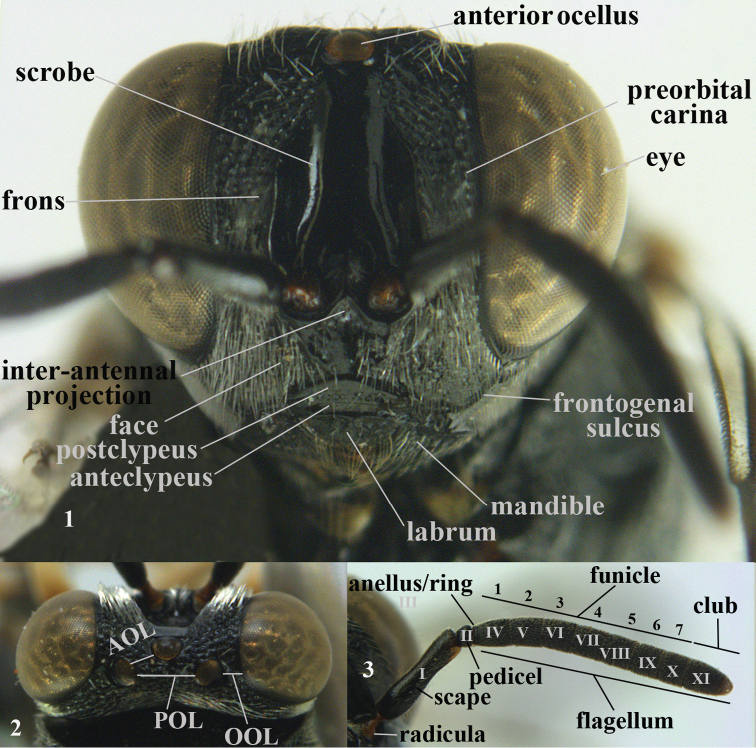
nomenclature of head (anterior, dorsal) and antenna, *Brachymeria lasus* Walker, female, Vietnam. In Roman ciphers the antennal segments and in Arabic ciphers the funicular segments.

LOL
 diameter of ocellus 


MV
 marginal vein (Fig. 6) 


**Figures 4–6.**
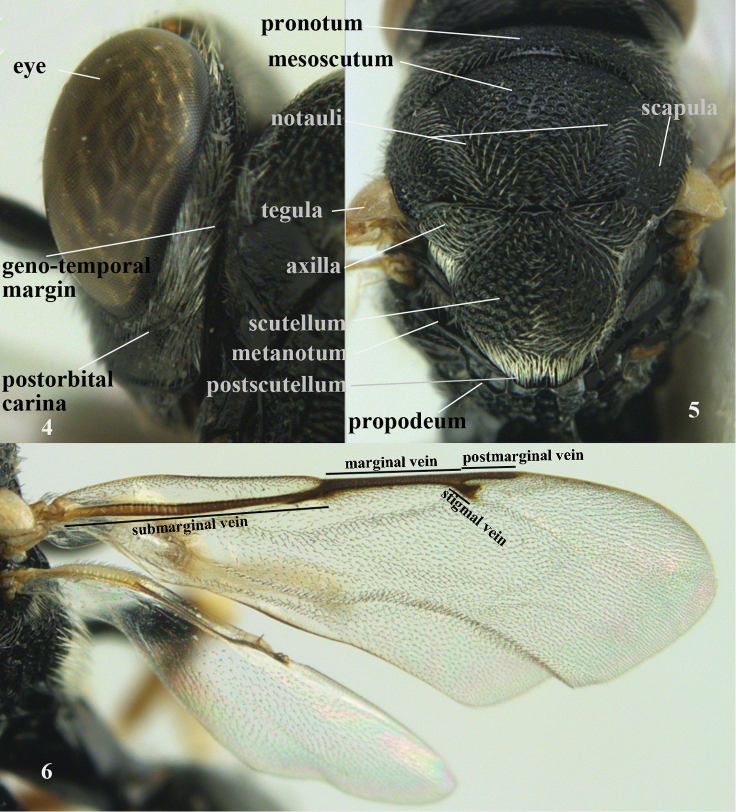
nomenclature of head (lateral), mesoscutum and wings, *Brachymeria lasus* Walker, female, Vietnam.

OOL
 minimum distance between posterior ocelli and eye (Fig. 2) 


PMV
 postmarginal vein (Fig. 6) 


POL
 distance between two posterior ocelli (Fig. 2) 


SMV
 submarginal vein (Fig. 6) 


sp. n.
 species nova (new species) 


STV
 stigmal vein (Fig. 6) 


S1-S5
 first-fifth metasomal (or gastral) sternites (Fig. 8) 


**Figures 7–8.**
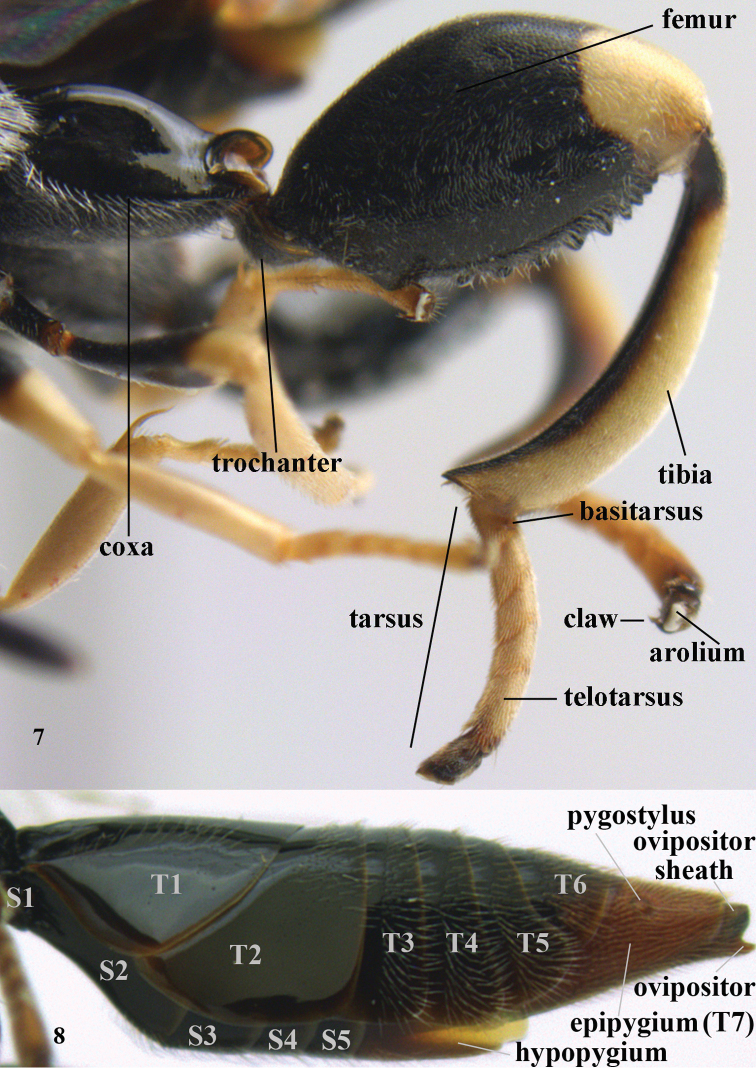
nomenclature of hind leg of *Brachymeria lasus* Walker, female, Vietnam and metasoma (lateral) of *Sthulapada vietnamensis* sp. n., holotype.

T1-T6
 first to sixth metasomal (or gastral) tergites (Fig. 8) 


## Taxonomy

### Key to genera of Chalcididae from Vietnam

**Table:** 

1	Hind tibia almost perpendicularly truncated at apex and with two spurs (Fig. 132); (Haltichellinae)	**2**
–	Hind tibia obliquely truncated at apex, forming broadly a subtriangular or elongate stout spine which is produced well beyond insertion of tarsus (Fig. 7); only one spur present (or even this one is inconspicuous)	**12**
2	Antennal flagellum usually much shorter than length of eye and with less than 11 distinguishable segments (Figs 185, 188); metasoma (gaster) usually narrow, tail like and attached at upper margin of propodeum (Fig. 185); STV subperpendicular and as long as MV (Fig. 185); (Smicromorphinae)	***Smicromorpha* Girault**
–	Antenna normal and with at least 11 segments (Fig. 135); metasoma normal, not tail-like, attached near base of hind coxae (Fig. 135); STV not as above (Figs 16, 135, 155)	**3**
3	MV more or less on wing margin (Figs 16, 143, 173); PMV present (occasionally stub-like); STV distinctly developed (Fig. 16); (Haltichellini)	**4**
–	MV short and distinctly removed from anterior wing margin (Fig. 169); PMV absent; STV usually rudimentary (Fig. 169); (Hybothoracini)	**11**
4	Tip of hind tibia with a robust outer spur and an outer carina (Fig. 195); clava long and narrowed (Fig. 195); fore tibia swollen (Fig. 197); postscutellum with longitudinal rugae (Fig. 198)	***Tanycoryphus* Cameron**
–	Tip of hind tibia without a slenderer outer spur (Fig. 173) and outer carina variable; clava shorter (Fig. 173); fore tibia usually less swollen (Figs 132, 135, 192); postscutellum with different sculpture	**5**
5	Hind tibia externally with an additional outer carina at least on its distal half (Figs 135, 173, 189)	**6**
–	Hind tibia without additional outer carina (Figs 23, 147, 163)	**9**
6	Pronotum with a median tubercle or tooth or raised carina or a marked triangle (Fig. 181; rarely weakly represented but distinctly indicated); [apex of scutellum in most cases distinctly produced posteriorly (and in a few cases emarginate)]	***Oxycoryphe* Kriechbaumer**
–	Pronotum bi-tuberculate or bi-toothed medially, without a tubercle or tooth or triangular area (Figs 138, 140, 141)	**7**
7	Pronotum with two humps or teeth medially (Fig. 194); hind tibia clavate (Fig. 192); hind tarsal segments usually swollen (Figs 189, 192); epipygium with a tooth directed posteriorly (Figs 189, 192)	***Sthulapada* Narendran**
–	Pronotum without any tooth or tubercle (Fig. 146); hind tibia not clavate (Fig. 143); tarsal segments normal (Fig. 143); epipygium without a tooth (Fig. 135)	**8**
8	Hind coxa with a tuft of pubescence dorsally (Fig. 145); medio-posteriorly apex of scutellum prolonged (Fig. 146)	***Heydoniella* Narendran**
–	Hind coxa without conspicuous pubescence (Fig. 139); medio-posteriorly apex of scutellum emarginated or bi-lobed or with two teeth (Figs 133, 137)	***Haltichella* Spinola**
9	Face with strong horse-shoe shaped carinae, from behind anterior ocellus to inner margin of eyes (Figs 19, 156); scrobe usually deep (Fig. 157)	**10**
–	Face without such a horse-shoe shaped carina (Fig. 148), if carinae present usually weaker and not turning mesad dorsally behind anterior ocellus (Fig. 150); scrobe usually shallow (Fig. 150)	***Hockeria* Walker**
10	Hind femur characteristically tri-lobed with comb of teeth (Figs 155, 163); pronotal carina indistinct or only laterally distinct and without medial tubercles (Fig. 157)	***Kriechbaumerella* Dalla Torre**
–	Hind femur bi- or uni-lobed or without lobe (Figs 9, 14, 16); pronotal carina distinct and with strong to weak medial tubercles (Fig. 10)	***Antrocephalus* Kirby**
11	T1 dorsally more or less flattened with at least some longitudinal carinae (two or more) anteriorly united by a transverse elevation or carina (Fig. 170); apex of scutellum arcuate or angulate (Fig. 172; in extralimital species often produced posteriorly into a spine or short horn); hind femur with a triangular tooth just beginning at comb of teeth near middle of femur (Fig. 169); hind tibia with a longitudinal band of closely spaced small pits dorsally; body often with slight or strong metallic refringence in some species (Fig. 171)	***Notaspidium* Dalla Torre**
–	T1 without longitudinal carinae, its posterior margin arcuately produced; other characters different from above, hind femur with a massive basal tooth in some species (Fig. 183); [horizontal ventral part of mesopleuron anteriorly with a carina defining margin of area for reception of fore coxa; in some species upper margin of clypeus produced anteriorly]	***Psilochalcis* Kieffer**
12	Metasoma with a petiolate first segment (Figs 85, 88, 98, 111); if petiole relatively short (much shorter than remainder of metasoma) then head with horns (Fig. 81)	**13**
–	Metasoma sessile (Fig. 27); frontal horns absent	**14**
13	Head with two projecting horns (Figs 81, 84); metasomal petiole shorter than half length of T1 (Fig. 85); antennal toruli located higher on projecting shield (Fig. 83); hind femur with smoothly arched comb of teeth (Fig. 79); (Dirhininae)	***Dirhinus* Dalman**
–	Head without horns; petiole longer than T1 (Figs 88, 98, 111); antennal toruli located very low on protruding shield (Fig. 113); hind femur with a ventral row of differently sized teeth or shaped comb of teeth (Figs 109, 119); (Epitraninae)	***Epitranus* Fabricius**
14	PMV much longer than MV (Fig. 165; narrow and usually more than twice MV); T1 large (usually more than half length of remainder of metasoma; Fig. 167); T2-T4 more or less reduced and partly hidden under T1; [females with a long ovipositor sheath]; (Cratocentrini)	***Megachalcis* Cameron**
–	PMV shorter than MV (Figs 65, 68); other characters different from above	**15**
15	Malar sulcus between eye and mouth distinct, usually indicated as a ridge or carina (Fig. 67); PMV usually longer than STV (Fig. 62); (Brachymeriini)	***Brachymeria* Westwood**
–	Malar sulcus indistinct or absent and malar area regularly punctate (Fig. 200); PMV hardly as long as or shorter than STV (Fig. 200); (Phasgonophorini)	***Trigonura* Sichel**

#### Antrocephalus

Taxon classificationAnimaliaHymenopteraChalcididae

Kirby, 1883

Figs 9 , 10–11 , 12–13 , 14–15 , 16–17 , 18 , 19–20 , 21–22 , 23–24

Antrocephalus Kirby, 1883: 54, 63. Type species: Halticella fascicornis Walker; designated by Kirby (1883).
Coelochalcis Cameron, 1904: 110. Type species: Coelochalcis carinifrons Cameron; by monotypy (synonymised with Antrocephalus by Narendran (1986)).
Dilla Strand, 1911: 210. Type species: Antrocephalus rufipes Kieffer; by original designation (synonymised with Antrocephalus by Steffan (1953)).
Stomatoceroides Girault, 1913b: 140. Type species: Stomatoceroides bicolor Girault; by original designation. (synonymised with Antrocephalus by Bouček (1988b)).
Metarretocera Girault, 1927: 325. Type species: Metarretocera bursi Girault, by monotypy (synonymised with Antrocephalus by Bouček (1988b)).
Tainania Masi, 1929: 159. Type species Tainania acutiventris Masi, by original designation (synonymised by Narendran, 1977 with Antrocephalus).
Sabatiella Masi, 1929c: 167–168. Type species: Sabatiella nigra Masi, by original designation (synonymised with Antrocephalus by Habu (1960)).
Stomatocerella Girault, 1930: [4]. Type species: Stomatocerella anna Girault; by monotypy (synonymised with Antrocephalus by Bouček (1988b)).
Uxa Girault, 1930:4. Type species Stomatoceroides clariscapus Dodd; by original designation [synonymized with Antrocephalus by Bouček (1988b)].
Dillisca Ghesquière, 1946: 367. Replacement name for Dilla Strand considered to be preoccupied by Dila Fisher de Waldheim (synonymized with Antrocephalus by Steffan (1953)).

##### Diagnosis.

The genus *Antrocephalus* is a difficult genus to define its limits. This is because the genus comes very close to *Hockeria* and rarely also to *Kriechbaumerella*. Only with extensive expertise in the taxonomy of these genera one can authoritatively identify this genus, which in future perhaps will be more reliably possible by using molecular markers.

##### Description.

Head with a horse shoe-shaped carina running behind anterior ocellus; scrobe fairly deep and reaching anterior ocellus; pronotum with anterior carinae and often with submedian tubercles in majority of species; apex of scutellum variable; geno-temporal margin with or without geno-temporal furrow; metasoma usually with two submedian short or long carinae in most species.

##### Hosts.

Pupae of Lepidoptera.

##### Distribution.

Europe, Asia, Australia and New Guinea. Apparently introduced in South America (Bouček 1988b).

##### Variation.

There is a wide variation of the general characters. In some species the pronotal carina and tubercles are weak or absent; in a few species the scrobe is shallow, not very deep as in typical species, in some others the metasoma without basal carinae on T1 indistinct.

##### Key to Vietnamese species of *Antrocephalus* Kirby (based on females)

**Table:** 

1	Basal carinae on T1 absent (but with a pit basally); pre-orbital carina joining directly with auricular carina (= dorsal carinate margin of clypeus); scutellum with a median fovea	**2**
–	Basal carinae on T1 present; pre-orbital carina not directly joining auricular carina; scutellum variable	**4**
2	Apex of scutellum with a thin split between both lobes; fore wing without two broad infuscations	***Antrocephalus lugubris* (Masi)**
–	Apex of scutellum weakly or well emarginate; fore wing with two broad infuscations	**3**
3	Pronotum with anterior carinae and submedially with two tiny tubercles; PMV about 1.5 × as long as MV; apex of scutellum well emarginate	***Antrocephalus maculipennis* Cameron**
–	Pronotum with anterior carinae and tubercles absent; PMV about as long as MV; apex of scutellum weakly emarginate	***Antrocephalus neogalleriae* sp. n.**
4	Geno-temporal furrow absent in front of occipital carina; pre-orbital carina less developed and not visible in lateral view (Fig. 9)	***Antrocephalus decipiens* (Masi)**
–	Geno-temporal furrow present and usually deep; pre-orbital carina distinct and visible in lateral view	**5**
5	PMV about 1.5 × longer than MV	**6**
–	PMV slightly longer than MV	***Antrocephalus sepyra* (Walker)**
6	Ventral comb of teeth of hind femur occupying 0.7 × length of femur (Fig. 16); apex of scutellum distinctly incised (Fig. 17); hind femur red	***Antrocephalus nasutus* (Holmgren)**
–	Ventral comb of teeth of hind femur occupying much less than 0.7 of femur (Fig. 23); apex of scutellum less emarginated (Fig. 24); hind femur black with base and apex red	***Antrocephalus validicornis* (Holmgren)**

**Figure 9.**
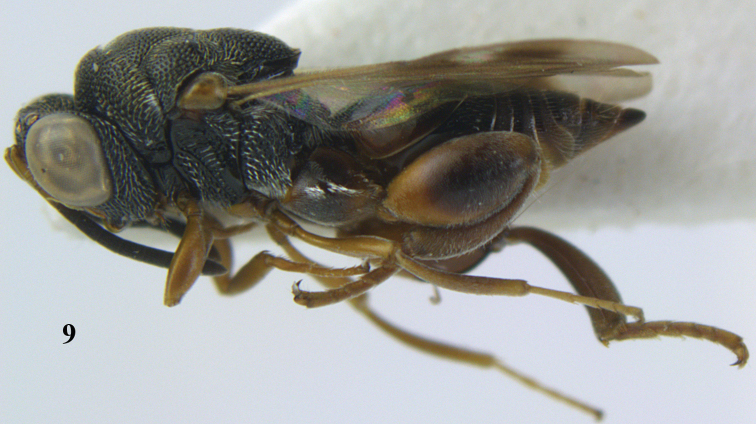
*Antrocephalus decipiens* (Masi), ♀, Vietnam, Cát Tiên N.P., habitus lateral.

**Figures 10–11.**
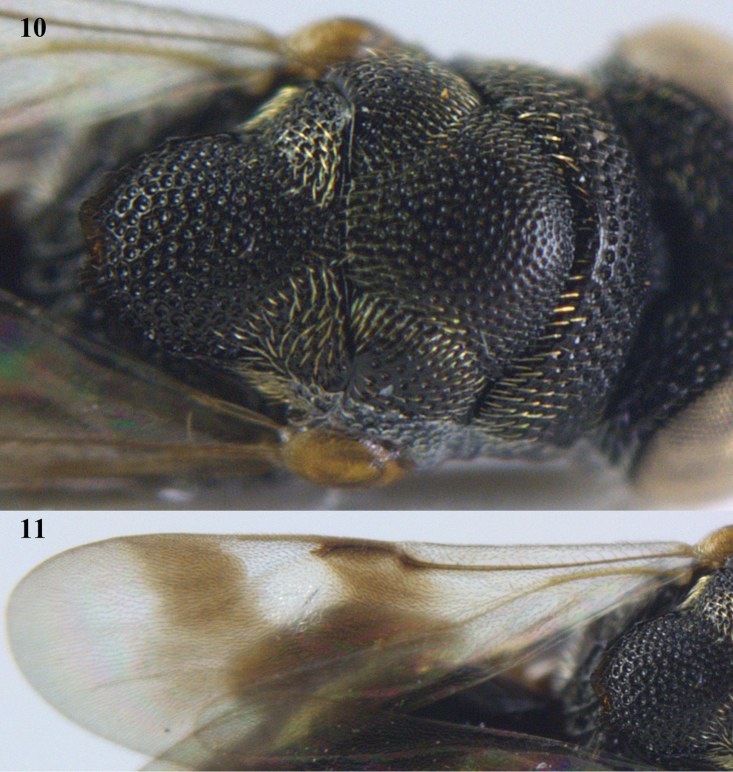
*Antrocephalus decipiens* (Masi), ♀, Vietnam, Cát Tiên N.P. **10** mesonotum dorsal **11** fore wing.

**Figures 12–13.**
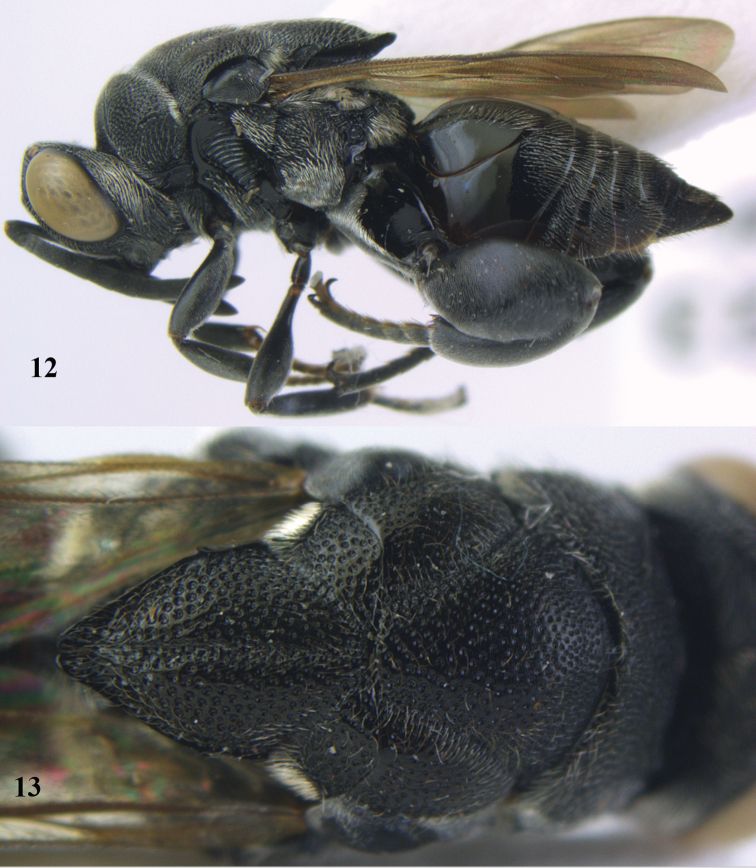
*Antrocephalus lugubris* (Masi), ♀, Vietnam, Cát Tiên N.P. **12** habitus lateral **13** mesonotum dorsal.

**Figures 14–15.**
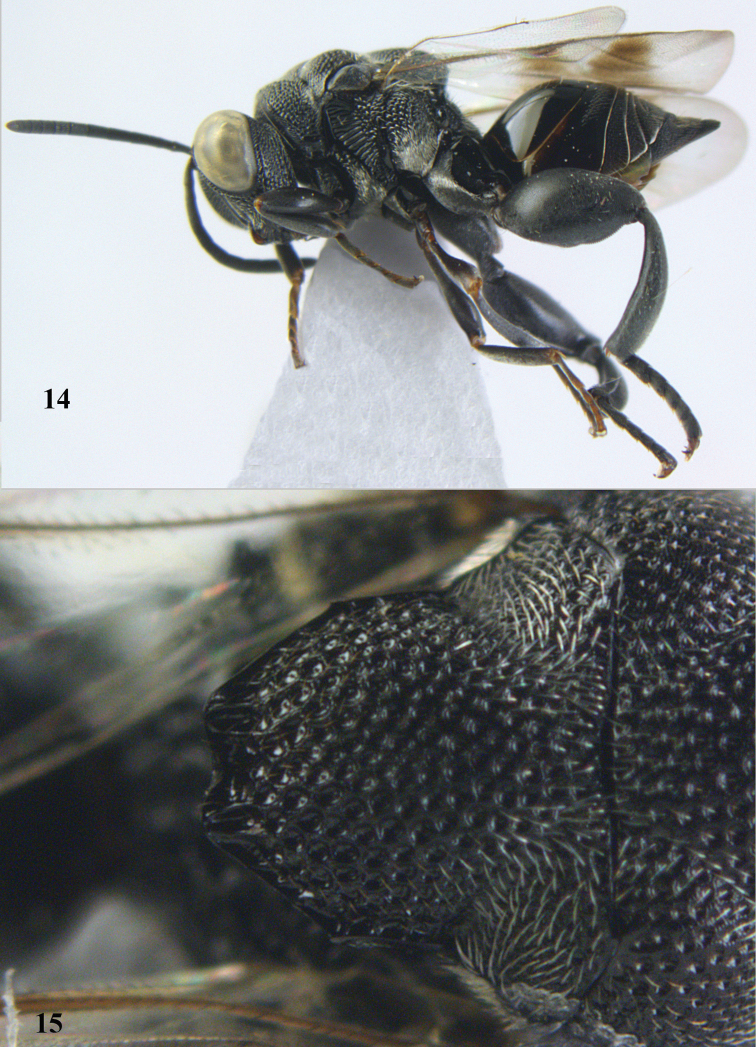
*Antrocephalus maculipennis* Cameron, ♀, Vietnam, Cát Tiên N. P. **14** habitus lateral **15** scutellum dorsal.

**Figures 16–17.**
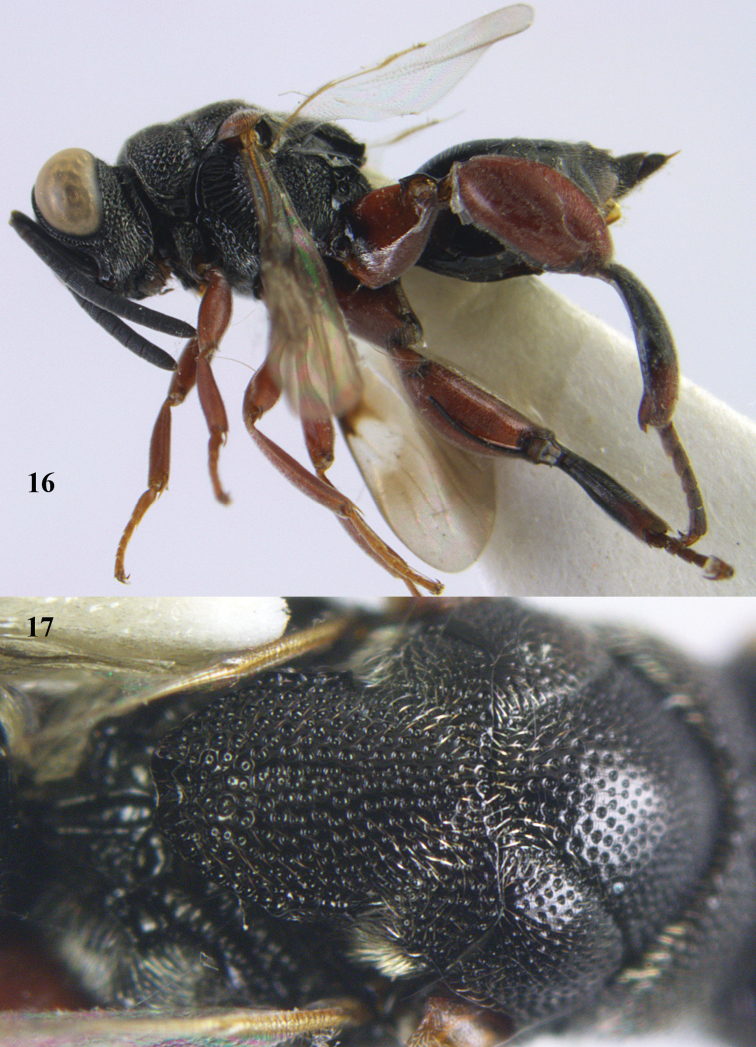
*Antrocephalus nasutus* (Holmgren), ♀, Vietnam, Cat Tien N.P. **16** habitus lateral **17** mesonotum dorsal.

**Figure 18.**
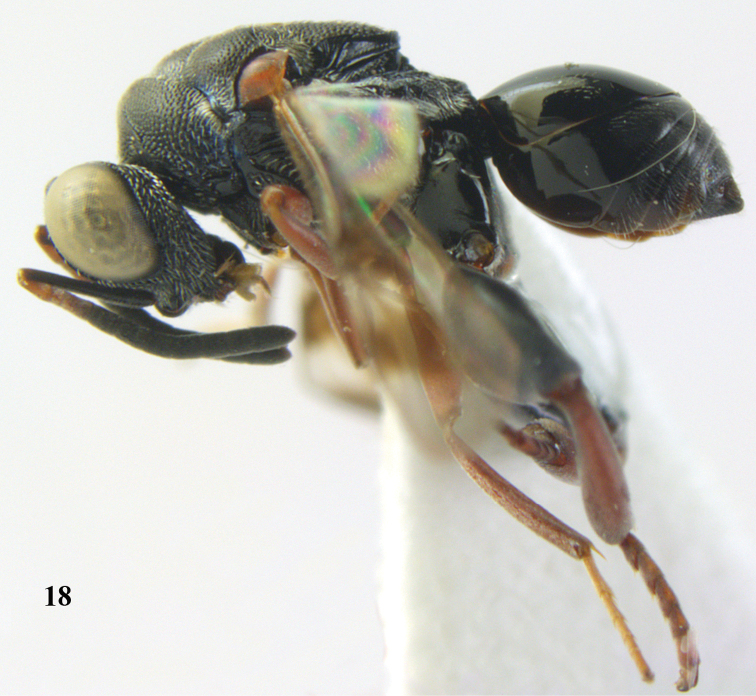
*Antrocephalus neogalleriae* sp. n., ♀, holotype, habitus lateral.

**Figures 19–20.**
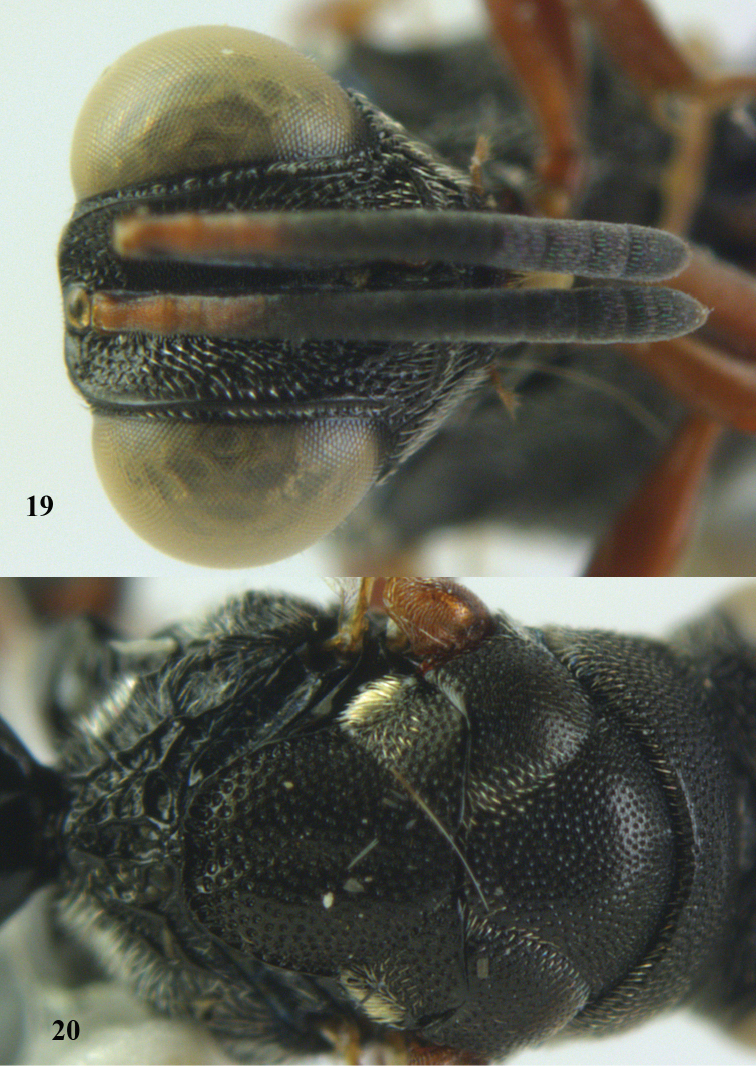
*Antrocephalus neogalleriae* sp. n., ♀, holotype. **19** head anterior **20** mesonotum dorsal.

**Figures 21–22.**
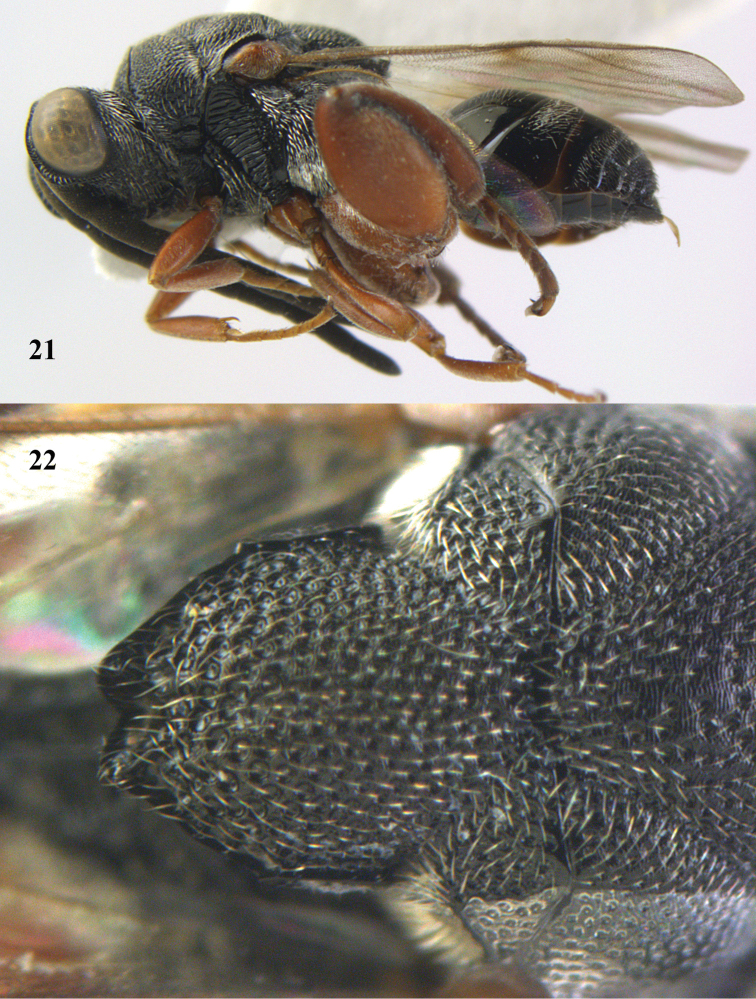
*Antrocephalus sepyra* (Walker), ♂, Vietnam, Núi Chúa N. P. **21** habitus lateral **22** scutellum dorsal.

**Figures 23–24.**
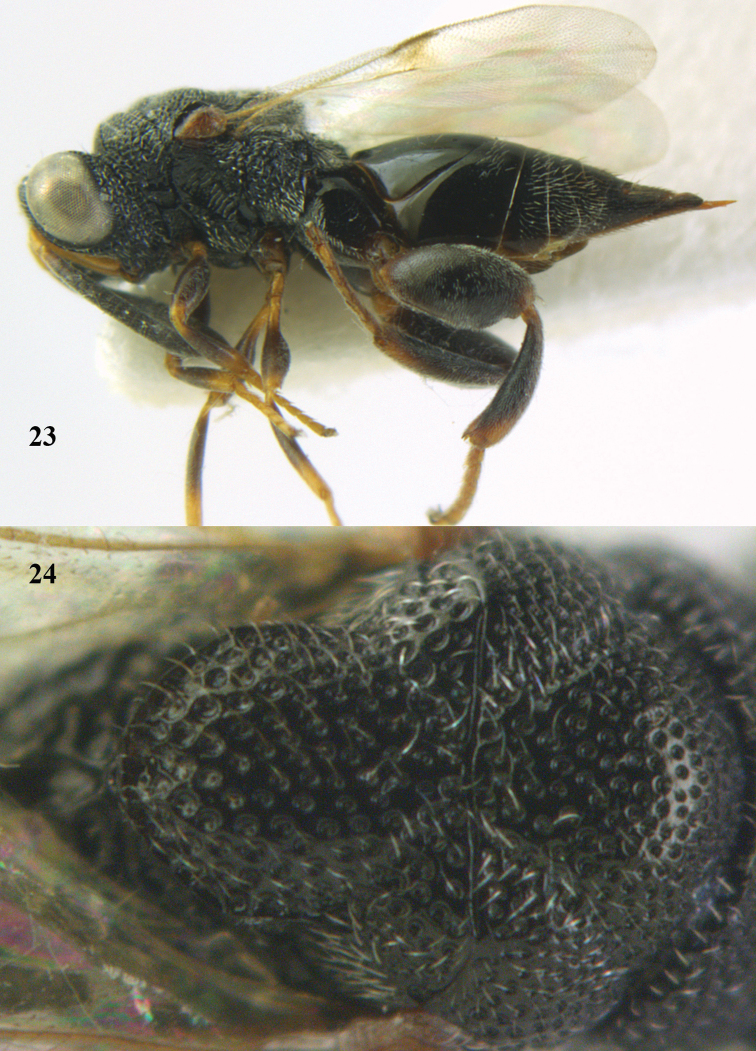
*Antrocephalus validicornis* (Holmgren), ♀, Vietnam, Núi Chúa N. P. **23** habitus lateral **24** mesonotum dorsal.

#### Antrocephalus decipiens

Taxon classificationAnimaliaHymenopteraChalcididae

(Masi, 1929)

Figs 9 , 10–11

Haltichella decipiens Masi, 1929a: 176 (lectotype (USNM No. 41887) designated by Narendran (1989), Philippines (examined)).
Antrocephalus decipiens ; Narendran 1989: 33–35.

##### Material

(RMNH, IEBR). 1 ♀, “S. **Vietnam**: Dóng Nai, Cát Tiên N. P. c. 100 m, 9. iv.–3. v. 2007, M. P. Quy, N. T. Manh & C. v. Achterberg, Malaise trap, RMNH’07”; 1 ♀, id., but 14–20.v.2007, Malaise traps 20–23, *Lagerstroemia* trail; 1 ♀, but 19–25.iv.2007; 1 ♀ ♀, “S. Vietnam: Dak Lak, Chu Yang Sin N. P., nr. dam, 800–900 m, Malaise trap, 2–10.vi.2007, C. v. Achterberg & R. de Vries, RMNH’07”; 1 ♀, “S. Vietnam: Ninh Thuân, Núi Chúa N. P., Northeast part, 23–30.v.2007, Malaise trap, C. v. Achterberg & R. de Vries, RMNH’07”.

##### Diagnosis.

In the key to Oriental species of *Antrocephalus* by Narendran (1989) *Antrocephalus decipiens* comes near *Antrocephalus nitidus* Narendran but differs from it in having: 1) interstices 0.5 × diameter of a pit on mesoscutum or less (in *Antrocephalus nitidus* interstices of pits on mesoscutum broader, often wider than half diameter of pits and shiny); 2) pits on mesoscutal disc much larger than those of *Antrocephalus nitidus*; 3) pubescence white or dirty white (in *Antrocephalus nitidus* golden yellow) and 4) basal carinae of T1 not longer than distance between them (in *Antrocephalus nitidus* basal carinae of T1 distinctly longer than distance between them).

##### Description.

♀, length of body 2.0–2.5 mm.

*Colour*. Black; femora and tibiae black with bases and apices pale brown or pale brownish yellow; scape black with basal half yellow in Vietnamese specimens, reaching anterior ocellus.

*Head*. Pre- and post-orbital carinae distinct.

*Mesosoma*. Pronotal carinae and tubercles absent; mesosoma with interstices narrower than half diameter of a pit and rugulose; apex of scutellum bi-lobed.

*Wings*. Fore wing with two black patches: one starting from MV and the other from anterior wing margin beyond PMV, both patches united each other medially encircling a white spot in between anteriorly.

*Legs*. Hind coxa with a carinate projection dorso-basally.

*Metasoma*. Metasoma longer than mesosoma; T1 with two parallel ridges or carinae at base; length of each carina subequal to width between them; T1 smooth and shiny dorsally.

##### Variation.

This is a very variable species. The colour of legs ranges from black to red. The black colour of hind femur is often brown or red or black with base and apex brown or red. In some specimens the dark infuscation of the fore wing is very faintly represented. In some specimens the antenna is completely black; in some others the basal funicular segments and scape (partly or completely) are reddish or brownish.

##### Hosts.

Unknown.

##### Distribution.

Widely distributed all over the Oriental region. New record for Vietnam.

#### Antrocephalus lugubris

Taxon classificationAnimaliaHymenopteraChalcididae

(Masi, 1932)

Figs 12–13

Tainania lugubris Masi, 1932b: 238 (♂, (DEI) Taiwan).
Antrocephalus lugubris ; Narendran 1977: 296 (new combination).
Tainania aceroscutellaris Sheng & Wen (in Sheng), 1989: 21 (Jiangxi; China (JXAU) (synonymised by Narendran and Padmasenan (1991) with Antrocephalus lugubris Masi).

##### Material.

1 ♀ (RMNH), “S. **Vietnam**: Dóng Nai, Cát Tiên N. P., ca 100 m, 14–20.v.2007, Mal. traps 20-23, *Lagerstroemia* tr[ail], C. v. Achterberg & R. de Vries, RMNH’07”; 3 ♂ (RMNH, IEBR), “S. Vietnam: Ninh Thuân, Núi Chúa N. P., northeast part, 90–150 m. 23–30.v.2007, Malaise trap, C. v. Achterberg & R. de Vries, RMNH’07”; 1 ♀ (BMBM), “Vietnam, [locality unknown], 7.xi.1986. C.M. Yoshimoto”.

##### Diagnosis.

This is a remarkable species with a characteristic apex of the scutellum.

##### Description.

♀♂: black; pre-orbital carina joins auricular carinae as in *Antrocephalus hakonensis* (Ashmead); apex of scutellum narrowly pointed with a split in the middle (Fig. 13), scutellum with a median longitudinal furrow; T1 without basal carinae but with a pit, callus with dense silvery pubescence; lateral tooth of propodeum well pronounced and directed upwards; hind coxa without a dorsal tooth; hind femur without an inner basal tooth.

##### Host.

Unknown.

##### Distribution.

India, Vietnam (new record), Singapore, Indonesia (Java), Philippines (Narendran 1989) and China (including Taiwan; Sheng and Wen 1989).

#### Antrocephalus maculipennis

Taxon classificationAnimaliaHymenopteraChalcididae

Cameron, 1905

Figs 14–15

Antrocephalus maculipennis Cameron, 1905: 95 (lectotype ♀ (BMNH No. Hym. 5, 258 on pin, selected by Narendran, 1989).

##### Material.

1 ♀ (RMNH), “S. **Vietnam**: Dak Lak, Chu Yang Sin N. P., Krong K’Mar, 590–840 m, 22–26.x.2005, Mal[aise] traps 13–23, C. v. Achterberg & R. de Vries, RMNH’05”.

##### Diagnosis.

This species comes near *Antrocephalus niger* (Masi) in the key to Oriental species of *Antrocephalus* by Narendran (1989), but differs from that species in having the fore wing with two dark brown or black infuscations whereas in *Antrocephalus niger* the fore wing has only one dark infuscation. This species also resembles *Antrocephalus cariniceps* (Cameron) in general appearance, but *Antrocephalus cariniceps* has a distinct long pair of basal carinae at the base of T1, whereas in *Antrocephalus maculipennis* such carinae are absent.

##### Description.

♀, length of body 5.6–6.6 mm. Black; fore wing with two brown patches: one starting from MV extending to middle or beyond posteriorly; second one extending from latter patch to posterior part of the wing. Body with silvery pubescence. Head wider than mesosoma (excluding tegulae); pre-orbital carina distinct, not joining auricular carina; post-orbital carina slightly indicated, malar groove carinate. Scape reaching anterior ocellus, apex of scutellum well emarginated; anterior pronotal carinae and median tubercles distinct; pits on mesosoma close and interstices narrower than half diameter of a pit and micro-sculptured; propodeum with postspiracular teeth present; metasoma acuminate towards apex.

##### Host.

*Opisina arenosella* Walker (Lepidoptera: Oecophoridae) (Pillai and Nair 1993).

##### Distribution.

India, East Malaysia (Sarawak) (Narendran 1989), Vietnam (new record).

#### Antrocephalus nasutus

Taxon classificationAnimaliaHymenopteraChalcididae

(Holmgren, 1868)

Figs 16–17

Haltichella nasuta Holmgren, 1868: 437 (lectotype, ♀ (NHRM), Philippines, Manila (lectotype designation by Narendran, 1989).
Antrocephalus rufipes Cameron, 1905: 95 (lectotype, ♀, Sarawak, Trusan (BMNH, No. 5-274), Narendran, 1989 designated and synonymised with Antrocephalus nasutus (Holmgren)).
Antrocephalus momius Masi, 1932a: 43 (lectotype, ♀, Formosa (= Taiwan), Kankau (DEI), Narendran, 1989 designated and synonymised with Antrocephalus nasutus (Holmgren)).
Antrocephalus nasutus ; Baltazar 1966: 145.
Antrocephalus longidentata Roy & Farooqi, 1984: 10 (♀, India, Orissa (INPC), synonymised with Antrocephalus nasutus by Narendran, 1989).

##### Material

(RMNH, IEBR). 1 ♀, “S. **Vietnam**: Dóng Nai, Cát Tiên N. P., *Ficus* trail, c. 100 m, 9–30.iv.2007, Malaise trap, M.P. Quy & N.T. Manh, RMNH’07”; 2 ♂, id., but 13–20.v.2005 and Botanical Garden; 1 ♀ + 2 ♂, “Vietnam: Ninh Thuân, Núi Chúa N. P., northeast part, 90–150 m, 23–30.v.2007, Malaise trap, C. v. Achterberg & R. de Vries, RMNH’07”; 1 ♂, “S. Vietnam: Dak Lak, Chu Yang Sin N. P., n[ea]r dam, c, 500 m, Malaise traps, 3–9.vi.2007, C. v. Achterberg & R. de Vries, RMNH’07”.

##### Diagnosis.

Similar to *Antrocephalus sepyra* (Walker) in general appearance, but differs from it in having the hind femur relatively longer than that of *Antrocephalus sepyra*, fore wing with brown infuscation behind MV and apex of scutellum deeply incised. It differs from *Antrocephalus fascicornis* (Walker) in having the face not as convex as that of *Antrocephalus fascicornis* and hind femur not as wide as that of *Antrocephalus fascicornis*. This species comes near *Antrocephalus atulyus* Narendran in the key to species of *Antrocephalus* by Narendran (1989), but *Antrocephalus atulyus* differs from this species in having 1) fore wing with golden pubescence (in *Antrocephalus nasutus* fore wing without golden pubescence); 2) interstices on pronotum and anterior part of mesoscutum smooth and shiny (in *Antrocephalus nasutus* interstices on pronotum and anterior part of mesoscutum rugulose) and 3) hind femur with prominent and long depression separating proximal and distal lobes (Fig. 16) (in *Antrocephalus nasutus* depression separating proximal and distal lobes shorter).

##### Host.

Unknown.

##### Distribution.

India, Indonesia (West Irian), Malaysia, Philippines, Singapore, Papua New Guinea (Narendran 1989) and Vietnam (new record).

##### Variation.

In Vietnamese specimens the fore wing has two patches and the hind tibia is blackish from base to middle and reddish on remaining part.

#### Antrocephalus neogalleriae sp. n.

Taxon classificationAnimaliaHymenopteraChalcididae

http://zoobank.org/91A4C675-FDD8-4E20-810A-146A3B19CF49

Figs 18 , 19–20

##### Type material.

Holotype, ♀ (RMNH), “S. **Vietnam**: Dóng Nai, Cát Tiên N. P., Dong trail, Mal. traps, c. 100 m, 1–8.iv.2007, Mai Phu Quy & Nguyen Tanh Manh, RMNH’07”.

##### Diagnosis.

This new species does not come close to any of the Oriental species of *Antrocephalus* described so far (Narendran 1989, Narendran and Sudheer 2005). It runs to *Antrocephalus galleriae* Subba Rao and *Antrocephalus maculipennis* Cameron in the key by Narendran (1989) but differs from them as follows: from *Antrocephalus galleriae* in having 1) metasoma distinctly shorter than mesosoma (in *Antrocephalus galleriae* metasoma distinctly longer than mesosoma); 2) metasoma 1.35 × as long as broad in dorsal view and not very acuminate posteriorly (in *Antrocephalus galleriae* metasoma 3 × as long as broad in dorsal view and acuminate posteriorly) and 3) fore wing with two infuscations (in *Antrocephalus galleriae* infuscations of fore wing absent).

This new species differs from *Antrocephalus maculipennis* in having: 1) pronotum with anterior carinae and tubercles absent (in *Antrocephalus maculipennis* pronotum with distinct anterior carinae and median tubercles); 2) PMV as long as MV (in *Antrocephalus maculipennis* PMV 1.5 × as long as MV) and 3) metasoma not acuminate posteriorly (in *Antrocephalus maculipennis* metasoma acuminate posteriorly.

##### Description.

Holotype, ♀, length of body 4.8 mm.

*Colour*. Black; eyes pale gray, ocelli pale reflecting gray; antenna black except pedicel; anellus and F1 light pink; maxillary stipes and galea pale yellow; maxillary palp pale brown; teeth of mandibles dark brown; tegula dark brown basally separated from pale yellow distal part by a pink brown median part; all coxae and hind femora black; for femur, mid femur and mid tibia yellowish red; fore tibia and hind tibia brownish red; fore and mid tarsi pale yellow, hind tarsi reddish brown; all telotarsi dark; remaining parts of mesosoma and metasoma black; wings hyaline with two dark brown infuscation: one behind MV and other distal to STV; MV and STV black, PMV brown, SMV pale yellowish brown.

*Head*. Width of head 1.2 × its height in anterior view, 2.2 × as wide as long in dorsal view, subequal to width of mesosoma; scrobe reaching anterior ocellus, cross striate; pre-orbital carina running upwards joining each other behind anterior ocellus; post-orbital carina running upwards to vertex; malar ridge and auricular carinae present; eyes with widely scattered minute pubescence; malar space 0.5 × eye height in profile; eye length 0.7 × eye height in profile; geno-temporal furrow absent; POL 4. 8x OOL; AOL twice OOL; LOL subequal to OOL; width of interocular distance twice POL; each posterior ocellus with a characteristic deep pit attached to it; face and vertex with deep close setigerous pits; face and gena with moderate pubescence; scape reaching anterior ocellus; relative L:W of antennal segments: scape = 31:5; pedicel = 8:4; F1 = 7:5; F2 = 7:5; F3 = 6:6; F4 = 6:6; F5 = 5:6; F6 = 5:6; F7 = 5:6; clava = 11:6.

*Mesosoma*. Pronotum with anterior transverse carina present only on sides, median part ecarinate; surface closely punctate with interstices narrow, ecarinate, rugulose; posterior margin of pronotum concave medially; spiracular part with dense silvery pubescence; mesoscutum similarly pitted as in pronotum; tegula rugulose and pubescent; scutellum closely punctate as in mesoscutum and pronotum; interstices narrow and rugulose with a median fovea; apex of scutellum weakly emarginate; axillae densely pubescent. Propodeum subhorizontal with distinct submedian carinae, surface areolate and punctate with dense silvery pubescence on either side.

*Wings*. Fore wing 2.9 × as long as wide, relative length of CC = 29; SMV = 21; MV = 6; PMV = 6; STV = 2.

*Legs*. Hind coxa with a prominent dorso-basal tooth; hind femur without an inner basal tooth, ventral margin bi-lobed, with a comb of teeth; hind tibia closely reticulate granulate.

*Metasoma*. Sessile, shorter than mesosoma (25:18); T1 a little exceeding middle of metasoma, with a pit basally, smooth and shiny; T2 to T5 micro-sculptured and rugulose with pubescence on sides; T6 densely rugulose and punctate, fully pubescent, 1.5 × as long as epipygium; visible part of ovipositor sheath in dorsal view 0.5 × length of epipygium.

*Male*. Unknown.

##### Host.

Unknown.

#### Antrocephalus sepyra

Taxon classificationAnimaliaHymenopteraChalcididae

(Walker, 1846)

Figs 21–22

Halticella sepyra Walker, 1846: 110 (India (lectotype selected by Bouček) (HDOU) (examined); synonymy by Narendran (1989) with Antrocephalus dividens (Walker)).
Chalcis dividens Walker, 1860: 357 (♀ Sri Lanka (BMNH) examined); junior synonym of Halticella sepyra Walker.
Halticella apicalis Walker, 1874: 400. (♀, Japan (BMNH) (examined); synonymy by Narendran (1989) with Antrocephalus dividens (Walker)).
Halticella (Stomatoceras) tinctipennis Cameron, 1888: 118 (Japan (BMNH) (examined); synonymy by Habu (1960) with Antrocephalus apicalis (Walker)).
Coelochalcis carinifrons Cameron, 1904: 111 (♂, India, Sikkim (BMNH) (examined); synonymy by Narendran (1989 with Antrocephalus dividens (Walker)).
Antrocephalus varipilosus Cameron, 1907: 580 (♂, India, Gujarat, Dredra (BMNH) examined; synonymy by Narendran (1989) with Antrocephalus dividens (Walker)).
Sabatiella nepalensis Mani & Dubey (in Mani et al.), 1974: 21 (♀, Nepal, Kathmandu (USNM) (examined); synonymy by Narendran (1989) with Antrocephalus dividens (Walker)).
Antrocephalus delhiatus Roy & Farooqi, 1984: 9 (♀, India, Delhi (INPC) (examined); synonymy by Narendran (1989) with Antrocephalus dividens (Walker)).

##### Material

(RMNH, IEBR, labelled as *Antrocephalus dividens*, *Antrocephalus fascicornis* or *Antrocephalus japonicus* by first author). 4 ♀ + 7 ♂, “**Vietnam**: Ninh Thuân, Núi Chúa N. P., northeast part, Malaise trap, 90–150 m, 23–30.v.2007, C. v. Achterberg & R. de Vries, RMNH’07”; 1 ♀ + 1 ♂, “S. Vietnam: Dóng Nai, Cát Tiên N. P., c 100 m, 13–20.v.2007, Botanical Garden, Malaise traps 14–19, C. v. Achterberg & R. de Vries, RMNH’07”; 2 ♀ + 1 ♂, id., but 14–20.v.2007, Malaise traps 20–23, *Lagerstroemia* trail; 1 ♀, id., but 9–30.iv.2007, *Ficus* trail; 1 ♀, id., but Dong trail, 9. iv.–9.v.2007, M.P. Quy, N.T. Manh & C. v. Achterberg, RMNH’07; 1 ♂, id., but 13–19.v.2007; 1 ♂, id., but 19–25.iv.2007; 2 ♂, id., but 1–8.iv.2007, Mai Phu Quy & Nguyen Tanh Manh; 1 ♀, “S. Vietnam: Dak Lak, Chu Yang Sin N. P., near dam, c. 500 m, 3–9.vi.2007 & 1–10.vi.2007, Malaise trap, C. v. Achterberg & R. de Vries, RMNH’07”; 1 ♂, id., but near river, c. 740 m, 1–10.vi.2007; 1 ♂, id., but Krong K’Mar, 740–900 m, 2–10.vi.2007; 1 ♂ (RMNH), “C. Vietnam: Ha Tinh, Vu Quang N. P., 111 m, , 18°19'40”N 105°26'29”E, 23.ix.-5.x.2009, Mal[aise] trap 23, C. v. Achterberg & R. de Vries, RMNH’09”; 1 ♂ (RMNH), “C. Vietnam: Ha Tinh, Vu Quang N. P., 1.x.2009, by hand, R. de Vries, RMNH’09”.

##### Diagnosis.

*Antrocephalus sepyra* (Walker) comes very near *Antrocephalus fascicornis* (Walker) in general appearance such as colour and sculpture on mesosoma. However, *Antrocephalus fascicornis* differs from *Antrocephalus sepyra* in having: 1) hind femur with a long row of comb teeth occupying a trifle shorter than three-fourth of outer ventral margin from apex to base (in *Antrocephalus sepyra* the length of row of comb teeth is shorter); and 2) head in profile with a sharp angle in front of eyes. (in *Antrocephalus sepyra* no such sharp angle in front of eyes in profile).

##### Description.

♀, length of body 4.0–5.5 mm.

*Colour*. Head black; eyes pale yellow or pale yellowish gray or black; tegula reddish brown or pale yellowish brown; all legs except black fore coxa ferruginous red; metasoma black; pedicel, ring segment and F1 pale in some specimens.

*Head*. Scape not reaching anterior ocellus.

*Mesosoma*. Pronotum with anterior carinae and tubercles; apex of scutellum weakly emarginated.

*Legs*. Hind coxa without a tooth dorsally; hind femur without an inner basal tooth.

*Metasoma*. Metasoma about as long as combined length of head and mesosoma. T1 smooth and shiny with a pair of short basal carinae.

*Male*. Antenna longer than that of ♀; metasoma shorter than head plus mesosoma.

##### Variation.

Colour of hind tibia varies from reddish brown to black (except apically) as emargination of apex of scutellum. Colour of hind femur is black with base and apex reddish brown and pedicel to F5 reddish brown in one specimen (labelled as “*Antrocephalus japonicus*”).

##### Host.

Unknown.

##### Distribution.

India, Nepal, Sri Lanka, Japan, Vietnam (new record).

#### Antrocephalus validicornis

Taxon classificationAnimaliaHymenopteraChalcididae

(Holmgren, 1868)

Figs 23–24

Haltichella validicornis Holmgren, 1868: 438 (♂, Java (NHRM); lectotype designated by Narendran 1989).
Antrocephalus validicornis ; Narendran 1989: 45.

##### Material.

1 ♀ (RMNH), “**Vietnam**: Ninh Thuân, Núi Chúa N. P., northeast part, Mal[aise] traps, 90–150 m, 23–30.v.2007, C. v. Achterberg & R. de Vries, RMNH’07”.

##### Diagnosis.

*Antrocephalus validicornis* comes near *Antrocephalus phaeospilus* Waterston in the key to species provided by Narendran (1989), but differs from it in having: 1) hind femur more than twice as long as wide (in *Antrocephalus phaeospilus* less than twice as long as wide); 2) hind femur black with base and apex red (in *Antrocephalus phaeospilus* completely red); 3) apex of scutellum only emarginate; not bi-lobed (in *Antrocephalus phaeospilus* apex of scutellum bi-lobed) and 4) eyes bare (in *Antrocephalus phaeospilus* eyes setose). *Antrocephalus validicornis* superficially resembles *Antrocephalus atulyus* Narendran (Narendran, 1989), but differs from it in having: 1) comb of teeth on ventral margin of hind femur starting near middle length of femur to apex (in *Antrocephalus atulyus* comb of teeth starts well before middle length of femur); 2) interstices of pronotum and of anterior part of mesoscutum rugose and somewhat carinate (in *Antrocephalus atulyus* interstices mostly smooth and shiny); 3) apex of scutellum emarginate (in *Antrocephalus atulyus* apex of scutellum bi-lobed) and 4) fore wing without any brown or black infuscation (in *Antrocephalus atulyus* fore wing with brownish infuscation adjoining MV). Among Vietnamese species of *Antrocephalus*, *Antrocephalus validicornis* comes near *Antrocephalus nasutus* (Holmgren) in having PMV distinctly longer than MV, but differs from it in having: 1) hind femur with ventral comb of teeth starting from middle length of hind femur, occupying 0.5 × length of femur (in *Antrocephalus nasutus* comb of teeth starts well before middle length of hind femur, occupying 0.7 × length of femur); 2) apex of scutellum shallowly emarginate (in *Antrocephalus nasutus* apex of scutellum deeply incised) and 3) hind femur black with base and apex red (in *Antrocephalus nasutus* hind femur red).

##### Description.

♀♂. Head with post-orbital carinae indistinct or absent; geno-temporal furrow distinct; eyes bare; interstices of pronotum and anterior part of mesoscutum rugose and somewhat carinate; apex of scutellum carinate; fore wing without black or brown infuscation; PMV distinctly longer than MV; hind femur black with base and apex red, its length more than twice its maximum width; hind femur without an inner basal tooth; metasoma with two carinae on T1, carinae much shorter than 1.25 × T1.

##### Host.

Unknown.

##### Distribution.

Vietnam (new record), Indonesia (Java), Malaysia (Narendran 1989).

#### Brachymeria

Taxon classificationAnimaliaHymenopteraChalcididae

Westwood, 1829

Figs 25 , 26 , 27–28 , 29–30 , 31–32 , 33–35 , 36–37 , 38–39 , 40–41 , 42–43 , 44–45 , 46–47 , 48–50 , 51 , 52–53 , 54–55 , 56 , 57–58 , 59 , 60–61 , 62 , 63–64 , 65 , 66–67 , 68–70

Brachymeria Westwood (in Stephens), 1829: 36. Type species: Chalcis minuta Fabricius; designated by Westwood, 1839.
Thaumatelia Kirby, 1883: 60. Type species: Chalcis separata Walker, by monotypy (synonymised with Brachymeria Westwood by Halstead 1991).
Oncochalcis Cameron, 1904: 162. Type species: Oncochalcis marginata Cameron, by monotypy; (synonymised with Brachymeria Westwood by Nikol’skaya 1960).
Holochalcis Kieffer, 1905: 258. Type species: Holochalcis madagascariensis Kieffer, by subsequent designation of Gahan and Fagan (1923); (synonymised with Brachymeria Westwood by Narendran (in Subba Rao & Hayat), 1987b).
Tumidicoxa Girault, 1911: 378. Type species: Tumidicoxa nigra Girault; by original designation; (synonymised with Brachymeria Westwood by Girault (1913)).
Thaumatelia Kirby, 1883: 60. Type species Chalcis separata Walker, by monotypy; (synonymised with Brachymeria Westwood by Halstead 1991).
Thaumateliana Girault, 1912: 160–161. Type species: Thaumateliana bicolor Girault, by monotypy; (synonymised with Thaumatelia Kirby by Narendran and Verghese 1989).
Pseudepitelia Girault, 1913: 104. Type species: Pseudepitelia rubrifemur Girault, by original designation; (synonymised with Brachymeria Westwood by Girault 1915a).
Brachepitelia Girault, 1913a: 106. Type species: Brachepitelia rubripes Girault, by original designation and monotypy; (synonymised with Brachymeria Westwood by Girault (1915a)).
Tumidicoxoides Girault, 1913b: 86. Type species: Tumidicoxoides kurandaensis Girault, by original designation; (synonymy with Brachymeria Westwood by Girault (1926)).
Tumidicoxella Girault, 1913c: 74. As a subgenus of Tumidicoxa; type species: Tumidicoxa (Tumidicoxella) nigra Girault by original designation.
Microchalcis Girault, 1915a: 328. Type species: Microchalcis atricorpus Girault by original designation; (synonymised by Bouček, 1988).
Dirrhinomorpha Girault & Dodd (in Girault 1915a): 327. Type species: Dirrhinomorpha angusta Girault & Dodd, by original designation; (synonymised with Brachymeria Westwood and treated as subgenus of Brachymeria by Bouček 1988).
Meyeriella Krausse, 1917: 95. Type species: Meyeriella indica Krausse, by monotypy (synonymised with Brachymeria Westwood by Narendran, 1986).
Neobrachymeria Masi, 1929c: 196–198. As a subgenus of Brachymeria. Type species: Brachymeria confalonierii Masi by original designation.
Australochalcis Girault, 1939: 326. Type species: Australochalcis humilicrus Girault, by original designation and monotypy (listed as synonym of Brachymeria Westwood by Bouček, 1988).
Matsumurameria Habu, 1960: 209. As a subgenus of Brachymeria. Type species: Chalcis taiwanus Matsumura, by original designation.
Gahanula Burks, 1960: 261. Type species: Brachymeria discreta Gahan, by original designation (as a subgenus of Brachymeria).

##### Diagnosis.

Head oval in profile; scrobe deep with carinate margins; in some species head with pre-orbital or post-orbital carinae or with both carinae present; malar sulcus carinate or ridged; antennal formula 11171 (clava 1 to 3 segmented). Mesosoma with umbilicate punctures; fore wing with PMV usually half or about half as long as MV and usually twice as long as STV. Hind coxa in ♀ in some cases with an inner ventro-mesal tooth; hind femur with a ventral row of irregular teeth and in some species with an inner basal tooth; hind tibia arcuate; metasoma sessile, T1 always the longest; ovipositor sheath slightly compressed and slightly exserted; in some species metasoma elongate.

##### Hosts.

The species are mostly primary parasitoids in pupae of holometabolous insects, especially of Lepidoptera, but some species attack Diptera, Coleoptera or Hymenoptera. Most species are primary parasitoids, but some are hyperparasitoids attacking parasitoid Hymenoptera or Diptera in Lepidoptera.

##### Distribution.

Worldwide.

##### Key to Vietnamese species of *Brachymeria* Westwood

**Table:** 

1	Postclypeus hardly or not differentiated from face and densely pubescent and groove absent (only smooth short anteclypeus visible); hind coxa with a trichoid zone on ventral side; [hind tibia completely yellow with base and ventral margin above black]	***Brachymeria taiwana* Habu**
–	Postclypeus distinctly differentiated from face and sparsely pubescent; hind coxa without a trichoid zone	**2**
2	Inner side of hind femur with a small basal tubercle	**3**
–	Inner side of hind femur without a basal tubercle	**4**
3	Hind femur 1.8–2.1 times as long as wide; dorsal side of hind femur in lateral view dilated straightly from base to widest part, hence also straightly contracted towards apex so that dorsal side is weakly angulate; apical whitish patch on hind femur is generally limited on outer dorsal side, not extending to inner side	***Brachymeria podagrica* (Fabricius)**
-	Hind femur less than 1.7 times as long as wide; dorsal side of hind femur in lateral view not dilated, but rounded from base to apex; apical yellow patch extend to inner side	***Brachymeria minuta* (Linnaeus)**
4	Inner side of hind coxa of ♀ with a minute tooth ventrally	**5**
–	Inner side of hind coxa of ♀ without minute tooth ventrally	**6**
5	Hind tibia yellow with base and ventral margin black (Fig. 7); scape longer, reaching level of anterior ocellus (Fig. 46)	***Brachymeria lasus* (Walker)**
–	Hind tibia black with more or less yellow subbasal area and yellow apical area dorso-laterally (Fig. 33); scape shorter, remaining distinctly below level of anterior ocellus (Fig. 33)	***Brachymeria coxodentata* Joseph, Narendran & Joy**
6	Post-orbital carina absent	**7**
–	Post-orbital carina present	**10**
7	Hind tibia entirely yellow, except its dark ventral carina; T1 densely finely punctate and with satin sheen	***Brachymeria carinata* Joseph, Narendran & Joy**
–	Hind tibia bicoloured medially or largely black or reddish black basally; T1 smooth or largely so and strongly shiny	**8**
8	Hind tibia yellow medially and only basally reddish black or black; apex of scutellum weakly emarginate	***Brachymeria margaroniae* Joseph, Narendran & Joy**
–	Hind tibia with blackish or brown band medially; apex of scutellum rounded	**9**
9	Second hind tarsal segment distinctly longer than wide in dorsal view (Fig. 70); hind tibia with brownish band medially (Fig. 68)	***Brachymeria shansiensis* Habu**
–	Second hind tarsal segment hardly longer than wide in dorsal view (Fig. 38); hind tibia with blackish band medially (Fig. 38)	***Brachymeria excarinata* Gahan**
10	Pre-orbital carinae raised and converge to join with scrobal margin; [scutellum with a single median ridge or carina; hind tibia completely yellow]	***Brachymeria scutellocarinata* Joseph, Narendran & Joy**
–	Pre-orbital carina absent **or** if present and joining scrobal margin then not raised	**11**
11	Hind tibia entirely yellow except ventral carina ventrally	**12**
–	Hind tibia at least ventrally and part of inner side black or dark brown	**14**
12	Setae dorsally on mesosoma and metasoma golden yellow and dense (Fig. 28); metasoma about as long as pronotum, mesoscutum and scutellum combined (Fig. 27)	***Brachymeria aurea* Girault**
–	Setae dorsally on mesosoma and metasoma silvery or greyish or pale yellow (Fig. 26); metasoma slightly shorter than pronotum, mesoscutum and scutellum combined (Fig. 29)	**13**
13	Hind femur with large black medial patch and remainder yellow; posterior lamella of scutellum emarginated medio-posteriorly	***Brachymeria megaspila* (Cameron)**
–	Hind femur black except an ivory patch apically (Fig. 52); posterior lamella of scutellum evenly convex medio-posteriorly (Fig. 53)	***Brachymeria marmonti* (Girault)**
14	Hind femur red or brownish red with its apex yellow (rarely hind femur red with black patch medially in some extralimital specimens); apex of scutellum emarginate and with dense silvery pubescence; hind tibia yellow with its base and ventral side black or dark brown	***Brachymeria bengalensis* (Cameron)**
–	Hind femur black with its apex pale yellow; if hind femur red then without yellow or pale part apically; apex of scutellum without dense silvery pubescence and convex to distinctly emarginate medio-posteriorly; colour of hind tibia variable	**15**
15	Metasoma with golden pubescence; remainder of body with white or grey pubescence; metasoma red but T1 medially and T2 to T5 anteriorly black; hind tibia black with weak brown subbasal spot and its apex yellow	***Brachymeria semirusula* sp. n.**
–	Metasoma with silvery or grey or white pubescence; metasoma completely black; colour of hind tibia variable	**16**
16	Hind tibia yellow with its base and ventral side black or dark brown	**17**
–	Hind tibia with different pattern, mostly black or black with its base and apex pale	**18**
17	Metasoma globose or subglobose in lateral view (Fig. 36); clava more than twice as long as preceding segment	***Brachymeria euploeae*** (**Westwood)**
–	Metasoma acuminate posteriorly in lateral view (Fig. 42); clava shorter than twice length of preceding segment	***Brachymeria jambolana* Gahan**
18	Scutellum with a weak median carina or smooth ridge; [body black with inner side of fore and mid tibiae blackish brown and minute tan spot at apex of hind femur; hind tibia completely black; metasoma acuminate posteriorly and T1 faintly sculptured]	***Brachymeria lugubris* (Walker)**
–	Scutellum without median carina or smooth ridge	**19**
19	Metasoma globose or subglobose in lateral view (Fig. 44); hind tibia black with a small subbasal pale spot and apically with yellow patch (Fig. 45)	***Brachymeria kamijoi* Habu**
–	Metasoma acuminate in lateral view (Figs 40, 48, 56); colour of hind tibia variable	**20**
20	Hind femur red, without yellowish spot apically (Fig. 25); pre-orbital carina hardly indicated; hind tibia ferruginous or black with base and apex reddish brown	***Brachymeria alternipes* (Walker)**
–	Hind femur black or dark brown and with yellow spot apically (Figs 40, 48, 56); pre-orbital carina present; colour of hind tibia variable	**21**
21	Apex of scutellum rounded (Figs 41, 50)	**22**
–	Apex of scutellum weakly emarginate to distinctly incised medio-posteriorly (fig. 57)	**23**
22	Scape slightly longer than combined length of F1 to F4; scrobe not reaching anterior ocellus (Fig. 49); area below scrobe with a distinct raised portion	***Brachymeria longiscaposa* Joseph, Narendran & Joy**
–	Scape about as long as combined length of F1 to F4; scrobe reaching anterior ocellusc; area below scrobe without a raised smooth part	***Brachymeria hime* Habu**
23	Base of hind tibia black; scape longer than combined length of F1 to F3 but not equal or exceeding combined length of F1 to F4; [scrobe not reaching anterior ocellus; pre- and post-orbital carinae present; apex of scutellum weakly emarginated]	***Brachymeria olethria* (Waterston)**
–	Base of hind tibia black, pale yellowish or yellowish brown; scape either shorter than combined length of F1 to F3 or at the most equal to combined length of F1 to F3	**24**
24	Scape shorter than combined length of F1 to F3; upper margin of clypeus smoothly curved and not angulate medially; clava as long as wide; apex of scutellum distinctly emarginate and bi-lobed (Fig. 57)	***Brachymeria neowiebesina* sp. n.**
–	Scape as long as combined length of F1 to F3; upper margin of clypeus angulate medially; clava slightly longer than 1.5 × its width; apex of scutellum weakly emarginate	***Brachymeria wiebesina* Joseph, Narendran & Joy**

**Figure 25.**
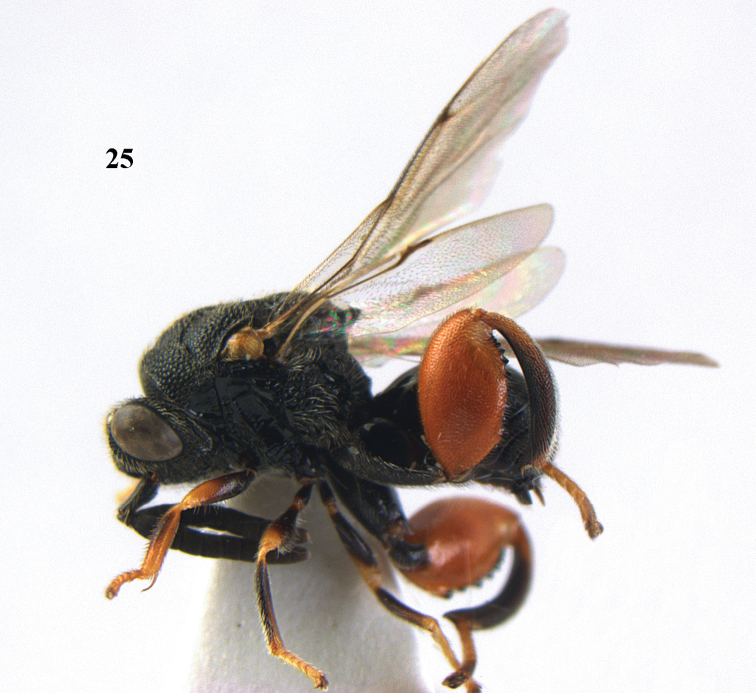
*Brachymeria alternipes* (Walker), ♂, Vietnam, Hoang Lien N. R., habitus, lateral.

**Figure 26.**
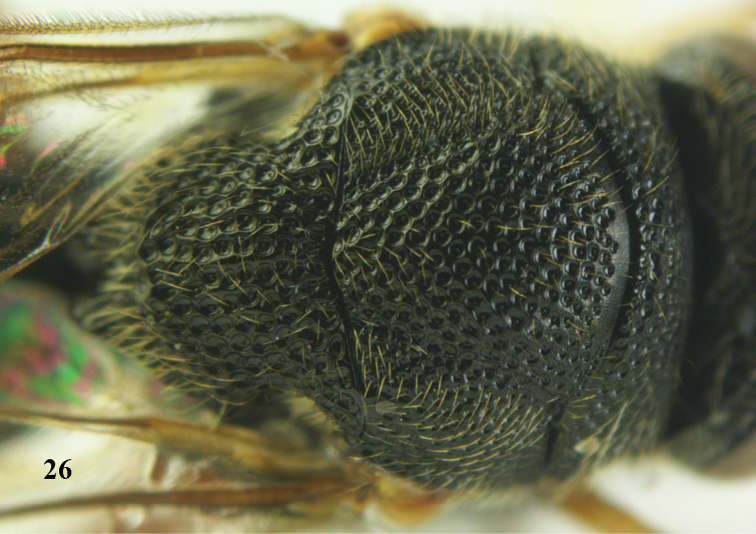
*Brachymeria alternipes* (Walker), ♂, Vietnam, Hoang Lien N. R., mesonotum, dorsal.

**Figures 27–28.**
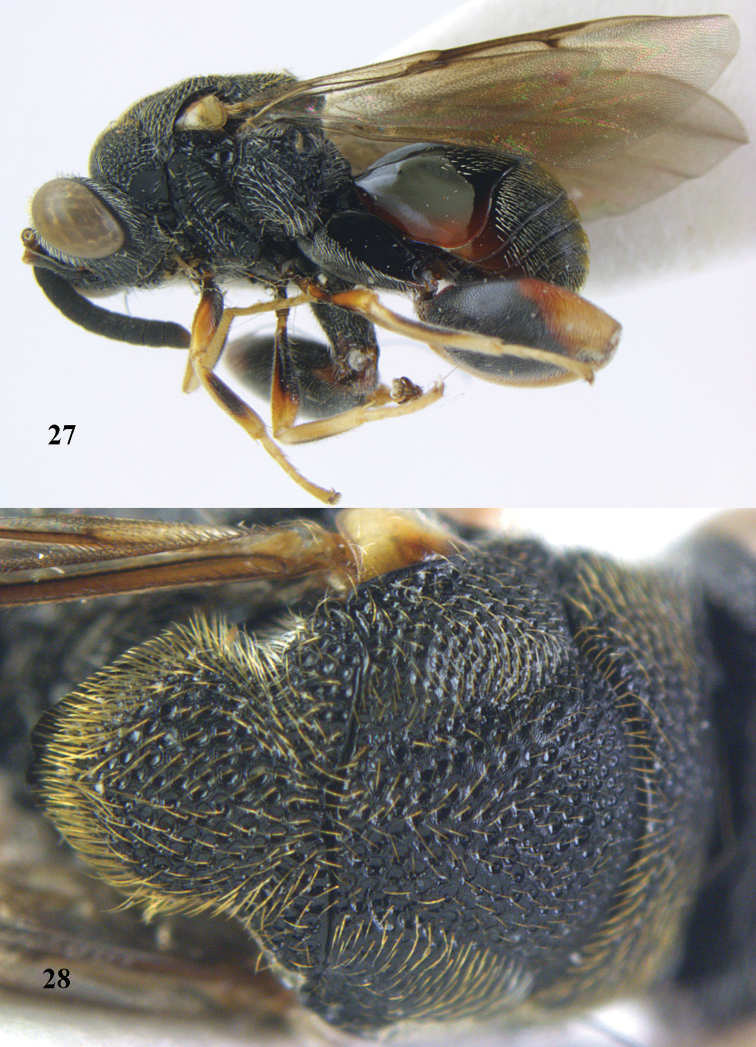
*Brachymeria aurea* (Girault), ♀, Vietnam, Cát Tiên N. P. **27** habitus lateral **28** mesonotum dorsal.

**Figures 29–30.**
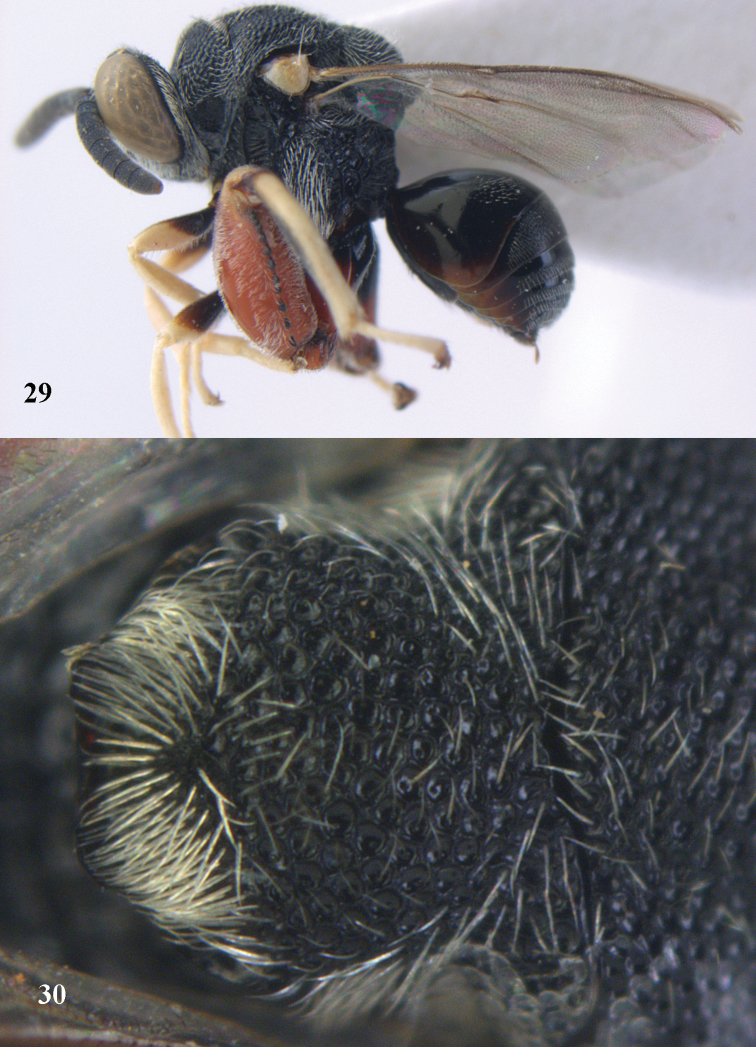
*Brachymeria bengalensis* (Cameron), ♀, Vietnam, Núi Chúa N. P. **29** habitus lateral **30** scutellum dorsal.

**Figures 31–32.**
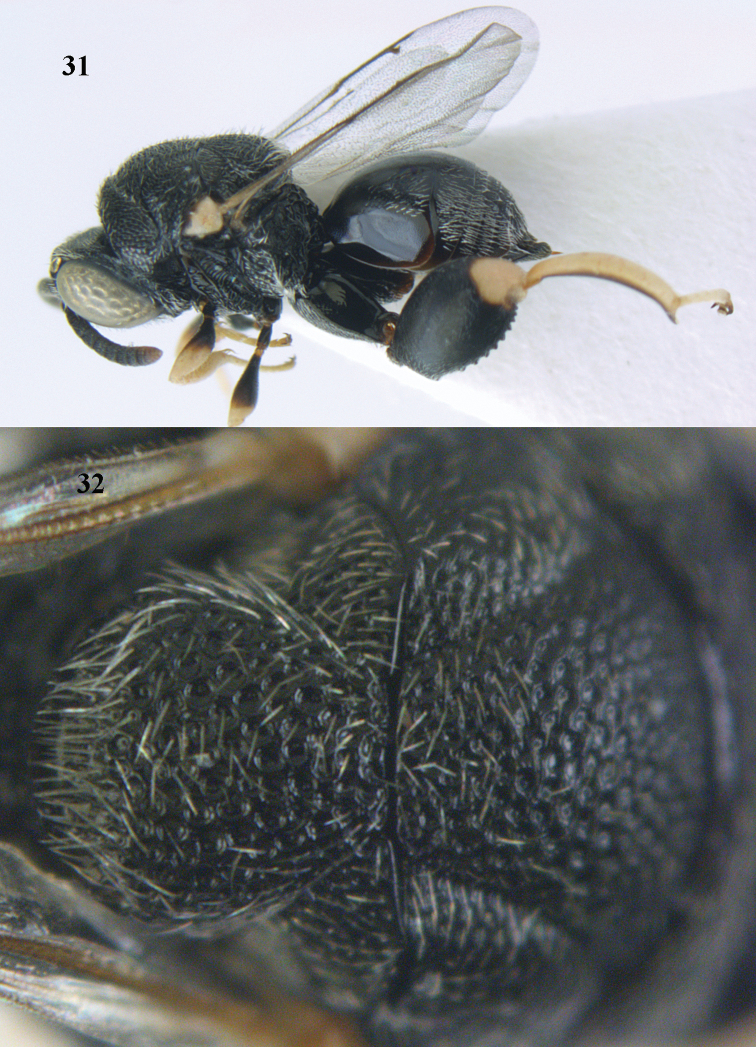
*Brachymeria carinata* Joseph, Narendran & Joy, ♀, Vietnam, Núi Chúa N. P. **31** habitus lateral **32** scutellum, dorsal.

**Figures 33–35.**
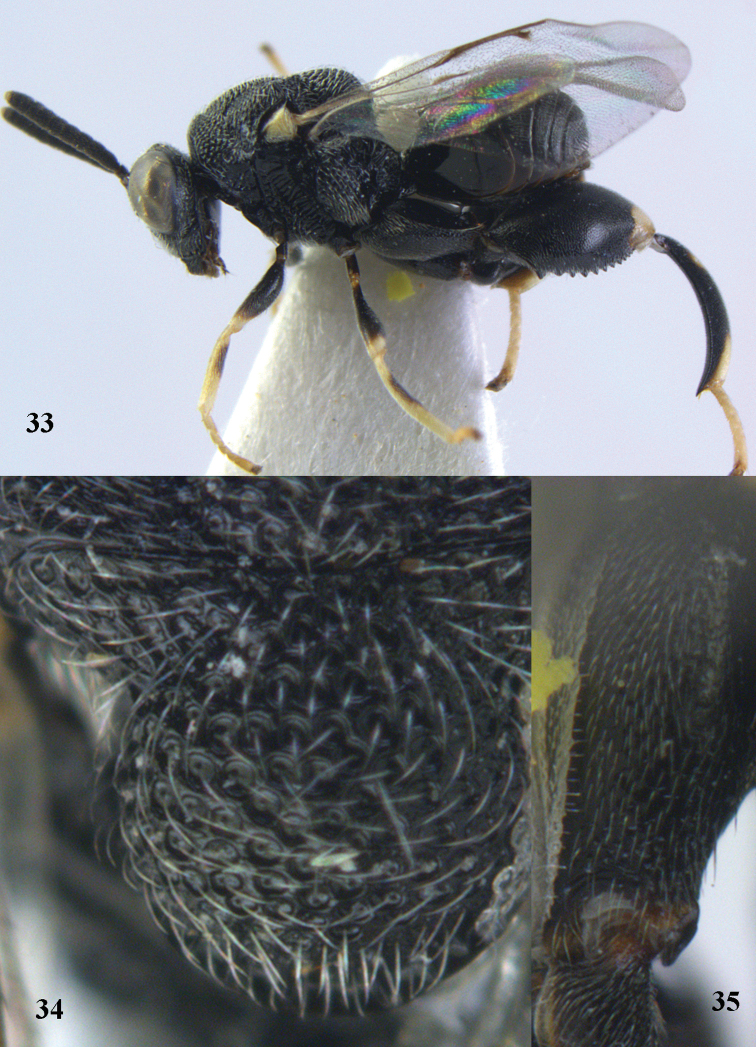
*Brachymeria coxodentata* Joseph, Narendran & Joy, ♀, Vietnam, Cat Ba N. P. **33** habitus lateral **34** scutellum dorsal **35** hind coxa ventral.

**Figures 36–37.**
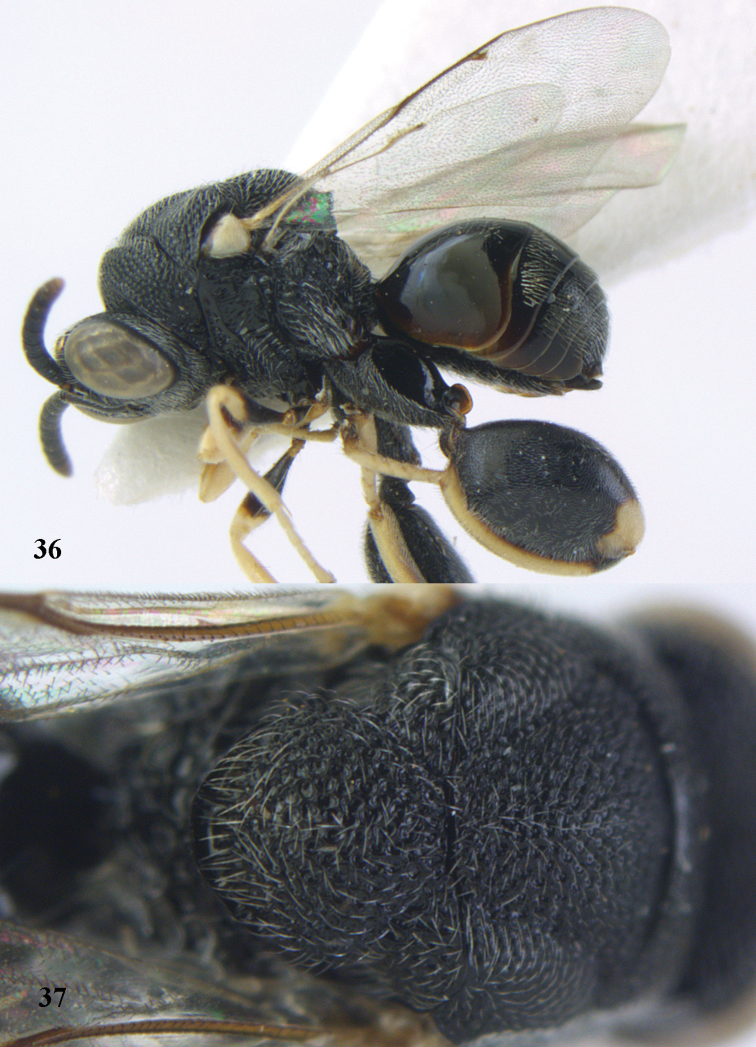
*Brachymeria euploeae* (Westwood), ♀, Vietnam, Chu Yang Sin N. P. **36** habitus lateral **37** scutellum dorsal.

**Figures 38–39.**
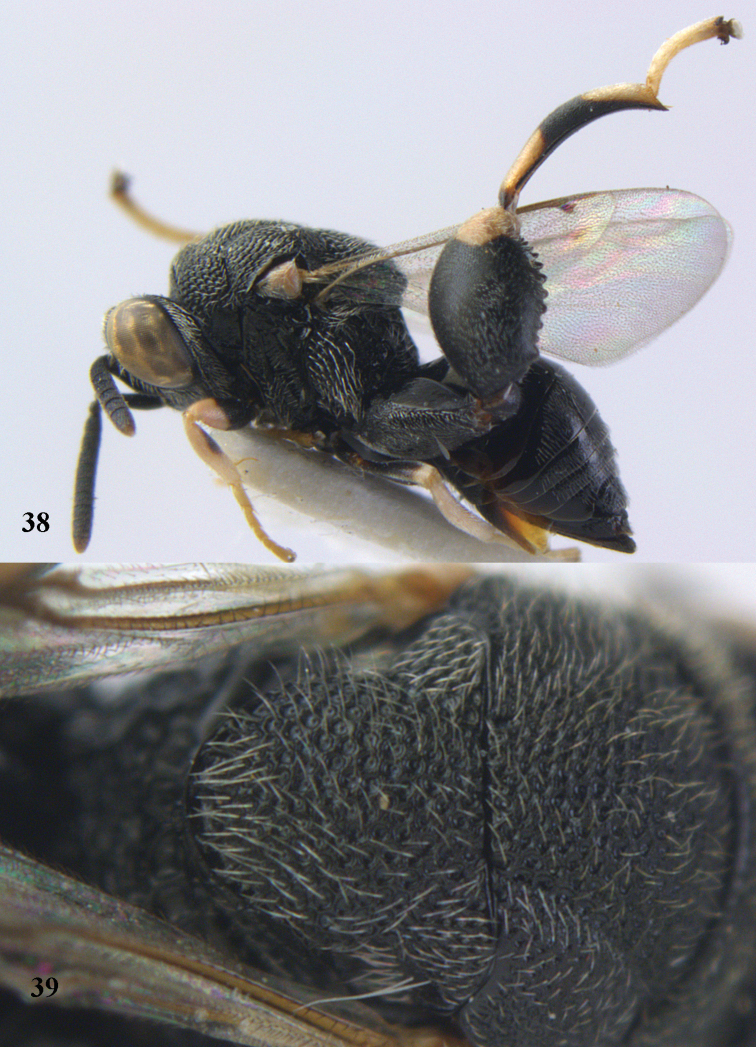
*Brachymeria excarinata* Gahan, ♀, Vietnam, Cát Tiên N. P. **38** habitus lateral **39** scutellum dorsal.

**Figures 40–41.**
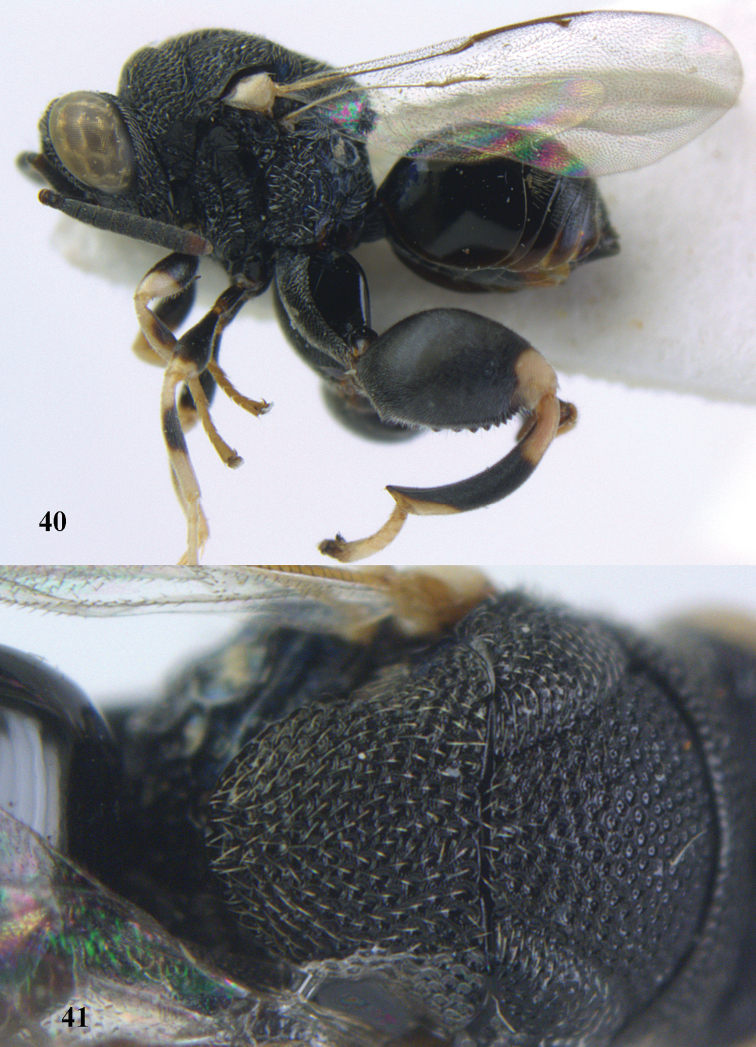
*Brachymeria hime* Habu, ♀, Vietnam, Cát Tiên N. P. **40** habitus lateral **41** scutellum dorsal.

**Figures 42–43.**
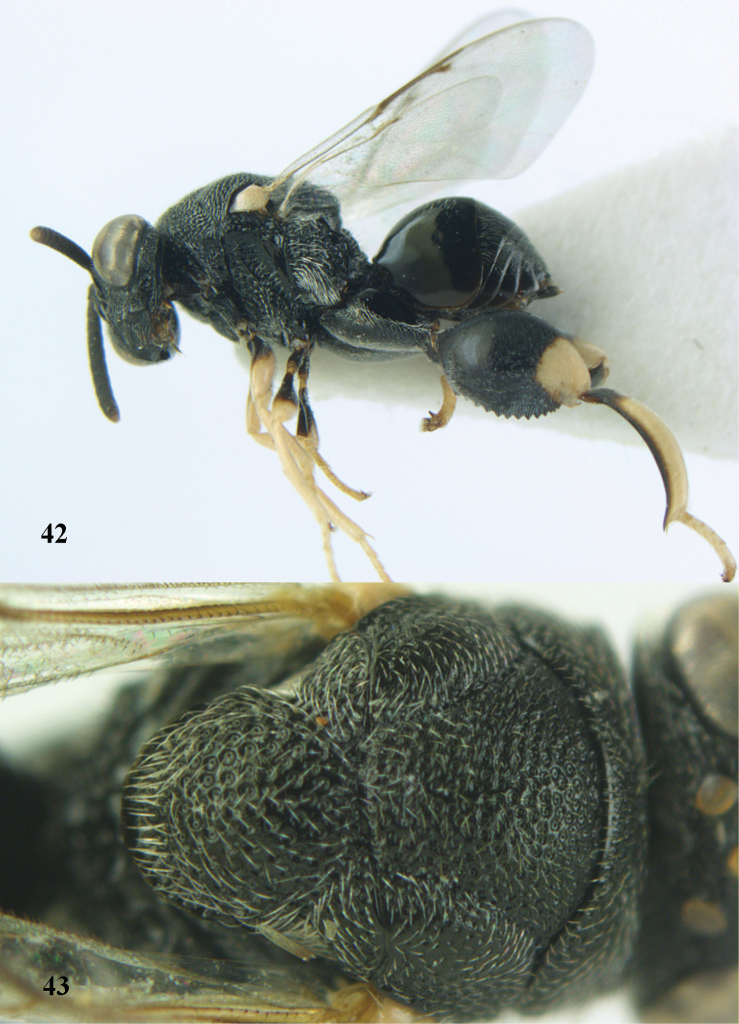
*Brachymeria jambolana* Gahan, ♀, Vietnam, Cát Tiên N. P. **42** habitus lateral **43** mesonotum dorsal.

**Figures 44–45.**
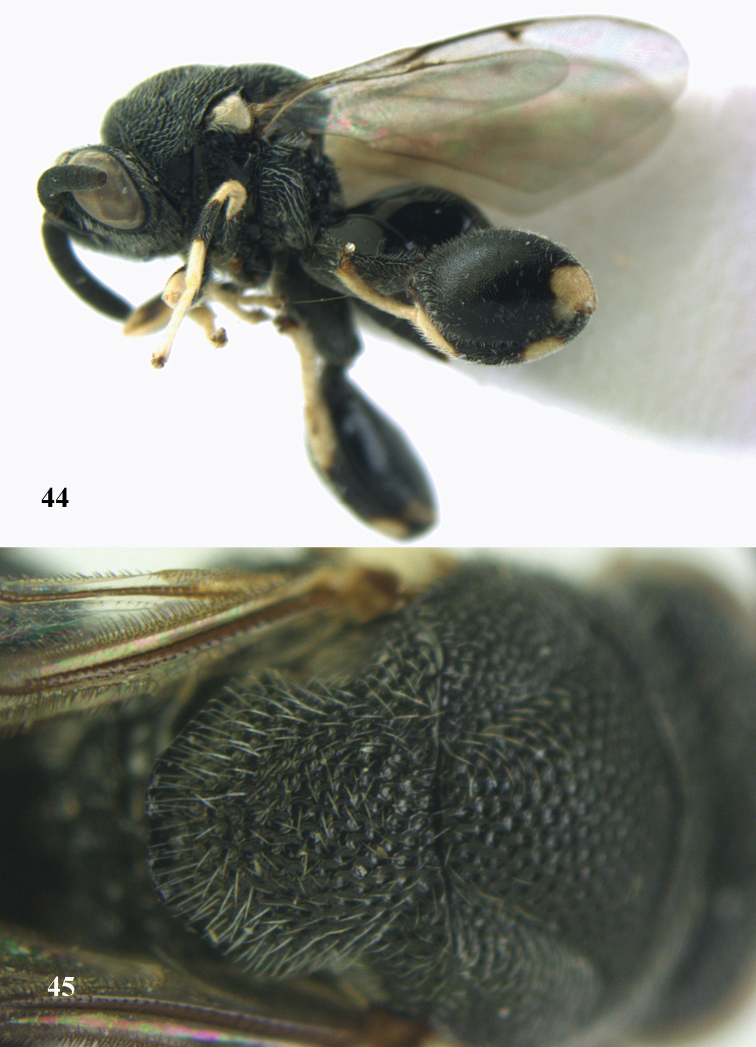
*Brachymeria kamijoi* Habu, ♀, Vietnam, Chu Yang Sin N. P. **44** habitus lateral **45** mesonotum dorsal.

**Figures 46–47.**
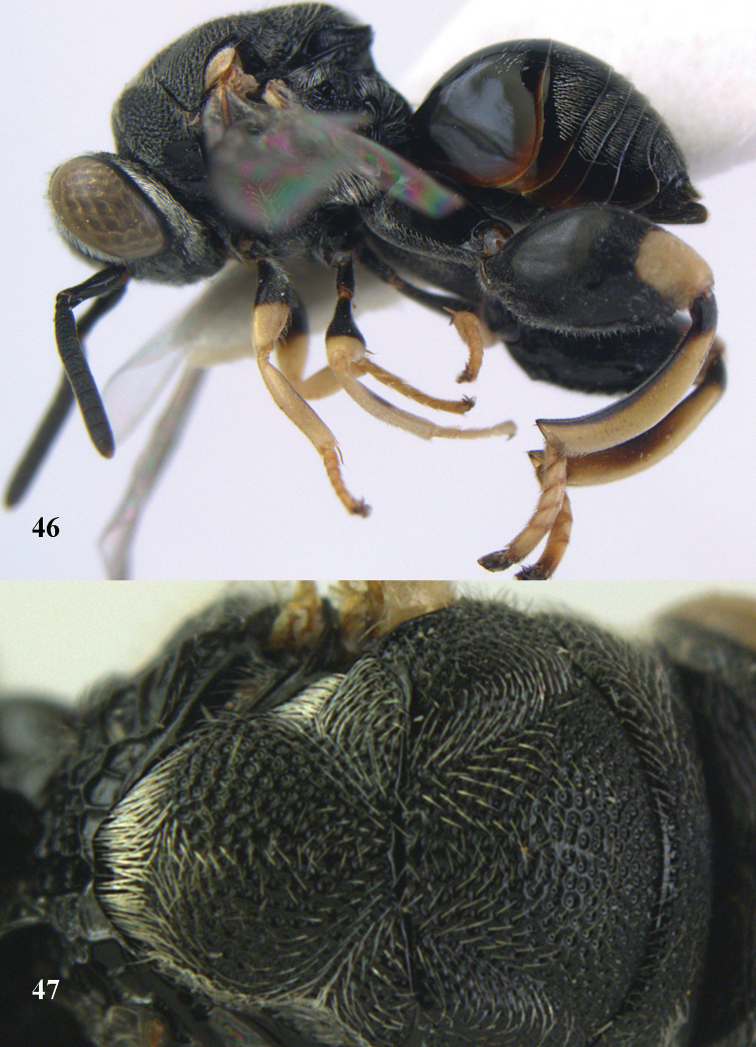
*Brachymeria lasus* (Walker), ♀, Vietnam, Cát Tiên N. P. **46** habitus lateral **47** mesonotum dorsal.

**Figures 48–50.**
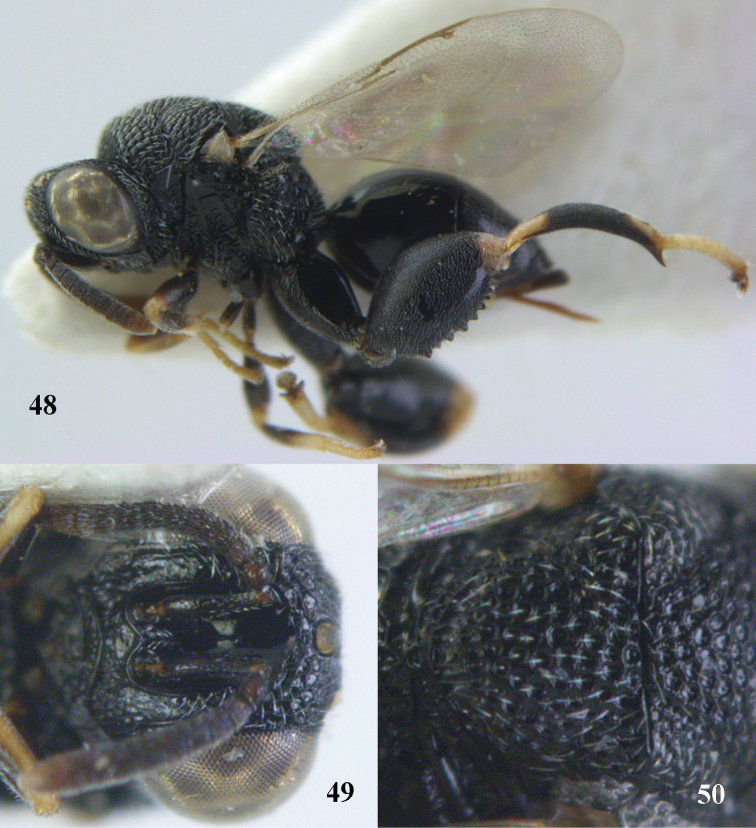
*Brachymeria longiscaposa* Joseph, Narendran & Joy, ♀, Vietnam, Cát Tiên N. P. **48** habitus lateral **49** head anterior **50** mesonotum dorsal.

**Figure 51.**
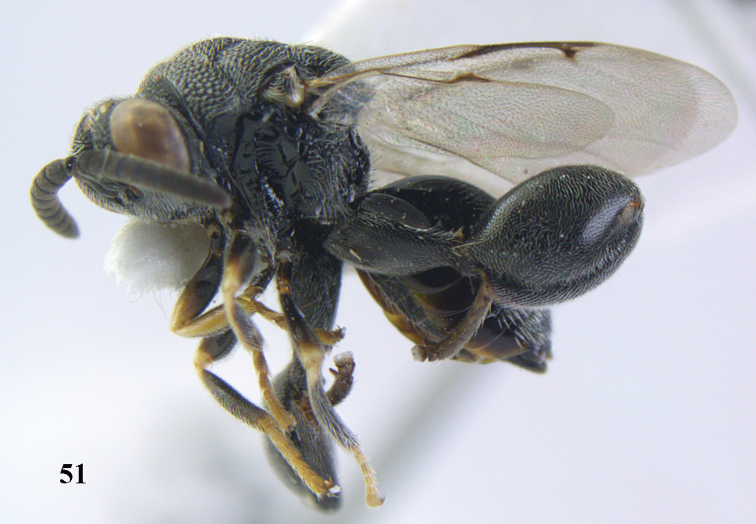
*Brachymeria lugubris* (Walker), ♀, Vietnam, Cát Tiên N. P., habitus lateral.

**Figures 52–53.**
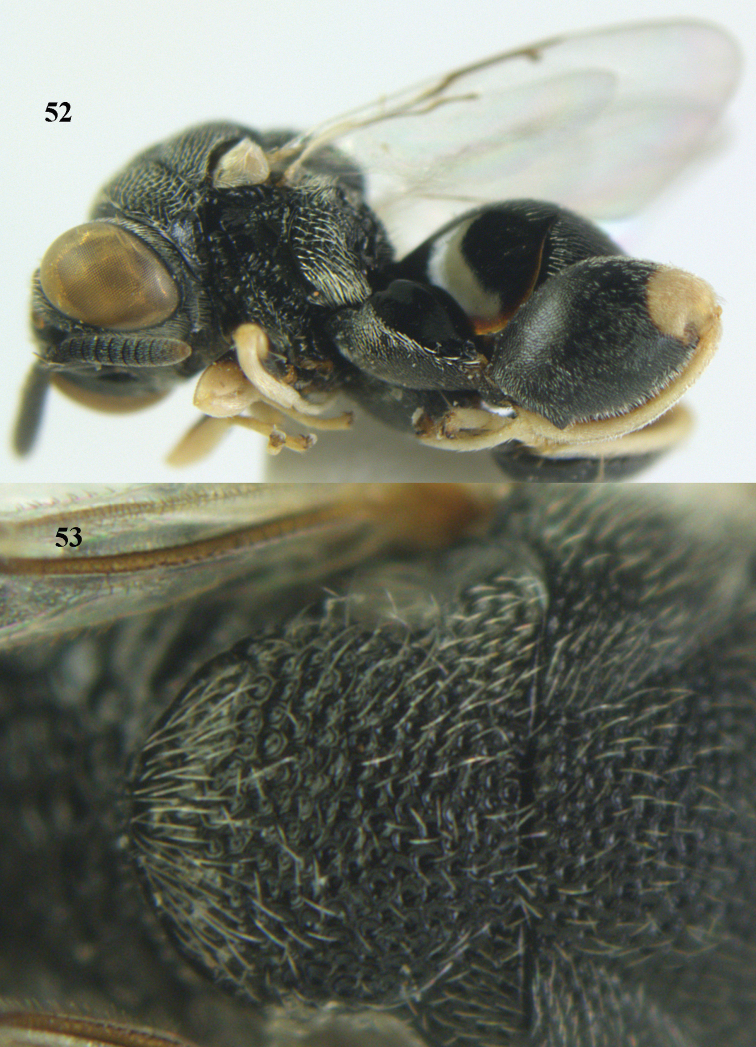
*Brachymeria marmonti* (Girault), ♀, Vietnam, Cát Tiên N. P. **52** habitus lateral **53** scutellum dorsal.

**Figures 54–55.**
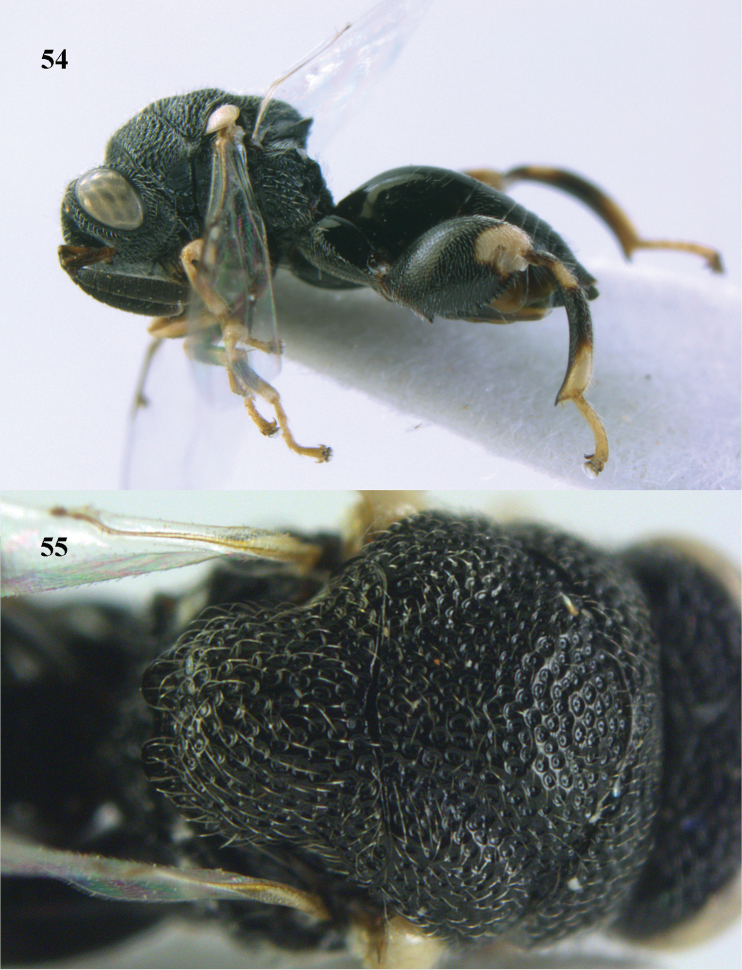
*Brachymeria minuta* (Linnaeus), ♀, Vietnam, Núi Chúa N. P. **54** habitus lateral **55** mesonotum dorsal.

**Figure 56.**
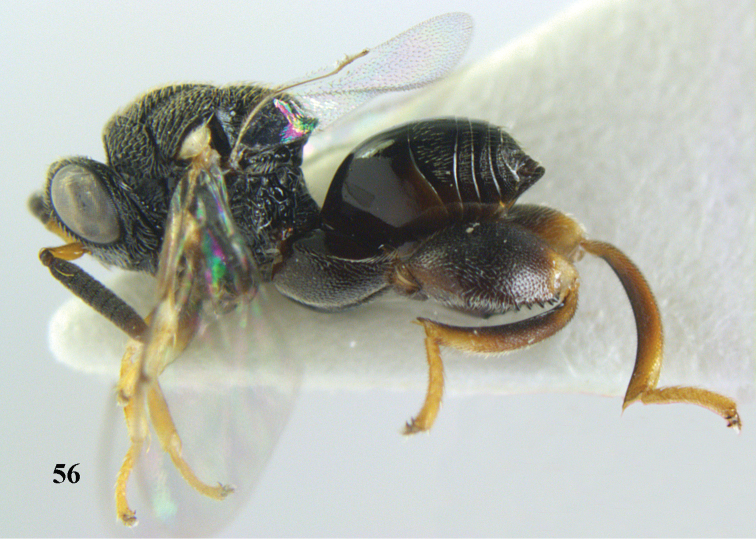
*Brachymeria neowiebesina* sp. n., ♀, holotype, habitus lateral.

**Figures 57–58.**
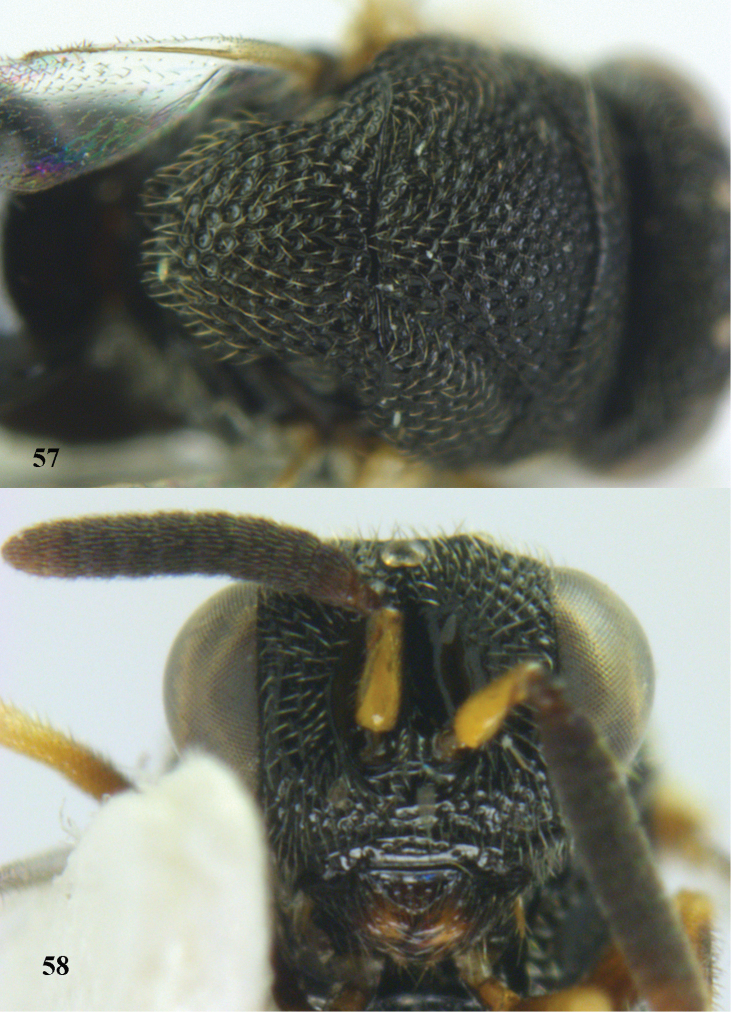
*Brachymeria neowiebesina* sp. n., ♀, holotype. **57** mesonotum dorsal **58** head anterior.

**Figure 59.**
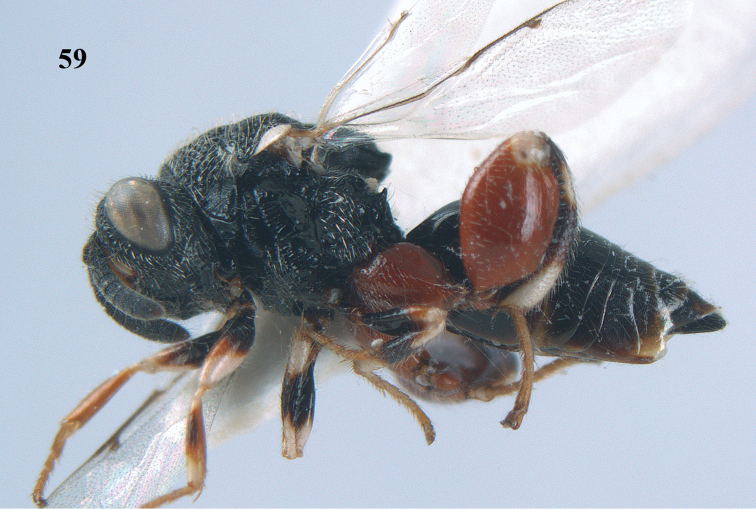
*Brachymeria podagrica* (Fabricius), ♀, Vietnam, Núi Chúa N. P., habitus lateral.

**Figures 60–61.**
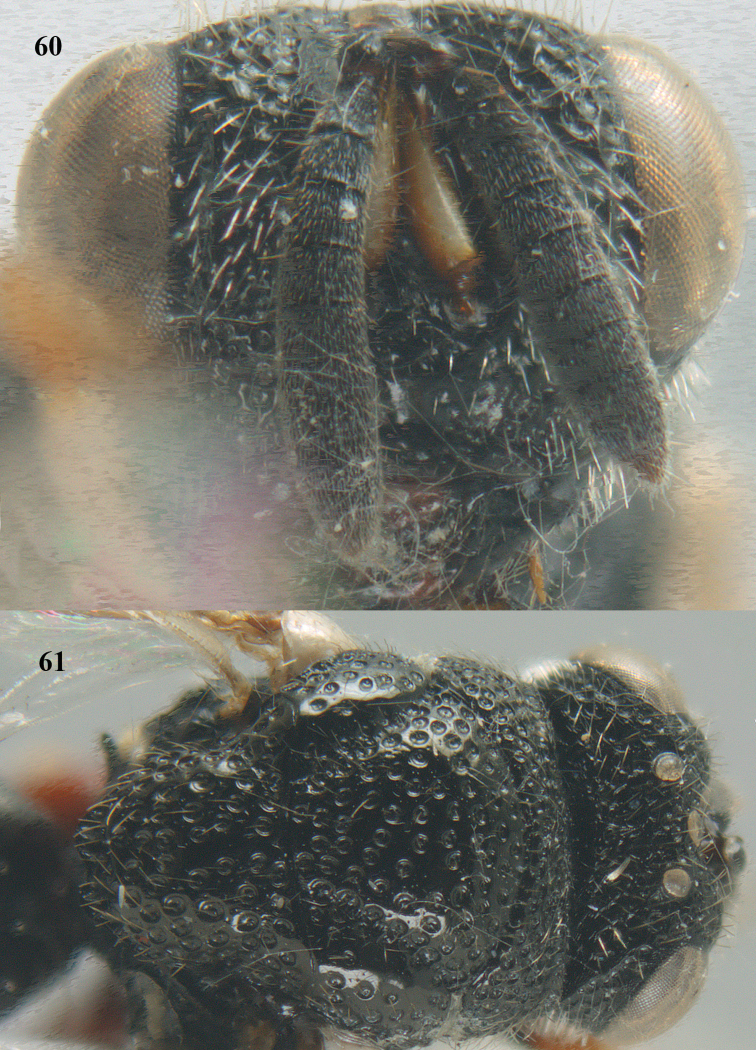
*Brachymeria podagrica* (Fabricius), ♀, Vietnam, Núi Chúa N. P. **60** head anterior **61** mesosoma dorsal.

**Figure 62.**
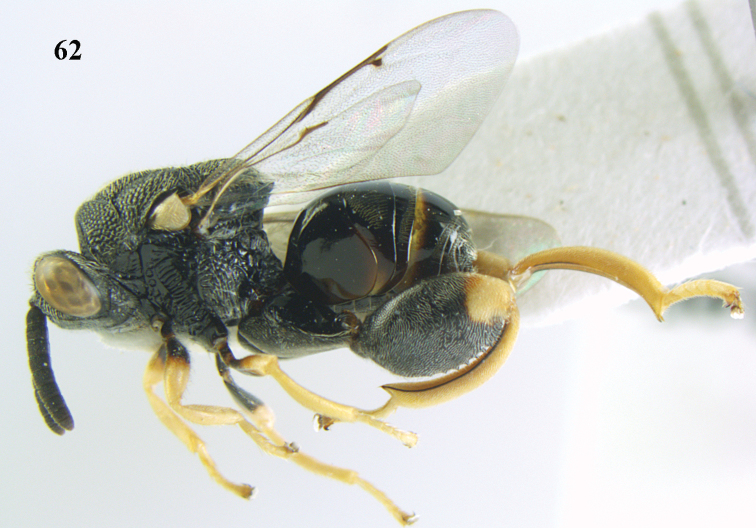
*Brachymeria scutellocarinata* Joseph, Narendran & Joy, ♀, Vietnam, Cát Tiên N. P., habitus lateral.

**Figures 63–64.**
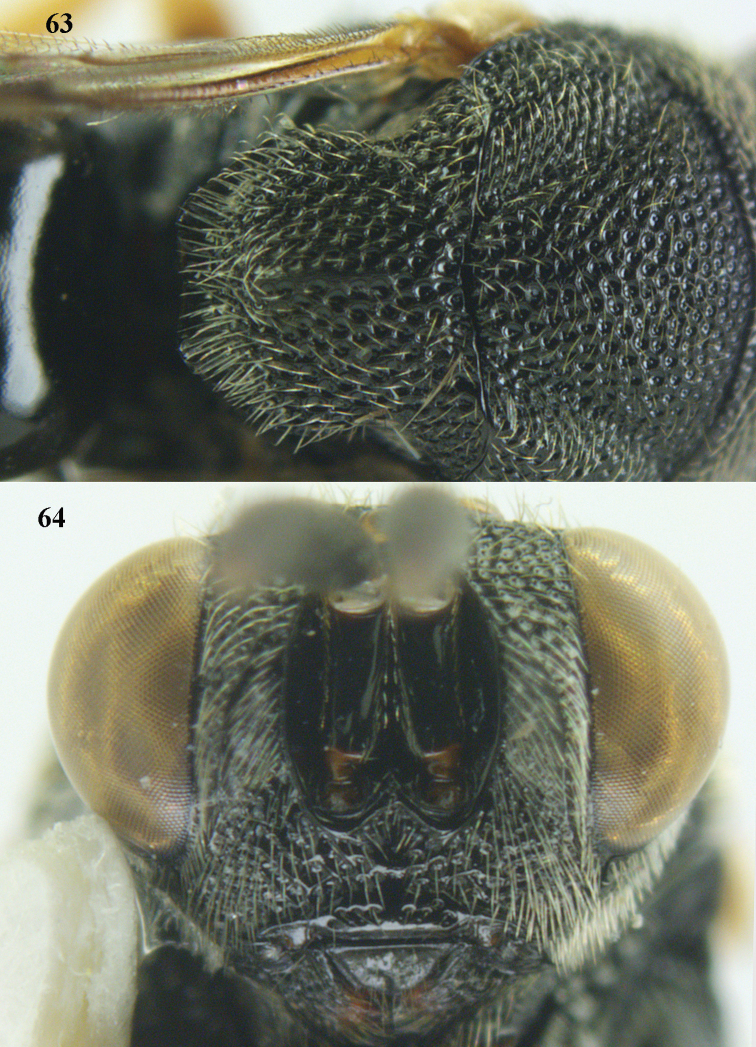
*Brachymeria scutellocarinata* Joseph, Narendran & Joy, ♀, Vietnam, Cát Tiên N. P. **63** mesonotum dorsal **64** head anterior.

**Figure 65.**
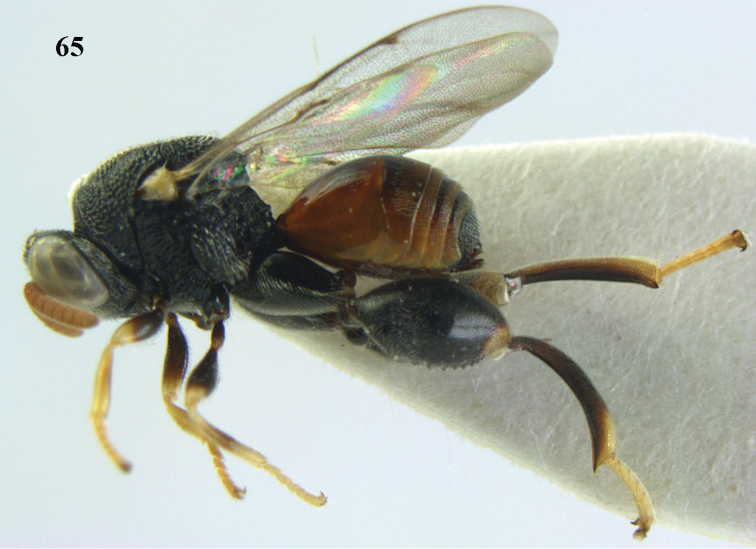
*Brachymeria semirusula* sp. n., ♀, holotype, habitus lateral.

**Figures 66–67.**
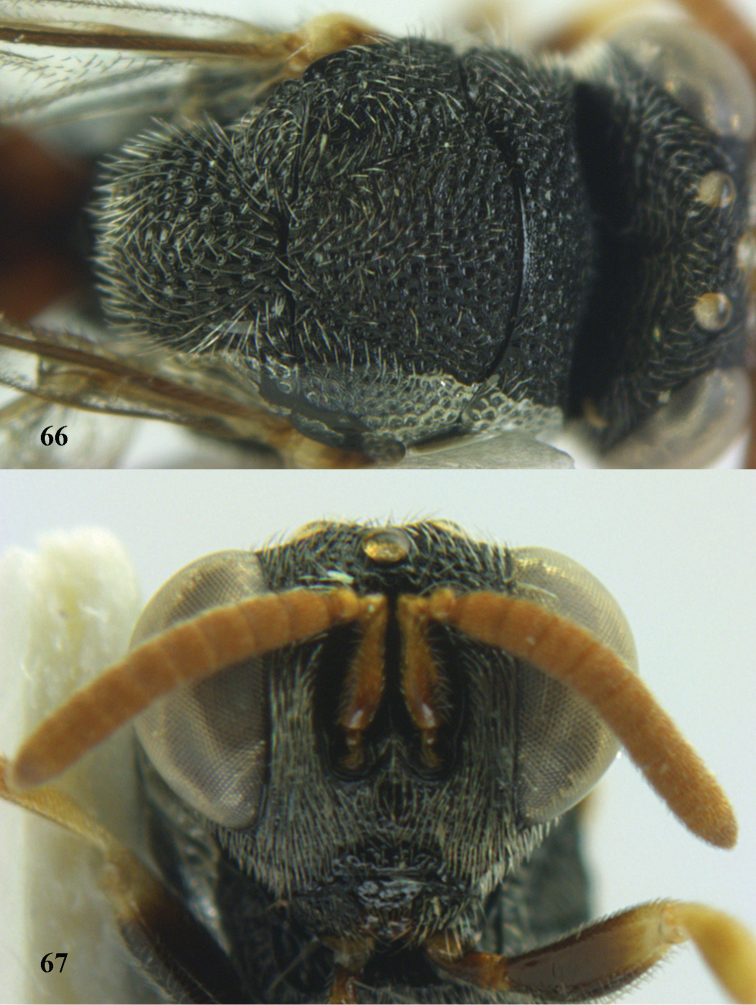
*Brachymeria semirusula* sp. n., ♀, holotype. **66** mesonotum dorsal **67** head anterior.

**Figures 68–70.**
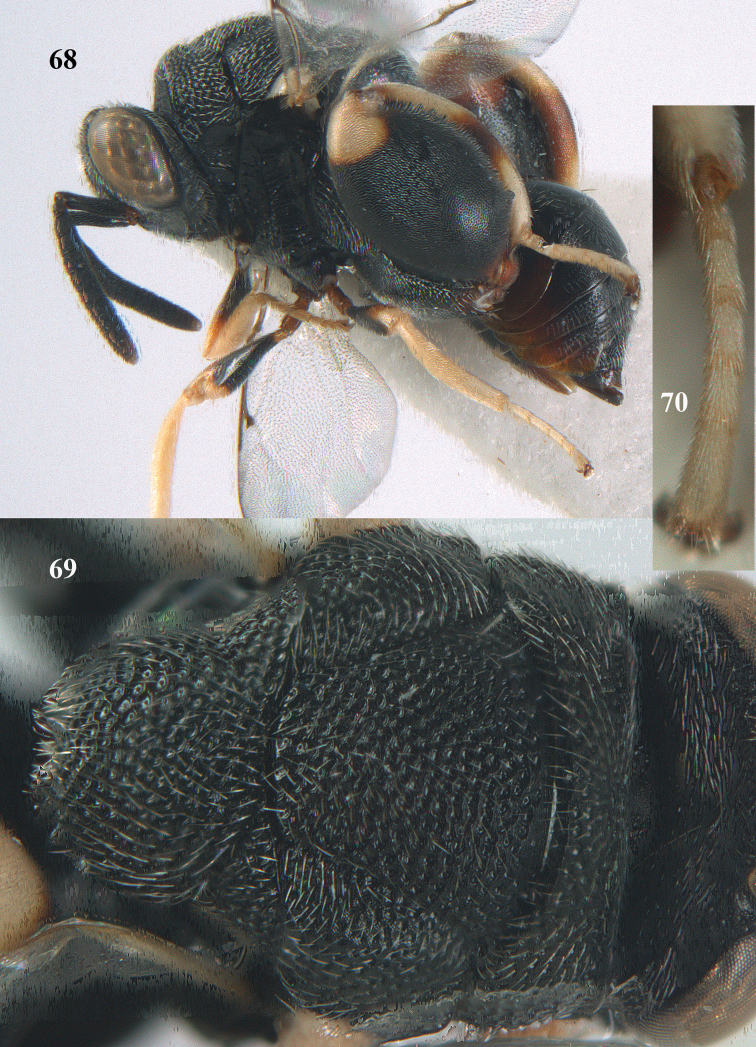
*Brachymeria shansiensis* Habu, ♀, Núi Chúa N. P. **68** habitus lateral **69** scutellum dorsal **70** hind tarsus dorsal.

#### Brachymeria alternipes

Taxon classificationAnimaliaHymenopteraChalcididae

(Walker, 1871)

Figs 25 , 26

Chalcis alternipes Walker, 1871: 49 (♂, Hong Kong (BMNH) (examined)).
Brachymeria alternipes ; Joseph, Narendran & Joy, 1973: 173; Narendran 1989: 245, 272.

##### Material.

1 ♂ (RMNH), “NW **Vietnam**: Tonkin, Hoang Lien N. R., SW Sa Pa, c 1550 m, 22–29.x.1999, Malaise traps, C. v. Achterberg, RMNH’99”.

##### Diagnosis.

This species resembles *Brachymeria atridens* (Walker) in general appearance, but differs from it in having: 1) scrobe distinctly reaching anterior ocellus (in *Brachymeria atridens* scrobe not reaching anterior ocellus); 2) very weak or indistinct pre-orbital carina, (in *Brachymeria atridens* pre-orbital carina strongly developed) and 3) hind femur without any patch (in *Brachymeria atridens* hind femur often with a black patch of varying size on disc).

##### Description.

♂, length of body 4.8–5.9 mm.

*Colour*. Black with cinereous pubescence; antenna black, radicula brown; tegula pale brownish yellow; fore and mid femora black with bases and apices pale brownish yellow; all trochanters black; hind femur red without a distinct apical yellow spot; hind tibia black with base and apex red.

*Head*. Head densely pubescent; head as broad as mesosoma; POL 1.7 × OOL in Vietnamese specimen; face and vertex with deep close umbilicate pits; scrobe smooth and shiny except area near anterior ocellus where it is rugulose, reaching anterior ocellus; antennal toruli situated well above level of lower eye margin; median area of face just below centre of interantennal projection slightly raised and smooth; labrum with approximately ten small round pits in Vietnamese specimen; malar space 0.4 × height of eye in profile; pre-orbital carina indistinct; post-orbital carina reaching geno-temporal margin. Antenna with scape densely pubescent dorsally, relative length: width of antennal segments of Vietnamese specimen: scape = 13:5; F1 = 8:7; F2 = 7:8; F3 = 6:8; F4 = 6:8; F5 = 5:7; F6 = 5:7; F6 = 5:7; clava = 14:7; antenna a little shorter than mesosoma.

*Mesosoma*. Pronotum with anterior margin carinate on sides; pronotum, mesoscutum and scutellum with close umbilicate pits. Interstices narrower than half diameter of a pit and mostly rugulose on anterior half of mesoscutum and on pronotum. Interstices smooth and carinate on scutellum; apex of scutellum emarginated and bi-lobed with dense pubescence all around posterior margin of scutellum. Upper dorsal margin of lateral panel of pronotum arched.

*Wings*. Fore wing 2.7 × longer than wide in Vietnamese specimen; MV a little more than 0.6 × length of SMV, a little more than 3 × as long as PMV; STV half-length of PMV; wing disc fully pilose; marginal fringe shorter than one-fourth length of STV.

*Legs*. Hind coxa without an inner ventro-mesal tooth, dorsal side smooth and shiny, ventral side punctate and pubescent; hind femur twice as long as its width with 9–10 irregular teeth on outer ventral margin, outer disc with close pits, interstices of pits smooth and shiny.

*Metasoma*. Oval, a little shorter than mesosoma; T1 smooth and shiny, its posterior margin straight; T2 with sparse minute pits on anterior half; pits denser laterally, micro-sculptured on posterior half, posterior margin straight; T3 shallowly and sparsely pitted, interstices reticulate; T4 to T6 with close setigerous pits, interstices mostly carinate.

*Female*. Similar to male.

##### Host.

*Anteraea proylei* Jolly (Lepidoptera: Saturniidae (Chalpathy et al. 1998).

##### Distribution.

Vietnam (new record), India, South China.

##### Variation.

The Vietnamese specimen differs from the type as follows: hind femur with relatively small yellow spot at the junction of base of hind tibia and apical ventro-lateral part of hind femur; the male antenna length subequal to mesosomal length and metasoma a little longer than mesosoma (27:24).

#### Brachymeria aurea

Taxon classificationAnimaliaHymenopteraChalcididae

(Girault, 1915)

Figs 27–28

Chalcis area Girault, 1915a: 321 (♀ (?), Australia, Queensland (QMB)).
Chalcis delli Girault, 1924b: 176 (♀ (?), Australia, Queensland (OMB); synonymised with Brachymeria aurea (Girault) by Bouček 1988b).
Brachymeria auratopubescence Joseph, Narendran & Joy, 1972: 345 (holotype ♀, BPBM) India; synonymised with Brachymeria aurea (Girault) by Narendran 1989).

##### Material

(RMNH, IEBR). 2 ♀, “S. **Vietnam**: Dóng Nai, Cát Tiên N. P., ca 100 m, 14–20.v.2007, Mal. traps 20–23, *Lagerstroemia* tr[ail], C. v. Achterberg & R. de Vries, RMNH’07”; 2 ♀, id., but 13–20.v.2007.

##### Diagnosis.

This species comes near *Brachymeria megaspila* Cameron in the key to species by Narendran (1989), but differs from that species in having: 1) golden yellow pubescence on body (in *Brachymeria megaspila* pubescence silvery white); 2) post-orbital carina weakly represented (in *Brachymeria megaspila* post-orbital carina strongly represented) and 3) metasoma as long as mesosoma (in *Brachymeria megaspila* metasoma shorter than mesosoma).

##### Description

(based on female from Cát Tiên N. P.). ♀, length 6.1 mm.

*Colour*. Black with following parts as follows: eye dull yellow; ocelli pale reflecting yellow; apex and base of scape pale brownish yellow; radicula pale brownish yellow; tegula yellow with basal margin dark reddish brown; all coxae concolorous with mesosoma; all femora black with apical half or apex yellow; fore and mid tibiae yellow with a black patch on outer middle part; hind tibia yellow with a dark brown strip each on inner and outer side at base (leaving a yellow strip at middle); all tarsi yellow; claws yellow except dark apex; arolium dark brown with pale yellow pads at apex. Pubescence on vertex, mesoscutum, scutellum and on metasoma golden yellow; pubescence on face, metapleuron and femora pale white.

*Head*. Width of head in anterior view 1.3 × its height (23:18); in dorsal view 2.3 × its length, subequal to width of mesosoma; POL twice OOL; interocular space about 3 × POL. Vertex with close setigerous pits, interstices narrower than diameter of a pit, micro-sculptured; setigerous pits on face closer and interstices carinate; scrobe reaching anterior ocellus, margins carinate; height of malar space 0.3 × height of eye in profile; eye height 1.7 × its length in profile; pre-orbital carina absent; post-orbital carina present reaching geno-temporal margin though broken at middle by a pit; area below scrobe slightly raised at middle, without an impunctate area (though interstices smooth and shiny in the raised part); lateral margins of scrobe faintly produced anteriorly beyond antennal toruli; anterior genal angle slightly acute; hind genal angle subrectangular. Antenna stout; radicula about 0.3 × length of scape; scape almost reaching anterior ocellus; ratios L:W of antennal segments: scape = 10:3; pedicel = 2:2; F1 = 3:3; F2 = 4:4; F3 = 3:4; F4 = 3:4; F5 = 3:4; F6 = 3:4; F7 = 3:4; clava = 4:4.

*Mesosoma*. Pronotum 3.2 × as broad as its median length (including collar), with close setigerous, umbilicate pits, interstices carinate and micro-sculptured, anterior carina separating collar obsolescent at middle; posterior margin of pronotum concave with a row of golden yellow setae directed posteriorly; middle lobe of mesoscutum punctate as in pronotum, a little broader than long (18:16); scutellum as broad as its length, with a distinct, dense row of golden yellow pubescence around margin; apex bi-lobed; scutellum fairly high in profile gently sloping towards apex but perpendicular tooth weakly developed; facies femoralis sunken; metapleuron with close setigerous pits, interstices carinate.

*Wings*. Fore wing 2.9 × longer than wide; relative length of CC = 27; SMV = 21; parastigma = 4; MV = 14; PMV = 6; STV = 3.

*Legs*. Hind coxa smooth and shiny dorsally, closely pitted and pubescent on ventral side, ventro-mesal tooth absent, 0.6 × as long as hind femur; hind femur 1.8 × as long as broad, outer disc closely pitted and pubescent, outer ventral margin a row of 11 differently sized teeth; first proximal tooth larger than others; hind femur with inner basal tooth absent.

*Metasoma*. Metasoma as long as mesosoma, 1.8 × as long as its height; T1 4 × as long as T2 in dorsal view, smooth and shiny, with a small area of pubescence at apical part latero-dorsally; T2 with dense setigerous pits and pubescence on sides and anterior part, remaining part densely micro-sculptured except on distal admarginal area. T6 with 8–9 cross rows of setigerous pits; ovipositor sheath slightly visible in dorsal view.

*Male*. Unknown.

##### Host.

*Delias argenthona* (F.) (Lepidoptera: Pieridae) (Bouček 1988b).

##### Distribution.

Philippines, Indonesia (Java, Sulawesi), Burma, Vietnam (new record) and Australia (Narendran 1989).

##### Variation.

In Australian specimens the golden yellow pubescence is more brilliant and dense than in the Vietnamese or Indian specimens. Area below scrobe is without distinct smooth raised impunctate region in Vietnamese specimens. The colour of the hind femur is slightly variable. The yellow colour at apex of hind femur extends to base through dorsal side in many specimens but in Vietnamese specimens it does not reach the posterior yellow basal part.

#### Brachymeria bengalensis

Taxon classificationAnimaliaHymenopteraChalcididae

(Cameron, 1897)

Figs 29–30

Chalcis bengalensis Cameron, 1897: 39 (♀, India (BMNH) (examined)).
Brachymeria bengalensis ; Mani 1938: 54; Narendran 1989: 243, 261.
Brachymeria yasumatsui Habu, 1963 (holotype ♀, Japan (KYUN); (synonymised with Brachymeria bengalense (Cameron) by Joseph, Narendran & Joy, 1973)).
Brachymeria scrobatae Joseph, Narendran & Joy, 1970: 286–289 (♀, holotype, India (DZUC); synonymised with Brachymeria bengalense (Cameron) by Joseph, Narendran & Joy, 1973).
Stypura variabilis Mani, 1935: 250 (♀, lectotype (NZSI) (designated and synonymised with Brachymeria bengalensis (Cameron) by Narendran 1986).

##### Material.

1 ♀ + 1 ♂ (RMNH, IEBR), “**Vietnam**: Ninh Thuân, Núi Chúa N. P., northeast part, Malaise traps, 90–150 m., 23–30.v.2007, C. v. Achterberg & R. de Vries, RMNH’07”; 1 ♂ (RMNH), “S. Vietnam: Dóng Nai, Cát Tiên N. P., c 100 m, 13–20.v.2007, Botanical Garden, Mal[aise] traps 14–19, C. v. Achterberg & R. de Vries, RMNH’07”; 3 ♀ (BPBM), “Vietnam, 8.xii.1960, C.M. Yoshimoto”.

##### Diagnosis.

This species comes near *Brachymeria deesensis* (Cameron, 1905) in the key to species by Narendran (1989) but differs in having the hind femur reddish or orange brown (often with dark patches) with the apex yellow whereas in *Brachymeria deesensis* the hind femur is black with the apex yellow. The shape of the apical emargination of the scutellum is also a little different in both species. It is possible that *Brachymeria deesensis* Cameron will become a form of *Brachymeria bengalensis* when it is better known.

##### Description.

♀, length of body 4.5–4.6 mm.

*Colour*. Black with following parts as follows: tegula yellowish brown; distal half of fore femur, fore tibia and fore tarsus, distal margin of mid femur, mid tibia and mid tarsus yellow or pale yellow. Coxae black or blackish brown, occasionally reddish brown or blackish red; hind trochanter reddish brown or blackish brown; hind femur red or brownish red with apex yellow and ventral row of teeth black; hind tibia yellow with base black or reddish brown which extends to distal end along ventral margin with a slightly increased width in middle part; tarsi pale yellow or whitish yellow with telotarsi black; metasoma black with lateral part reddish or brownish. Wings hyaline with veins dark brown; pubescence silvery.

*Head*. Width of head in anterior view a little wider than its height; wider than mesosoma in dorsal view; surface distinctly and shallowly pitted, interstices carinate; scrobe reaching anterior ocellus, smooth and shiny; POL 3.3 × OOL; width of ocellar area three-fourths as wide as interocular space; interocellar space twice as wide as major axis of hind ocellus; face with pre-orbital carina absent; post-orbital carina present connecting geno-temporal margin and malar ridge; height of malar space 0.09–0.25 × height of eye in profile. Antenna not longer than mesosoma; scape not exceeding anterior ocellus; as long as F1, F2 and F3 combined; F1 a little wider than long or hardly a little wider than long; F1 to F5 almost equal in length; F6 and F7 equal in length and each slightly shorter than F5; clava a little less than twice length of F7.

*Mesosoma*. Mesosoma compactly pitted dorsally, interstices narrow and somewhat carinate, shagreened throughout; scutellum apex explanate and reflexed, distinctly emarginated and bi-lobed, with dense pubescence; propodeum with an obtuse, rather distinct tooth behind spiracle on either side.

*Wings*. Fore wing 2.5 × longer than wide; relative lengths of fore wing veins: SMV = 37; MV = 21; PMV = 8.

*Legs*. Hind coxa pubescent on ventral side, without an inner ventro-mesal tooth; hind femur 1. 8 × longer than wide, outer side finely punctate, not reticulate, with dense relatively short pubescence, inner side without a protuberance or tooth at base, outer ventral margin with a row of 11 to 12 black differently sized teeth.

*Metasoma*. Metasoma a little shorter than pronotum, mesoscutum and scutellum combined, high in profile, widest a little before middle, abruptly declined posteriorly; T1 smooth, T2 with dense small pits with large setigerous pits at dorso-basal, latero-dorsal and dorso-lateral areas; T6 with close pits, interstices carinate and shagreened. Ovipositor sheath visible in dorsal view.

*Male*. Resembles female in almost all features except width of head equal to mesosoma and antenna slightly stouter.

##### Hosts.

*Pieris brassicae* (Linnaeus) (Lepidoptera: Pieridae) (Devi and Singh 2002), *Erias vitella* Fabricius (Lepidoptera: Noctuidae) (Narendran 1989).

##### Distribution.

India, Malaya, Indonesia, New Guinea, Vietnam, Laos, Thailand, Japan (including Ryukyu Islands), China (Taiwan) and Philippines (Joseph, Narendran & Joy 1973; Narendran 1989).

##### Variation.

The hind femur often has a black patch on outer disc and in some specimens a weak carina is found produced from ventral scrobal margin as in *Brachymeria bengalensis scrobatae* Joseph, Narendran & Joy.

#### Brachymeria carinata

Taxon classificationAnimaliaHymenopteraChalcididae

Joseph, Narendran & Joy, 1970

Figs 31–32

Brachymeria carinata Joseph, Narendran & Joy, 1970: 22 (♀, India (Calicut) (BMNH)).
Brachymeria shansiensis vietnamensis Joseph, Narendran & Joy, 1972: 348 ((♂ “♀”) Vietnam (BPBM) (synonymised by Narendran 1989, with Brachymeria carinata Joseph, Narendran & Joy).
Brachymeria (Neobrachymeria) ghani Joseph, Narendran, & Joy, 1973: 196. (♀, India (Madurai) (USNM) (synonymised by Narendran 1989, with Brachymeria carinata Joseph, Narendran & Joy).

##### Material.

1 ♀ (RMNH), “**Vietnam**: Ninh Thuân, Núi Chúa N. P., dry south part; Mal. traps, 100–180 m, 22–29.v.2007, C. v. Achterberg & R. de Vries, RMNH’07”; 1 ♀ (BPBM), “Vietnam, 8.xi.1960, C.M. Yoshimoto”; 2 ♀, “Vietnam, 12–28.xii.1963, Gayden”.

##### Diagnosis.

This species comes near *Brachymeria margaroniae* Joseph, Narendran & Joy in general appearance, but differs from that species in having: 1) hind tibia with base yellow (in *Brachymeria margaroniae* hind tibia with base reddish black); 2) area below scrobe with median raised smooth portion absent (in *Brachymeria margaroniae* area below scrobe with a raised median smooth portion) and 3) MV about 3 × PMV (in *Brachymeria margaroniae* MV twice PMV).

##### Description

(based on specimen from Núi Chúa N.P.), ♀, length of body 4.3 mm.

*Colour*. Black with following parts as follows: eyes dull grayish yellow with pale reflecting yellow spots; ocelli pale reflecting yellow; tegula pale yellow; all coxae and trochanters concolorous with mesosoma; femora black with apices yellow; all tibiae and tarsi yellow with a black line along ventral margin of hind tibia; wings hyaline with veins brown. Pubescence silvery white.

*Head*. Width of head in anterior view 1.2 × its height; width in dorsal view slightly broader than mesosoma (excluding tegulae) (26:25), 2.4 × its length. POL 4 × OOL; AOL 2.3 × OOL; interocular distance 2.2 × POL; pre-orbital carina present but not reaching malar ridge; post-orbital carina absent; scrobe reaching anterior ocellus; area below scrobe without a raised smooth area, parascrobal space and face longitudinally striate reticulate and densely pubescent; area above parascrobal space and vertex with close umbilicate setigerous pits, interstices rugose and carinate; height of malar space 0.2 × height of eye in profile; eye height 2.4 × its length in profile; anterior genal angle slightly acute, 80° to the vertical axis of malar space; posterior genal angle obtuse to the subvertical geno-temporal margin; gena densely pubescent. Antennal radicula 0.2 × length of scape; scape not reaching anterior ocellus. Relative L:W of antennal segments:scape = 14:3; pedicel = 3:4; anellus = 1:3; F1 = 3:5; F2 = 4:5; F3 = 4:5; F4 = 3:6; F5 = 3:6; F6 = 3:6; F7 = 3:6; clava = 6:6.

*Mesosoma*. Pronotum, mesoscutum and scutellum with close posterior margin of pronotum, slightly concave; lateral panel of pronotum micro-reticulate, without cross striae or carinae and with transverse fovea at lower margin; pronotum with a cross carina adjacent to posterior margin; carina of anterior side limited to sides only; middle lobe of mesoscutum a little shorter than its width (15:17); scutellum a little wider than long (14:12), apex entire; propodeum 50° to the plain of scutellum (not vertical or subvertical); postspiracular tooth indistinct.

*Wings*. Fore wing 2.6 × longer than wide; relative length of veins SMV 13; parastigma = 5; MV = 17; PMV = 6; STV = 3.

*Legs*. Hind coxa smooth and shiny on dorsal half, densely pubescent and punctate on ventral side, inner ventro-mesal tooth absent; hind femur 1.8 × as long as wide, outer ventral margin with a row of 12 differently sized teeth; inner basal tooth absent; mesosternal shelf present.

*Metasoma*. Metasoma slightly longer than mesosoma in dorsal view (30:26); T1 reaching middle, densely micro-sculptured; T2 to T5 densely micro-sculptured and pitted, with pubescence on sides; T6 with 7–8 cross rows of shallow pits, pits and interstices rugose. Ovipositor sheath projecting posteriorly and visible in dorsal view.

*Male*. Unknown.

##### Hosts.

Hyperparasitic in Psychidae (Lepidoptera).

##### Distribution.

Vietnam, India, China, Malaysia (North Borneo).

#### Brachymeria coxodentata

Taxon classificationAnimaliaHymenopteraChalcididae

Joseph, Narendran & Joy, 1970

Figs 33–35

Brachymeria coxodentata Joseph, Narendran & Joy, 1970: 283 (holotype, ♀, India (BMNH) (in original description holotype by mistake as ‘♂’), 1973: 16 (redescribed and keyed); Narendran 1989: 248 (diagnosis and keyed).

##### Material.

1 ♀ (RMNH), “N. **Vietnam**: Hai Phong, Cat Ba N. P., 87 m, N20°47'55", E107°00'19", 18–24.x.2009, Mal. trap, C. v. Achterberg & R. de Vries, RMNH’09”; 1 ♀ (RMNH), “S. Vietnam: Dóng Nai, Cát Tiên N. P., ca 100 m, 13–20.v.2007, Mal. traps 24–29, eco-trail, C. v. Achterberg & R. de Vries, RMNH’07”; 1 ♀ (IEBR), id., but Botanical Garden, Malaise traps 14–19, 13–20.v.2007; 1 ♀ (BPBM), “Vietnam, R.E. Luch, 13.vi.1960”.

##### Diagnosis.

This species comes near *Brachymeria tapunensis* Joseph, Narendran & Joy in the key to species by Joseph et al. (1973), but differs from it in having: 1) punctures on the mesosoma close and interstices carinate (in *Brachymeria tapunensis* interstices of punctures on mesosoma as wide as the diameter of a pit on the median regions of scutellum and scapulae); 2) radicula 0.4 × length of scape (in *Brachymeria tapunensis* radicula is 0.2 × length of scape) and 3) metasoma shorter than combined length of pronotum, mesoscutum and scutellum (in *Brachymeria tapunensis* metasoma longer than combined length of pronotum, mesoscutum and scutellum).

##### Description

(based on specimen from Cat Ba N. P.). ♀, length of body 3.4 mm.

*Colour*. Black with following parts as follows: tegula pale yellowish white. Fore and mid legs: coxa concolorous with body, trochanter black with base and apex pale brown; femur black with apex pale whitish yellow; tibia pale yellow with a dark brown patch on ventro-lateral parts medially; tarsi pale yellow; telotarsi dark brown; hind leg: coxa, trochanter concolorous with mesosoma; femur black with apex pale yellow; tibia black with a pale relatively smaller yellow spot subbasally and one larger yellow spot at apex dorso-laterally; tarsi pale whitish yellow; telotarsi dark brown. Pubescence on body whitish; wings hyaline and veins dark brown.

*Head*. Head in dorsal view slightly wider than mesosoma (excluding tegula), 2.3 × its length; width in anterior view 1.2 × its height; POL 1.8 × OOL; AOL a little shorter than OOL (3:4); width between eyes in dorsal view 3.4 × POL; eye height in profile 1.5 × its length; height of malar space 0.3 × eye height; surface of head closely pitted with interstices carinate and rugose; scrobe smooth and shiny, reaching anterior ocellus, a little longer than wide; parascrobal space 0.4 × width of scrobe; height of eye in anterior view 1.5 × its width; width of clypeus 2.5 × its length; pre-orbital carina absent; post-orbital carina present, reaching geno-temporal margin; lateral ridges of scrobe not extended in front beyond antennal toruli. Antenna with relatively long radicula, 0.4 × as long as length of scape; scape not reaching anterior ocellus. Relative L:W of antennal segments:scape = 7:7; pedicel = 5:6; ring segment = 2:5 F1 = 7:7; F2 = 7:8; F3 = 7:8; F4 = 7:8; F5 = 7:8; F6 = 6:9; F7 = 6:9; clava = 12:10.

*Mesosoma*. Mesosoma with close umbilicate pits, interstices carinate and rugose, without any wider areas in scutellum; scutellum 1.2 × as wide as its length, high in lateral view, perpendicularly declined posteriorly, apical margin rounded

*Wings*. Fore wing 2.7 × longer than wide. Relative length of fore wing veins: SMV = 29; MV = 12; PMV = 7; STV = 3.

*Legs*. Hind coxa with an inner ventro-mesal tooth; hind femur 1.6 × longer than wide, outer ventral margin with 12 differently sized teeth.

*Metasoma*. Metasoma a little shorter than pronotum, mesoscutum and scutellum combined (17:19); 1.2 × longer than wide, about 1.5 × longer than high; T1 smooth and shiny; T6 with 5–6 cross rows of pits, each row with about 18–25 rows of pits.

*Male*. Similar to ♀ except hind coxa without ventro-mesal tooth.

##### Host.

Gregarious hyperparasitoid in hesperiid pupa on *Lilium* sp. (Narendran 1989).

##### Distribution.

India, Philippines, Vietnam (new record), Malaysia, Thailand (Narendran 1989).

#### Brachymeria euploeae

Taxon classificationAnimaliaHymenopteraChalcididae

(Westwood, 1837)

Figs 36–37

Chalcis euploeae Westwood, 1837: 6 (♀, India (HDOU)).
Brachymeria euploeae ; Joseph, Narendran & Joy, 1973: 36; Narendran 1989: 243, 251, 252 (keyed and comments).
Chalcis hearseyi xanthopterus Waterston, 1922: 8 (India (BMNH, FRID) (examined) (synonymised with Brachymeria euploeae (Westwood) by Narendran 1985).
Brachymeria flavotibialis Husain & Agarwal, 1982b: 505 (♀, India, (ZDAMU) (synonymised with Brachymeria euploeae (Westwood) by Narendran 1989).

##### Material

(RMNH, IEBR). 1 ♀, “S. **Vietnam**: Dak Lak, Chu Yang Sin N. P., ca 750 m, 1–10.vi.2007, Mal traps near dam and edge of forest, C. v. Achterberg & R. de Vries, RMNH’07”; 1 ♀, “S. Vietnam: Dóng Nai, Cát Tiên N. P., ca 100 m, 14–20.v.2007, Mal traps 20–23, *Lagerstroemia* tr[ail], C. v. Achterberg & R. de Vries, RMNH’07”; 1 ♀, “Vietnam: Tinh Thuân, Núi Chúa N. P., northwest part, Mal. trap 17, c. 150 m, 24–30.v.2007, C. v. Achterberg & R. de Vries, RMNH’07”.

##### Diagnosis.

This species comes near *Brachymeria jambolana* Gahan in the key to species by Narendran (1989), but differs from it in having the metasoma subglobose and ovipositor sheath not visible in dorsal view (in *Brachymeria jambolana* metasoma acuminate and ovipositor sheath visible in dorsal view). Also the length:width ratio of the antennal segments is different. The female from Núi Chúa N.P. has the apical antennal segment obliquely cut off and may concern a new species.

##### Description

(female from Chu Yang Sin N.P.). ♀, length of body 4.4 mm.

*Colour*. Black with following parts as follows: eye gray with reflecting yellow spots; ocelli pale reflecting yellow; tegula pale yellowish white with basal margin dark brown; apices of all femora, fore and mid tibiae and all tarsi pale yellow; hind tibia pale yellow with base black; telotarsi dark brown; wings hyaline with veins dark brown. Pubescence white.

*Head*. Width of head in anterior view 1.4 × its height; width in dorsal view 2.1 × its length, a little narrower than mesosomal width (16:18); POL twice OOL; AOL subequal to OOL; interocular width 5.3 × POL; vertex and face with close umbilicate setigerous pits, interstices carinate and rugose; area below antennal toruli distinctly punctate without a median smooth area; scrobe reaching anterior ocellus; height of malar space 0.3 × height of eye in profile; height of eye 1.6 × eye length in profile; anterior genal angle acute, 60° to the vertical axis of height of malar space; posterior genal angle obtuse; eye very sparsely and minutely pubescent (careful observation is necessary to observe the minute pubescence of eye); post-orbital carina weakly represented; post-orbital carina reaching geno-temporal margin. Antenna with radicula 0.2 × as long as scape; scape not reaching anterior ocellus. Relative L:W of antennal segments:scape = 22:4; pedicel = 4:6; ring segment = 1:4; F1 = 8:7; F2 = 7:8; F3 = 7:8; F4 = 7:8; F5 = 7:8; F6 = 7:8; F7 = 7:8; clava = 13:8; tip of clava with micropilar area.

*Mesosoma*. Pronotum, mesonotum and scutellum with close umbilicate pits, interstices carinate and rugose; posterior margin of pronotum concave; dorso-anterior corner of lateral panel of pronotum micro-sculptured, remaining part irregularly sculptured; middle lobe of mesoscutum a little longer than its width (15:12); scutellum as long as wide, shorter than middle lobe of mesoscutum (10:15); apex of scutellum entire; propodeum subvertical; postspiracular tooth weakly represented.

*Wings*. Fore wing 2.5 × longer than wide; relative length of SMV = 49; parastigma = 9; MV = 30; PMV = 10; STV = 5.

*Legs*. Hind coxa smooth and shiny dorsally; vertical side with close setigerous pits, without a ventro-mesal tooth; hind femur 1.5 × as long as wide and with a row of 12 differently sized teeth; inner basal tooth absent.

*Metasoma*. Metasoma shorter than mesosoma (24:29), 1.4 × longer than high; T1 smooth and shiny with a set of few setigerous pits on latero-dorsal part of posterior half; T1 reaching middle of metasoma; T2 smooth and shiny with a row of dot-like pits on anterior margin which becomes wide at middle part; sides of T2 with 2–3 cross rows of setigerous pits;T6 with 6 cross rows of pits. Ovipositor sheath not visible in dorsal view.

*Male*. Unknown.

##### Hosts.

Hyperparasitoid in Lepidoptera (Arctiidae, Bombycidae, Drepanidae, Gelechiidae, Geometridae, Hesperiidae, Hyblaeidae, Lasiocampidae; Limacodidae; Lycaenidae, Lymantriidae, Noctuidae, Notodontidae, Nymphalidae, Oecophoridae, Pieridae, Psychidae, Pyralidae, Tortricidae and Zygaenidae) with Hymenoptera: (Braconidae, Ichneumonidae) or Diptera (Tachinidae). (For detailed host list, see Noyes (2011).

##### Distribution.

Oriental region, Australia & U.S.A. New record for Vietnam.

#### Brachymeria excarinata

Taxon classificationAnimaliaHymenopteraChalcididae

Gahan, 1925

Figs 38–39

Brachymeria excarinata Gahan, 1925: 90 (♀, Philippines (USNM) (examined)).
Brachymeria apantelesi Risbec, 1956: 806 (♀♂, Gaorua (MNHN) (synonymised with Brachymeria excarinata Gahan by Narendran 1989).
Brachymeria excarinata plutellae Joseph, Narendran & Joy, 1972: 19 (♂, India (BMNH) (as a subspecies of Brachymeria excarinata Gahan)).

##### Material.

1 ♀ (RMNH), “S. **Vietnam**: Dóng Nai, Cát Tiên N. P., Dong trail, Malaise traps, c. 100 m, 1–8.iv.2007, Mai Phu Quy & Nguyen Tanh Manh, RMNH’07”; 1 ♀ (RMNH), id., but 13–20.v.2007, eco-trail, Malaise traps 25–29; 2 ♀ + 3 ♂ (RMNH, IEBR), “Vietnam: Vinh Phú, Tién Phong, 30.ix.2003, Chi Vu Tri, RMNH’03”; 1 ♂ (RMNH), “Vietnam: Ninh Thuân, Núi Chúa N. P., dry south part; Mal. traps, 100–180 m, 22–29.v.2007, C. v. Achterberg & R. de Vries, RMNH’07”; 1 ♂ (RMNH), “N. Vietnam: Hoa Binh, Pa Co, surr[oundings of] hotel, 12.x.2009, R. de Vries, RMNH’07”.

##### Diagnosis.

This species comes near *Brachymeria manjerica* Narendran in the key to species by Narendran (1989), but differs from *Brachymeria manjerica* in having: 1) pre-orbital carina distinct (in *Brachymeria manjerica* pre-orbital carina absent); 2) head wider than mesosoma (head not wider than mesosoma in *Brachymeria manjerica*), and 3) metasoma completely black (in *Brachymeria manjerica* metasoma reddish brown from T3 to ovipositor sheath).

##### Description.

♀, length of body 1.4–4.5 mm.

*Colour*. Black except following: tegula clear yellow; coxae and trochanters black; femora black with apex yellow; fore tibia yellow with a black patch at middle on outer and ventral sides; mid tibia yellow with blackish band medially (Fig. 38); hind tibia yellow with subbasal and apical yellow spots; fore wing hyaline with veins black or brown.

*Head*. Width of head a little over its height in anterior view, wider than mesosoma in dorsal view; surface weakly or faintly pitted dorsally, rather irregularly carinate on dorsal part of face; faintly carinate on ventral part of face and gena; smooth at middle of face below scrobe; surface in scrobe polished; scrobe reaching anterior ocellus. Pre-orbital carina present; post-orbital carina absent. Distance between outer margin of posterior ocelli (= width of ocellar area) three-fourths distance between eyes (width of interocular space); POL twice OOL; malar ridge 0. 32 × height of eye in profile; posterior genal angle more or less arcuate; right mandible with three pointed teeth. Antenna inserted a little above level of ventral margin of eyes; scape not exceeding anterior ocellus, as long as segments F1 to F4 combined; relative LW of antennal segments:scape = 37:7; pedicel = 9:6; ring segment = 1:5; F1 = 9:8; F2 = 9:9; F3 = 8:10; F4 = 9:11; F5 = 9:11; F6 = 9:11; F7 = 9:11; clava = 17:11.

*Mesosoma*. Mesosoma with close umbilicate pits on dorsum, interstices carinate; apex of scutellum rounded.

*Wings*. Fore wing 2.4–2.5 × longer than wide; relative lengths of veins: SMV = 26; MV = 12; PMV = 5; STV = 2.

*Legs*. Hind coxa without an inner ventro-mesal tooth; hind femur generally a little less than twice as long as wide; outer ventral margin with a row of 10–12 teeth; second hind tarsal segment hardly longer than wide in dorsal view (Fig. 38).

*Metasoma*. Metasoma somewhat pointed posteriorly, subequal in length to mesosoma; T1 smooth, reaching middle of metasoma; T2 finely and densely punctate at dorsal side;T6 very rough owing to rather shallow bristled pits and distinct micro-sculpture. Ovipositor sheath visible from above.

*Male*. Similar to ♀ but antenna stouter and metasoma relatively shorter.

##### Hosts.

Hyperparasitoid in several species of Lepidoptera (Arctiidae, Gelechiidae, Hesperiidae, Noctuidae, Oecophoridae, Pyralidae, Tortricidae, and Yponomeutidae), Coleoptera (Chrysomelidae) with Hymenoptera (Braconidae). For detailed list see Noyes (2011).

##### Distribution.

Widely distributed in Oriental region (including Vietnam), China, Japan, Papua New Guinea and Cameroon (Risbec 1956; Joseph et al. 1973; Narendran and Joseph 1975, Narendran 1989 and Noyes 2011).

##### Variation.

The black colour of tibia becoming faint or absent in many specimens. In some specimens the whole body may be liver brown (*Brachymeria excarinata plutellae*).

#### Brachymeria hime

Taxon classificationAnimaliaHymenopteraChalcididae

Habu, 1960

Figs 40–41

Brachymeria hime Habu, 1960: 199–201 (holotype ♀, Japan (NIAS)), 1962: 58–61 (redescription); Narendran 1989: 247, 264 (comments and keyed).

##### Material

(RMNH, IEBR). 1 ♀, “S. **Vietnam**: Ninh Thuân, Núi Chúa N. P., northeast part, Malaise traps, 90–150 m, 23–30.v.2007, C. v. Achterberg & R. de Vries, RMNH’07”; 1 ♀, “S. Vietnam: Dóng Nai, Cát Tiên N. P., Dong trail. Malaise traps, c. 100 m, 1–8.iv.2007, Mai Phu Quy & Nguyen Tanh Manh, RMNH’07”; 1 ♀, id., but 19–25.iv.2007; 1 ♀, id., but 9.iv.–13.v.2007, M.P. Quy, N.T. Manh & C. v. Achterberg; 1 ♂ + 1 ♀, id., but 13–20.v.2007, Botanical Garden, Malaise traps 14–19, C. v. Achterberg & R. de Vries, RMNH’07.

##### Diagnosis.

This species resembles *Brachymeria secundaria* Ruschka in colour and appearance, but differs from it in having: 1) well developed pre-orbital carina (in *Brachymeria secundaria* pre-orbital carina almost indistinct); 2) scrobe reaching anterior ocellus (in *Brachymeria secundaria* scrobe not reaching anterior ocellus); 3) eyes less convex than of *Brachymeria secundaria* and 4) scutellum low in profile (scutellum high in profile in *Brachymeria secundaria*).

##### Description.

♀, length of body 3.5–5.8 mm.

*Colour*. Black; tegula clear yellow; coxae black; trochanters brown or black; femora black with apices yellow; tibiae yellow with a black band or patch in middle dividing yellow area into anterior and posterior parts, often connected dorsally in fore and mid tibiae; pubescence silvery.

*Head*. Head slightly wider than mesosoma, scrobe reaching anterior ocellus; pre- and post-orbital carinae present; post-orbital carina reaching geno-temporal margin; interantennal projection thin; area below scrobe coarsely punctate; POL distinctly longer than twice OOL; antenna with scape not reaching anterior ocellus.

*Mesosoma*. Mesosoma with close umbilicate pits, interstices subcarinate and reticulate; apex of scutellum rounded; dorsal margin of pronotal panel well arched; hind coxa without an inner ventro-mesal tooth; hind femur without an inner basal tooth, outer ventral margin with a raw of irregular teeth.

*Wings*. Fore wing with PMV one-third as long as MV; SMV twice as long as MV.

*Metasoma*. Metasoma sessile, slightly longer than (or equal to) mesosoma; T1 smooth, its posterior margin straight; ovipositor sheath and epipygium as in figure 37 of Joseph et al. (1973).

*Male*. Similar to ♀ except for stouter antenna and shorter metasoma.

##### Hosts.

*Grapholitha molesta* Busck (Lepidoptera: Tortricidae); *Eutectona machaeralis* (Lepidoptera: Pyralidae), *Nephoteryx eugraphella* Ragonot (Lepidoptera: Phyticidae) (Habu 1962; Sudheendrakumar 1986; Narendran 1989).

##### Distribution.

India, Nepal, China (including Taiwan), Philippines, Vietnam (new record) and Japan (Narendran 1989).

#### Brachymeria jambolana

Taxon classificationAnimaliaHymenopteraChalcididae

Gahan, 1942

Figs 42–43

Brachymeria jambolana Gahan, 1942: 41 (♀, holotype, India (USNM), examined); Narendran 1989: 243, 273 (keyed).

##### Material.

1 ♀ (RMNH), “S. **Vietnam**: Dóng Nai, Cát Tiên N. P., c. 100 m, 9–26.iv.2007, Crocodile tr[ail], Mal. traps, Mai Phu Quy & Nguyen T. Manh, RMNH’07”; 1 ♂ (IEBR), id., but 9.iv.-19.v.2007.

##### Diagnosis.

This species is very close to *Brachymeria euploeae* (Westwood) in colour, but differs from *Brachymeria euploeae* in having the metasoma not globose or subglobose (as in *Brachymeria euploeae*) and the ovipositor sheath is visible in dorsal view (in *Brachymeria euploeae* ovipositor sheath not visible in dorsal view).

##### Description

(female from Cát Tiên N. P.). ♀, length of body 4.3–4.9 mm.

*Colour*. Black; antenna black or dark brown; tegula, apical half of fore femur, apical one-third of mid femur, a large spot at apex of hind femur, fore and mid tibiae entirely, and all tarsi yellow. Hind tibia yellow with a narrow band at extreme base and the ventral carina, black. Pubescence on body grayish white and dense on front of head.

*Head*. Head with pre-orbital carina absent or hardly distinct; post-orbital carina present and reaching geno-temporal margin; antennal clava slightly shorter or at the most as equal to twice length of preceding segment;

*Mesosoma*. Pits on mesosoma close and interstices carinate and rugose; apex of scutellum rounded or slightly entire. Dorsal margin of pronotal panel slightly arched.

*Wings*. Fore wing with PMV 0.33–0.36 × length of MV; SMV slightly shorter than twice MV.

*Legs*. Hind coxa without ventro-mesal tooth; hind femur without an inner basal tooth, outer ventral margin with a row of irregular teeth.

*Metasoma*. Metasoma sessile but very short petiole visible; subacute and ovate; T1 smooth and shiny; its posterior margin straight; following tergites weakly shagreened.

*Male*. Unknown.

##### Hosts.

*Danaus* sp. (Lepidoptera: Danaidae); *Orgya postica* (Walker) (Lepidoptera: Lymantriidae); *Cerea subtilis* Walker (Lepidoptera: Noctuidae); *Papilio agamemnon* (Linnaeus) (Lepidoptera: Papilionidae) (Howlader 1979; Narendran 1986).

##### Distribution.

India, Vietnam (new record); Indonesia (Java and Sumatra) (Noyes 2011).

#### Brachymeria kamijoi

Taxon classificationAnimaliaHymenopteraChalcididae

Habu, 1960

Figs 44–45

Brachymeria kamijoi Habu, 1960: 188 (♂, Japan (EIHU)), 1962: 21–22 (redescription); Narendran, 1989: 256 (note & keyed).

##### Material

(RMNH, IEBR). 2 ♀, “S. **Vietnam**: Dak Lak, Chu Yang Sin N. P., n[ea]r dam, 800–940 m, 2–10.vi.2007, Malaise traps, C. v. Achterberg & R. de Vries, RMNH’07”; 1 ♂, id., but near river, c. 740 m, 1–10.vi.2007; 1 ♀, id., but Krong K’Mar, 740–900 m, 2–10.vi.2007; 1 ♀ + 1 ♂, “S. Vietnam: Dóng Nai, Cát Tiên N. P., ca 100 m, 13–20.v.2007, Mal. traps 25-29, eco-trail, C. v. Achterberg & R. de Vries, RMNH’07”.

##### Diagnosis.

Although Narendran (1989) recorded a female of this species from Philippines, no detailed description was provided. Hence the female of this species is described here in detail. *Brachymeria kamijoi* comes near *Brachymeria fiskei* Habu in the key to species by Habu (1960), but differs from that species in having: 1) the hind tibia black at base except a small yellow spot before base (in *Brachymeria fiskei* hind tibia widely yellowish at base) and 2) the scutellum uniformly and densely pitted (in *Brachymeria fiskei* scutellum with a narrow impunctate smooth space medially). This species also resembles *Brachymeria nephantidis* Gahan in the general colour of the body, but differs from it in having: 1) the scutellum emarginate at its apex (in *Brachymeria nephantidis* scutellum apex evenly curved and entire); and 2) metasoma subglobose (in *Brachymeria nephantidis* metasoma ovate).

##### Description

(female from Chu Yang Sin N.P.). ♀, length of body 5.0–5.3 mm.

*Colour*. Black; eye and ocelli grayish yellow; tegula whitish yellow; all coxae black; all femora black with apices yellow; fore and mid tibiae yellow with black band at middle; hind tibia black with subbasal and apical yellow patches or spots; all tarsi whitish yellow; telotarsi dark brown. Pubescence silvery.

*Head*. Head densely pubescent on face and gena; scrobe separated from anterior ocellus by rugose-reticulate area; POL 2.5 × OOL; each posterior ocellus separated from anterior ocellus by a distance shorter than OOL; pre and post-orbital carina present; MS 0.25 × eye height in profile; eye height 1.7 × eye length in profile; antenna with scape not reaching anterior ocellus. Relative L:W of antennal segments:scape = 13:4; pedicel = 2:4; F1 = 4:5; F2 = 3:5; F3 = 3:4; F4 = 3. 5:7; F5 = 4:6; F6 = 4:6; F7 = 4:6; clava = 8:6.

*Mesosoma*. Mesosoma completely pitted dorsally, interstices narrow and carinate; mesoscutum and scutellum convex; apex of scutellum distinctly emarginated with a row of dense pubescence on all around margins of scutellum.

*Wings*. Fore wing with relative length of veins: SMV = 45; MV = 30; PMV = 8; STV = 4.

*Legs*. Hind coxa without an inner basal tooth, outer ventral margin with 13 irregular sized teeth.

*Metasoma*. Metasoma globose or subglobose, shorter than mesosoma (25:31); T1 reaching half-length of metasoma, smooth and shiny; T2 minutely and densely punctate, punctures becoming faint near dorso-basal area; T2 with fairly dense pubescence laterally; T3 to apex of metasoma pubescent; T6 perpendicular, with 3–4 cross rows of irregular pits; epipygium in dorso-posterior view shorter than half length of T6.

*Male*. Similar to ♀ except for stouter antenna.

##### Host.

Unknown.

##### Distribution.

Vietnam (new record), Japan, Philippines (Narendran 1989).

#### Brachymeria lasus

Taxon classificationAnimaliaHymenopteraChalcididae

(Walker, 1841)

Figs 46–47

Chalcis lasus Walker, 1841: 219 (lectotype ♂ (designated by Bouček 1988) India (Calcutta) (BMNH) (examined); Joseph et al. (1973) transferred Chalcis lasus to Brachymeria in the sense of Brachymeria obscurata and Brachymeria euploeae of previous authors).
Chalcis inclinator Walker, 1862: 355 (♂, China (lectotype designated by Bouček 1988b), Hong Kong (BMNH, HDOU); Joseph et al. (1973) synonymised it with Brachymeria lasus (Walker, 1841)).
Chalcis nitator Walker, 1862: 356 (North Australia, ♂ (lectotype designated by Bouček 1988b, and synonymised it with Brachymeria lasus (Walker, 1841)).
Chalcis obscurata Walker, 1874: 399–400 (Japan, (BMNH), Joseph et al. (1973) synonymised it with Brachymeria lasus (Walker, 1841)).
Oncochalcis marginata Cameron, 1904: 162 (♀, India (BMNH) (examined); Mani (1938) transferred it to Brachymeria Westwood).
Chalcis punctiventris Cameron, 1911: 3 (♀, Sarawak, (BMNH); Joseph et al. (1973) synonymised it with Brachymeria lasus (Walker, 1841)).
Chalcis papuana Cameron, 1913: 85 (♀, Indonesia, (ITZA) (lectotype designated by Bouček 1988b, and synonymized it with Brachymeria lasus (Walker, 1841)).
Tumidicoxa regina Girault, 1913a: 103 (♂, holotype, Australia (UMB); synonymised with Brachymeria lasus (Walker, 1841) by Bouček (1988b)).

##### Material

(RMNH, IEBR). 1 ♀, “S. **Vietnam**: Dóng Nai, Cát Tiên N. P.; Mal. traps 14–19, c. 100 m, 13–20.v.2007, C. v. Achterberg & R. de Vries, RMNH’07”; 1 ♀, id., but Bird trail, Malaise traps 30–35, 15–20.v.2007; 2 ♀, id., but Botanical Garden, 13–20.v.2007, Malaise traps 14–19; 2 ♀ + 2 ♂, id., but Dong trail, 1–8.iv.2007; 2 ♂, id., but 9–30.iv.2007; 2 ♀ + 5 ♂, id, but 9.iv.-19.v.2007; 1 ♂, id., but 1–9.x.2005, *Ficus* trail, Malaise traps 1–8; 1 ♀ + 3 ♂, id., but 19–25.iv.2007, Dong trail, Mai Phu Quy & Nguyen Tanh Manh; 1 ♀, id., but near Head Quarters, 3–8.x.2005; 1 ♀, “S. Vietnam: Dak Lak, Chu Yang Sin N. P., n[ea]r dam, c. 500 m, Mal[aise] traps, 3–9.vi.2007, C. v. Achterberg & R. de Vries, RMNH’07”.

##### Diagnosis.

This species comes very close to *Brachymeria albotibialis* (Ashmead) in general colour and in having hind coxa with an inner ventro-mesal tooth. However, it differs from *Brachymeria albotibialis* in having: 1) T1 smooth and shiny (in *Brachymeria albotibialis* T1 shagreened); 2) metasoma ovate and about as long as mesosoma (in *Brachymeria albotibialis* metasoma distinctly shorter than mesosoma, not ovate, T6 subvertical) and 3) area below scrobe with a smooth part (in *Brachymeria albotibialis* area below scrobe without a smooth part).

##### Description

(based on Vietnamese specimens). ♀, length of body 6.8 mm.

*Colour*. Black with following parts as follows: eyes grayish yellow with reflecting spots; ocelli pale reflecting yellow; all coxae and trochanters concolorous with mesosoma; tegula yellow; all femora black with apices yellow; fore and mid tibiae yellow with a black patch on inner middle part; hind tibia yellow with base and inner ventral marginal area black; all tarsi yellow; telotarsi black; fore wing hyaline with veins dark brown.

*Head*. Width of head in anterior view 1.3 × its height (24:18); head width in dorsal view 2.7 × as long as its length, as wide as mesosoma (excluding tegulae); POL 2.4 × OOL; AOL a little shorter than OOL (4:5); width between eyes in dorsal view 2.8 × POL; vertex, occiput and face with umbilicate setigerous close pits, interstices carinate and rugose; pre-orbital carina absent; post-orbital carina present, reaching geno-temporal margin; area below scrobe with a smooth portion at middle; scrobe reaching anterior ocellus, surface smooth; height of malar space 0.25 × height of eye; eye height 1.8 × its length in profile; anterior genal angle acute, hind genal angle rectangular but widely rounded. Antenna with scape reaching anterior ocellus but not exceeding it; relative L:W of antennal segments: scape = 35:7; pedicel = 5:7; ring segment = 6:1; F1 = 9:8; F2 = 9:9; F3 = 9:9; F4 = 9:9; F5 = 9:10; F6 = 8:10; F7 = 8:10; clava = 15:10.

*Mesosoma*. Median length of pronotum 0.3 × width of pronotum, with close umbilicate pits, interstices narrower than diameter of a pit, rugose and micro-striate, not carinate; mesoscutum length a little over twice median length of pronotum (17:8), with close pits as in pronotum; interstices a little wider on median part of scapula; scutellum a little shorter than mesoscutum (16:17), a little wider than long (18:16), with close umbilicate, setigerous pits, interstices shorter than diameter of a pit, smooth and shiny; scutellum high in profile, abruptly declined posteriorly, apex rather widely explanate, weakly bi-lobed.

*Wings*. Fore wing 2.6 × longer than wide; relative length of fore wing veins: SMV = 28; MV = 15; PMV = 7; STV = 3.

*Legs*. Hind coxa strongly and densely punctate on ventral side with a tooth on inner ventral side; hind femur 1.9 × as long as its width, minutely densely punctate and densely pubescent on outer and inner sides, inner basal tooth absent, outer ventral margin with a row of 9 differently sized teeth.

*Metasoma*. Metasoma subequal in length to mesosoma or slightly longer than mesosoma; T1 slightly reaching beyond middle, smooth and shiny; T2 with setigerous close pits on sides, dorsally with one or 2 rows of minute pits near anterior margin, remaining parts of T2 with extremely minute pit like sculpture; T6 with 8 to 9 cross rows of closely set setigerous pits, interstices with dense micro-sculpture. Ovipositor sheath visible in dorsal view, 0.6–0.7 × as long as epipygium in dorsal view; epipygium 0.4 × as long as T6 in dorsal view.

*Male*. Similar to female but hind coxa without inner ventro-mesal tooth and funicle with trichoid sensillae on ventral side.

##### Hosts.

Polyphagous species, parasitising pupae of a wide range of Lepidoptera. Occasionally hyperparasitic on Lepidoptera with Hymenoptera or Diptera (for detailed host lists see Noyes 2011).

##### Distribution.

Australia, China (including Taiwan), Fiji, Guam, India, Indonesia (Irian, Java), Japan, Korea, Malaysia (Sarawak, Palau), Philippines, Papua New Guinea, U.S.A. and Vietnam.

##### Variation.

The colour of hind tibia varies greatly in specimens of different regions. In many south eastern forms the black patch of hind tibia is much more pronounced than those of South Indian forms. In Japanese specimens the black colour of hind tibia is more pronounced than any other regional specimens seen by the first author.

#### Brachymeria longiscaposa

Taxon classificationAnimaliaHymenopteraChalcididae

Joseph, Narendran & Joy, 1972

Figs 48–50

Brachymeria longiscaposa Joseph, Narendran & Joy, 1972: 343–345 (♀, Taiwan (BPBM)), 1973: 343 (keyed and repeated description); Narendran 1989: 246, 265 (keyed and commented).

##### Material.

1 ♀ (RMNH), “S. **Vietnam**: Dóng Nai, Cát Tiên N. P., c. 100 m. 19–25.iv.2007, Mal. traps. Dong trail, Mai Phu Quy & Nguyen Tanh Manh, RMNH’07”; 1 ♀ (BPBM), “Vietnam, 8–19.vii.1960, C.M. Yoshimoto”; 1 ♀ (BPBM), “Vietnam, Fyan (1200 m) 11.vii.-9.viii.1961”.

##### Diagnosis.

This species comes near *Brachymeria secundaria* (Ruschka) in the key to species by Narendran (1989), but differs from *Brachymeria secundaria* in having: 1) scape longer than combined length of F1, F2, F3 and F4 (in *Brachymeria secundaria* scape shorter than F1, F2, F3 and F4); 2) pre-orbital carina well developed and joining malar ridge (in *Brachymeria secundaria* pre-orbital carina indistinct) and 3) scutellum not high in lateral view, sloping down gently posteriorly (in *Brachymeria secundaria* scutellum high in lateral view, subvertical posteriorly).

##### Description

(female from Cát Tiên N. P.). ♀, length of body 2.4 mm.

*Colour*. Black with following parts as follows: eye gray with pale yellow reflecting spots; ocelli pale reflecting yellow; tegula pale whitish yellow; antenna black with apex of clava pinkish brown; all coxae concolorous with mesosoma; trochanters dark brown; fore and mid femora black with base and apex pale yellow; hind femur black with apex pale yellow; all tibiae black with base and apex yellow (the yellow colour of base and apex weakly connected dorsally in fore tibia); tarsi yellow; telotarsi brown; arolium white at apex; fore wing hyaline with veins dark brown. Pubescence on body white.

*Head*. Width of head in anterior view 1.2 × its height, width in dorsal view twice its length, equal to width of mesosoma (excluding tegulae); vertex shallowly pitted on POL and adjacent area behind, pits deeper in other parts of vertex; interstices and inside of pits reticulate, and weakly carinate. POL 3 × OOL; AOL 0.5 × POL; interocular distance 4.3 × POL. Scrobe not reaching anterior ocellus parascrobal space shallowly punctate and rugose; area below scrobe with a relatively small smooth area in middle; lateral margins of scrobe slightly produced anteriorly; pre-orbital carina distinct, reaching malar ridge; post-orbital carina reaching geno-temporal margin; height of malar space 0.2 × eye profile; anterior genal angle 70° to the vertical axis; posterior genal angle subvertical and obtuse; eyes very minutely and sparsely pubescent. Antenna with scape not reaching anterior ocellus; radicula 0.2 × length of scape; relative L:W of antennal segments:scape = 16:4; pedicel = 4:4; ring segment = 1:3; F1 = 3:5; F2 = 4:5; F3 = 4:5; F4 = 4:5; F5 = 4:5; F6 = 4:5; F7 = 4:5; clava = 10:5.

*Mesosoma*. Pronotum, mesoscutum and scutellum with close setigerous pits, interstices narrower than half diameter of a pit, weakly carinate, rugose; pronotum 3.1 × as broad as long, as long as mesoscutum (excluding tegulae); posterior margin of pronotum concave; middle lobe of mesoscutum 1.6 × as broad as its length; scutellum as long as mesoscutum, as long as broad and apex rounded; propodeum 70° declining to the vertical axis of scutellum; postspiracular teeth indistinct.

*Wings*. Fore wing 2.7 × longer than wide; relative length of CC = 21; SMV = 18; parastigma = 3; MV = 14; PMV = 4; STV = 2.

*Legs*. Hind coxa smooth and shiny dorsally, closely pitted and pubescent on ventral side, ventro-mesal tooth absent; hind femur 1.7 × as long as broad, inner basal tooth absent; outer ventral margin with a row of 10 differently shaped teeth.

*Metasoma*. Metasoma as long as mesosoma in lateral view, 1.7 × as long as its height; T1 smooth and shining, almost reaching middle of metasoma, its posterior margin straight; T2 faintly shagreened, 0.3 × as long as T1 in dorsal view; T3, T4 and T5 weakly shagreened; T6 reticulate without distinct pits; ovipositor sheath visible in dorsal view.

*Male*. See Joseph, Narendran and Joy (1973).

##### Host.

Unknown.

##### Distribution.

Vietnam, China (Taiwan).

#### Brachymeria lugubris

Taxon classificationAnimaliaHymenopteraChalcididae

(Walker, 1871)

Fig. 51

Chalcis lugubris Walker, 1871: 49 (♀, China (Hong Kong), (BMNH) (lectotype designated by Bouček 1988b).
Brachymeria lugubris ; Joseph, Narendran & Joy, 1973: 302–304.
Chalcis atrata Kirby, 1883: 76. (♀, Australia, (BMNH) (lectotype designated by Bouček 1988) synonymised with Brachymeria lugubris by Bouček 1988b).

##### Material.

1 ♀ (RMNH), “S. **Vietnam**: Dóng Nai, Cát Tiên N. P., c. 100 m., 15–20.v.2007, Mal. traps 30–35, Bird trail, C. v. Achterberg & R. de Vries, RMNH’07”; 1 ♀ (BPBM), “Vietnam, 28.xi.1960, C.M. Yoshimoto”.

##### Diagnosis.

*Brachymeria lugubris* resembles *Brachymeria fiskei* (Crawford) in general black colour and in having pre- and post-orbital carinae and apex of scutellum emarginate. However, *Brachymeria lugubris* differs from *Brachymeria fiskei* in having 1) scutellum with a median ridge or carina (in *Brachymeria fiskei* scutellum with only a smooth longitudinal area); 2) hind tibia completely black (in *Brachymeria fiskei* hind tibia black with base and apex pale); 3) anterior genal angle obtuse (in *Brachymeria fiskei* anterior genal angle acute) and 4) T1 faintly sculptured (in *Brachymeria fiskei* T1 smooth and shiny).

##### Description.

♀, length of body 5.5–6.4 mm.

*Colour*. Black; tegula black with yellow margins; hind leg black with minute tan spot at apex of hind femur; inner side of fore and mid tibiae blackish brown. Pubescence dense and silvery.

*Head*. Head with scrobe reaching anterior ocellus; height of malar space 0.3 × height of eye in profile; AOL a little over 0.3 × POL; interocular distance at vertex about 2.8 × POL; POL a little over twice OOL; POL a little over 2.3 × diameter of posterior ocellus. Pre-orbital and post-orbital carinae present; post-orbital carina reaching geno-temporal margin; area below scrobe with a very small (smaller than diameter of anterior ocellus) median smooth portion; lateral ridges of scrobe not produced anteriorly; anterior and posterior genal angles rectangular. Antenna with scape not reaching anterior ocellus; F1 to F5 subequal in size; F6 a little shorter than F5 and a little longer than F7; clava slightly shorter than F6 plus F7 combined, 1.8–2.0 × as long as wide.

*Mesosoma*. Mesosoma with rounded, umbilicate and close pits; interstices narrower than half diameter of a pit and rugose on pronotum and mesoscutum; interstices on scutellum narrower than diameter of a pit and smooth; scutellum with a median longitudinal ridge or carina; scutellum with apical margin emarginated with dense pubescence.

*Wings*. Fore wing slightly longer than 2.8 × its width; relative length of CC = 47; SMV = 41; parastigma = 7; MV = 21; PMV = 8; STV = 4.

*Legs*. Hind coxa without an inner ventro-mesal tooth; hind femur 1.7–1.8 × as long as wide, without an inner basal tooth; outer ventral margin with a row of differently sized 12 teeth.

*Metasoma*. Length of metasoma subequal to pronotum, mesoscutum and scutellum combined, a little less than 1.7 × as long as its height; T1 faintly sculptured; T2 with a few punctures at baso-dorsal part, rest of dorsal side with dense micro-sculpture, lateral part punctate and pubescent; T6 with 5 irregular cross rows of shallow pits, interstices and inside of pits rugulose. Ovipositor sheath visible from above.

##### Hosts.

Hyperparasitoid in Lepidoptera (*Bombyx mori* Linn. (Bombycidae), *Hyblaea puera* Cramer (Hyblaeidae), *Mahasena corbetti* Tams (Psychidae), *Antheraea proylei* Jolly (Saturniidae), *Atteva fabriciella* Swederes (Yponomeutidae) and *Artona catoxantha* Hampson (Zygaenidae)) with Diptera (Tachinidae: *Bessa remota* Aldrich, *Eozenillia equatorialis* Townesend, *Exorista bombycis* (Louis), *Exorista sorbilans* (Wiedemann), *Trycholyga bombycis* Beck).

##### Distribution.

Vietnam (new record), Indonesia (Java), China (Hong Kong, Taiwan), Philippines (Joseph, Narendran & Joy 1973); Australia, and Malaysia. (Noyes 2011).

#### Brachymeria margaroniae

Taxon classificationAnimaliaHymenopteraChalcididae

Joseph, Narendran & Joy, 1973

Brachymeria margaroniae Joseph, Narendran & Joy, 1973: 108 (♂, India (BMNH)).
Brachymeria (Brachymeria) josephi Husain & Agarwal, 1982b: 507 (♀, India, Uttar Pradesh (ZDAMU) (synonymised with Brachymeria margaroniae Joseph, Narendran & Joy by Narendran 1989)).

##### Material.

1 ♀ (BPBM), “**Vietnam**, 8-16.xi.1960, C.M. Yoshimoto”.

##### Diagnosis.

This species comes near *Brachymeria jayaraji* Joseph, Narendran & Joy in having similar colour of hind tibia but differs from *Brachymeria jayaraji* in having: 1) area below scrobe with a raised, smooth, median portion (in *Brachymeria jayaraji* area below scrobe with raised median portion absent) 2) apex of scutellum wide and slightly emarginated (in *Brachymeria jayaraji* apex of scutellum narrower and not at all emarginated) and 3) hind femur black with apex yellow (in *Brachymeria jayaraji* hind femur usually red with a blackish patch near yellow apex).

##### Description.

♀♂, length of body 3.7–4.0 mm.

*Colour*. Black; tegulae yellow; coxae, trochanters and femora black except the distal tips of femora which are yellow; tibiae yellow except base of hind tibia reddish black extending to distal region along ventral margin with a slight extension in the middle towards dorsal region, tarsi yellowish.

*Head*. Pre-orbital carina faint, almost indistinct; post-orbital carina absent; anterior genal angle nearly rectangular; hind genal angle slightly obtuse and rounded off; area below scrobe with a raised median smooth portion. Antennal scape not exceeding anterior ocellus.

*Mesosoma*. Mesosoma provided with, reticulate, rounded, umbilicate and close pits; interstices of pits narrow and rugose; apex of scutellum very slightly emarginated.

*Wings*. Fore wing a little more than 2.5 × as long as wide; relative length of SMV = 34; parastigma = 3; MV = 14; PMV = 7; STV = 3.

*Legs*. Hind coxa without an inner ventro-mesal tooth; hind femur about 1.8 × as long as wide, with a row of 11 differently sized teeth on outer ventral margin; basal inner tooth on hind femur absent.

*Metasoma*. Metasoma longer than pronotum, mesoscutum and scutellum combined in ♀, pointed narrow apically. T1 shagreened.

##### Host.

*Diaphania* (= *Margaronia*) *indica* (Lepidoptera: Pyralidae).

##### Distribution.

Vietnam, India and Philippines.

#### Brachymeria marmonti

Taxon classificationAnimaliaHymenopteraChalcididae

(Girault, 1924)

Figs 52–53

Chalcis marmonti Girault, 1924b: 175 (♀, Australia, Queensland (OMB)).
Brachymeria marmonti ; Narendran 1989: 253–254; Dahms 1984: 795; Bouček 1988: 71.
Brachymeria koduvalliensis ; Joseph, Narendran & Joy, 1972: 345 (holotype ♀, BMNH) India; synonymised with Brachymeria marmonti (Girault) by Bouček 1988).
Chalcis wittei Schmitz, 1946: 47 (♀?, (Tervuren)) Congo; synonymised with Brachymeria marmonti (Girault) by Bouček 1988).

##### Material

(RMNH). 1 ♀, “S. **Vietnam**: Dóng Nai, Cát Tiên N. P., Dong trail, Mal. traps, c. 100 m, 1–8.iv.2007, Mai Phu Quy & Nguyen Tanh Manh, RMNH’07”.

##### Diagnosis.

Similar to *Brachymeria megaspila* (Cameron), but *Brachymeria marmonti* has hind femur black except an ivory patch apically (with large black medial patch and remainder yellow in *Brachymeria megaspila*) and posterior lamella of scutellum evenly convex medio-posteriorly (emarginate medio-posteriorly).

##### Description.

(♀) Pre- and post-orbital carinae present; scrobe reaching anterior ocellus; scutellum with interspaces between punctures narrower than half diameter of punctures and often carinate; apex of scutellum rounded.

##### Host.

Hyperparasitoid of Lepidoptera through Braconidae and Ichneumonidae.

##### Distribution.

Afrotropical, Oriental and Australian regions. New record for Vietnam.

#### Brachymeria megaspila

Taxon classificationAnimaliaHymenopteraChalcididae

(Cameron, 1907)

Chalcis megaspila Cameron, 1907: 581 (♀, India, (lectotype designated by Bouček 1988) (BMNH) (examined)).
Brachymeria megaspila ; Mani 1938: 56.
Chalcis koebelei Crawford, 1910: 207 (China (Hong Kong), ♀ (USNM) (synonymised with Brachymeria megaspila by Bouček 1988b)).
Chalcis poema Girault, 1927: 324 (Australia, ♀ (SAMA) (synonymised with Brachymeria megaspila by Bouček 1988b)).

##### Material.

4 ♀ (BPBM), “**Vietnam**, 7.viii.1961, N.R. Spencer”.

##### Diagnosis.

This species resembles very closely the Palaearctic *Brachymeria femorata* (Panzer) from which it can be separated by the relatively small pits on the outer disc of the hind femur (pits relatively larger on outer disc of hind femur in *Brachymeria femorata*) and in having head compressed from anterior to posterior direction (in *Brachymeria femorata* head not compressed). Only with long experience in the taxonomy of *Brachymeria* one can distinguish these two sibling species.

##### Description.

♀, length of body 4.5–5.0 mm.

*Colour*. Black; tegula pale yellow; coxae black, hind trochanter reddish brown; fore femur yellow with basal area blackish brown on outer side; mid femur blackish brown at basal two-thirds and clear yellow at remaining part; hind femur yellow or reddish yellow with a black patch in middle; tibiae clear yellow except that outer ventral carina of hind tibia is black; tarsi pale yellow. Pubescence silvery grey.

*Head*. Head with scrobe reaching anterior ocellus; AOL 0.3 × POL; interocular space at vertex 2.2 × POL; POL 3.8 × OOL. Pre-orbital carina absent; post-orbital carina present, reaching geno-temporal margin; lateral ridges of scrobe produced anteriorly beyond antennal toruli; area below scrobe with a small median smooth and raised portion; height of malar space slightly less than 0.3 × height of eye in profile; anterior genal angle slightly acute, posterior genal angle nearly rectangular. Antennal scape almost reaching anterior ocellus, shorter than F1, F2 and F3 combined; pedicel a little wider than long; F1 to F4 almost equal in length; width slightly increasing from segments F2 to F7; clava 1.5 × as long as wide, a little over twice as long as F7.

*Mesosoma*. Mesosoma with reticulate, rounded, umbilicate and close pits; interstices narrow and smooth except on pronotum where these interstices are rugose and narrow; scutellum length subequal to its width, fairly high in profile, almost perpendicularly declined posteriorly; apical flange emarginated with dense pubescence.

*Wings*. Fore wing 2.8 × as long as wide; MV a little over 0.6 × SMV; PMV a little over 0.4 × PMV and a little over twice STV.

*Legs*. Hind coxa without a ventro-mesal tooth; hind femur a little over 2.6 × as long as wide; its outer ventral margin with a row of 12–13 differently sized teeth; without an inner basal tooth or protuberance.

*Metasoma*. Metasoma a little shorter than head, mesoscutum and scutellum combined; 1.3 × as long as wide; T1 smooth;T2 with large distinct punctures in the basal half with a smooth area in median region, its distal half in the dorsal region and distal one-fourth in the latero-dorsal regions finely micro-sculptured. T6 subperpendicular, with 6 or 7 cross rows of distinct and deep pits, each row with several pits. Ovipositor sheath slightly visible in dorsal view.

##### Hosts.

Lepidoptera: *Opisina arenosella* Walker (Oecophoridae), *Eurema blanda* Boisdual, *Eurema hecabe* (Linnaeus), *Eurema hecale* Zuleika, and *Delias* sp. (Pieridae) (Noyes 2011).

##### Distribution.

Vietnam, Indonesia (Java), India, Australia (Narendran 1989), Sri Lanka, Malaysia, China (Hong Kong) and Papua New Guinea (Noyes 2011).

#### Brachymeria minuta

Taxon classificationAnimaliaHymenopteraChalcididae

(Linnaeus, 1767)

Figs 54–55

Vespa minuta Linnaeus, 1767: 952 (♀ ? “in Europa australi” (= South Europe) (lectotype designated by Day, 1979) (Linnean Society, London)).
Chalcis minuta ; Fabricius 1787: 272–273.
Brachymeria minuta ; Westwood 1832: 127.
Chalcis pusilla Fabricius, 1787: 272–273 (“Halae Saxonum” (= Halle, Germany), South India (Tamil Nadu: Tranguebar), ♂, neotype India, Tamil Nadu, designated by Bouček and Delvare 1992 (BMNH)); Hübner 1789: 58.
Sphex femoralis Geoffrey (in Fourcroy), 1785: 437 (“France”, (MNHN) (synonymised with Brachymeria minuta (Linnaeus) by Graham (1994)).
Chalcis brevicornis Klug, 1834: 4 (ZMB) (synonymised with Brachymeria minuta (Linnaeus) by Bouček, 1952)).
Chalcis scrobiculata Foerster, 1859: 93 (♀♂, Germany (NHMV) (synonymised with Brachymeria minuta (Linnaeus) by Habu, 1960)).
Chalcis tricolor Foerster, 1859: 98 (♀♂, Germany (NHMV) (synonymised with Brachymeria minuta (Linnaeus) by Habu, 1960)).
Chalcis fumata Thomson, 1876: 18 (Sweden (LUZN) (synonymised with Brachymeria minuta (Linnaeus) by Habu, 1960)).
Chalcis paraplesia Crawford, 1910: 14, 18 (Japan (USNM) (synonymised with Brachymeria minuta (Linnaeus) by Habu, 1960)).
Chalcis jezoensis Matsumura, 1918: 166–167 (Japan (EIHU) (synonymised with Brachymeria minuta (Linnaeus) by Habu, 1960)).
Brachymeria picea Nikol’skaya, 1952: 91 (♂, Russia (ZMMU) (synonymised by Nikol’skaya, 1960, with Brachymeria minuta (Linnaeus)).
Brachymeria putturensis Joseph, Narendran & Joy, 1971: 229–242 (♀, India (ZMUC) (synonymised with Brachymeria minuta (Linnaeus) by Joseph, Narendran & Joy, 1973)).
Brachymeria puturensis longigastralis Joseph, Narendran & Joy, 1971: 232–234 (synonymised with Brachymeria minuta (Linnaeus) by Joseph, Narendran & Joy, 1973)).
Brachymeria fuchuensis Habu, 1962: 19 (♂ (EIHU) (synonymised with Brachymeria minuta (Linnaeus) by Narendran, 1989)).

##### Material.

1 ♀ (RMNH), “**Vietnam**: Ninh Thuân, Núi Chúa N. P., dry south part, Mal traps, 100–180 m, 22–29.v.2007. C. v. Achterberg & R. de Vries, RMNH’07”; 1 ♂ (IEBR), “S. Vietnam: Dóng Nai, Cát Tiên N. P., Dong trail, Mal. traps, c. 100 m, 19–5.iv.2007, Mai Phu Quy & Nguyen Tanh Manh, RMNH’07”.

##### Diagnosis.

*Brachymeria calopeplae* Joseph, Narendran & Joy is close to *Brachymeria minuta* (Linnaeus) but differs by having: 1) pits on middle part of mesoscutum and scutellum mostly as wide as diameter of a pit and smooth and shiny (in *Brachymeria minuta* pits on middle part of mesoscutum and scutellum closer and less than diameter of a pit and partially carinate); 2) yellow part of hind femur almost half of femur (in *Brachymeria minuta* yellow part of hind femur much smaller than that of *Brachymeria calopeplae*); 3) T6 distinctly and deeply pitted (in *Brachymeria minuta* T6 shallowly pitted); 4) parasitoid of *Calopepla leayana* (Coleoptera: Chrysomelidae) (*Brachymeria minuta* so far not reported from Coleoptera). Joseph et al. (1972) described *Brachymeria calopeplae* as a distinct new species and later in 1973 downgraded it as a subspecies of *Brachymeria minuta*. Narendran (1989) treated *Brachymeria calopeplae* as distinct species without formally reinstating its independent old species status. Here we reinstate its species status (status revised) since later studies of more specimens from the hosts *Calopepla leayana* (Latreille) (Coleoptera: Chrysomelidae) showed uniform unique characteristics of this species, which necessitates it to return to its independent species status.

##### Description

(female from Núi Chúa N. P.). ♀, length of body 5.7 mm.

*Colour*. Black; eyes grayish yellow; ocelli pale reflecting yellow; tegula whitish yellow; scape black with base and apex brown, pedicel brownish black; remaining antennal segments black; distal half of mandibles brown; coxae black; trochanters brownish black; femora black with whitish yellow apical part; fore tibia brownish yellow with whitish yellow at basal part and outer apical part, with blackish long patch at outer median part; mid tibia shiny black with base and apex yellow; hind tibia black with subbasal spot and apical part yellow. Pubescence on body grayish white; wings hyaline with veins dark brown.

*Head*. Width of head 1.2 × its height in anterior view; in dorsal view width 3.1 × its length, as long as mesosoma (including tegulae); POL 2.3 × OOL; AOL subequal to OOL; interocular distance 2.4 × POL, vertex and face with close, umbilicate, setigerous pits, interstices carinate and rugose; area below antennal toruli without a raised smooth part; scrobe reaching anterior ocellus; height of malar space 0.4 × height of eye in profile; pre-orbital and post-orbital carina present; post-orbital carina reaching geno-temporal margin. Antenna with radicula 0.1 × length of scape; relative L:W of antennal segments: scape = 15:5; pedicel = 5:4; ring segment = 1:4; F1 = 5:6; F2 = 5:6; F3 = 5:7; F4 = 5:7; F5 = 5:7; F6 = 4:7; F7 = 4:7; clava = 8:7.

*Mesosoma*. Mesosoma with close, umbilicate, setigerous pits, interstices carinate in some places, remainder smooth except with faint micro-sculpture on pronotum; mesoscutum a little longer than scutellum; scutellum wider than long (19:17); apex of scutellum emarginated and bi-lobed; scutellum high in lateral view, gently declined posteriorly; propodeum declined 70° to the vertical axis of scutellum; postspiracular tooth distinct.

*Wings*. Fore wing 2.7 × as long as wide; relative length of CC = 44; SMV = 37; parastigma = 7; MV = 16; PMV = 6; STV = 3.

*Legs*. Hind coxa smooth and shiny on dorsal half, punctate and pubescent on ventral half, without a ventro-mesal tooth; hind femur 1.6 × as long as wide, with an inner basal tooth, outer ventral margin with a row of 13 differently sized teeth.

*Metasoma*. Metasoma longer than mesosoma (33:26); widest before middle; T1 smooth and shiny; T2 with rather distinct micro-sculpture, except on basal and apical narrow areas on ventro-lateral parts, minutely and sparsely pubescent on dorso-basal and dorso-lateral parts; T6 weakly and shallowly pitted and pubescent on basal half, distal half mostly smooth and shiny; ovipositor sheath well visible in dorsal view.

*Male*. See Joseph, Narendran & Joy (1973).

##### Hosts.

Diptera (Calliphoridae, Sarcophagidae, Tachinidae) and Lepidoptera (Arctiidae, Gelechiidae, Hesperiidae, Lasiocampidae, Lymantriidae, Noctuidae, Pieridae, Tortricidae, Yponomeutidae). (For detailed list see Noyes 2011).

##### Distribution.

Old World. New record for Vietnam.

#### Brachymeria neowiebesina sp. n.

Taxon classificationAnimaliaHymenopteraChalcididae

http://zoobank.org/9641DCDA-91CA-4DE0-B9B4-6217BF71CBE4

Figs 56 , 57–58

##### Type material.

Holotype, ♀ (RMNH), “S. **Vietnam**: Dak Lak, Chu Yang Sin N. P., nr dam, c 500 m, 3–9.vi.2007, Mal. traps, C. v. Achterberg & R. de Vries, RMNH’07”. Paratype (IEBR): ♂, “Vietnam: Kom Tum, Chu Mon Ray N. P., Mal. traps, 700–900 m, 26.ix.-5.x.2006, Mal Phu Quy & Nguyen Thanh Manh, RMNH’07”.

##### Diagnosis.

This new species comes near *Brachymeria wiebesina* Joseph, Narendran & Joy in general appearance, but differs from it in having: 1) apex of scutellum deeply emarginate and distinctly bi-lobed (in *Brachymeria wiebesina* apex of scutellum weakly emarginate); 2) scape shorter than combined length of F1, F2 and F3 (in *Brachymeria wiebesina* scape as long as F1, F2 and F3 combined); 3) scape yellow and remaining antennal segments black (in *Brachymeria wiebesina* antenna including scape blackish red or brownish black); 4) upper margin of clypeus smoothly curved and not angulate medially (in *Brachymeria wiebesina* upper margin of clypeus angulate medially); 5) clava as long as wide (in *Brachymeria wiebesina* clava about 1.6 × its width); 6) clava shorter than twice length of F7 (in *Brachymeria wiebesina* clava longer than twice length of F7); 7) outer ventral margin of hind femur with 9 teeth (in *Brachymeria wiebesina* outer ventral margin of hind femur with 13 small teeth) and 8) T1 smooth and shiny, without any trace of minute pits (in *Brachymeria wiebesina* T1 with faint minute punctures on medio-posteriorly).

##### Description.

Holotype, ♀, length of body 3.5 mm.

*Colour*. Black; tegula whitish yellow; antenna black, except yellow scape; fore and hind coxae black, mid coxa brown; all trochanters pale yellowish brown; fore and mid femora dark brown with bases and apices yellow; hind femur black with apex pale yellow; fore tibia yellow with pale brown medially; mid tibia yellow with brown medially; hind tibia dark brown with base and apex pale yellow; tarsi yellow; telotarsi brown; wings hyaline with veins dark brown.

*Head*. Width of head 1.2 × its height in anterior view, 3.1 × its length in dorsal view, as wide as pronotum or mesoscutum (excluding tegulae); parascrobal area not bulging; fronto-vertex 1.4 × eye height in anterior view; malar ridge present; height of malar space 0.4 × eye height in profile; eyes bare; length of eye in profile 0.6 × eye height; pre-orbital and post-orbital carinae present; scrobe not reaching anterior ocellus, separated from anterior ocellus by a diameter of anterior ocellus; POL twice OOL; AOL a little shorter than OOL (5:6); LOL equal to OOL; upper margin of clypeus smoothly curved, not angulate medially, a row of sparse setae in median third, its upper margin raised and removed from antennal sockets by about twice clypeal height in middle; interantennal projection with 6 setae on either side, labrum subangulate on distal margin, with scattered setigerous pits; face and vertex with close, umbilicate setigerous pits; interstices between pits carinate; area below antennal toruli with close pits, median smooth area absent. Antenna with scape not reaching anterior ocellus; radicula 0.3 × length of scape; relative L:W of antennal segments:scape = 9:4; pedicel = 3:4; F1 = 4:5; F2 = 4:5; F3 = 4:6; F4 = 3:6; F5 = 4:6; F6 = 3:6; F7 = 3:6; clava = 6:6.

*Mesosoma*. Mesosoma slightly broader than long (23:20); pronotum almost as broad as mesoscutum; pronotum, mesoscutum and scutellum with close, umbilicate, setigerous pits; interstices carinate; pilosity moderately distributed including apex of scutellum, not very densely pubescent; pronotum with anterior carina moderately strong but confined to sublateral thirds of sclerite; notauli with a row of pits; scutellum slightly convex and gradually sloping to apex, its width subequal to its length; in dorsal view apical rim deeply incised in the middle and bi-lobed; propodeum vertical to scutellum, with large deep pits and alveolae; septa of areolation relatively high; postspiracular teeth indistinct; submedian tooth of anterior margin of mesopleuron weakly developed, metapleuron densely pubescent.

*Wings*. Fore wing 2.4 × as long as its width; relative length of CC = 37; SMV = 32; MV = 19; PMV = 5; STV = 3.

*Legs*. Hind coxa with inner ventro-mesal tooth absent, with close shallow setigerous pits, fully pubescent; hind femur 1.6 × as long as broad (teeth excluded) with 9 ventral marginal teeth, first tooth relatively large, outer disc with close setigerous pits, inner disc with less pits and pubescence.

*Metasoma*. Metasoma ovate; in dorsal view only slightly pointed posteriorly; 0.7 × as wide as long, subequal to length of mesosoma; T1 smooth and shiny, slightly exceeding middle of metasoma, its posterior margin straight; T2 to T5 micro-sculptured and with a single row of setae; T6 with 3–4 cross rows of setigerous pits and pubescence; posterior margins of T2 to T5 concave medially; ovipositor sheath slightly visible in dorsal view, fully pubescent; hypopygium reaching base of T6.

*Male*. Length of body 3.2 mm. Similar to ♀ but metasoma not pointed at apex.

##### Host.

Unknown.

##### Etymology.

Named after *Brachymeria wiebesina* Joseph, Narendran & Joy, because of its superficial resemblance to that species.

#### Brachymeria olethria

Taxon classificationAnimaliaHymenopteraChalcididae

(Waterston, 1914)

Chalcis olethria Waterston, 1914: 257 (♂, Nigeria, Ibadan, (BMNH) (examined)).
Brachymeria olethria ; Narendran 1989: 245, 271.
Brachymeria raoi Joseph, Narendran & Joy, 1972: 21 (♀, India (BMNH) (synonymised with Brachymeria oletria by Narendran 1989)).

##### Material.

2 ♀ (BPBM), “**Vietnam**, 14.x.1960, C.M. Yoshimoto”.

##### Diagnosis.

This species resembles *Brachymeria menoni* Joseph, Narendran & Joy, but differs from it in having: 1) scrobe not reaching anterior ocellus (in *Brachymeria menoni* scrobe reaches anterior ocellus); 2) AOL about 0.5 × of POL (in *Brachymeria menoni* AOL 0.3 × POL); 3) metasoma distinctly longer than pronotum, mesoscutum and scutellum combined (in *Brachymeria menoni* metasoma shorter than pronotum, mesoscutum and scutellum combined).

##### Description.

♀, length of body 4.0–4.2 mm.

*Colour*. Black; tegulae pale yellowish brown; all coxae and trochanter black or brownish black; fore and mid femora black or brownish black with a pale reddish brown or yellow brown tip; hind femur completely black or brownish black with tip pale yellowish brown; fore and mid tibiae brownish black except the brownish black or brownish yellow tip; hind tibia brownish black or black with tip yellowish brown tip; all tarsi pale.

*Head*. Head with scrobe not reaching anterior ocellus; area below scrobe at middle fairly smooth but not clearly demarcated; length of eye 2.3 × its width; AOL about 0.5 × POL; interocular space a little over 2.3 × POL; pre- and post-orbital carinae present; post-orbital carina reaching geno-temporal margin; anterior genal angle nearly rectangular and rounded; hind genal angle rectangular. Antenna with scape not reaching anterior ocellus, longer than F1 to F3 combined; pedicel longer than wide; F1 almost as long as wide; F2, F3, F4 and F5 almost equal in length; clava about 2.1 × as long as F7, and about 1.6 × its width.

*Mesosoma*. Mesosoma reticulate, rounded, umbilicate and close pits; interstices of pits narrower than diameter of a pit, rugose; apex of scutellum weakly emarginate.

*Wings*. Fore wing 2.5–2.6 × as long as wide; MV a little shorter than half SMV; PMV a little shorter than half MV; STV about half of PMV.

*Legs*. Hind coxa without an inner ventro-mesal tooth; hind femur about 1.7 × as long as its width, without an inner basal tooth, outer ventral margin with a row 9–12 differently sized teeth.

*Metasoma*. Metasoma longer than pronotum, mesoscutum and scutellum combined; hardly a little more than twice its height, highest at middle; T1 smooth and shiny, reaching middle length of metasoma; T2 micro-sculptured; T6 rugose with few punctures and sparse pubescence.

*Male*. Similar to ♀ except T1 with delicate reticulation medially; F1 wider than long; apex of scutellum rounded.

##### Hosts.

Lepidoptera (Pyralidae; Gelechiidae, and Momphidae) and Hemiptera (Pseudococcidae) (Noyes 2011).

##### Distribution.

Africa, India, Vietnam, China, Malaysia (Borneo) and Indonesia (Java) (Joseph, Narendran & Joy 1973 and Narendran 1989).

#### Brachymeria podagrica

Taxon classificationAnimaliaHymenopteraChalcididae

(Fabricius, 1787)

Figs 59 , 60–61

Chalcis podagrica Fabricius, 1787: 272 (♀, India, Tamil Nadu, Tranquebar, (lectotype designated by Bouček 1972) (ZMUC)).
Chalcis Fonscolombei Dufour, 1841: 11–19 (♀, France, (lectotype designated by Burks 1936) (MNHN) (synonymised with Brachymeria podagrica by Bouček 1972)).
Chalcis Alphius Walker, 1846: 108. (♂, lectotype (designated by Bouček 1972), India (HDOU) (synonymised with Brachymeria podagrica by Bouček 1972)).
Chalcis xerxena Walker, 1846: 83 (♀, Philippines (lectotype designated by Bouček 1972) (synonymised with Brachymeria podagrica by Bouček 1972)).
Chalcis Amenocles Walker, 1846: 83–84 (♀, Sierra Leone (BMNH) (synonymised with Brachymeria podagrica by Bouček 1972)).
Chalcis restituta Walker, 1862: 351–352 (♀, Jamaica, (lectotype designated by Bouček and Delvare 1992) (BMNH) (synonymised with Brachymeria podagrica by Bouček and Delvare 1992)).
Brachymeria pulchripes Holmgren, 1868: 436 (♂, Philippines (lectotype designated by Bouček 1972) (BMNH) (synonymised with Brachymeria podagrica by Bouček 1972)).
Chalcis mansueta Walker, 1871: 48 (♀, Hong Kong (lectotype selected by Bouček 1972) (BMNH) (synonymised with Brachymeria podagrica by Bouček 1972)).
Chalcis callipes Kirby, 1883: 75 (♀, Japan, (lectotype designated by Bouček 1972) (BMNH) (synonymised with Brachymeria podagrica by Bouček 1972)).
Chalcis mikado Cameron, 1888: 117 (♀, Japan (lectotype designated by Bouček 1972) (synonymised with Brachymeria podagrica by Bouček 1972)).
Chalcis ecentrica Cameron, 1897: 39 (♂, India (lectotype designated by Bouček 1972) (HDOU) (synonymised with Brachymeria podagrica by Bouček 1972).
Chalcis borneanus Cameron, 1905: 52 (Borneo (lectotype designated by Bouček 1972) (BMNH) (synonymised with Brachymeria podagrica by Bouček 1972)).
Chalcis dipterophaga Girault & Dodd (in Girault), 1915a: 320–321 (♂, Australia (QMB) (synonymised with Brachymeria podagrica by Bouček 1988b)).
Chalcis garutianus Gunther (in Haller & Gunther), 1936: 73 (♂, Java (lectotype designated by Bouček 1972) (SFTD) (synonymised with Brachymeria podagrica by Bouček 1972)).
Brachymeria becari Masi, 1929a: 142 (♀, Somalia (BMNH) (synonymised with Brachymeria podagrica by Narendran 1985)).
Chalcis neglecta Masi, 1916: 84 (♀?, Italy (DEI?) (synonymised with Brachymeria fonscolombei (Dufour) by Masi 1951)).
Tumidicoxides kurandaensis Girault, 1913b: 86 (♀, Australia (QMB) (synonymised with Brachymeria podagrica by Bouček 1988b)).
Tumidicoxides paucipunctatus Girault, 1915a: 326 (♀, Australia (QMB) (synonymised with Brachymeria podagrica by Bouček 1988b)).
Chalcis vegai Girault, 1924b: 175 (♀, Australia (QMB) (synonymised with Brachymeria podagrica by Bouček 1988b)).
Brachymeria (Matsumurameria) aligharhensis Husain & Agarwal, 1982b: 499–501 (♀, India, (ZDAMU) (synonymised with Brachymeria podagrica by Narendran 1989)).

##### Material.

2 ♀ (RMNH, IEBR), “S. **Vietnam**: Ninh Thuân, Núi Chúa N. P., northeast part, 90–150 m, 23–30.v.2007, Malaise trap, C. v. Achterberg & R. de Vries, RMNH’07”; 1 ♀ (RMNH), “S. Vietnam: Dóng Nai, Cát Tiên N. P., Dong trail, Mal. traps, c. 100 m, 1–8.iv.2007, Mai Phu Quy & Nguyen Tanh Manh, RMNH’07”; 15 ♀ (BPBM), “Vietnam, [locality unknown], 8–26.xi.1960 & 11.xii.1960, C.M. Yoshimoto”; 2 ♀ (BPBM) “Vietnam, [locality unknown], 13.v.1960, S. Quate”;1 ♀ (BPBM), “Vietnam, [locality unknown], 6.vii.1961, N.R. Spencer”.

##### Diagnosis.

This species is very close to *Brachymeria minuta* considering the head and the mesosoma and the colour pattern of the hind tibia. However, it differs from *Brachymeria minuta* in having: 1) hind femur 1.8–2.1 × as long as wide (in *Brachymeria minuta* hind femur less than 1.7 × as long as wide); 2) dorsal side of hind femur in lateral view dilated straightly from base to widest part, hence also straightly contracted towards apex so that the dorsal side is weakly angulate (in *Brachymeria minuta* the dorsal side of hind femur in lateral view not dilated but rounded from base to apex); and 3) the apical whitish patch on hind femur is generally limited on outer dorsal side, not extending on to inner side (in *Brachymeria minuta* apical yellow patch extend also to inner side).

##### Description.

♀, length of body 5–7 mm.

*Colour*. Black with following parts as follows: mandibles brown or dark brown with base and apex often reddish brown; antenna black or blackish brown or reddish sometimes partially or wholly; funicle sometimes faintly reddish; tegulae almost white or pale yellow; fore and mid coxae almost shiny black; hind coxa black or reddish brown; trochanters black or brown or reddish; fore and mid femora black or brownish red with tips pale yellow or creamy white; hind femur brownish red with apex white or yellow, white colour usually not extending to inner side; fore and mid tibiae reddish brown with pale yellow or white at base and apex; hind tibia reddish brown or black with yellow or white subbasally and apically.

*Head*. Head with pre- and post-orbital carinae present; post-orbital carina reaching geno-temporal margin; scrobe reaching anterior ocellus; area below interantennal projection smooth medially; POL a little over twice OOL; AOL 0.3 × POL; minimum interocular distance at vertex 2.7 × POL. Malar ridge present, height of eye in profile 2.4–3.3 × height of malar space in lateral view; anterior and posterior genal angles rectangular or subrectangular. Antenna: scape not exceeding anterior ocellus, as long as combined length of F1 to F4 or a little shorter; pedicel almost as long as wide; F1 almost as long as wide; F2 to F7 slightly increasing in width and decreasing in length; clava more than twice as long as F7.

*Mesosoma*. Mesosoma distinctly pitted dorsally; pits generally becoming somewhat larger and sparse posteriorly; interstices rather carinate on pronotum but almost flat and smooth on other parts; scutellum rather gently declined posteriorly, apex somewhat widely explanate and reflexed, distinctly emarginate and bi-lobed; propodeum with postspiracular tooth on either side present.

*Wings*. Fore wing 2.6–2.7 × as long as wide. MV 0.5–0.6 × as long as SMV; PMV one-fourth as long as MV, twice as long as STV or somewhat less.

*Legs*. Hind coxa with distinct dense punctures and pubescence on ventral side, without an inner ventro-mesal tooth; hind femur 1.8–2.1 × as long as wide; with one inner basal tooth or protuberance; outer ventral margin with a row of 9–11 differently sized teeth.

*Metasoma*. Metasoma slightly longer than pronotum, mesoscutum and scutellum combined, widest at middle; T1 smooth; T2 with sparse minute setigerous punctures on baso-dorsal part except narrow part at middle, punctures extending posteriorly at latero-dorsal parts, on dorsal half of lateral parts punctures denser and larger, micro-sculpture distinct; T3 and T4 with transverse line of several setae dorsally with rather dense punctures and setae dorsally of sides, micro-sculpture distinct dorsally and on dorsal half of lateral part; T6 shallowly pitted, micro-sculpture distinct; ovipositor sheath visible in dorsal view.

*Male*. Length of body 3.5–5.0 mm. Legs somewhat darker in colour than in female; funicle with trichoid sensillae on ventral side.

##### Hosts.

Primary parasitoid of blowflies (Sarcophagidae) and other Diptera viz., Calliphoridae, Muscidae, Tephritidae and of Lepidoptera (Psychidae, Yponomeutidae and Lymantriidae).

##### Distribution.

Cosmopolitan (and known from Vietnam).

##### Variation.

The size of the interstices of pits on mesoscutum and scutellum are very variable in this species from carinate to wider than diameter of pits. In some very rare cases the hind femur is black with apex yellow and in such cases it is likely to be confused with *Brachymeria minuta* (Linnaeus).

#### Brachymeria scutellocarinata

Taxon classificationAnimaliaHymenopteraChalcididae

Joseph, Narendran & Joy, 1972

Figs 62 , 63–64

Brachymeria scutellocarinata Joseph, Narendran & Joy, 1972: 45 (♀, Java (RMNH)); Narendran 1989: 241, 255 (keyed and commented).

##### Material.

1 ♀ (RMNH), “S. **Vietnam**: Dóng Nai, Cát Tiên N. P., ca 100 m, 13–20.v.2007, Malaise traps 25–29, eco-trail, C. v. Achterberg & R. de Vries, RMNH’07”.

##### Diagnosis.

This species comes near *Brachymeria ambonensis* Narendran in the key to species by Narendran (1989), but differs from that species in having: 1) scutellum with a single median carina (in *Brachymeria ambonensis* scutellum with more than one longitudinal carina; 2) pre-orbital carina joins scrobal margin (in *Brachymeria ambonensis* pre-orbital carina not meeting scrobal margin); 3) hind tibia yellow (in *Brachymeria ambonensis* hind tibia black with apex yellow) and 4) basal part of hind femur black (in *Brachymeria ambonensis* basal part of hind femur reddish).

##### Description

(female from Cát Tiên N. P.). ♀, length of body 6.2 mm.

*Colour*. Black with following parts as follows: eyes dull yellow; ocelli pale brownish yellow; tegula pale brownish white; fore and mid trochanters pale brown; apical half of fore and mid femora, apex of hind femur, all tibiae and tarsi whitish yellow; apex of tarsal claws and arolium dark brown; fore wing hyaline, veins dark brown.

*Head*. Width of head in anterior view 1.4 × its height; width in dorsal view 2.5 × its length, subequal in width to mesosoma (excluding tegulae); POL 3 × OOL; AOL equal to OOL; interocular space 2.8 × POL; scrobe reaching anterior ocellus; pre-orbital carina running upwards on face from malar ridge and converging and meeting scrobal margin; post-orbital carina present reaching geno-temporal margin; vertex and face with close, setigerous pits, interstices narrow and micro-sculptured, narrower than diameter of a pit; area below interantennal projection with a narrow smooth strip-like area; height of malar space twice eye height in profile; eye height 1.7 × its length in profile; eyes bare; anterior and posterior genal angles subrectangular. Antenna with scape not quite reaching anterior ocellus; radicula 0.2 × length of scape; tip of clava with micro-sensillae. Relative L:W of antennal segments:scape = 20:5; pedicel = 5:5; ring segment = 1:4; F1 = 7:8; F2 = 7:8; F3 = 6:8; F4 = 7:9; F5 = 6:9; F6 = 8:10; F7 = 6:10; clava = 9:10.

*Mesosoma*. Mesosoma with rounded, umbilicate, setigerous, close pits; interstices carinate and rugose; scutellum with a median longitudinal raised ridge; pronotum 3.1 × as broad as long; anterior carina absent medially; middle lobe of mesoscutum 1.2 × its width, longer than scutellum (15:12); scutellum wider than long (15:12), apex shallowly but widely emarginated with a row of dense pubescence; propodeum with large irregular foveolae, vertical to the axis of scutellum; postspiracular tooth indistinct.

*Wings*. Fore wing 2.6 × as long as broad; relative length of CC = 21; SMV = 17; parastigma = 4; MV = 10; PMV = 3; STV = 2.

*Legs*. Hind coxa smooth and shiny dorsally, ventrally with dense minute pits and pubescence, inner ventro-mesal tooth on hind coxa absent; hind femur 1.6 × as long as broad, with an inner basal tooth; outer ventral margin of hind femur with a row of 12 differently sized teeth.

*Metasoma*. Metasoma slightly shorter than mesosoma (28:30), 1.5 × as long as high; T1 smooth and shiny, its posterior margin entire; T2 shagreened with setigerous pits on latero-dorsal part, its posterior margin slightly concave; posterior margin of T3 slightly concave, shagreened; T4 smooth and shiny with a row of setae on posterior margin, longer than T3; T5 about half as long as T5, with a setigerous cross row of pits with the posterior margin, posterior margin entire; T6 vertical, with 5–6 cross rows of setigerous pits. Ovipositor sheath not visible in dorsal view.

*Male*. Unknown.

##### Host.

*Sturmia* sp. (Tachinidae) pupa (Narendran 1989).

##### Distribution.

Vietnam (new record), Indonesia (Java), Malaysia (Borneo), India.

#### Brachymeria semirusula sp. n.

Taxon classificationAnimaliaHymenopteraChalcididae

http://zoobank.org/7D1ED683-ACFF-474C-BC25-D62D4E5E8FDF

Figs 65 , 66–67

##### Type material.

Holotype, ♀ (RMNH), “S. **Vietnam**: Dak Lak, Chu Yang Sin N. P., n[ea]r dam, c. 500 m, 3–9.vi.2007, Mal. traps, C. v. Achterberg & R. de Vries, RMNH’07”.

##### Diagnosis.

This new species resembles *Brachymeria croceogastralis* Joseph, Narendran & Joy in having metasoma with red colour, but differs from *Brachymeria croceogastralis* in having: 1) hind tibia black with a faint brown subbasal spot and yellow apex (in *Brachymeria croceogastralis* hind tibia whitish yellow with 0.4 length of tibia from base black without any subbasal spot and the black colour extending to apex through ventral margin); 2) hind coxa and femur black with apex of femur pale yellow (in *Brachymeria croceogastralis* hind coxa and femur liver brown with apex yellow); 3) T1 not reaching middle of metasoma (in *Brachymeria croceogastralis* T1 reaching middle of metasoma); 4) area below scrobe punctate (in *Brachymeria croceogastralis* area below scrobe narrow and smooth medially; 5) malar space 0.6 × height of eye (in *Brachymeria croceogastralis* malar space 0.3 × height of eye); 6) clava 1.2 × as long as wide (in *Brachymeria croceogastralis* clava 1.6 × as long as wide) and 7) fore wing 2.7 × as long as wide (in *Brachymeria croceogastralis* fore wing less than 2.5 × as long as wide).

##### Description.

Holotype, ♀, length of body 4.1 mm.

*Colour*. Black with following parts as follows: eyes grayish yellow with reflecting yellow spots; ocelli pale reflecting yellow; antenna reddish pink; tegula whitish yellow; all coxae and femora black with apices of femora yellow; fore and mid tibiae yellow with slight darker patch at middle; hind tibia black with a weak brown subbasal spot and yellow apex; tarsi yellow; metasoma red with black dorsally on median part of T1, and anterior halves of T2 to T5. Pubescence silvery on head and mesosoma but golden on metasoma.

*Head*. Width of head 1.2 × its height in dorsal view, 2.5 × as broad as long in dorsal view, 1.1 × as broad as pronotum; parascrobal area not bulging; fronto-vertex 1.2 × eye height in anterior view; malar ridge present; malar space 0.4 × eye height in profile; pre-orbital carina absent; post-orbital carina slightly branched, reaching geno-temporal margin; scrobe almost reaching anterior ocellus; POL 2.3 × OOL; AOL equal to OOL; LOL a little longer than OOL (4:3); width of interocular space 2.9 × POL; face and vertex with close umbilicate, setigerous pits, interstices rugulose, narrower than diameter of a pit; interantennal projection shortly grooved, with 6 setae on either side; upper margin of clypeus angulate medially, not smoothly curved, with scattered pubescence, raised and removed from antennal sockets by about clypeal height in middle. Antennal formula 11173; scape not reaching anterior ocellus; relative L:W of antennal segments:scape = 11:3; pedicel = 2:3; F1 = 4:4; F2 = 3:4; F3 = 3:4; F4 = 3:4; F5 = 3:5; F6 = 3:5; F7 = 3:5; clava = 6:5.

*Mesosoma*. Mesosoma 1.1 × as long as broad (at mesoscutum); mesoscutum as broad as pronotum, interstices carinate and most areas with micro-reticulations; pilosity dense all over including apex of scutellum; pronotum with anterior carina moderately strong, confined to sublateral thirds of sclerite; notauli slightly curved, distinctly groove like with pits; scutellum slightly convex and gradually sloping to apex, its width sub-equal to its length, in dorsal view apical rim slightly emarginate, subvertical to scutellum; propodeum with large deep pits and alveolae; septa of areolation relatively high; postspiracular teeth absent; submedian tooth of anterior margin of mesopleuron distinct; metapleuron densely pubescent.

*Wings*. Fore wing 2.7 × as long as its breadth; relative length of CC = 27; SMV = 19; MV = 13; PMV = 6; STV = 3.

*Legs*. Hind coxa without any tooth; ventral side closely punctate and pubescent; hind femur twice as long as broad (excluding teeth), with 10 teeth on outer ventral margin, externally and internally densely punctate with dense short pubescence.

*Metasoma*. Metasoma ovate, only slightly pointed posteriorly in dorsal view, 0.7 × as broad as long, subequal to length of mesosoma; T1 slightly shorter than half as long as metasoma, smooth, lateral pubescence very fine with a very few setae present as a small lateral patch; T2 micro-reticulate with a few setigerous pits laterally; T3 to T5 with 2 rows of golden pilosity on each tergite; T6 with 7 cross rows of pits, each pit with a seta, interstices of pits narrower than diameter of a pit, with micro-sculpture; epipygium 0.4 × length of T6, without distinct median carina; ovipositor sheath hardly visible in dorsal view, with a bunch of setae at apex; hypopygium reaching T5.

*Male*. Unknown.

##### Host.

Unknown.

#### Brachymeria shansiensis

Taxon classificationAnimaliaHymenopteraChalcididae

Habu, 1961

Figs 68–70

Brachymeria shansiensis Habu, 1961: 80–82 (♀, holotype, China (EIHU); Joseph, Narendran & Joy, 1970: 22–23. (keyed and redescribed); Narendran 1989: 239, 267 (keyed).

##### Material.

1 ♀ (RMNH), “**Vietnam**: Ninh Thuân, Núi Chúa N. P., dry south part; Mal[aise] traps, 100–188 m, 22–29.v.2007, C. v. Achterberg & R. de Vries, RMNH’07”.

##### Diagnosis.

This species is very similar to *Brachymeria excarinata* Gahan by having similar colour and no post-orbital carina. However, it differs from *Brachymeria excarinata* in having T1 shagreened (sculpture absent in *Brachymeria excarinata*), eyes with sparse pubescence (without pubescence) and the subbasal yellow colour of hind tibia much further developed than that of *Brachymeria excarinata*.

##### Description

(female from Núi Chúa N. P.). ♀, length of body 4.2 mm.

*Colour*. Black with following parts as follows: eyes pale grayish yellow with reflecting yellow spots; ocelli reflecting pale yellow; tegula yellowish white; all coxae concolorous with mesosoma; trochanters reddish brown; femora black with apex yellow; fore and mid tibiae pale yellowish white; hind tibia yellowish white with brownish or dark brown median band (Fig. 68); all tarsi white; telotarsi brown, its apical pulvilli with whitish pads; wings hyaline with veins brown.

*Head*. Width of head in anterior view 1.4 × as broad as its length; width in dorsal view 2.9 × as broad as long, as broad as mesoscutum (excluding tegulae); face and vertex with close setigerous pits, interstices micro-sculptured, narrower than half diameter of a pit. POL 4 × OOL; AOL longer than OOL (7:5); interocular width 2.1 × POL; scrobe smooth and shiny, reaching anterior ocellus; height of malar space 0.4 × height of eye in profile; eyes sparsely pubescent; height of eye 1.6 × eye length in profile; anterior genal angle subhorizontal; posterior genal angle obtuse subhorizontal; pre-orbital carina present; post-orbital carina absent; area below interantenal projection narrowly smooth. Antenna with radicula 0.2 × length of scape; scape not quite reaching anterior ocellus. Relative L:W of antennal segments:scape = 16:5; pedicel = 4:4; ring segment = 1:4; F1 = 5:6; F2 = 5:7; F3 = 5:7; F4 = 5:7; F5 = 5:7; F6 = 5:8; F7 = 4:7; clava = 9:6.

*Mesosoma*. Pronotum with anterior marginal carina separating collar absent medially, posterior margin a little concave, surface with close umbilicate setigerous pits, interstices sculptured, somewhat carinate; mesoscutum with close pits as in pronotum, apex entirely or largely rounded. Propodeum with median longitudinal foveola and sublateral foveolae present; postspiracular tooth hardly district.

*Wings*. Fore wing 2.7 × as long as wide; speculum slightly open behind; relative length of CC = 24; SMV = 11; parastigma = 2; MV = 10; PMV = 5; STV = 2.

*Legs*. Hind coxa densely punctate and pubescent on ventral side without an inner ventro-mesal tooth; hind femur 1.9 × as long as wide, outer disc mat like with dense minute pubescence, outer ventral margin with a raw of 14 differently sized teeth, without an inner basal tooth; second hind tarsal segment distinctly longer than wide in dorsal view (Fig. 70).

*Metasoma*. Metasoma slightly longer than mesosoma (22:21); T1 faintly shagreened; T2 with a patch of pubescence on each side dorsally; T3 to T5 with cross rows of setae dorsally and sides. T6 with 6–7 cross rows of setigerous pits, interstices reticulate. Ovipositor sheath visible in dorsal view, a little shorter than epipygium in dorsal view.

*Male*. Similar to female, but metasoma slightly shorter than or nearly as long as mesosoma.

##### Distribution.

China, India, Vietnam (new record).

##### Variation.

In some specimens entire base of hind tibia reddish brown while in a few others reddish brown only latero-basally.

#### Brachymeria taiwana

Taxon classificationAnimaliaHymenopteraChalcididae

(Matsumura, 1911)

Chalcis taiwanus Matsumura, 1911: 149 (♀ lectotype, Taiwan (EIHU) (lectotype designated by Habu 1960)).
Brachymeria (Matsumurameria) taiwana ; Habu 1960: 209.
Brachymeria clypealis Joseph, Narendran & Joy, 1970: 25 (♀, holotype, India (NZSI) (synonymised with Brachymeria (Matsumurameria) taiwana (Matsumura) by Joseph, Narendran & Joy, 1973)).
Brachymeria flagellata Joseph, Narendran & Joy, 1971: 234 (♀, holotype, India (NHRM) (synonymised with Brachymeria (Matsumurameria) taiwana (Matsumura) by Joseph, Narendran & Joy, 1973)).

##### Material.

2 ♀ (RMNH), “S. **Vietnam**: Dóng Nai, Cát Tiên N. P., Dong trail, Mal[aise] traps, c. 100 m, 1–8.iv.2007, Mai Phu Quy & Nguyen Tanh Manh, RMNH’07”; 1 ♀ (IEBR), id., but 7.x.2005, Crocodile trail; 1 ♀ (RMNH), id., but 14–20.v.2007, Malaise traps 20–23, *Lagerstroemia* trail; 1 ♀ (BPBM), “Vietnam, [locality unknown], 6.vii.1961, N.R. Spencer”.

##### Diagnosis.

This species resembles Brachymeria (Matsumurameria) criculae (Kohl) in the colour pattern of the hind tibia and in having the apex of the scutellum rounded. However, it differs from *Brachymeria criculae* in having the post-orbital carinae present (in *Brachymeria criculae* post-orbital carina absent); hind femur black (in *Brachymeria criculae* hind femur red or orange red or blackish red) and metasoma black (in *Brachymeria criculae* metasoma liver brownish red).

##### Description

(females from Vietnam). ♀, length of body 4.5–4.6 mm.

*Colour*. Black with following parts as follows: coxae black with apices brownish; fore and mid trochanters brown or reddish brown; hind trochanters black; all femora black with apices yellow; fore and mid tibiae yellow with ventral median part black; hind tibia yellow with base black which extends through ventral margin to distal end with a slightly broader part in middle; wings hyaline with veins dark brown.

*Head*. Head wider than its height in anterior view; in dorsal view slightly wider than mesosoma, scrobe nearly reaching anterior ocellus; ventral part of face with dense pubescence; upper margin of clypeus confluent with face; pre-orbital carinae hardly distinct; post-orbital carina reaching geno-temporal margin; POL 2.3 × OOL, diameter of posterior ocellus a little more than OOL; geno-temporal furrow indistinct; height of malar space 0.3 × height of eye in profile. Antenna with scape reaching anterior ocellus but not exceeding it, almost as long as length of F1 to F4 combined; F1 slightly longer than wide; F2 as long as wide, slightly wider but shorter than F1; clava twice as long as F7.

*Mesosoma*. Mesosoma with close umbilicate setigerous pits, pits relatively small; pits on posterior part of mesoscutum and on scutellum somewhat deep; pits faint on scapulae (especially near notauli) and on axillae; interstices somewhat carinate only on pronotum and on posterior part of mesoscutum; interstices relatively wide on other parts, faintly reticulate or striate on mesoscutum; scutellum with apex rounded; propodeum without tooth laterally.

*Wings*. Fore wing 2.5 × as long as wide; PMV 0.3–0.5 × as long as MV.

*Legs*. Hind coxa without an inner ventro-mesal tooth; hind femur more than 1.5 × as long as wide with about fifteen teeth on outer ventral margin, teeth relatively small, without an inner basal tooth.

*Metasoma*. Metasoma subequal in length to mesosoma or shorter than mesosoma; T1 smooth and shiny; remaining tergites with dense setigerous pits; ovipositor sheath not visible in dorsal view.

*Male*. Similar to female except for a little stouter antenna.

##### Host.

Unknown.

##### Distribution.

India, Vietnam, Indonesia (Java), China (Taiwan) (Joseph, Narendran & Joy 1973).

#### Brachymeria wiebesina

Taxon classificationAnimaliaHymenopteraChalcididae

Joseph, Narendran & Joy, 1972

Brachymeria wiebesina Joseph, Narendran & Joy, 1972: 35–37 (♀, holotype, Malaysia (Sarawak) (BPBM)); Narendran 1989: 245, 266 (keyed and commented).

##### Material.

3 ♂ (BPBM), “**Vietnam**, [locality unknown], 7.vii.1961. N.R. Spencer”.

##### Diagnosis.

This species comes near *Brachymeria olethria* (Waterston) in the key to species by Narendran (1989), but differs from *Brachymeria olethria* in having: 1) scape subequal or as long as combined length of F1 to F3 (in *Brachymeria olethria* scape distinctly longer than combined length of F1 to F3); and 2) area below scrobe punctate (smooth in *Brachymeria olethria*).

##### Description

(after Joseph, Narendran and Joy 1973). ♀, length of body 2.7 mm.

*Colour*. Black; tegula dull yellow; antenna blackish red or sometimes brownish black; coxae, trochanters and femora black except tips of femora which are yellow; fore tibia yellow with a pale brownish colour at middle; tarsi pale yellow; hind tibia pale blackish-brown with a yellowish patch at the tip and at base; hind tarsi pale yellow.

*Head*. Head almost as wide as mesosoma, a little less than one and a half times its own length; surface of head faintly pitted, interstices and inside of pits rugulose; scrobe not reaching anterior ocellus; pre- and post-orbital carinae present; anterior ocellus slightly larger than a hind ocellus; area below scrobe punctate; distance between anterior and posterior ocelli slightly more than one-third POL; POL 3 × OOL; interocular distance two and one-third POL. Scape not exceeding anterior ocellus, as long as combined length of F1+F2+F3; F1 and F2 almost equal in length; clava a little more than twice length of preceding segment.

*Mesosoma*. Mesosoma with small, umbilicate, close pits, interstices narrower than diameter of pits, rugose; apex of scutellum weakly emarginated.

*Wings*. Fore wing about 2.5 × its width; PMV about one-third MV.

*Metasoma*. Metasoma hardly longer than combined length of head, pronotum, mesoscutum and scutellum. T1 smooth and shiny, reaching middle of metasoma. Ovipositor sheath visible in dorsal view.

*Male*. Resembles female in almost all features except stouter antenna and shorter metasoma.

##### Host.

A sweet potato beetle (Coleoptera: Chrysomelidae).

##### Distribution.

India, Malaysia (Sarawak), Thailand, Philippines, Singapore and Vietnam (Narendran 1989).

#### Dirhinus

Taxon classificationAnimaliaHymenopteraChalcididae

Dalman, 1818

Figs 71–72 , 73–74 , 75–76 , 77–78 , 79–80 , 81–82 , 83 , 84–85

Dirhinus Dalman, 1818: 75–76. Type species: Dirhinus excavatus Dalman; by monotypy.
Eniaca Kirby, 1883: 57. Type species: Chalcis cornigera Jurine, original designation. Synonymised with Dirhinus by Masi (1919).
Hontalia Cameron, 1884: 112–113. Type species: Hontalia caerulea Cameron; by monotypy. Synonymised with Dirhinus by Burks (1936).
Dirrhinoidea Girault, 1912: 165–166. Type species: Dirrhinoidea maculata Girault; by original designation. Synonymised with Dirhinus by Masi (1947).
Pareniaca Crawford, 1913: 312. Type species: Pareniaca schwarzi Crawford; by original designation. Synonymised with Dirhinus by Burks (1936).
Eniacella Girault, 1913c: 35. Type species: Eniacella rufricornis Girault, by monotypy. Synonymised with Dirhinus by Masi (1947).
Eniacomorpha Girault, 1915a: 354. Type species: Eniacomorpha vultura Girault; by monotypy. Synonymised with Dirhinus by Bouček and Narendran (1981).
Dirhinoides Masi, 1947: 49. Type species: Dirhinus pachycerus Masi, original designation. Synonymised with Dirhinus by Bouček and Narendran (1981).

##### Diagnosis.

This genus belongs to the subfamily Dirhininae which contains another genus *Aplorhinus* Masi (from Borneo) which differs from *Dirhinus* in having the scutellum with a median projection posteriorly, exceeding base of T1, the hind femur with an unusually large basal tooth and T1 covering almost the entire metasoma.

##### Description.

Head with face produced into two strong, edged horns; fore wing with MV unusually long, but PMV and STV rudimentary. Metasoma with striate petiole. Hind femur beneath with smoothly arched comb of minute teeth.

##### Hosts.

Diptera (Calliphoridae, Sarcophagidae, Muscidae, Glossinidae and Tephritidae).

##### Distribution.

Asia, Australia, Europe and New World.

##### Key to Vietnamese species of *Dirhinus* Dalman (based on females)

**Table:** 

1	Apical half of antenna of female strongly widened apically (Fig. 80), clava unusually swollen (basal claval segment more than twice as broad as long); clava with an area of micro-pilosity on one side	**2**
–	Apical half of antenna of female slightly widened apically (Figs 72, 76), clava not unusually swollen or broad; width of basal segment of clava less than twice its length; without an area of micro-pilosity	**3**
2	Each frontal horn with a transverse anterior margin, without a notch apically; mesoscutum and scutellum densely punctate without any broad smooth areas; apex of scutellum with a tooth-like protuberance (Fig. 93)	***Dirhinus neoclaviger* sp. n.**
–	Each horn with a notch apically, not with transverse margin; mesoscutum and scutellum with broad smooth areas on scapulae and scutellum, punctures sparse in middle of mesoscutum; apex of scutellum without a tooth-like protuberance (Fig. 79)	***Dirhinus claviger* Bouček & Narendran**
3	Frontal horn robust (Fig. 73), less protruding in front of eyes; apical antennal segments brown	***Dirhinus anthracia* Walker**
–	Frontal horn slenderer (Fig. 76), distinctly protruding in front of eyes; apical antennal segments dark brown or black	**4**
4	Median areola of propodeum almost parallel-sided (Fig. 78); each frontal horn in dorsal view at anterior ocular line slightly narrower than scrobal gap anteriorly (Fig. 77)	***Dirhinus auratus* Ashmead**
–	Median areola of propodeum not parallel-sided anteriorly; each frontal horn in dorsal view at anterior ocular line not narrower than scrobal gap anteriorly; [not yet found in Vietnam]	***Dirhinus secundarius* Masi**

**Figures 71–72.**
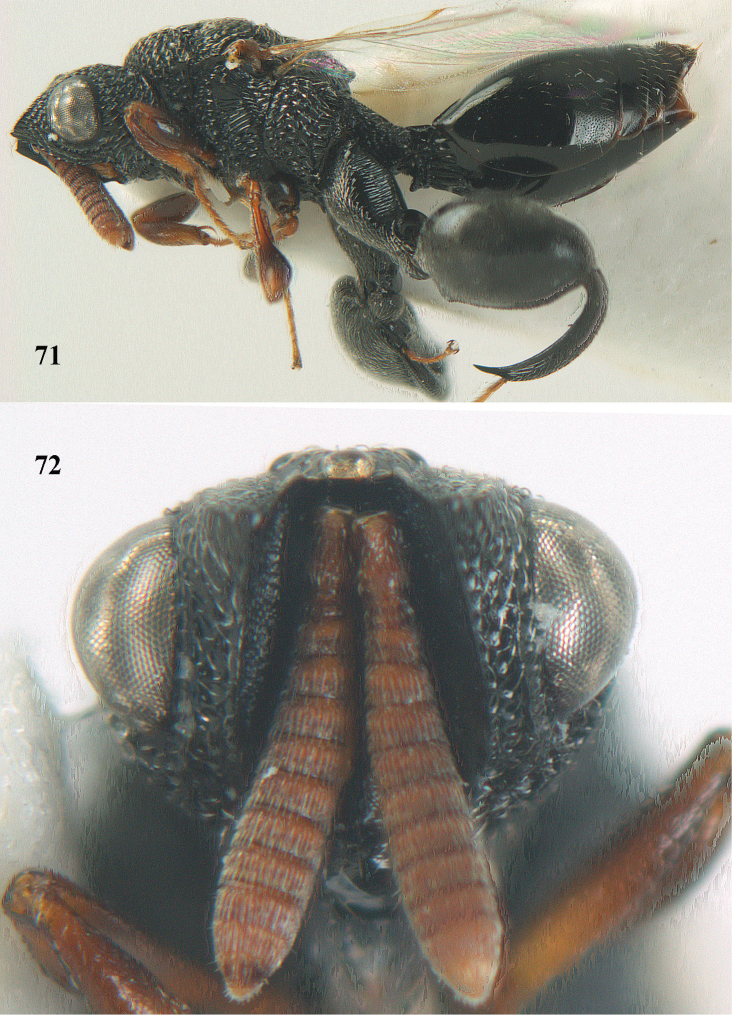
*Dirhinus anthracia* Walker, ♀, Núi Chúa N. P. **71** habitus lateral **72** head anterior.

**Figures 73–74.**
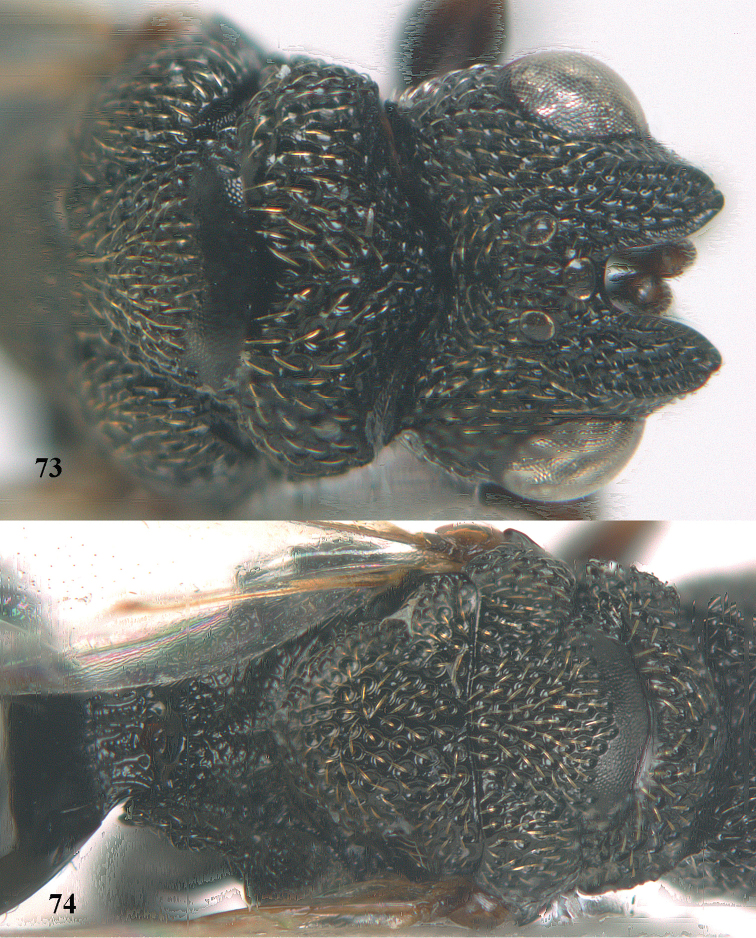
*Dirhinus anthracia* Walker, ♀, Núi Chúa N. P. **73** head dorsal **74** mesosoma dorsal.

**Figures 75–76.**
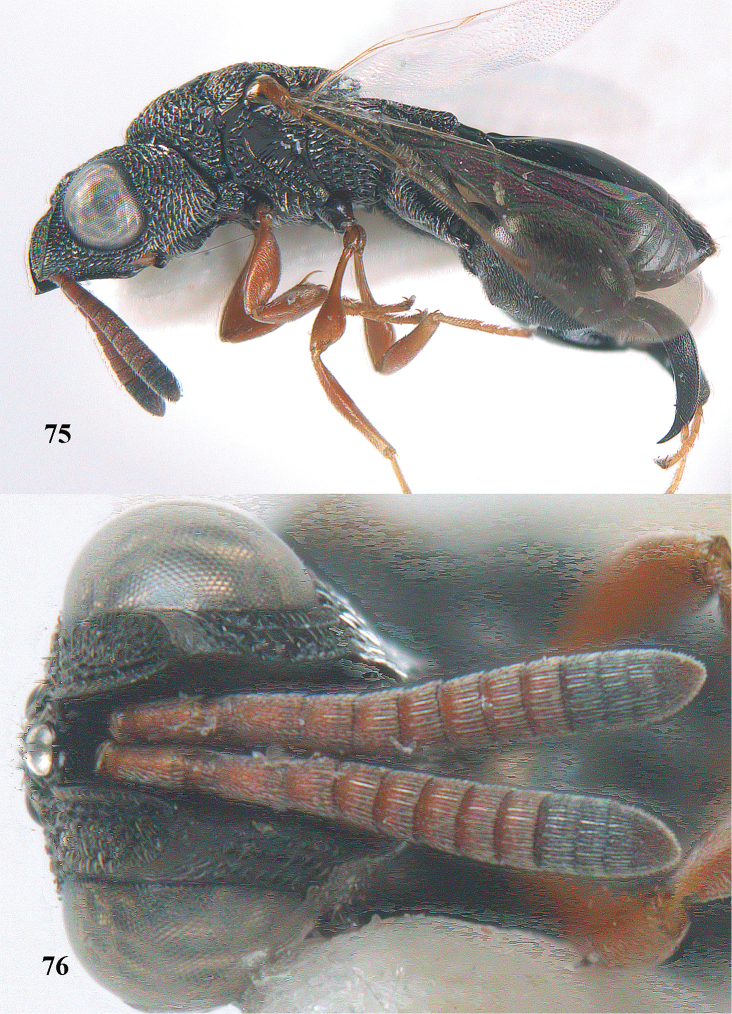
*Dirhinus auratus* Ashmead, ♀, Núi Chúa N. P. **75** habitus lateral **76** head anterior.

**Figures 77–78.**
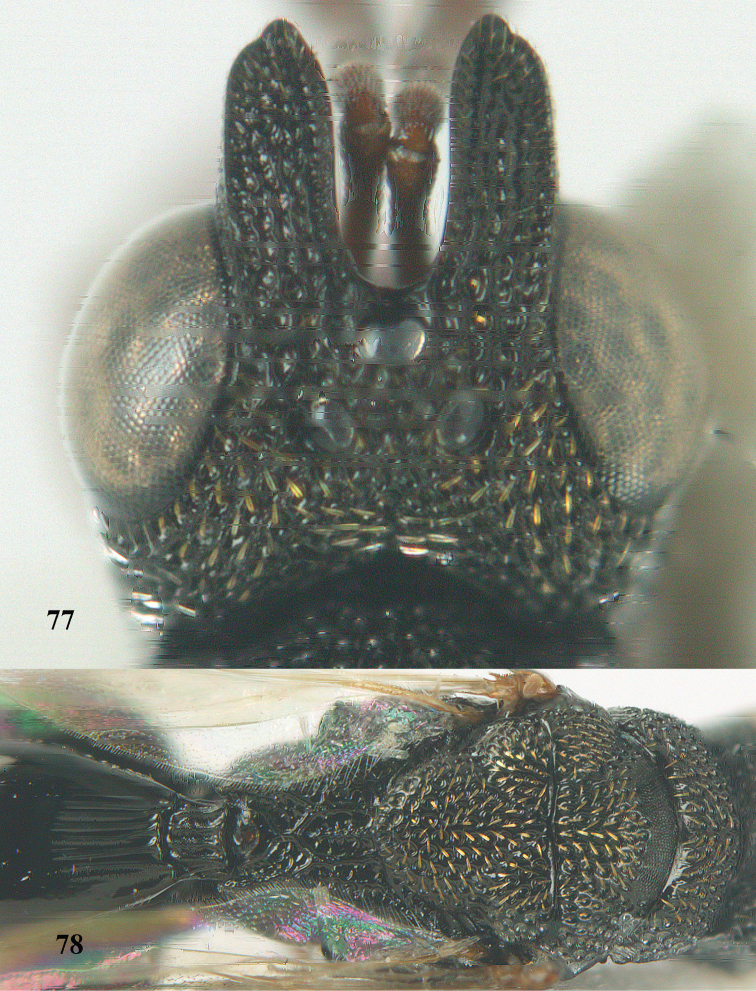
*Dirhinus auratus* Ashmead, ♀, Núi Chúa N. P. **77** head dorsal **78** mesosoma and base of metasoma dorsal.

**Figures 79–80.**
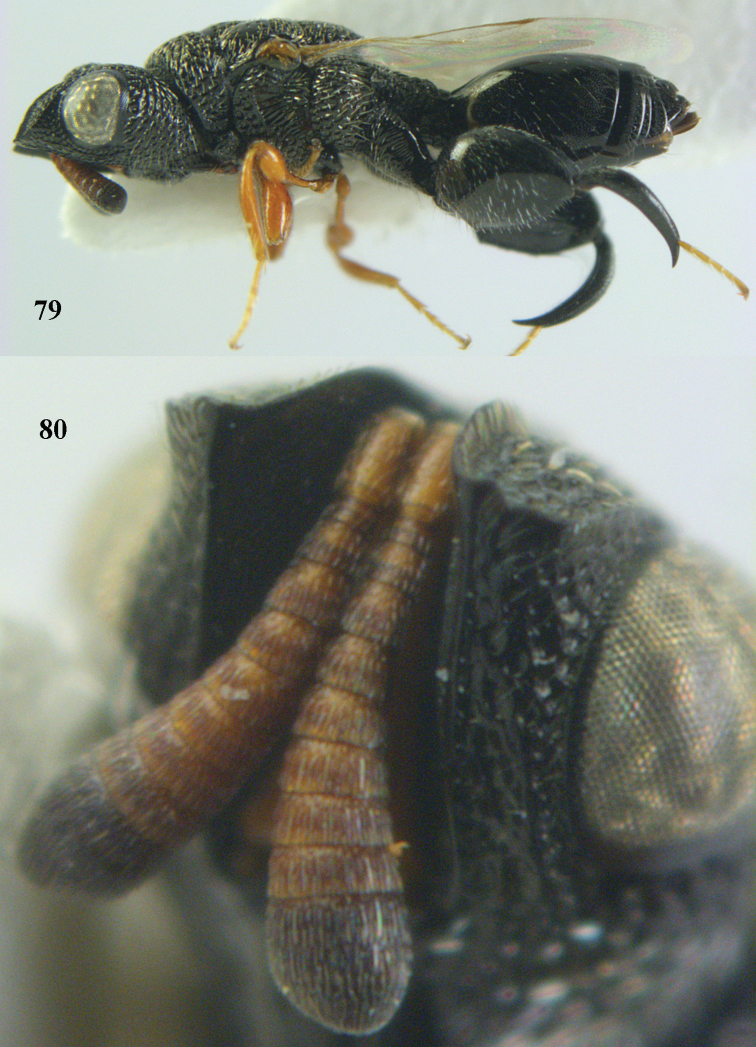
*Dirhinus claviger* Bouček & Narendran, ♀, Núi Chúa N. P. **79** habitus lateral **80** head, latero-anterior.

**Figures 81–82.**
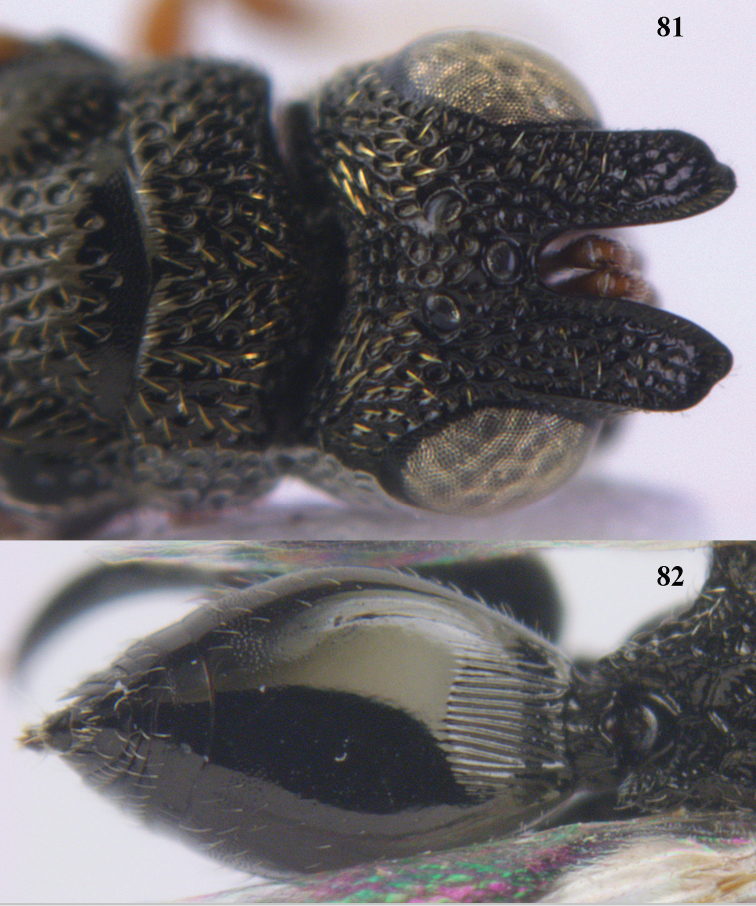
*Dirhinus claviger* Bouček & Narendran, ♀, Núi Chúa N. P. **81** head dorsal **82** metasoma dorsal.

**Figure 83.**
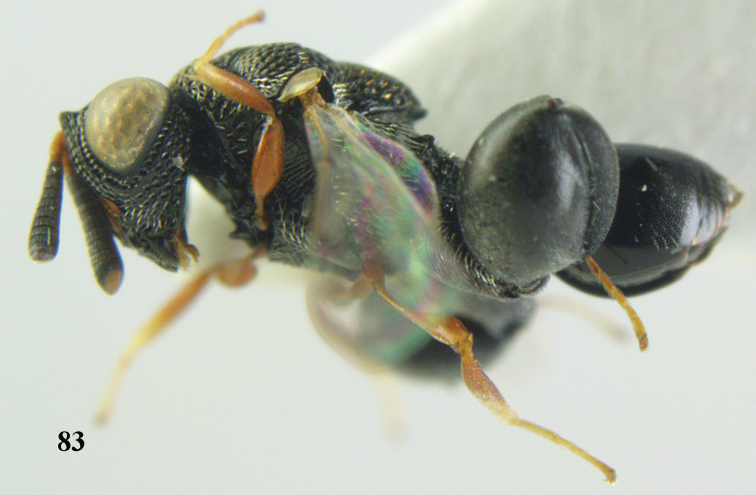
*Dirhinus neoclaviger* sp. n., ♀, holotype, habitus lateral.

**Figures 84–85.**
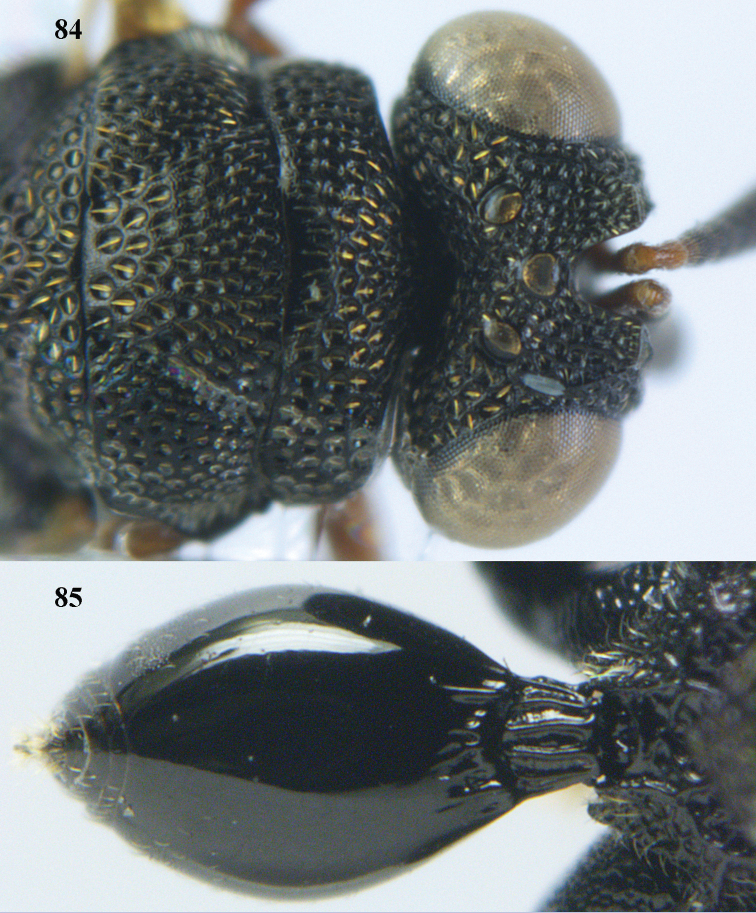
*Dirhinus neoclaviger* sp. n., ♀, holotype. **84** head dorsal **85** metasoma dorsal.

#### Dirhinus anthracia

Taxon classificationAnimaliaHymenopteraChalcididae

Walker, 1846

Figs 71–72 , 73–74

Dirhinus anthracia Walker, 1846: 7, 85; Narendran 1989: 294–295 (examined).
Eniacella rufricornis Girault, 1913c: 35; Bouček and Narendran 1981: 239 (synonymised by Bouček and Narendran 1981 with Dirhinus anthracia Walker).
Eniacella bicornuticeps Girault, 1915a: 353; Bouček and Narendran 1981: 239 (synonymised by Bouček and Narendran 1981 with Dirhinus anthracia Walker).
Dirhinus sarcophagae Froggat, 1919: 835; Bouček and Narendran 1981: 239 (synonymised by Bouček and Narendran 1981 with Dirhinus anthracia Walker).
Dirhinus frequens Masi, 1933: 7; Bouček and Narendran 1981: 239 (synonymised by Bouček and Narendran 1981 with Dirhinus anthracia Walker).
Dirhinus intermedius Mani & Dubey, 1972: 404–407; Bouček and Narendran 1981: 239 (synonymised by Bouček and Narendran 1981 with Dirhinus anthracia Walker).
Dirhinus georgei Mani & Dubey, 1974: 31–33; Bouček and Narendran 1981: 239 (synonymised by Bouček and Narendran 1981 with Dirhinus anthracia Walker).
Dirhinus aligarhensis Husain & Agarwal, 1981: 183; Narendran 1989: 294 (examined) (synonymised by Narendran 1989 with Dirhinus anthracia Walker).
Dirhinus ignobilicornis Husain & Agarwal, 1981: 187; Narendran 1989: 294 (examined) (synonymised by Narendran 1989 with Dirhinus anthracia Walker).

##### Material.

1 ♀ (RMNH), “**Vietnam**: Ninh Thuân, Núi Chúa N. P., 90–150 m, Northeast part, 23–30.v.2007, C. v. Achterberg & R. de Vries, RMNH’07”.

##### Diagnosis.

See notes under *Dirhinus auratus*.

##### Hosts.

*Bactrocera cucurbitae* (Coq.) (Diptera: Tephritidae); *Bactrocera dorsalis* (Hendel) (id.); *Calliphora stygia* (F.) (Diptera: Calliphoridae); *Sarcophaga aurifrons* Mac. (Diptera: Sarcophagidae); *Blaesoxipha pachytyli* (Skuse) (id.); *Musca domestica* L. (Diptera: Muscidae); *Exorista bombycis* (Louis) (Diptera: Tachinidae); *Exorista sorbillans* (Wiedemann) (id.); *Ceracia fergusoni* (Malloch) (id.); *Artona catoxantha* Hampson (Lepidoptera: Zygaenidae); *Plecoptera reflexa* G. (Lepidoptera: Noctuidae); *Bombyx mori* (L.) (Lepidoptera: Bombycidae); *Palpita machaeralis* W. (Lepidoptera: Pyralidae) and *Chortoicetes terminifera* (Walker) (Orthoptera: Acrididae).

##### Distribution.

India (Manipur; Punjab; Uttar Pradesh), Vietnam (new record), China (Taiwan), Philippines, South Africa, Zambia, Australia (New South Wales; Queensland); (Bouček and Narendran 1981; Narendran 1989).

#### Dirhinus auratus

Taxon classificationAnimaliaHymenopteraChalcididae

Ashmead, 1905

Figs 75–76 , 77–78

Dirhinus auratus Ashmead, 1905: 402 (♀, lectotype (selected by Bouček & Narendran, 1981), Philippines (Manila) (USNM) (examined)).
Dirhinus pambaeus Mani & Dubey (in Mani et al.), 1974: 33–36 (♂, holotype, India (Kerala) (USNM) (examined) (synonymised by Bouček and Narendran 1981 with Dirhinus auratus Ashmead)).
Dirhinus circinus Husain & Agarwal, 1981: 182. (♀, India (Aligarh) (ZDAMU) (examined) (synonymised with Dirhinus auratus Ashmead by Narendran 1989)).

##### Material.

1 ♀ (RMNH), “**Vietnam**: Ninh Thuân, Núi Chúa N. P., 90–150 m, Northeast part, 23–30.v.2007, C. v. Achterberg & R. de Vries, RMNH’07”; 1 ♀ (IEBR), “S. Vietnam: Dóng Nai, Cát Tiên N. P., Dong trail, Malaise traps, c. 100 m, 1–8.iv.2007, Mai Phu Quy & Nguyen Tanh Manh, RMNH’07”; 1 ♀ (BPBM), “Vietnam, 20 km S of Dalat, 1300 m, 12.ix.1960, Gressitt”; 1 ♀ (BPBM), “Vietnam, Ap Hung Lam, 21 kms N.W. of Dilinh, 1100 m, ix–x.1960, Yoshimoto”.

##### Diagnosis.

This species comes near *Dirhinus anthracia* Walker in the key to species by Bouček and Narendran (1981), but differs from *Dirhinus anthracia* in having: 1) each horn in dorsal view at anterior ocular line slightly narrower than scrobal gap (in *Dirhinus anthracia* each horn in dorsal view at level with anterior eye margin broader than scrobal gap); 2) median areola of propodeum elongate with almost parallel sides (in *Dirhinus anthracia* median aeola of propodeum with convex sides, shortly oval); 3) scutellum without an impunctate strip (in *Dirhinus anthracia* scutellum anteriorly with an impunctate strip); 4) striate area on T1 narrower than long, with few striae and its hind margin produced in middle (in *Dirhinus anthracia* striate area of T1 nearly straight, the area subquadrate or even broader than long) and 5) parascrobal space usually with less than two compete rows of punctures between pre-orbital carina and scrobal margin (in *Dirhinus anthracia* narrow space between eye and lower part of scrobe with pre-orbital carina separated from scrobes by fully 2 rows of punctures).

##### Description.

♀♂, length of body 3.1–4.1 mm.

*Colour*. General body colour black with antenna, tegulae, fore and mid legs (except coxae) mainly reddish.

*Head*. Head below each horn without distinct additional teeth; facial edge of scrobe sinuate, apex of each horn with distinct notch; each horn in dorsal view at anterior ocular line slightly narrower than scrobal gap; parascrobal area in ♀ hardly one-third as broad as scrobal cavity, with only one compete row of punctures between pre-orbital carina and scrobal edge; head in lateral view about 0.7 × wider than high (Fig. 75); genal length subequal to short diameter of eye; pedicel slightly longer than second flagellar segment; clava nearly twice as long as wide.

*Mesosoma*. Mesosoma not flattened; scutellum extensively punctate, without impunctate strip; median areola of propodeum elongate with almost parallel sides.

*Wings*. Fore wing pilosity usually distinct.

*Legs*. Hind tibia without distinct external additional carina.

*Metasoma*. Petiole of ♀ with an area of four carinae about 1.5 × as broad as long, with fewer striae apically than in middle.

##### Host.

*Dacus* sp. (Diptera: Tephritidae).

##### Distribution.

Vietnam, India, Pakistan, Sri Lanka, Thailand, Laos, China (Taiwan) and Philippines (Bouček and Narendran 1981; Narendran 1989).

#### Dirhinus claviger

Taxon classificationAnimaliaHymenopteraChalcididae

Bouček & Narendran, 1981

Figs 79–80 , 81–82

Dirhinus claviger Bouček & Narendran, 1981: 237 (♀, holotype, India (BMNH)); Narendran 1989: 290, 293 (keyed)).
Dirhinus clavatus Hussain & Agarwal, 1981: 185 (♀, holotype, Aligarh (ZDAMU) (synonymised with Dirhinus claviger Bouček & Narendran by Narendran 1989)).

##### Material.

2 ♀ (RMNH, IEBR), “**Vietnam**: Ninh Thuân, Núi Chúa N. P., dry South part, 22–29.v.2007, C. v. Achterberg & R. de Vries, RMNH’07”.

##### Diagnosis.

This is a unique species with the following combination of characteristics: clavate antenna, an extra outer carina near hind tibial sulcus, very reduced pilosity and striate area of T1 0.40–0.45 × length of T1.

##### Description

(female from Vietnam). ♀, length of body 3.7 mm.

*Colour*. Black with following parts as follows: scape pale yellowish brown; pedicel and funicular segments pale yellowish brown with a black tinge; clava dark brown; eyes gray with pale reflecting yellowish spots; ocelli reflecting pale whitish yellow; fore and mid legs (except black coxae) pale brownish yellow; hind leg black except pale yellow tarsi; telotarsi brown; wings hyaline, veins brown.

*Head*. In dorsal view sides of head moderately converging behind eyes; eyes longer than temples (9:5); ocellar area distinctly elevated; POL a little shorter than OOL (7:8); AOL twice OOL; each horn with a notch present outside of apex; each horn in basal third 1.5 × as broad as scrobal gap, latter 0.7 × breadth of pre-claval segment of antenna; pre-orbital carina present; facial edge of scrobes weakly sinuate in lateral view; antenna short, strongly clavate, clava slightly longer than broad (8:7), with a large area of micro-pilosity on one side; all flagellar segments transverse, pre-claval segment nearly 3 × as broad as long; pedicel as long as flagellar segments 2 and 3.

*Mesosoma*. Mesosoma hardly depressed; its pits dense but not crowded, leaving broad smooth areas on scapulae and on median part of scutellum; middle lobe of mesoscutum with pits close to anterior admarginal area without punctures, smooth, interstices smooth and carinate. Pronotum in median line very slightly depressed, sides hardly converging forward; scutellum slightly broader than long (11:9), apex curved and rounded. Propodeum with hind corners 1.3 × nearer to each other than to metanotal margin; median areola nearly round, slightly longer than broad. Adcoxal lateral tooth of metapleuron a little less than 90°.

*Wings*. Fore wing 3.4 × as long as broad, pilosity mostly reduced, a little more numerous near apical margin; PMV subequal in length to STV.

*Legs*. Hind femur 1.3 × as long as broad, with interstices of setigerous pits shorter to a little longer than width of 3^rd^ hind tarsal segment; basal tooth of comb slightly more prominent than other teeth. Hind tibia with well developed external carina, extending over half length towards knee, furrow between this carina and tarsal sulcus flat.

*Metasoma*. Metasoma as long as mesosoma; petiole twice as broad as its length, with 2 pairs of strong raised longitudinal carinae enclosing foveola; anterior margin strongly emarginated in middle; striate area of T1 with 14–16 strong longitudinal striae, their length 0.4 × length of T1, hind margin of striate area almost straight; hind half of T1 shiny, mostly smooth, but with a cross row of microscopic pits before apex, pits faint in middle part of cross row but a little more dense on sides.

*Male*. So far not recorded from Vietnam.

##### Host.

Unknown.

##### Distribution.

Vietnam (new record), India, Sri Lanka, (Bouček and Narendran 1981; Narendran 1989).

##### Variation.

Length of body 3.5–3.7 mm. Each frontal horn in basal third 1.5–1.7 × as broad as scrobal gap; propodeum with hind corners about 1.2–1.3 × nearer to each other than to metanotal margin; petiole 1.7–2.0 × as long as broad; striate area of T1 almost straight to slightly arcuate at apex; length of striate area of T1 0.40–0.45 × length of T1.

#### Dirhinus neoclaviger sp. n.

Taxon classificationAnimaliaHymenopteraChalcididae

http://zoobank.org/D6AFD476-5275-4FCE-962D-328B1ED69B48

Figs 83 , 84–85

##### Type material.

Holotype, ♀ (RMNH), “**Vietnam**: Ninh Thuân, Núi Chúa N. P., 90–150 m, Northeast part, 23–30.v.2007, C. v. Achterberg & R. de Vries, RMNH’07”.

##### Diagnosis.

This new species resembles *Dirhinus claviger* Bouček & Narendran by the shape of the antenna, but differs from it in having: 1) anterior margin of frontal horns transverse and not notched (in *Dirhinus claviger* anterior margin of each frontal horn notched and not transverse); 2) frontal horns relatively shorter than any of the Oriental species of *Dirhinus*; 3) mesoscutum and scutellum densely punctate without any broad smooth areas (in *Dirhinus claviger* with broad smooth areas present on scapulae and scutellum and punctures sparse in middle of mesoscutum); 4) distance between setigerous punctures on outer surface of hind femur not greater than breadth of third hind tarsal segment (in *Dirhinus claviger* distance between punctures on hind femur slightly greater than breadth of third hind tarsal segment); 5) striate area of T1 very much reduced in between outermost carinae (in *Dirhinus claviger* striate area of T1 not so reduced between outermost carinae but quadrate) and 6) apex of scutellum with a tooth-like protuberance (in *Dirhinus claviger* no such protuberance present at apex of scutellum).

This new species resembles *Dirhinus himalayanus* Westwood in having frontal horns without notch at apex, but differs from it in having frontal horns with transverse anterior margin (in *Dirhinus himalayanus* frontal horns rounded at anterior margin); 2) frontal horns relatively short (in *Dirhinus himalayanus* frontal horns longer); 3) mesoscutum and scutellum without impunctate broad area (in *Dirhinus himalayanus* mesoscutum and scutellum sparsely punctate); 4) antenna with micro-pilosity on clava (in *Dirhinus himalayanus* antennal clava without micro-pilosity), 5) T1 without strong basal carinae (in *Dirhinus himalayanus* T1 with strong basal carinae) and 6) apex of scutellum with a tooth-like protuberance (in *Dirhinus himalayanus* apex of scutellum rounded without protuberance).

##### Description.

Holotype, ♀, length of body 4.1 mm.

*Colour*. General body colour black; scape, pedicel and anellus pale brownish yellow; F1 pale brown; remaining segments black; eye and ocelli pale reflecting yellow; trochanters, femora and tibiae of fore and mid legs pale brownish yellow; fore tarsus pale brownish yellow; all coxae, hind trochanter, hind femur and hind tibia black; tegula pale yellow; wings hyaline; veins brown.

*Head*. Head in dorsal view with sides distinctly though moderately converging behind eyes; eyes clearly longer than temples; ocellar area distinctly elevated; each horn extremely small, its length from hind ocellus 2.8 × width of anterior margin of horn; anterior margin of each horn transversely margined, notch or teeth absent; each horn as broad as scrobal gap at its basal third; pre-orbital carina noticeable but blunt; facial edge of scrobes in lateral view only weakly sinuate. Antenna short strongly clavate; clava slightly longer than broad and on one side (facing anteriorly when antenna folded into scrobe) with large, round flat area of micro-pilosity; all flagellar segments transverse, F1 very slightly so; width of base of clava a little over 4 × length of basal segment of clava; pedicel about as long as F2 and F3 combined.

*Mesosoma*. Mesosoma not depressed, with close, setigerous punctate, interstices narrower than diameter of a puncture, rugulose or faintly reticulate; no smooth area on scapula and scutellum; pronotum in median line not depressed, sides hardly converging forward; scutellum including apical tooth-like protuberance very slightly longer than broad (12:10); relative length of tooth-like protuberance 0.2 × width of scutellum; propodeum with median areola nearly round, as long as broad; adcoxal lateral tooth of metapleuron slightly less than 90°.

*Wings*. Wing pubescence denser on distal half than on proximal half, setae relatively short; PMV absent.

*Legs*. Hind femur 1.4 × as long as broad, outer disc with dense setigerous shallow micro-pits, basal tooth of comb of teeth prominent; hind tibia with weak external carina extending over more than half length towards knee, area between this carina and tarsal sulcus flat, narrower than that of *Dirhinus himalayanus*, partly blurred by setigerous punctures.

*Metasoma*. Petiole a little wider than long (11:8), slightly diverging posteriorly, anterior margin not emarginated in middle area, with 5 carinae, area of carinae as long as broad; striate area of T1 with irregular length and very short carinae basally except the outermost one on either side (which are shorter than the width between them (3:5)), carinae not subquadrate; median short carinae 0.1 × length of T1; length of outermost lateral carinae 0.3 × length of T1; hind margin of T1 slightly arcuate; T1 shiny, mostly smooth with micro-sculptured on either side before apex.

*Male*. Unknown.

##### Host.

Unknown.

##### Etymology.

Named after *Dirhinus claviger* Bouček & Narendran because the shape of the antenna is similar.

#### Dirhinus secundarius

Taxon classificationAnimaliaHymenopteraChalcididae

Masi, 1933

Dirhinus secundarius Masi, 1933: 10–11 (♀, Taiwan, (syntypes) (MNHN)); Habu 1960: 131–363 (redescription); Narendran 1989: 290, 301 (keyed).

##### Description

(based on Habu 1960). ♀.

*Colour*. Black with following parts as follows: tegulae reddish brown; fore and mid trochanters brown; fore and mid femora reddish black with base and apex brown; fore and mid tibiae brown with basal half slightly reddish; tarsi pale brown; claws and arolium somewhat dark; wings hyaline with somewhat brownish tinge; veins pale brown or dark reddish. Pubescence on body silvery (in some specimen golden dorsally).

*Head*. Head with horns slightly diverging towards apex, apex with a distinct notch; horns a little wider than space between them; a little more than one-third as long as head; twice as long as wide. Antenna inserted at level of ventral margin of eyes; anterior tentorial pits distinctly delimited by longitudinal carina at outer margin, not distinctly smooth; height of malar space 0.6 × major axis of compound eye; antenna with scape longer than combined length of F1 to F3, 2.1 × as long as clava; F1 a little longer than F2 (12:10); clava about 2.6 × as long as F7.

*Mesosoma*. Mesosoma with scutellum a little longer than wide, with close pits, interstices carinate; median areola of propodeum subparallel-sided, distinctly longer than wide, with fine secondary carinae; depression posterior to median areola long, lateral costae with two teeth; anterior tooth behind spiracle distinct, posterior tooth small.

*Wings*. Fore wing with MV 1.2 × as long as SMV.

*Metasoma*. Petiole slightly longer than wide (12:10) with 4 longitudinal carinae; metasoma a little longer than combined length of pronotum, mesoscutum and scutellum; T1 almost reaching middle of metasoma, with 8 long and six short carinae at base, with posterior margin not straight but produced posteriorly in middle.

##### Host.

Unknown.

##### Distribution.

China (Taiwan), Japan.

##### Remarks.

This species is not known from Vietnam, but included here because it may occur in North Vietnam.

#### Epitranus

Taxon classificationAnimaliaHymenopteraChalcididae

Walker, 1834

Figs 86 , 87–88 , 89–90 , 91–93 , 94 , 95–96 , 97–99 , 100–102 , 103 , 104 , 105–106 , 107–109 , 110–112 , 113–114 , 115–116 , 117–119 , 120–121 , 122–124 , 125 , 126–128 , 129–131

Epitranus Walker, 1834: 21, 26. Type species: Epitranus fulvescens Walker, by monotypy.
Chalcitella Westwood, 1835: 70. Type species: Chalcitella evanioides Westwood, by monotypy. Synonymized with Epitranus by Burks (1936).
Anacryptus Kirby, 1883: 56. Type species: Epitranus impulsator Walker, by monotypy. Synonymized with Epitranus by Burks (1936).
Arretocera Kirby, 1883: 56. Type species: Epitranus albipennis Walker, by monotypy. Synonymized with Epitranus by Burks (1936).
Neoanacryptus Girault, 1913b: 86. Type species: Neoanacryptus petiolatus Girault, by original designation. Synonymized with Epitranus by Masi (1940).
Chalcitelloides Girault, 1914: 30. Type species: Chalcitelloides nigriscutum Girault, by original designation and monotypy. Synonymized with Epitranus by Burks (1936).
Paranacryptus Girault, 1915a: 349. Type species: Paranacryptus sanguineus Girault, by original designation. Synonymized with Epitranus by Masi (1940).
Pararetoceroides Mani, 1938: 149. Type species: Arretoceroides ceylonensis Mani, by monotypy. Synonymized with Epitranus by Habu (1960).
Lamoundella Shafee & Dutt, 1986: 83. Type species: Lamoundella aligarhensis Shafee & Dutt, by original designation and monotypy. Synonymized with Epitranus by Narendran and Padmasenan (1986).

##### Diagnosis.

Belongs to the group of genera with a petiolate first metasomal segment and is easy recognizable because of the absence of cephalic horns and the very low antennal toruli.

##### Description.

Antennal toruli located very low on a clypeal shield protruding over oral region; scrobe virtually absent; MV very long, STV rudimentary; PMV absent. Hind femur with a comb of teeth or an irregular row of teeth on outer ventral margin; metasoma with slender, striate petiole; metasoma bulging ventrally.

##### Hosts.

Lepidoptera (Pyralidae and Tineidae).

##### Distribution.

Old World tropics and doubtfully New World (Bouček 1982).

##### Key to Vietnamese species of *Epitranus* Walker

**Table:** 

1	Dorsal face of propodeum at most with a short areola, posteriorly connected to median carina and narrowed posteriorly or only a median carina connected to curved posterior lamella; [scutellum flat and near level of propodeum; mesosoma black]	**2**
–	Dorsal face of propodeum with long areola, posteriorly connected to curved posterior lamella and narrowed anteriorly	**3**
2	Epipygium (= tergite above ovipositor sheath) of ♀ distinctly longer than wide and metasoma distinctly acute apically (Fig. 122); dorsal tarsal groove of hind tibia confined to distal third of tibia; [interspaces between punctures on mesoscutum narrower than punctures]	***Epitranus oxytelus* Walker**
–	Epipygium (= tergite above ovipositor sheath) of ♀ about as long as wide and metasoma nearly obtuse apically; dorsal tarsal groove of hind tibia confined to distal two-fifths of tibia	***Epitranus parvidens* (Strand)**
3	Anterior half of fore wing yellowish (Fig. 91); dorsal tarsal groove of hind tibia up to subbasal tooth of tibia; posterior half of hind femur with widely spaced teeth (Fig. 90); [malar space reddish brown; head in anterior view rather elongate (Fig. 94); basal half of T1 reddish brown]	***Epitranus ater* Bouček**
–	Anterior half of fore wing hyaline; dorsal tarsal groove of hind tibia remain distinctly removed from subbasal tooth of tibia; **if** nearly reaching subbasal tooth then posterior half of hind femur with narrowly spaced teeth (Fig. 96)	**4**
4	Hind femur finely regularly serrate (Fig. 86), at least with 20 small teeth; head mainly reddish brown; hind tibia with medium-sized subbasal tooth (fig. 86); frons with fine punctures (Fig. 87)	***Epitranus albipennis* Walker**
–	Hind femur coarser and irregularly serrate or dentate (Figs 109, 119, 127), at most with 13 small teeth; head black; hind tibia with minute subbasal tooth (Fig. 127); frons with coarse punctures (Fig. 129)	**5**
5	Area below marginal vein of fore wing glabrous and without or an indistinct “hairline” (= curved line of setae connected to stigma vein)	**6**
–	Area below marginal vein of fore wing partly setose and with a distinct “hairline”	**9**
6	Hind coxa slender basally, subparallel-sided and about as long as hind femur (Fig. 108); third antennal segment of ♀ elongate (Fig. 110); [outer side of hind coxa yellowish brown basally]	***Epitranus narendrani* sp. n.**
–	Hind coxa widened subbasally and distinctly shorter than hind femur (Figs 119, 127); third antennal segment of ♀ robust (Fig. 129)	**7**
7	T1 slender in lateral and dorsal view (Figs 113, 116) and its longitudinal costulae becoming obsolescent apically (Fig. 116); hind coxa and femur mainly fuzzy dark brown (Fig. 114)	***Epitranus neonigriceps* sp. n.**
–	T1 rather robust in lateral and dorsal view (Figs 117, 118, 126, 130) and its longitudinal costulae distinct apically (Fig. 130); hind coxa and femur partly or entirely reddish or yellowish brown (Figs 119, 127)	**8**
8	Mesosoma black (Fig. 117); fore wing without any indication of a “hair line”; hind coxa and femur entirely reddish brown (Fig. 119); [interspaces between punctures on mesoscutum about as wide as punctures]	***Epitranus nigriceps* Bouček**
–	Mesosoma with reddish-brown pattern (Fig. 126); fore wing with an indistinct “hairline” (= curved line of few setae connected to stigmal vein); hind coxa and femur partly black (Fig. 127)	***Epitranus ramnathi* (Mani & Dubey)**
9	Malar space in anterior view with dense and rather long setosity (Fig. 103); head of ♀ in anterior view distinctly triangular (Fig. 103); [hind femur basally and apically reddish brown; mesosoma black]	***Epitranus erythrogaster* Cameron**
–	Malar space in anterior view with sparse and short setosity (Fig. 97); head of ♀ in anterior view more transverse (Fig. 97)	**10**
10	Dorsal tarsal groove of hind tibia surpassing middle of tibia (Fig. 99); frons with fairly fine sculpture (Fig. 97); pronotum and mesoscutum partly reddish brown with scutellum black; hind femur partly yellowish brown (Fig. 96)	***Epitranus crassicornis* Bouček**
–	Dorsal tarsal groove of hind tibia 0.3–0.4 times length of tibia, not surpassing middle of tibia (Fig. 104); frons with very coarse rugulose sculpture; mesosoma entirely black; hind femur entirely dark brown (Fig. 104)	***Epitranus gauldi* Bouček**

**Figure 86.**
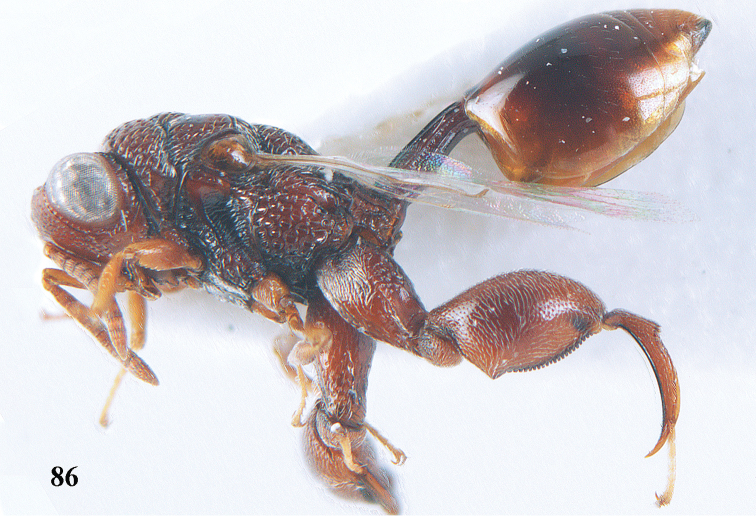
*Epitranus albipennis* Walker, ♀, Vietnam, Núi Chúa N. P., habitus lateral.

**Figures 87–88.**
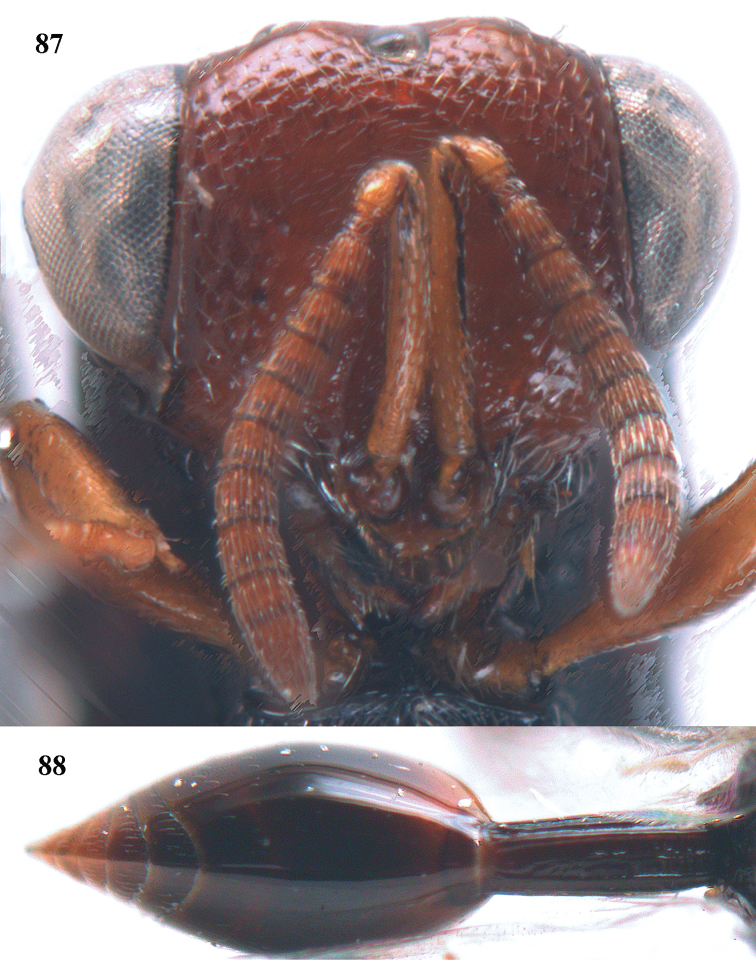
*Epitranus albipennis* Walker, ♀, Vietnam, Núi Chúa N. P. **87** head anterior **88** metasoma dorsal.

**Figures 89–90.**
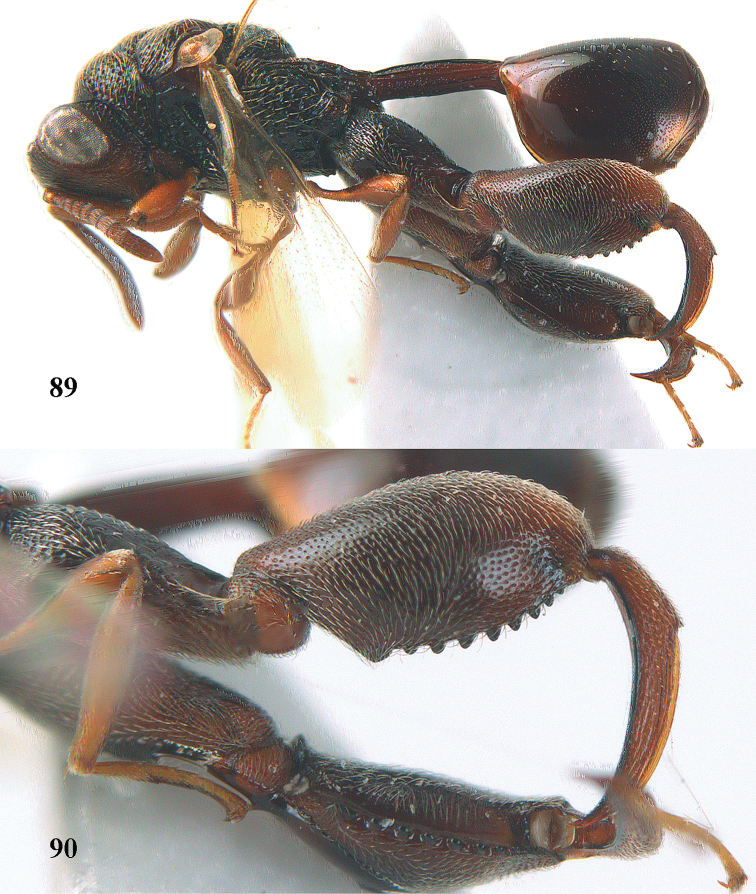
*Epitranus ater* Bouček, ♀, Vietnam, Chu Yang Sin N. P. **89** habitus lateral **90** hind leg lateral.

**Figures 91–93.**
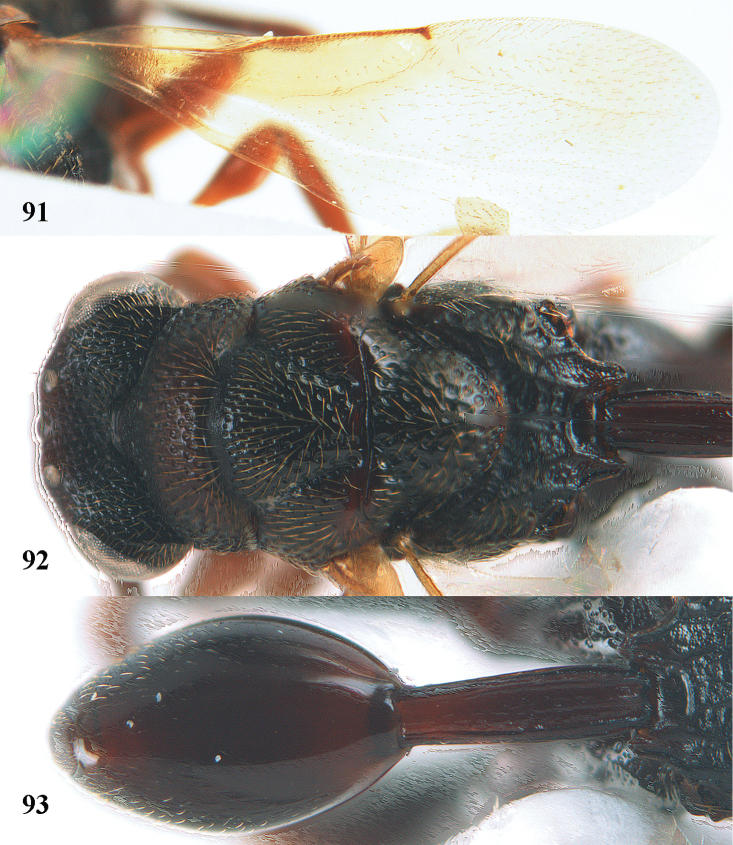
*Epitranus ater* Bouček, ♀, Vietnam, Chu Yang Sin N. P. **91** fore wing **92** mesosoma dorsal **93** metasoma dorsal.

**Figure 94.**
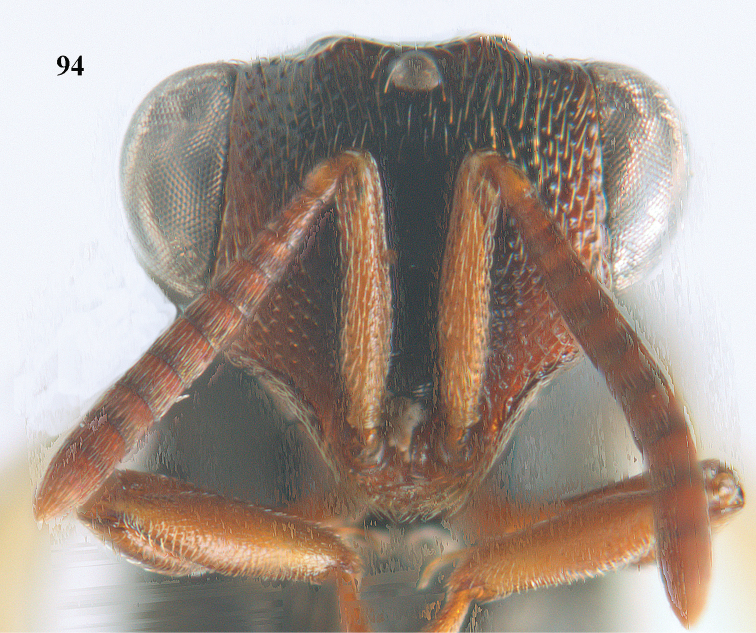
*Epitranus ater* Bouček, ♀, Vietnam, Chu Yang Sin N. P., head anterior.

**Figures 95–96.**
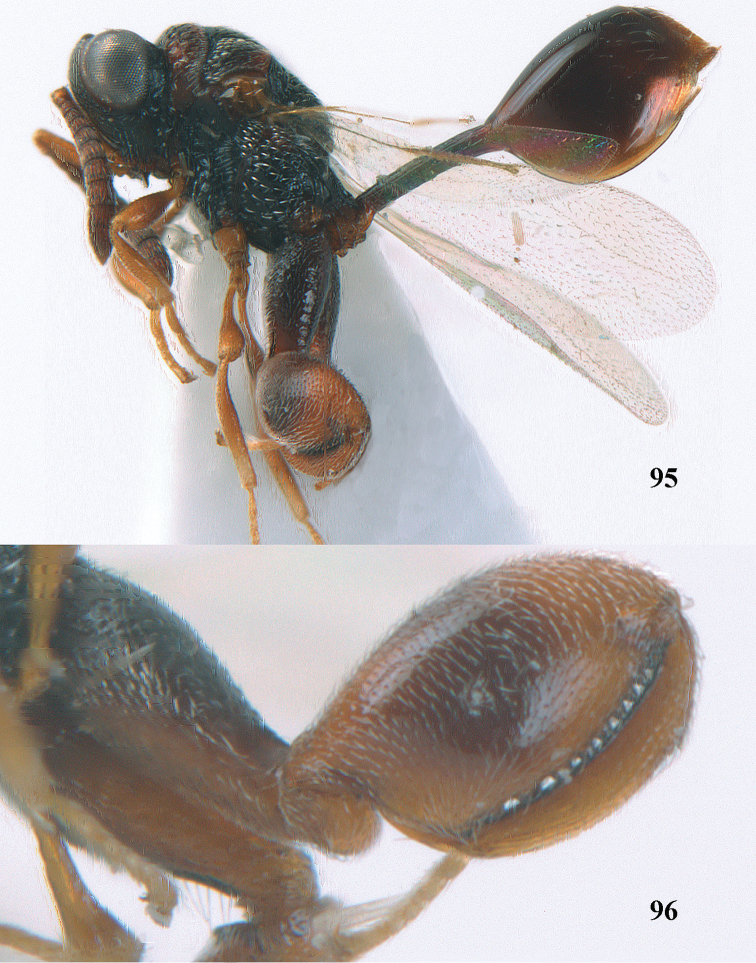
*Epitranus crassicornis* Bouček, ♀, Vietnam, Cát Tiên N. P. **95** habitus lateral **96** hind leg lateral.

**Figures 97–99.**
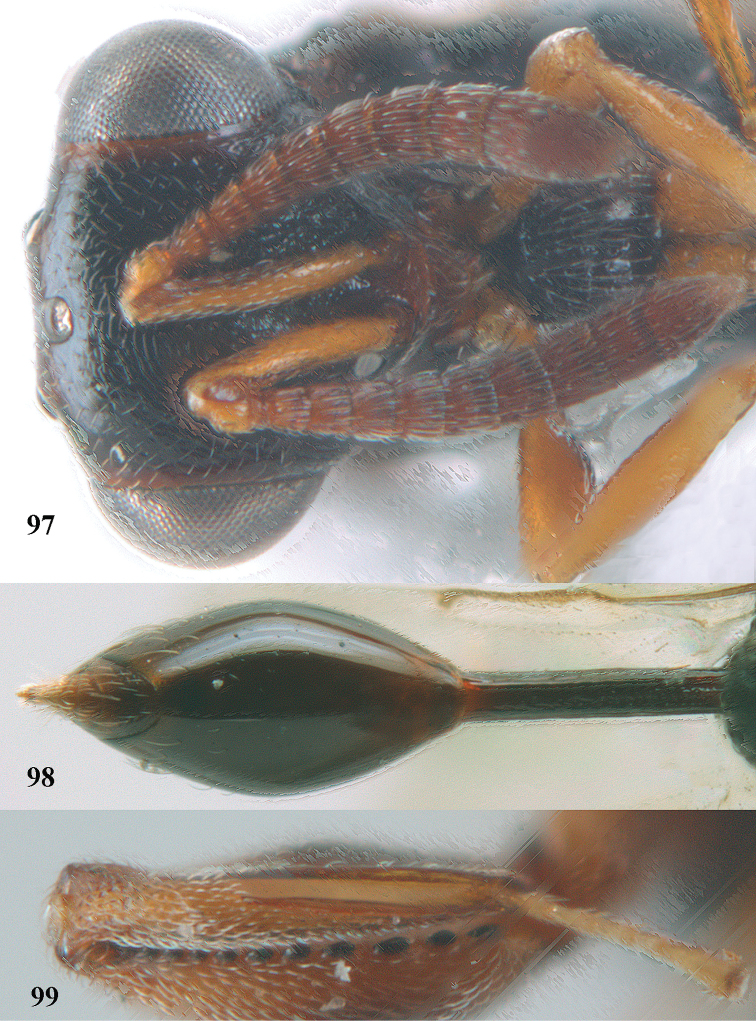
*Epitranus crassicornis* Bouček, ♀, Vietnam, Cát Tiên N. P. **97** head, anterior **98** metasoma dorsal **99** hind tibia dorsal.

**Figures 100–102.**
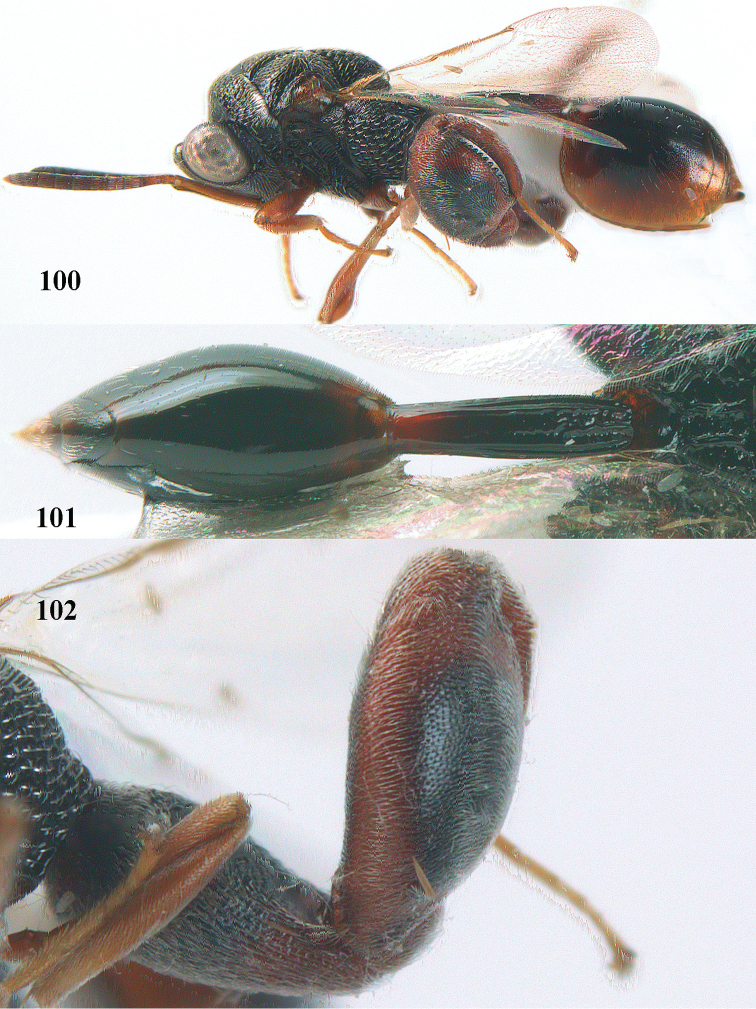
*Epitranus erythrogaster* Cameron, ♀, Vietnam, Cát Tiên N. P. **100** habitus lateral **101** metasom dorsal **102** hind leg lateral.

**Figure 103.**
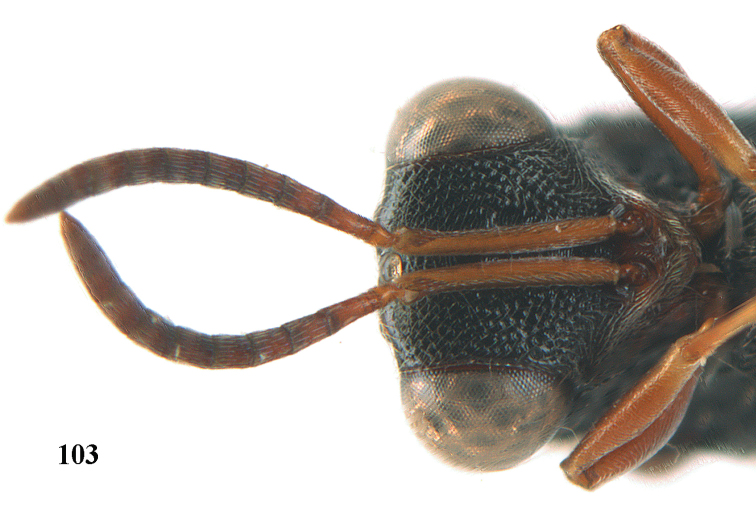
*Epitranus erythrogaster* Cameron, ♀, Vietnam, Cát Tiên N. P., head anterior.

**Figure 104.**
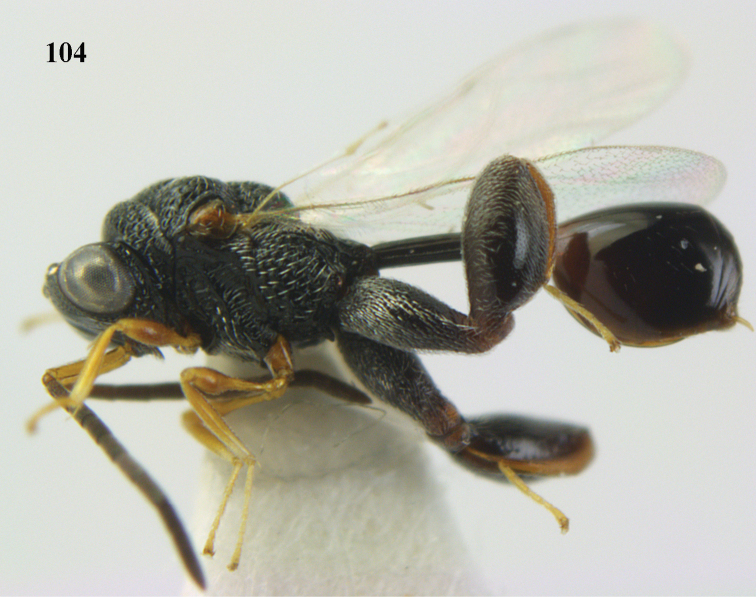
*Epitranus gauldi* Bouček, ♂, Vietnam, Phong Dién N. R., habitus lateral.

**Figures 105–106.**
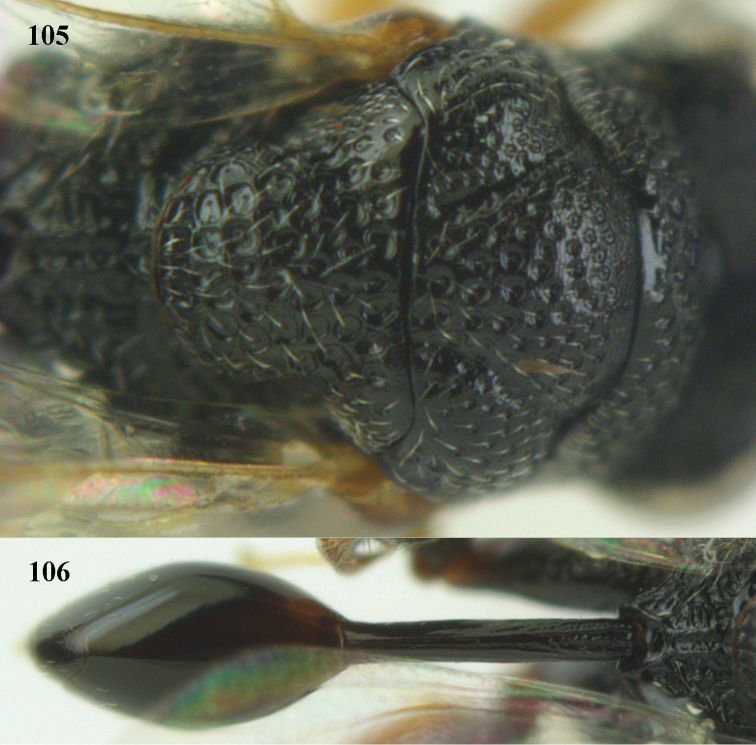
*Epitranus gauldi* Bouček, ♂, Vietnam, Phong Dien N. R. **105** mesonotum dorsal **106** metasoma dorsal.

**Figures 107–109.**
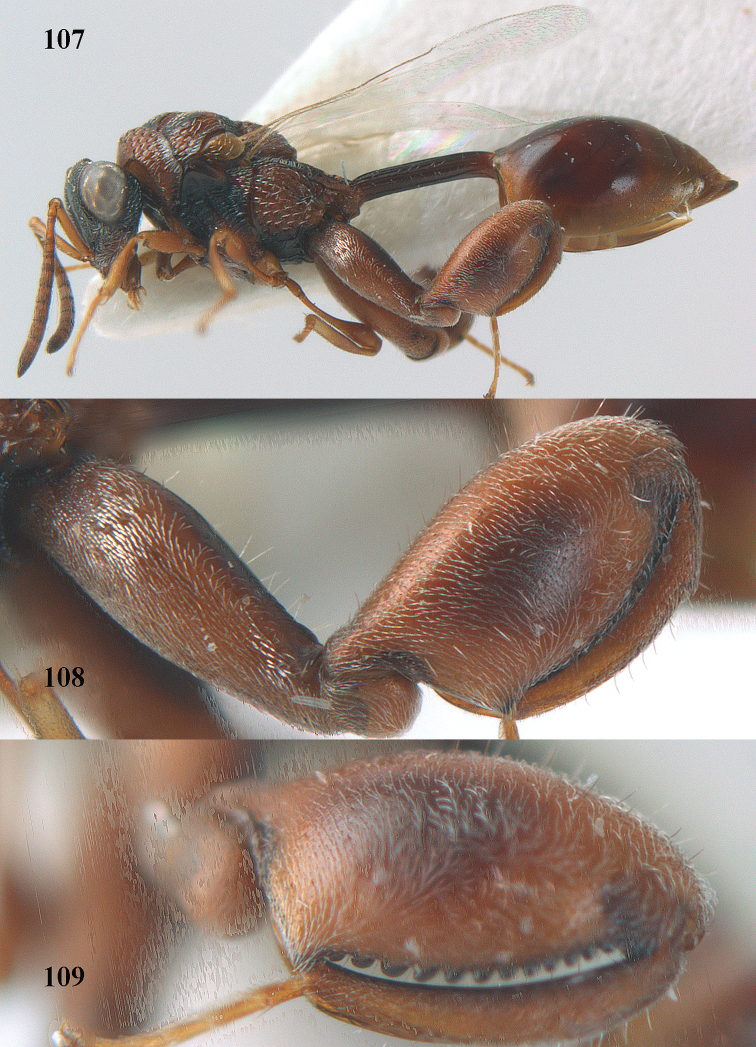
*Epitranus narendrani* sp. n., ♀, holotype. **107** habitus lateral **108** hind leg lateral **109** hind femur lateral.

**Figures 110–112.**
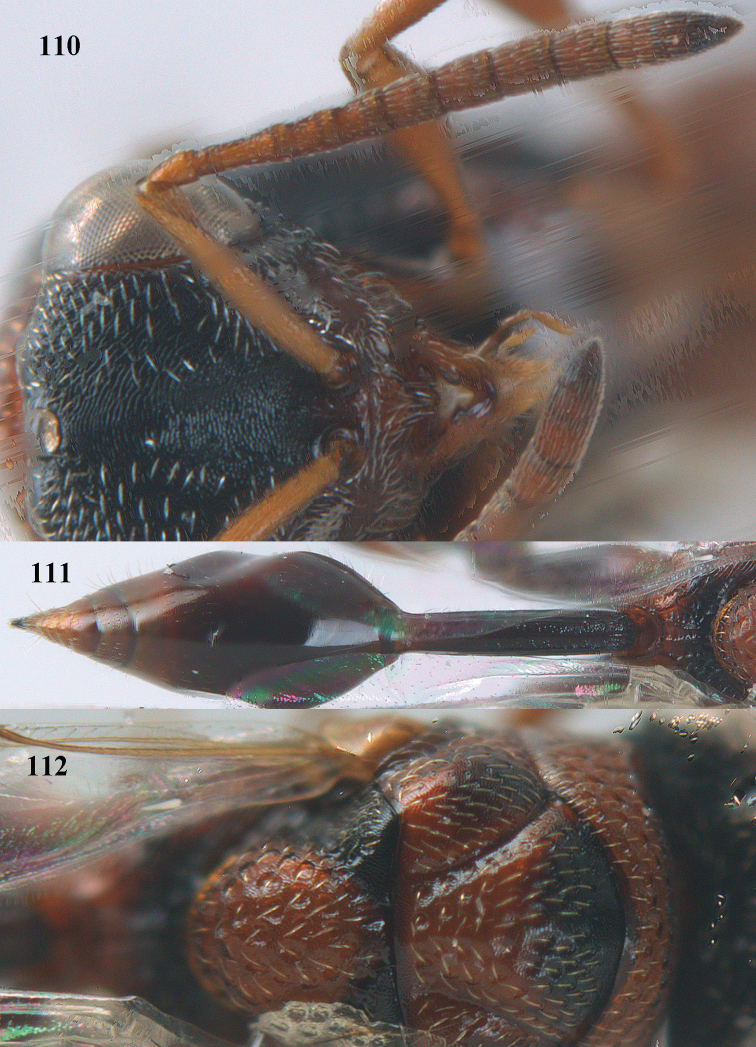
*Epitranus narendrani* sp. n., ♀, holotype. **110** antenna anterior **111** metasoma dorsal **112** mesosoma dorsal.

**Figures 113–114.**
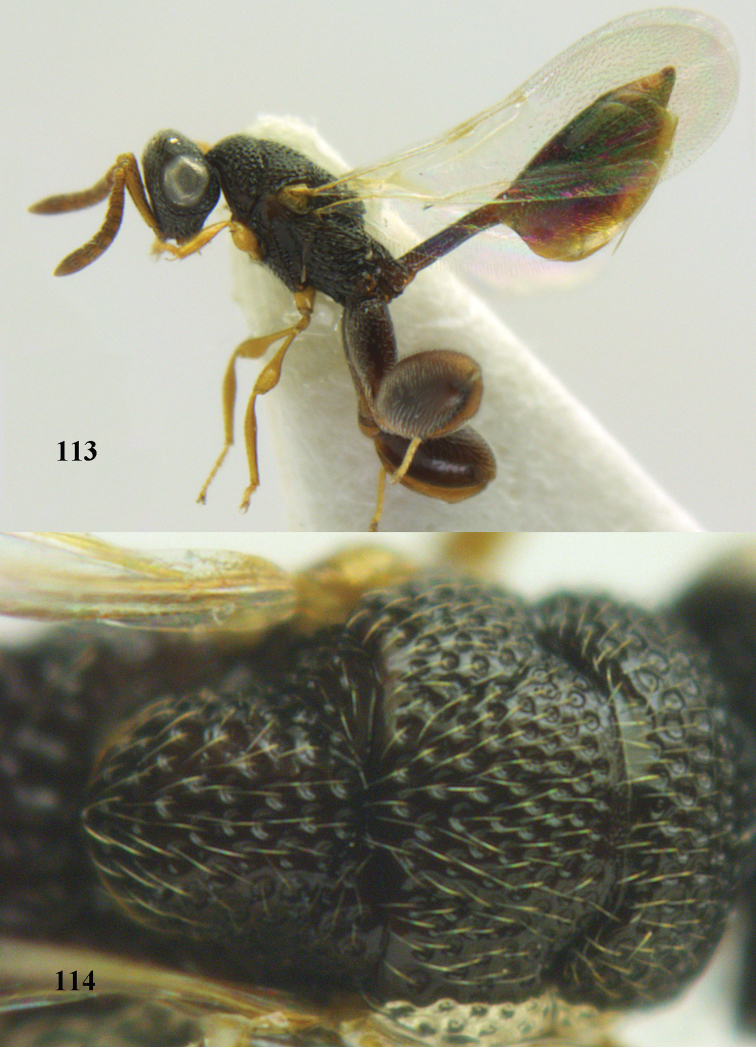
*Epitranus neonigriceps* sp. n., ♀, holotype. **113** habitus lateral **114** mesonotum dorsal.

**Figures 115–116.**
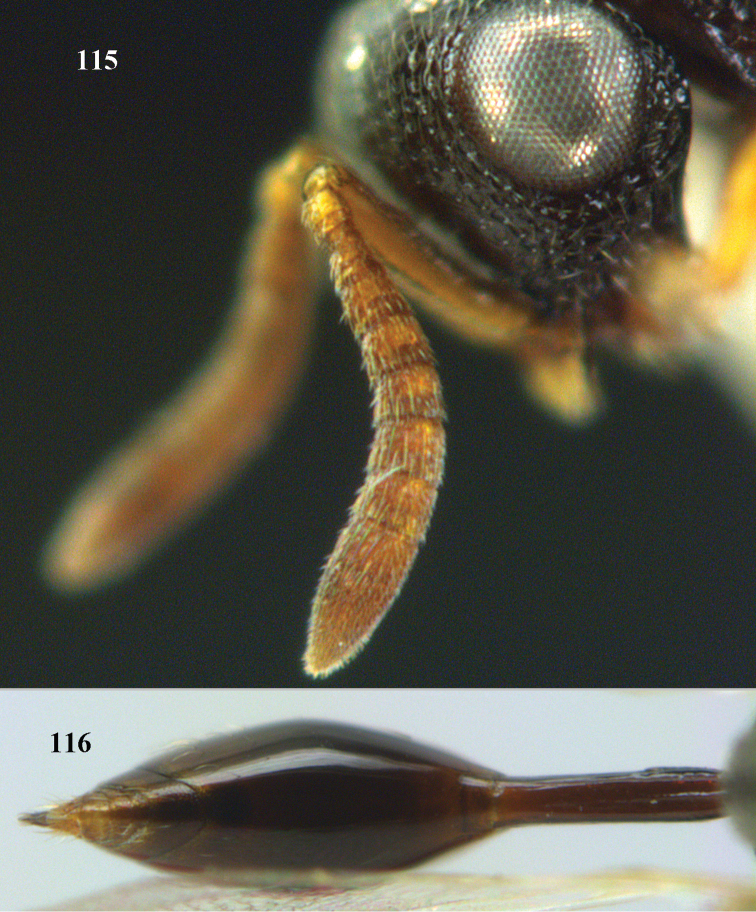
*Epitranus neonigriceps* sp. n., ♀, holotype. **115** antenna lateral **116** metasoma dorsal.

**Figures 117–119.**
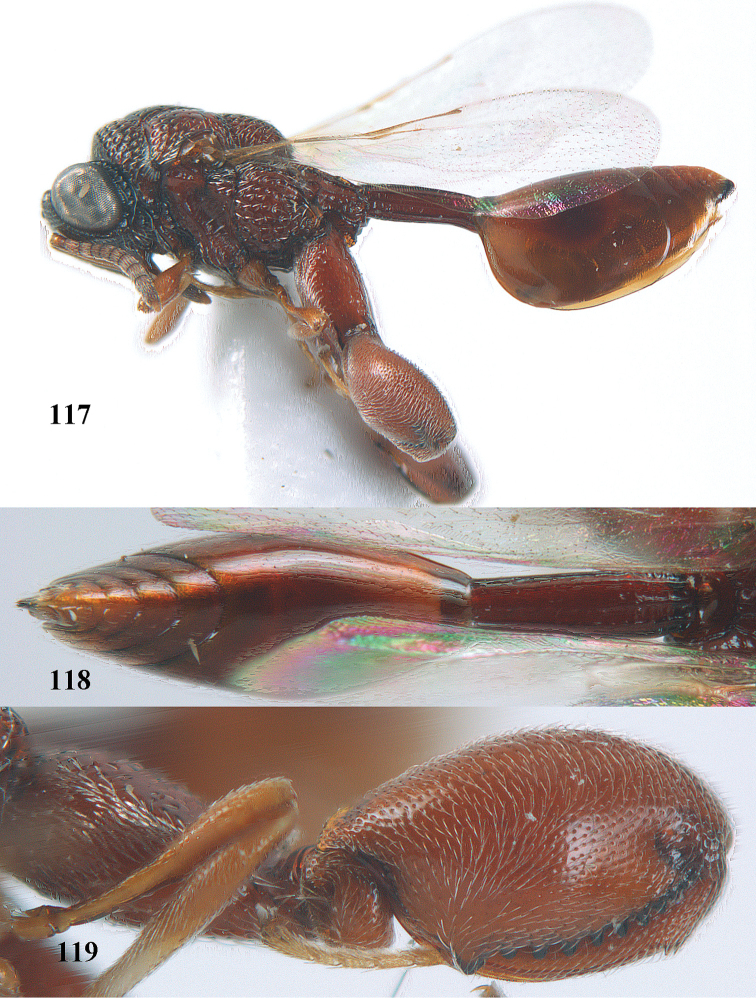
*Epitranus nigriceps* Bouček, ♀¸ Cát Tiên N. P. **117** habitus lateral **118** metasoma dorsal **119** hind leg lateral.

**Figures 120–121.**
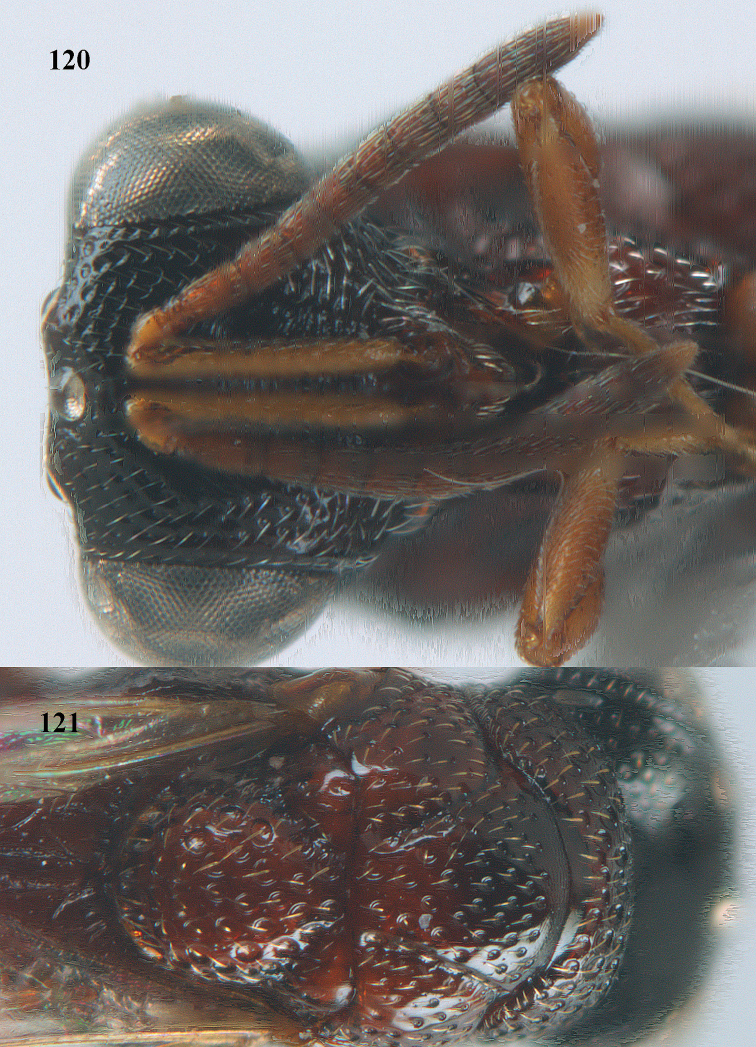
*Epitranus nigriceps* Bouček, ♀¸ Cát Tiên N. P. **120** head anterior **121** mesosoma dorsal.

**Figures 122–124.**
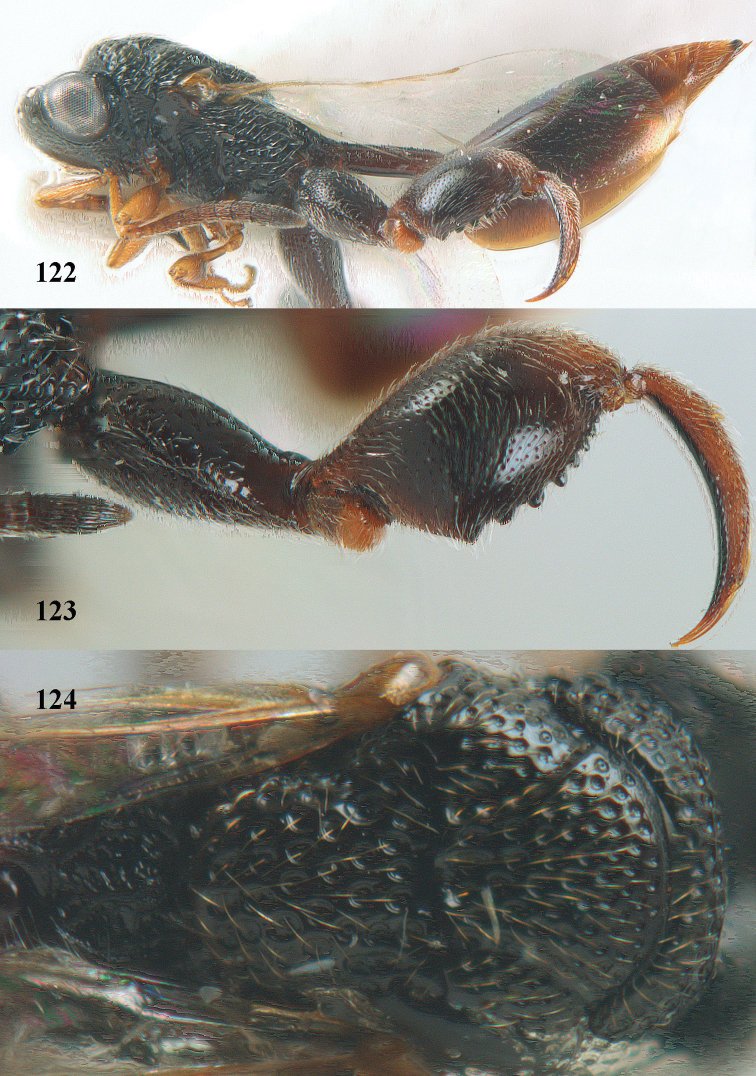
*Epitranus oxytelus* Bouček, ♀¸ Chu Yang Sin N. P. **122** habitus lateral **123** hind leg lateral **124** mesosoma dorsal.

**Figure 125.**
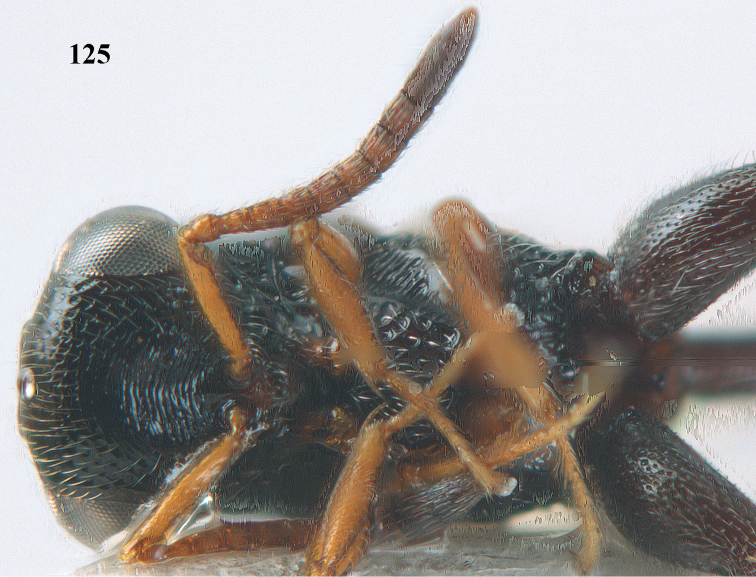
*Epitranus oxytelus* Bouček, ♀¸ Chu Yang Sin N. P., head anterior.

**Figures 126–128.**
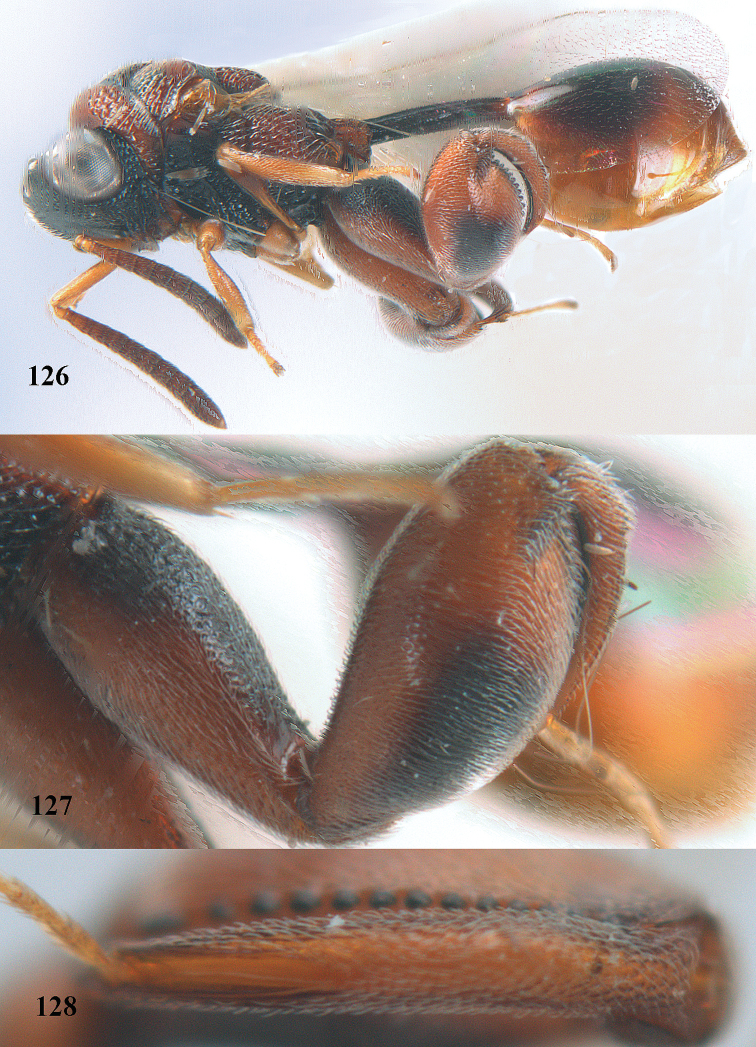
*Epitranus ramnathi* Mani & Dubey, ♀¸ Thuong Cuu. **126** habitus lateral **127** hind leg lateral **128** hind tibia dorsal.

**Figures 129–131.**
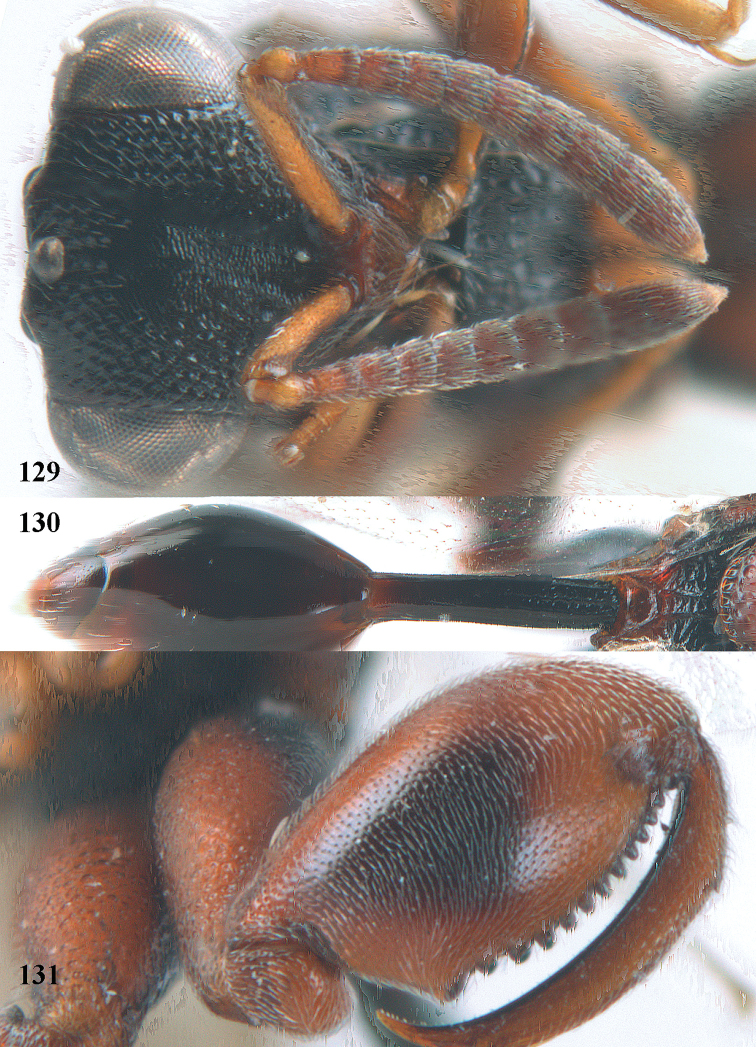
*Epitranus ramnathi* Mani & Dubey, ♀¸ Thuong Cuu. **129** head anterior **130** metasoma dorsal **131** hind femur lateral.

#### Epitranus albipennis

Taxon classificationAnimaliaHymenopteraChalcididae

Walker, 1874

Figs 86 , 87–88

Epitranus albipennis Walker, 1874: 400 (♀, holotype, Japan (BMNH)).
Anacryptus japonicus Ashmead, 1904: 147 (♂, holotype, Japan, (lost) (synonymized with Epitranus albipennis Walker by Habu 1960)).
Anacryptus koebelei Ashmead, 1904: 148 (♀, holotype, Japan, (USNM) (synonymized with Epitranus albipennis Walker by Habu 1960)).
Anacryptus clavipes Cameron, 1911: 19 (♀, lectotype, E. Malaysia (Sarawak) (BMNH) (synonymized with Epitranus albipennis Walker by Bouček 1982)).
Anacryptus rufinus Masi, 193: 11 (♂, lectotype, China (Taiwan) (SDEI) (synonymized with Epitranus albipennis Walker by Bouček 1982)).
?Anacryptus sontakayi Mani & Kurian, 1953: 4 (♂, holotype, India (?)).
Anacryptus marattensis Mani & Dubey, 1973: 31–33 (♀, holotype, India (USNM) (synonymized with Epitranus albipennis Walker by Bouček 1982)).
Epitranus perticellus Hussain & Agarwal, 1982c: 419–421 (♀, holotype, India (ZDAMU) (synonymized with Epitranus albipennis Walker by Narendran 1989)).
Epitranus kashmiriensis Hussain & Agarwal, 1982c: 421–423 (♀, holotype, India (ZDAMU) (synonymized with Epitranus albipennis Walker by Narendran 1989)).

##### Material.

1 ♀, “S. **Vietnam**: Ninh Thuân, Núi Chúa N. P., northeast part, 90–150 m, 23–30.v.2007, Malaise trap, C. v. Achterberg & R. de Vries, RMNH’07”.

##### Distribution.

India; Indonesia (Sumatra); Japan; Malaysia (West Malaysia); China (Guangdong; Taiwan); Vietnam (new record).

#### Epitranus ater

Taxon classificationAnimaliaHymenopteraChalcididae

Bouček, 1982

Figs 89–90 , 91–93 , 94

Epitranus ater Bouček, 1982: 588 (♂, holotype, Laos (BPBM)).

##### Material.

1 ♀ (RMNH), “S. **Vietnam**: Dak Lak, Chu Yang Sin N. P., Krong K’Mar, 760–770 m, 21–26.v.2005, Mal[aise] traps 7–12, C. v. Achterberg & R. de Vries, RMNH’05”; 1 ♀ (IEBR), “S. Vietnam: Ninh Thuân, Núi Chúa N. P., northeast part, 90–150 m, 23–30.v.2007, Malaise trap, C. v. Achterberg & R. de Vries, RMNH’07”.

##### Distribution.

Laos; Vietnam (new record).

#### Epitranus crassicornis

Taxon classificationAnimaliaHymenopteraChalcididae

Bouček, 1982

Figs 95–96 , 97–99

Epitranus crassicornis Bouček, 1982: 608–609 (♀, holotype, Laos (BPBM)).

##### Material.

1 ♀ (RMNH), “S. **Vietnam**: Dóng Nai, Cát Tiên N. P., *Ficus* trail, c. 100 m, 1–9.x.2005, Mal[aise] traps 1–8, C. v. Achterberg & R. de Vries, RMNH’05”; 1 ♀ (BPBM), “Vietnam, Vinh Long, 10.vi.1960, R.E. Leech”.

##### Diagnosis.

This species comes near *Epitranus erythrogaster* Cameron in the key to species by Bouček (1982) and Narendran (1989), but differs from *Epitranus erythrogaster* in having: 1) malar space with few setae (in *Epitranus erythrogaster* malar space setose); 2) ♀ antenna usually short (in *Epitranus erythrogaster* antenna of ♀ always longer and slenderer); and 3) flagellum and pedicel combined only 1.1 × head width (in *Epitranus erythrogaster* flagellum and pedicel much longer than 1.1 × head width).

##### Description.

♀, length of body 3.5 mm.

*Colour*. Head (except mouth region) black; mesosoma rusty brown with ventral part black; metasoma dark brown; wings hyaline.

*Head*. Clypeus dorsally concave, pilose, its distal margin vaguely tri-lobed; face with fine sculpture, scrobal part mainly reticulate to finely cross striate; scrobal carinae converging at more acute angle and reaching anterior ocellus; head width 1.3 × its height in anterior view; POL 1.8 × OOL; pedicel plus flagellum 2.5 × as long as scape; scape not reaching anterior ocellus; F1 subquadrate; following segments slightly transverse; clava subconical, twice as long as broad, shorter than 3 preceding segments combined.

*Mesosoma*. Mesosoma with pilosity on mesoscutum and scutellum slightly golden; propodeum with percurrent median area delimited by distinct submedian carinae which are subparallel-sided.

*Legs*. Hind femur with 9–10 differently sized teeth on outer ventral margin.

*Wings*. Wings hyaline, pilosity on fore wing extensive, forming a hairline or steak directed obliquely basad from apex of STV.

*Metasoma*. Petiole about 6 × as long as broad; metasoma 1.3 × as long as petiole, 1.4 × as long as its height in lateral view and blunt apically.

*Male*. Unknown.

##### Host.

Unknown.

##### Distribution.

Laos, Malaysia and Vietnam (Narendran 1989).

#### Epitranus erythrogaster

Taxon classificationAnimaliaHymenopteraChalcididae

Cameron, 1888

Figs 100–102 , 103

Epitranus erythrogaster Cameron, 1888: 119 (♀, lectotype selected by Bouček (1982), Japan (Nagasaki) (BMNH) (examined)).
Anacryptus sculpturatus Crawford, 1910: 129 (♀, lectotype, Philippines (Manila) (USNM) (lectotype selection and synonymized with Epitranus erythrogaster Cameron by Bouček 1982)).
Anacryptus kankauensis Masi, 1933: 12–14 (♀, lectotype, Taiwan (Kankau) (DEI) (lectotype selection and synonymized with *Epitranus erythrogaster* Cameron by Bouček 1982)).
Arretoceroides ceylonensis Mani, 1936: 128–129 (♂, holotype, Sri Lanka (Peradeniya) (lost?) (synonymized with *Epitranus erythrogaster* Cameron by Bouček (1982) by implication)).
Anacryptus raoi Mani & Kurian, 1953: 4–5 (“♂”, holotype, India (Uttar Pradesh, Agra) (lost?) (synonymized with *Epitranus erythrogaster* Cameron by Bouček (1982) by implication)).
Chalcitelloides devadatta W. Fernando, 1957: 212–213 (syntypes, Sri Lanka (Kalawewa) (lost?) (synonymized with *Epitranus erythrogaster* Cameron by Bouček (1982) by implication)).
Chalcitelloides ajatasattu W. Fernando. 1957: 241–242 (syntypes, Sri Lanka (Talawakele) (lost?) (synonymized with *Epitranus erythrogaster* Cameron by Bouček (1982) by implication)).
Pararretoceroides austini W. Fernando, 1957: 241–242 (syntypes, Sri Lanka (Talawakele) (lost?) (synonymized with *Epitranus erythrogaster* Cameron by Bouček (1982) by implication)).
Arretcera ambadevia Mani & Dubey (in Mani, Dubey, Kaul & Saraswat), 1973: 13–16 (♂, holotype, India (Borivile National Park nr. Mubai, Thana Hill (USNM) (synonymized with *Epitranus erythrogaster* Cameron by Bouček (1982)).
Arretocera nilamburense Mani & Dubey (in Mani, Dubey, Kaul & Saraswat), 1973: 16–17 (♀, holotype, India (Kerala, Nilambur) (USNM) (synonymized with *Epitranus erythrogaster* Cameron by Bouček (1982)).
Arretocera pallava Mani & Dubey (in Mani, Dubey, Kaul & Sarawat), 1973: 19–21 (♂, holotype, India (Tamil Nadu) (USNM) (synonymized with *Epitranus erythrogaster* Cameron by Bouček (1982)).
Arretocera tanjorensis Mani & Dubey (in Mani, Dubey, Kaul & Saraswat), 1973: 25–28 (♀, holotype, India (Andhra Pradesh) (USNM) (synonymized with *Epitranus erythrogaster* Cameron by Bouček (1982)).
Chalcitella nilamburensis Mani & Dubey (in Mani, Dubey, Kaul & Saraswat), 1974: 26–27 (“♂” (= ♀), holotype, India (Nilambur) (USNM) (synonymized with *Epitranus erythrogaster* Cameron by Bouček (1982)).
Arretocera malabarensis Mani & Dubey (in Mani, Dubey, Kaul & Saraswat), 1974: 28–29 (“♂” (= ♀), holotype, India (Kerala) (USNM) (synonymized with *Epitranus erythrogaster* Cameron by Bouček (1982)).
Epitranus acuminatus Husain & Agarwal, 1982c: 425 (♀, holotype, India (Aligarh) (ZDAMU) (examined) (synonymized with *Epitranus erythrogaster* Cameron by Narendran (1989)).

##### Material.

3 ♀ (RMNH, IEBR), “S. **Vietnam**: Dóng Nai, Cát Tiên N. P., *Ficus* trail, c. 100 m, 1–9.x.2005, Mal[aise] traps 1–8, C. v. Achterberg & R. de Vries, RMNH’05”; 2 ♂ (RMNH, IEBR), id., but 10–29.iv.2007; 1 ♀ (RMNH), id., but 9–26.iv.2007, Crocodile trail; 1 ♂ (RMNH), “C. Vietnam: Ha Tinh, Vu Quang N. P., 111 m, 18°19'40"N 105°26'29"E, 23.ix.–5.x.2009, Mal[aise] trap 23, R. de Vries, RMNH’09”; 4 ♀ (RMNH, IEBR), “S. Vietnam: Dak Lak, Chu Yang Sin N. P., n[ea]r dam, 500 m, Mal[aise] traps, 3–9.vi.2007, C. v. Achterberg & R. de Vries, RMNH’07”; 1 ♀ (RMNH), “N. Vietnam: Ninh Binh, Cuc Phuong N. P., n[ea]r entrance, c. 225 m, 1–15.v.2000, Malaise trap 2, Mai Phu Quy, RMNH’00”; 1 ♀ (IEBR), id., but near centre, 1.xi.–20.xii.2000; 3 ♂ (RMNH, IEBR) “S. Vietnam: Ninh Thuân, Núi Chúa N. P., northeast part, 90–150 m, 23–30.v.2007, Mal[aise] traps, C. v. Achterberg & R. de Vries, RMNH’07”; 151 ♀ + 120 ♂ (DZCU), “India, Kerala, T.C. Narendran & Party, 1984–1989”; 2 ♀ (DZCU), “India, Kerala, Calicut University Campus, 1979, Bouček & Narendran”.

##### Diagnosis.

This species comes near *Epitranus crassicornis* Bouček in the key to species by Bouček (1982) and Narendran (1989), but differs from it in having: 1) malar space setose (malar space with very few setae in *Epitranus crassicornis*); 2) antenna not unusually short (in *Epitranus crassicornis* antenna unusually short and stout, pedicel and flagellum combined only 1.1 × width of head), and 3) mesosoma usually black (in *Epitranus crassicornis* mesosoma red).

##### Description.

♀♂, length of body 2.4–4.9 mm.

*Colour*. Body mostly black with reddish colour on legs beyond coxae and at least ventrally on metasoma.

*Head*. Scrobal area flat with cross striae merging with reticulations; antenna extremely variable in length. In ♀ combined length of pedicel and flagellum 1.0–1.2 × as long as wide; T7 1.3 × as long as broad, scape in ♀ 0.8–1.2 × as long as width of vertex, usually reaching lower margin of anterior ocellus but sometimes shorter. In ♂ flagellum 1.3–2.6 × breadth of head; F1 2.0–3.4 × as long as broad and 0.4–0.8 × length of scape.

*Mesosoma*. Punctures on pronotum and mesoscutum close, with interstices narrower than diameter of a puncture, mostly smooth, occasionally rugulose; punctures on scutellum deeper than punctures of mesoscutum, interstices smooth and shiny.

*Wings*. Fore wing 2.8–3.0 × as long as broad, pilosity fairly extensive including a distinct line of setae from the end of fore wing venation to the base of wing and a hair line ventrally of costal cell.

*Legs*. Hind coxa twice as long as wide, outer ventral margin with 7–12 teeth; hind tibia with subbasal hump often indistinct or indicated by suberect setae and concealed small denticles; tarsal sulcus usually confined to one-third above basal insertion.

*Metasoma*. Petiole in ♀ 3.8–5.6 x, in ♂ 4.6–6.8 × as long as broad. Petiole shorter than metasoma in both sexes, 0.6–0.7 × length of metasoma.

##### Variation.

Bouček (1982) stated that “extreme forms are connected by numerous transitional forms”.

##### Host.

*Corcyra cephalonica* Stainton (Lepidoptera: Pyralidae) in rice storage in Kerala (India).

##### Distribution.

India, Nepal, Sri Lanka, Vietnam (Dai Lanh, Bian; Bam Me Thuot; Dalat), Thailand, Laos, Malaysia, Indonesia, Philippines, China (Taiwan), Japan (Bouček 1982; Narendran 1989).

#### Epitranus gauldi

Taxon classificationAnimaliaHymenopteraChalcididae

Bouček, 1982

Figs 104 , 105–106

Epitranus gauldi Bouček, 1982: 609 (♂, holotype, Brunei (BMNH)).

##### Material.

1 ♂ (RMNH), “C. **Vietnam**: Thu Thien Hué, Phong Dién N. R., n[ea]r base camp, 15 km W [of] Phong My, 50–100 m, 23.iii.–6.iv.2001, Mal[aise] traps 6-9, C. v. Achterberg & R. de Vries, RMNH’01”.

##### Distribution.

Brunei; Malaysia (Sarawak); Vietnam (new record).

#### Epitranus narendrani

Taxon classificationAnimaliaHymenopteraChalcididae

van Achterberg sp. n.

http://zoobank.org/A4D4A6AC-13E8-4A1D-A188-279E7AD61C04

Figs 107–109 , 110–112

##### Type material.

Holotype, ♀ (RMNH), “S. **Vietnam**: Dak Lak, Chu Yang Sin N. P., n[ea]r dam, c. 500 m, 3–9.vi.2007, Mal[aise] traps, C. v. Achterberg & R. de Vries, RMNH’07”.

##### Diagnosis.

The new species resembles *Epitranus ramnathi* but differs from it by having the clypeal shield with a median carina and shield ventrally distinctly narrowed (without median carina and shield evenly curved ventrally in *Epitranus ramnathi*), F1 (= third antennal segment) slender and distinctly narrowed basally (rather robust and less narrowed basally), hind coxa subparallel-sided basally (distinctly widened) and yellowish brown basally (black) and hind coxa dorsally mainly finely punctate (mainly transversely rugose).

##### Description.

Holotype, ♀, length of body 4.5 mm.

*Colour*. Head black, but clypeus, malar space, face, inner antennal area, mandible and narrowly orbita reddish brown; ocelli subhyaline; scape and pedicel pale yellow, remaining segments yellowish brown with apical segment mainly dark brown; mesosoma orange brown, but mesoscutum anteriorly, axillae, propodeum anteriorly, mesopleuron and mesosternum anteriorly black; mesosoma dorsally with short and sparse golden setae; tegulae pale yellow; metasoma dark reddish brown mixed with yellow ventrally and T1 (= petiole) blackish brown; legs brownish yellow but fore and middle tarsi, apices of fore and middle tibiae, middle coxa and hind tarsus pale yellowish, and medial disc of hind femur (both sides) dark reddish brown; wings hyaline with basal veins yellowish and apical veins brown; wing membrane hyaline.

*Head*. Head as broad as mesoscutum (including tegulae); width in anterior view 1.2 × its height; width in dorsal view 2.4 × its maximum length; POL:OOL:AOL:LOL = 32:13:16:11; eye height in lateral view 1.3 × its length; malar space 0.6 × eye height in lateral view and densely pale yellowish setose; frons laterally and dorsally with close, setigerous punctures, interstices narrower than diameter of a pit in most spaces, shiny and scrobal area with fine arcuate striation (Fig. 110); occiput and vertex similarly punctate as frons dorsally; frons in dorsal view rather flat; ocelli in broad triangle; clypeal shield with medio-longitudinal carina and narrowed apically, sides somewhat upturned, sub-torular carinae weak, narrow and interantennal space with short transverse carina ventrally and a median carina; pre-orbital carina weak; post-orbital carina strong, reaching posterior margin of gena; no malar ridge or carina, scape slender and distinctly reaching anterior ocellus, flagellum adpressed setose, distinctly narrowed basally and subparallel-sided apically (Fig. 110), F1 elongate, about 2.5 × longer than wide apically and slightly narrower than pedicel in dorsal view; submedial antennal segments slightly longer than wide.

*Mesosoma*. Mesosoma mainly with coarse umbilicate, setigerous and granulate punctures, interstices narrower than diameter of a puncture and with satin sheen; pronotum with anterior lateral carina distinct and complete, but antero-dorsal branch indistinct; lateral ledge present; scutellum slightly longer than broad, medially distinctly convex; propodeum with median area distinct, complete and anteriorly distinctly narrowed, connected to curved posterior lamella (Fig. 111) and its surroundings with short transverse rugae.

*Wings*. Fore wing with very long marginal vein (Fig. 107) and area below it glabrous and with an indistinct hair line present, short STV and apical fringe absent.

*Legs*. Hind coxa slender basally, about as long as hind femur, densely finely punctate and adpressed setose but with some long erect setae (Fig. 108); hind femur with a large basal tooth followed by 12 gradually smaller teeth and interspaces narrower than of *Epitranus ramnathi* (Fig. 109); hind tibia with slightly indicated subbasal tooth, adpressed setose and with some long erect setae, its tarsal sulcus confined to distal 0.4 of tibia; spine reaching trochanter in folded position.

*Metasoma*. Petiole (= T1) 5.5 × as long as its maximum width, 2.8 × as long as scutellum, 0.9 × length of T2; metasoma elongate, compressed and pointed apically.

*Male*. Unknown.

##### Host.

Unknown.

##### Distribution.

Vietnam.

**Remarks.** Named in honour of the deceased first author by the second author for his great contributions to our knowledge of the Oriental Hymenoptera.

#### Epitranus neonigriceps sp. n.

Taxon classificationAnimaliaHymenopteraChalcididae

http://zoobank.org/0EB28ED2-5DBB-4410-93D6-5B993DCBC6E6

Figs 113–114 , 115–116

##### Type material.

Holotype, ♀ (RMNH), “**Vietnam**, Viet Try, n[ea]r Tanh Son, Thuong Cuu, 20°59'N, 105°8'N, 360–400 m, 11–16.x.1999, Malaise traps, R. de Vries, RMNH’99”. Paratypes (RMNH, IEBR): 3 ♀, same data as holotype; 2 ♀, “N. Vietnam: Ninh Binh, Cuc Phuong N. P., n[ea]r entrance, c. 225 m, 15–27.v.2000, Mai Phu Quy, RMNH’00”; 1 ♀, id., but near centre, 1.xi.–20.xii.2000.

##### Diagnosis.

This new species comes to *Epitranus nigriceps* Bouček in the keys by Bouček (1982) and Narendran (1989), but differs from it in having: 1) interstices between punctures narrower than diameter of a puncture on mesoscutum and axillae (in *Epitranus nigriceps* interstices between punctures broader than diameter of a puncture in many places on mesoscutum and axillae); 2) post-spiracular teeth present (in *Epitranus nigriceps* post-spiracular teeth absent); 3) post-orbital carina reaching posterior margin of gena (in *Epitranus nigriceps* post-orbital carina running upwards to near lateral ocellus); 4) eyes sparsely setose (in *Epitranus nigriceps* eyes bare); 5) funicular segments mostly transverse (in *Epitranus nigriceps* funicular segments slightly elongate); 6) mesosoma and metasoma darker (in *Epitranus nigriceps* mesosoma and metasoma more reddish); 7) metasoma pointed at apex (in *Epitranus nigriceps* metasoma rather blunt at apex); 8) sublateral and submedian carinae of propodeum a little convex (in *Epitranus nigriceps* sublateral and submedian carinae of propodeum straight), and 9) POL 1.3 × OOL (in *Epitranus nigriceps* POL 1.7 × OOL).

##### Description.

Holotype, ♀, length of body 3.5 mm.

*Colour*. Head black; eyes gray with punctures, reflecting yellow spots; ocelli pale reflecting yellow; scape and pedicel yellow; anellus yellowish brown; remaining segments pale brown; clypeus and mandibles pale brownish yellow; mesosoma black with tegulae pale yellow; metasoma liver brown mixed with black; fore and mid legs yellow except fore coxa slightly darker basally; hind coxa, trochanter and femur reddish brown with darker median part of outer disc of hind femur and brownish yellow apex of hind coxa; hind tibia pale brownish yellow; all tarsi pale yellow; wings hyaline with veins pale yellowish hyaline.

*Head*. Head as broad as mesoscutum (including tegulae); width in anterior view 1.2 × its height; width in dorsal view twice its length; POL:OOL:AOL:LOL = 8:6:5:4; eye height in profile 1.2 × its length; malar space 0.6 × eye height in profile; face with close, setigerous punctures, interstices narrower than diameter of a pit in most spaces, smooth, shiny; occiput and vertex similarly punctate as face; face in dorsal view broadly convex; ocelli in broad triangle; scrobal area with arcuate striation; clypeal shield tri-lobed at apex, sides upturned, concave surface dull with some short setae, sub-torular carinae weak, narrow interantennal space with small lamina; pre-orbital carina weak; post-orbital carina strong, reaching posterior margin of gena; no malar ridge (carina), scape not reaching anterior ocellus, flagellum barely thickened distally; F1 as broad as pedicel in dorsal view; pre-claval segments slightly wider than long.

*Mesosoma*. Mesosoma with close umbilicate, setigerous punctures, interstices narrower than diameter of a puncture, smooth and shiny except reticulate area near anterior margin of mesoscutum; pronotum with anterior lateral carina very weakly represented, not high; lateral ledge present. Scutellum longer than broad (9:8), its dorsum moderately convex; propodeum with median area precurrent, with a pair of submedian carinae enclosing areola, postspiracular teeth distinct.

*Wings*. Fore wing with short STV, fringe absent, pilosity distinct on distal half, glabrous below veins and no distinct hairline.

*Legs*. Hind coxa with distinct close setigerous punctures on outer side, narrow interstices smooth; hind femur with a large basal tooth followed by 8–9 gradually smaller teeth; tibia with slightly indicated hump, moderate dorsal pilosity, tarsal sulcus confined to distal 0.4 of tibia; spine reaching trochanter in folded position.

*Metasoma*. Petiole 3.8 × as long as broad, twice as long as scutellum, 0.8 × length of T1; metasoma elongate, apex pointed.

*Male*. Unknown.

##### Host.

Unknown.

##### Distribution.

Vietnam.

##### Variation.

In some paratypes the funicle is more yellowish brown and the metasoma is darker than in the holotype.

#### Epitranus nigriceps

Taxon classificationAnimaliaHymenopteraChalcididae

Bouček, 1982

Figs 117–119 , 120–121

Epitranus nigriceps Bouček, 1982: 600–602 (♀, holotype, India (BMNH)).

##### Material.

1 ♀ (RMNH), “S. **Vietnam**: Dóng Nai, Cát Tiên N. P., *Ficus* trail, c. 100 m, 1–9.x.2005, Mal[aise] traps 1–8, C. v. Achterberg & R. de Vries, RMNH’05”; 1 ♂ (BPBM), “Vietnam, Fyan, 1000 m, vii.–viii.1961”.

##### Distribution.

India; Sri Lanka; Malaysia (Sarawak); Vietnam (Bouček 1982).

#### Epitranus parvidens

Taxon classificationAnimaliaHymenopteraChalcididae

(Strand, 1911)

Anacryptus parvidens Strand, 1911: 7–8 (♂, lectotype, Indonesia (ZMHU)).
Epitranus parvidens ; Bouček 1982: 615–617.
Arretocera ultima Mani & Dubey, 1973: 28–30 (♀, holotype, India (USNM) (synonymized with Anacryptus parvidens Strand by Bouček (1982)).
Chalcitella monticola Mani & Dubey, 1973: 10–12 (♂, holotype, India (USNM) (synonymized with Anacryptus parvidens Strand by Bouček (1982)).
Chalcitella borivilia Mani & Dubey, 1973: 8–10 (♂, holotype, India (USNM) (synonymized with Anacryptus parvidens Strand by Bouček (1982)).
Chalcitella malabarensis Mani & Dubey, 1974: 24–25 (♀, holotype, India (USNM) (synonymized with Anacryptus parvidens Strand by Bouček (1982)).
Epitranus nigrus Hussain & Agarwal, 1982c: 423–425 (♀, holotype, India (ZDAMU) (synonymized with Anacryptus parvidens Strand by Narendran (1989)).

##### Distribution.

Indonesia (Bintang); India; Sri Lanka; Malaysia (West Malaysia); Vietnam (Ban Me Thuot; Bouček 1982).

#### Epitranus oxytelus

Taxon classificationAnimaliaHymenopteraChalcididae

Bouček, 1982

Figs 122–124 , 125

Epitranus oxytelus Bouček, 1982: 616–617 (♀, holotype, India (BMNH)).

##### Material.

1 ♀ (RMNH), “S. **Vietnam**: Dak Lak, Chu Yang Sin N. P., n[ea]r dam, c. 500 m, Malaise traps, 3–9.vi.2007, Mal[aise] traps, C. v. Achterberg & R. de Vries, RMNH’07”; 1 ♀ (IEBR), “N. Vietnam: Ninh Binh, Cuc Phuong N. P., n[ea]r centre, c. 225 m, 15–27.v.2000, Malaise trap 2, Mai Phu Quy, RMNH’00”; 1 ♀ (RMNH), id., but 10.ii.–15.iii.2000; 1 ♀ (BPBM), “Vietnam, Vinh Long, 10.vi.1960, R.E. Leech”.

##### Distribution.

India; Vietnam (new record).

#### Epitranus ramnathi

Taxon classificationAnimaliaHymenopteraChalcididae

(Mani & Dubey, 1973)

Figs 126–128 , 129–131

Arretocera ramnathi Mani & Dubey, 1973: 21–23 (♀, holotype, India (BMNH)).
Epitranus ramnathi ; Bouček 1982: 598–600.
Chalcitella annexia Mani & Dubey, 1973: 7–8. (♀, holotype, India (USNM) (synonymized with Arretocera ramnathi Mani & Dubey by Bouček (1982)).
Arretocera ophiomontana Mani & Dubey, 1973: 18–19. (♀, holotype, Nepal (USNM) (synonymized with Arretocera ramnathi Mani & Dubey by Bouček (1982)).
Arretocera sancti-johani Mani & Dubey, 1973: 23–25. (♀, holotype, Nepal (USNM) (synonymized with Arretocera ramnathi Mani & Dubey by Bouček (1982)).
Epitranus giganticus Hussain & Agarwal, 1982c: 427 (♀, holotype, India (ZDAMU) (synonymized with Arretocera ramnathi Mani & Dubey by Narendran (1989)).
Epitranus melongenus Hussain & Agarwal, 1982c: 427 (♀, holotype, India (ZDAMU) (synonymized with Arretocera ramnathi Mani & Dubey by Narendran (1989)).
Epitranus simplexus Hussain & Agarwal, 1982c: 427–429 (♀, holotype, India (ZDAMU) (synonymized with Arretocera ramnathi Mani & Dubey by Narendran (1989)).
Epitranus areolatus Hussain & Agarwal, 1982c: 429 (♀, holotype, India (ZDAMU) (synonymized with Arretocera ramnathi Mani & Dubey by Narendran (1989)).
Epitranus rossicorpus Hussain & Agarwal, 1982c: 429–431 (♀, holotype, India (ZDAMU) (synonymized with Arretocera ramnathi Mani & Dubey by Narendran (1989)).

##### Material.

1 ♀ (RMNH), “**Vietnam**: Viet Try, n[ea]r Tanh Son, Thuong Cuu, 20°59'N, 105°8'E, 350–400 m, 11–16.x.1999, Malaise traps, R. de Vries, RMNH’99”; 1 ♀, “N. Vietnam: Ninh Binh, Cuc Phuong N. P., n[ea]r entrance, c. 225 m, 15.iv.–1.v.2000, Malaise trap 2, Mai Phu Quy, RMNH’00”; 1 ♀ (RMNH), “S. Vietnam: Dak Lak, Chu Yang Sin N. P., Krong K’Mar, 740–900 m, 2–10.vi.2007, Malaise traps, C. v. Achterberg & R. de Vries, RMNH’07”.

##### Distribution.

India, Nepal, Vietnam (new record).

##### Variation.

The head of Vietnamese specimens is mainly black (Figs 126, 129), but Indian specimens may have the head more or less reddish brown (Hussain and Agarwal 1982c).

#### Haltichella

Taxon classificationAnimaliaHymenopteraChalcididae

Spinola, 1811

Figs 132 , 133–134 , 135–136 , 137–138 , 139 , 140–142

Haltichella Spinola, 1811: 148. Type species: Chalcis bispinosa Fabricius; proposed by Masi (1929a) (= Chalcis rufipes Olivier; as proposed by Schmitz (1946)).
Halticella Stephens, 1829: 36. Unjustified emendation.
Microchalcis Kieffer, 1905: 255. Type species: Microchalcis quadridens Kieffer, by monotypy (synonymised with Haltichella Spinola by Bouček (1952)).
Haltichellodes Steffan, 1955: 384. Type species: Haltichella pulla Steffan, (by monotypy, described as subgenus of Haltichella and synonymised Haltichella Spinola by Bouček 1988b).

##### Diagnosis.

This genus comes very near *Neohaltichella* Narendran in the key to Oriental genera of Chalcididae by Narendran (1989), but differs from *Neohaltichella* in having: 1) base of T1 with longitudinal carinae (in *Neohaltichella* T1 without any carinae); 2) T1 relatively larger than that of *Neohaltichella*; 3) head and mesosoma less stout than that of *Neohaltichella*, and 4) body not densely pubescent (in *Neohaltichella* body densely pubescent).

##### Description.

Hind tibia with an additional carina externally, T1 with at least a pair of longitudinal carinae at base; often supplemented by additional striae or carinae which are often anteriorly united by a transverse carina; T1 relatively larger (exceeding mostly half of metasoma or reaching middle of metasoma); apex of scutellum bi-dentate or bi-lobed; pronotum with anterior carina absent or confirmed to lateral thirds; dorsal part of horseshoe-shaped carina indistinct or irregular.

##### Hosts.

Noyes (2011) listed the following families of Lepidoptera (Bucculatricidae; Gelechiidae; Momphidae; Notodontidae; Oecophoridae; Pyralidae; Tortricidae) and Hymenoptera (Braconidae; Ichneumonidae).

##### Distribution.

Cosmopolitan (Bouček 1988b; Noyes 2011).

##### Key to Vietnamese species of *Haltichella* Spinola (based on females)

**Table:** 

1	Metasoma with more than two basal carinae (usually up to five carinae) on T1 (Fig. 82); [antenna strongly clavate (Fig. 83), 0.6–0.7 × its length; apex of scutellum with two diverging teeth]	***Haltichella clavicornis* Ashmead**
–	Metasoma with only two carinae at base of T1	**2**
2	Metasoma long and pointed in ♀, about 3.5 × as long as its height in lateral view, much longer than mesosoma; apex of scutellum with two small teeth; hind tibia reddish brown	***Haltichella delhensis* Roy & Farooqi**
–	Metasoma shorter and less pointed in ♀, about twice as long as its height in lateral view, slightly shorter than or at the most as long as mesosoma; apex of scutellum with two large teeth; hind femur black with base brown	***Haltichella nipponensis* Habu**

**Figure 132.**
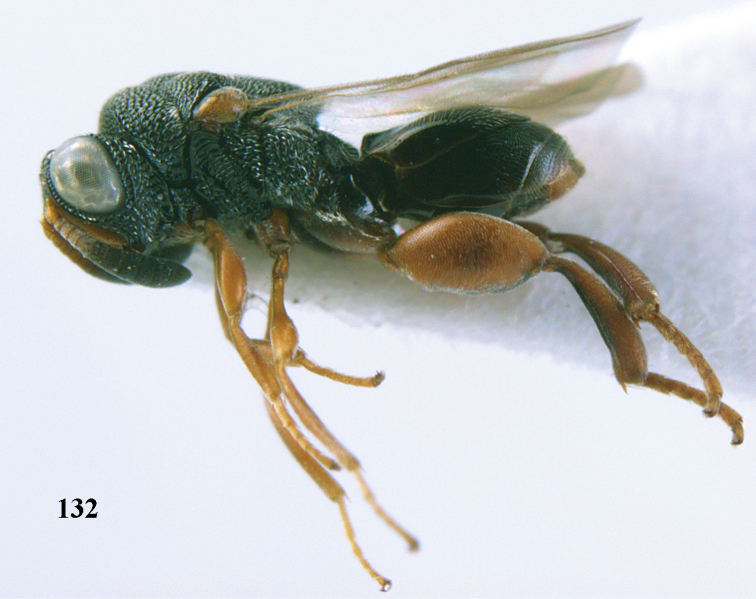
*Haltichella clavicornis* (Ashmead), ♀, Cát Tiên N.P., habitus lateral.

**Figures 133–134.**
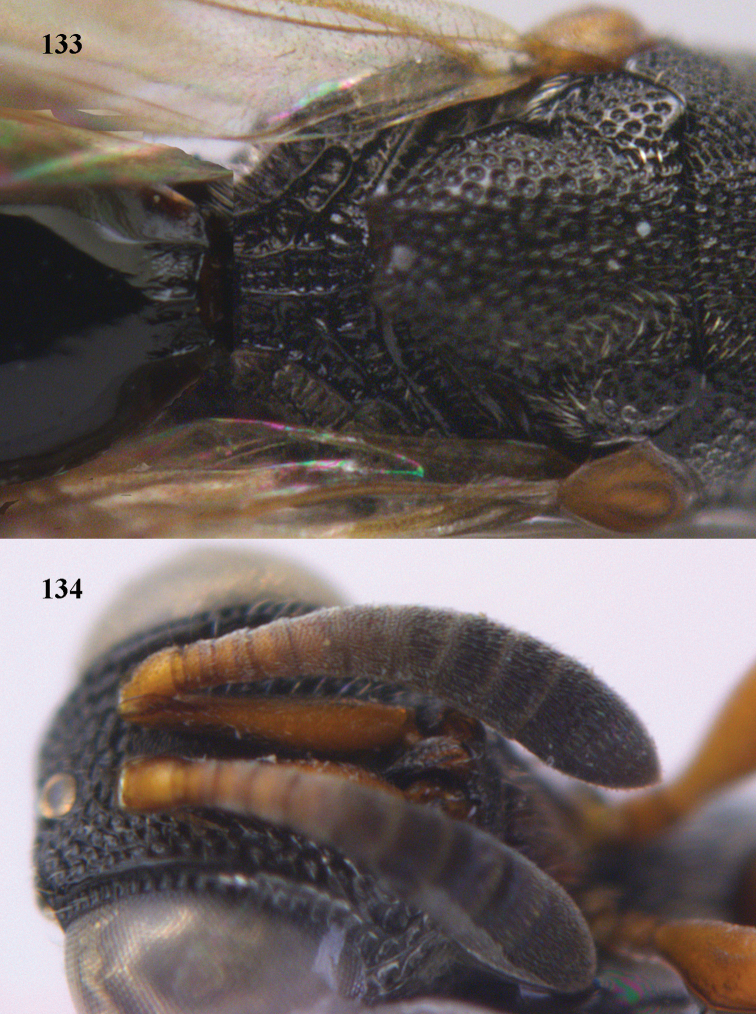
*Haltichella clavicornis* (Ashmead), ♀, Cát Tiên N.P. **133**. apical half of mesosoma and base of metasoma dorsal **134** antenna antero-lateral.

**Figures 135–136.**
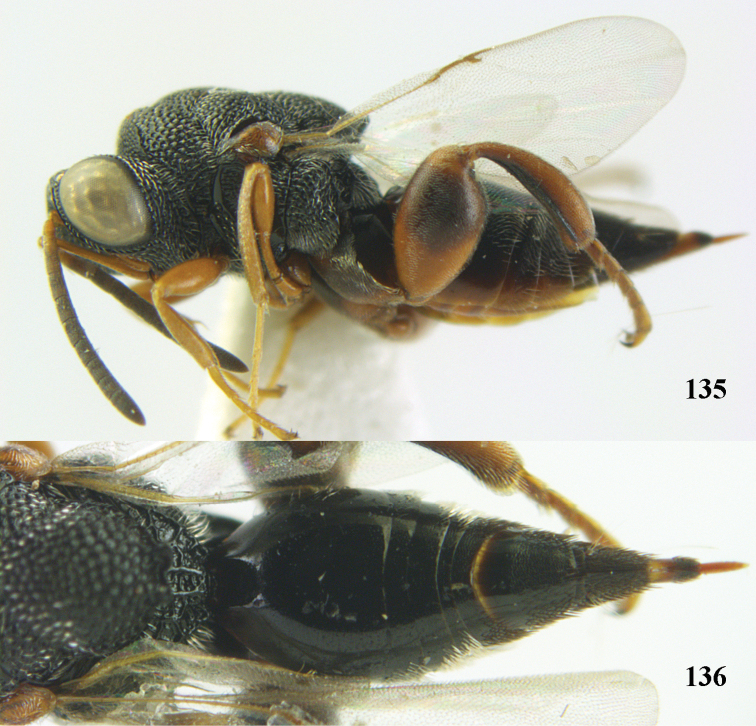
*Haltichella delhensis* Roy & Farooqi, ♀, Cát Tiên N.P. **135** habitus lateral **136** metasoma dorsal.

**Figures 137–138.**
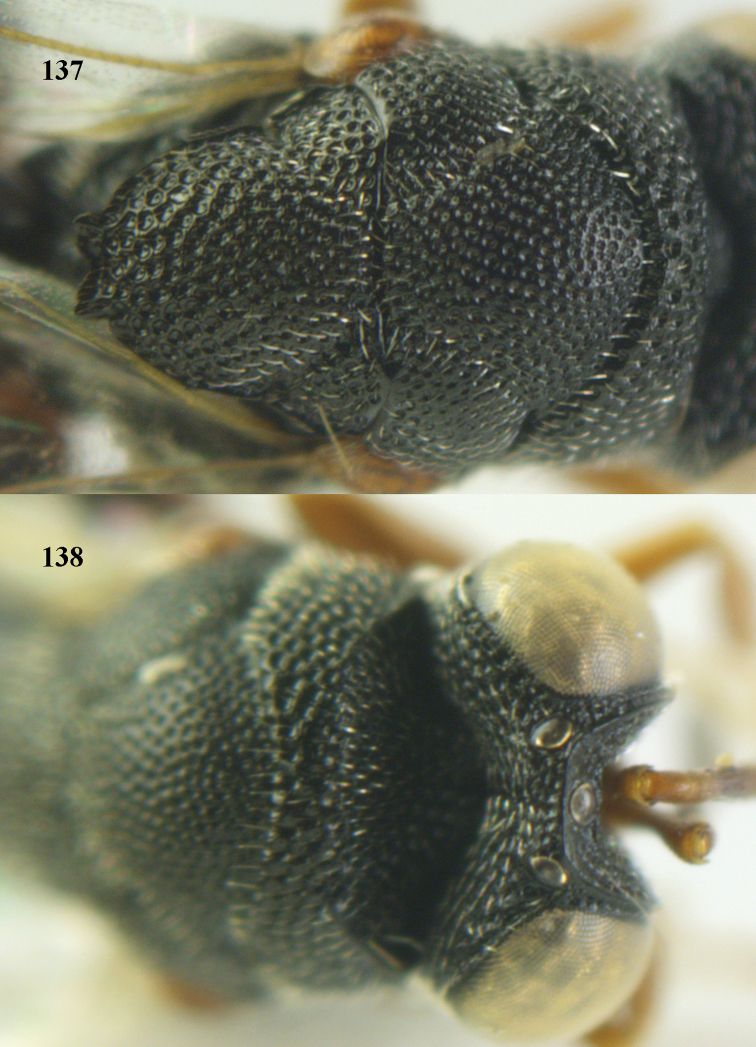
*Haltichella delhensis* Roy & Farooqi, ♀, Cat Tien N.P. **137** mesosoma dorsal **138** head dorsal.

**Figure 139.**
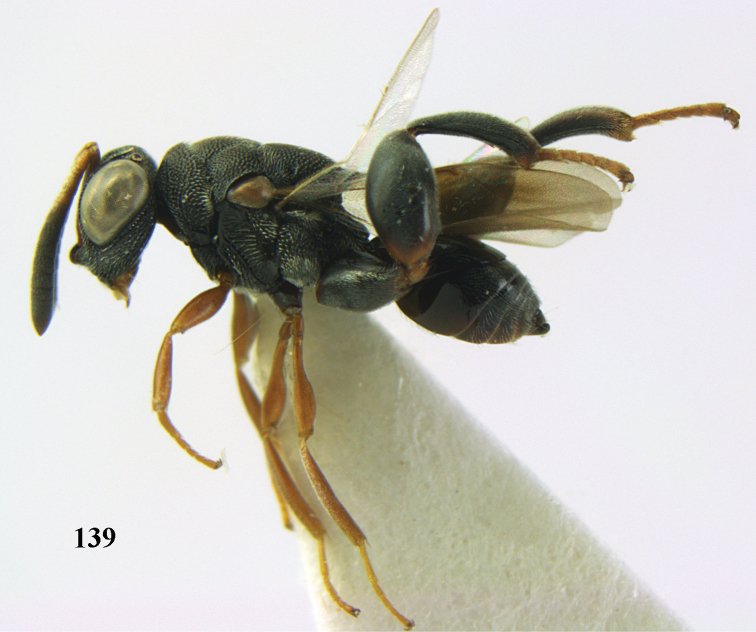
*Haltichella nipponensis* Habu, ♀, Cát Tiên N.P., habitus lateral.

**Figures 140–142.**
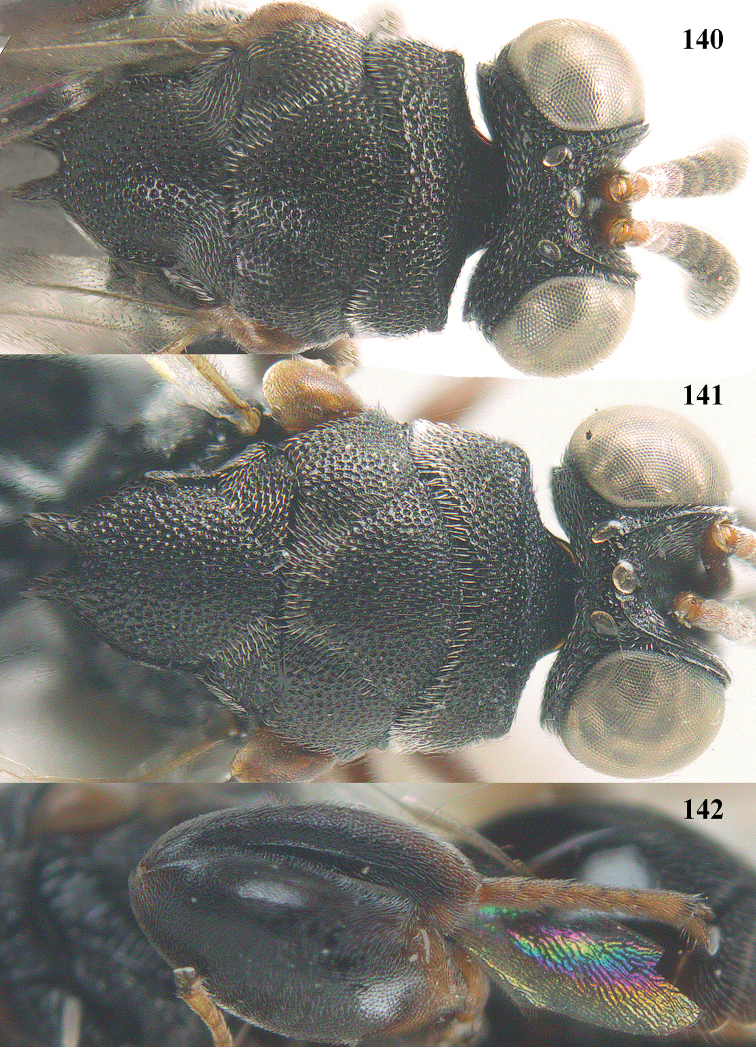
*Haltichella nipponensis* Habu, ♀, Chu Yang Sin N. P., but **141** ♂, Cát Tiên N.P. **140–141** head and mesosoma dorsal **142** hind leg lateral.

#### Haltichella clavicornis

Taxon classificationAnimaliaHymenopteraChalcididae

(Ashmead, 1904)

Figs 132 , 133–134

Stomatoceras clavicornis Ashmead, 1904: 148 (holotype, Japan (USNM) examined).
Haltichella clavicornis ; Habu 1960: 241.
Haltichella macroclava Roy & Farooqi, 1984: 27 (♀, holotype, India (INPC) examined) (synonymised with Haltichella clavicornis (Ashmead) by Narendran (1989)).

##### Material

(RMNH, IEBR). 2 ♀, “N. **Vietnam**: Ninh Binh, Cuc Phuong N. P., nr. entrance, c 225 m, 15.iv.–1.v.2000, Malaise trap 2, Mai Phu Quy, RMNH’00”; 1 ♀, “S. Vietnam: Dak Lak, Chu Yang Sin N. P., n[ea]r dam, c. 500 m, 3–9.vi.2007, Mal[aise] traps, C. v. Achterberg & R. de Vries, RMNH ’07”; 1 ♂, “S. Vietnam: Dóng Nai, Cát Tiên N. P., Bot[anic] Garden, 13–20.v.2007, Mal. traps 14–19, C. v. Achterberg & R. de Vries, RMNH’07”; 1 ♀, id., 15–29.v.2007, M.P. Qui, N.T. Manh & C. v. Achterberg; 1 ♀, id., but Bird trail, 1–8.iv.2007, Mai Phu Quy & Nguyen Tanh Manh; 1 ♀, id., but Malaise traps 9–12, 1–9.x.2005; 1 ♀, id., but Dong trail, 1–9.iv.2007; 1 ♂, id., but *Lagerstroemia* trail, Malaise traps 20–23, 14–20.v.2007; 1 ♂ (BPBM), “Vietnam, W. Quat, 19–21.v.1960”.

##### Diagnosis.

This is a unique species, which can be separated from all other *Haltichella* species by its unusually swollen clava of the antenna of ♀, the scutellum with a median longitudinal fovea and the diverging teeth at apex of scutellum.

##### Description.

♀, length of body: 3.0–3.5 mm.

*Colour*. Black, except for following parts: from scape to F6 varying from dark brown to black; mid coxa dark brown; other segments of fore and mid legs except coxae reddish or yellowish brown with middle part of femora slightly darker; hind femur pale brown or reddish brown with dark patch on disc; hind tibia and tarsi reddish brown.

*Head*. Antenna distinctly clavate.

*Mesosoma*. Scutellum flat with a median longitudinal fovea; apex of scutellum bi-dentate, short teeth diverging, not parallel.

*Metasoma*. T1 occupying two-third to three-fourths of metasoma; with usually 5 longitudinal basal carinae (fig. 82); T1 smooth and shiny.

*Male*. Length 2.8–3.2 mm. Antenna longer than that of ♀, not clavate.

##### Host.

Unknown.

##### Distribution.

India, Nepal, Laos, Vietnam, Malaysia, Philippines (Narendran 1989).

#### Haltichella delhensis

Taxon classificationAnimaliaHymenopteraChalcididae

Roy and Farooqi, 1984

Figs 135–136 , 137–138

Haltichella delhensis Roy & Farooqi, 1984: 26 (♀, holotype, India (Delhi) (INPC) (examined)); Narendran 1989: 148, 151 (diagnosis and keyed).

##### Material

(RMNH, IEBR). 2 ♀, “S. **Vietnam**: Dóng Nai, Cát Tiên N. P., Dong trail, Malaise traps, c. 100 m, 1–8.iv.2007, Mai Phu Quy & Nguyen Tanh Manh, RMNH’07”.

##### Diagnosis.

This species can be distinguished from other species by its relatively long and pointed metasoma and apex of scutellum with two relatively small teeth.

##### Description.

♀, length of body 3.6–4.4 mm.

*Colour*. Black, legs and tegulae reddish brown; scape, pedicel, anellus, ring segment, F1, F2, brownish to reddish brown; rest of flagellum dark brown.

*Head*. Pre- and post-orbital carinae present; geno-temporal furrow indistinct; scrobe reaching anterior ocellus.

*Mesosoma*. Mesosoma with close pits and interstices narrow; apex of scutellum with two small teeth.

*Metasoma*. Metasoma long and pointed (Fig. 85).

*Male*. Unknown.

##### Host.

Unknown.

##### Distribution.

India, Vietnam (new record).

##### Variation.

Vietnamese specimens have the hind femur with black patch on the disc and are slightly larger than the type specimens. In one specimen the fore and middle legs are more yellowish brown.

#### Haltichella nipponensis

Taxon classificationAnimaliaHymenopteraChalcididae

Habu, 1960

Figs 139 , 140–142

Haltichella nipponensis Habu, 1960: 245 (♀, holotype, Japan (NIAS)).

##### Material

(RMNH, IEBR, partly labelled as *Haltichella achterbergi*). 1 ♀, “S. **Vietnam**: Dak Lak, Chu Yang Sin N. P., n[ea]r dam, 740–790 m, 2–10.vi.2007, Malaise traps, C. v. Achterberg & R. de Vries, RMNH’07”; 1 ♀, id., but Krong K’Mar, 740–790 m, 2–10.vi.2007; 1 ♀, id., but Malaise traps 13–23, 590–840 m, 22-26.x.2005; 1 ♀ + 1 ♂, “S. Vietnam: Dóng Nai, Cát Tiên N. P., *Ficus* trail, Malaise trap, c. 100 m, 9–30.iv.2007, M. P. Quy & N. T. Manh, RMNH’07”; 1 ♂, id., but Botanical Garden, Malaise traps 14–19, 13–20.v.2007; 2 ♀, id., but Dong trail, 13–19.v.2007; 1 ♂, id., but 1–8.iv.2007; 2 ♂, eco-trail, Malaise traps 25-29, 13–20.v.2007; 1 ♂, id., but Bird trail, Malaise traps 30–35, 15–20.v.2007; 1 ♂, “Vietnam: Ninh Thuân, Núi Chúa N. P., Northeast part, Malaise trap, 90–150 m, 23–30.v.2007, C. v. Achterberg & R. de Vries, RMNH’07; 3 ♀, “N. Vietnam: Vinh Phuc, Tam Dao, 1050–1175 m, 14–17.v.2007, Malaise trap, C. v. Achterberg & R. de Vries, RMNH’07”.

##### Diagnosis.

This species resembles the European *Haltichella rufipes* (Olivier) in general appearance, but the apical teeth of scutellum are more widely spaced (more than 1.5 × as wide as distance from each other in *Haltichella nipponensis*) (in *Haltichella rufipes* distance almost equal to their length; Habu 1960). Differs from *Haltichella achterbergi* Narendran, 1990, by having the dorsal carinae of the first metasomal tergite widely spaced (close to each other in *Haltichella achterbergi*) and apical teeth of scutellum diverging (parallel-sided in *Haltichella achterbergi*).

##### Description.

♀, length of body 2.8–4.1 mm.

*Colour*. Black; antenna brown, slightly reddish, F3 to tip of clava blackish; apical teeth of scutellum large and slightly reddish; fore and middle coxae, trochanters, femora slightly dark; hind coxa and femur almost black or reddish black with hind trochanter and base of hind femur brown; hind tibia almost black or reddish.

*Head*. Antennal scape reaching anterior ocellus, longer than combined length of F1 to F4; eyes pubescent.

*Legs*. Hind coxa with coxal tooth on baso-dorsal side; hind femur 2.3 × as long as wide, smooth without micro-sculpture.

*Male*. Length of body 2.7–3.1 mm. Differs from ♀ mainly by having micro-sculpture on T1.

##### Variation.

In Vietnamese specimens fore and middle legs (except coxae) are more yellowish brown with reddish tinge than in Indian specimens and scutellum not reddish apically, but black.

##### Distribution.

Japan, India, Vietnam (new record).

#### Heydoniella

Taxon classificationAnimaliaHymenopteraChalcididae

Narendran, 2003

**Figures 143–144.**
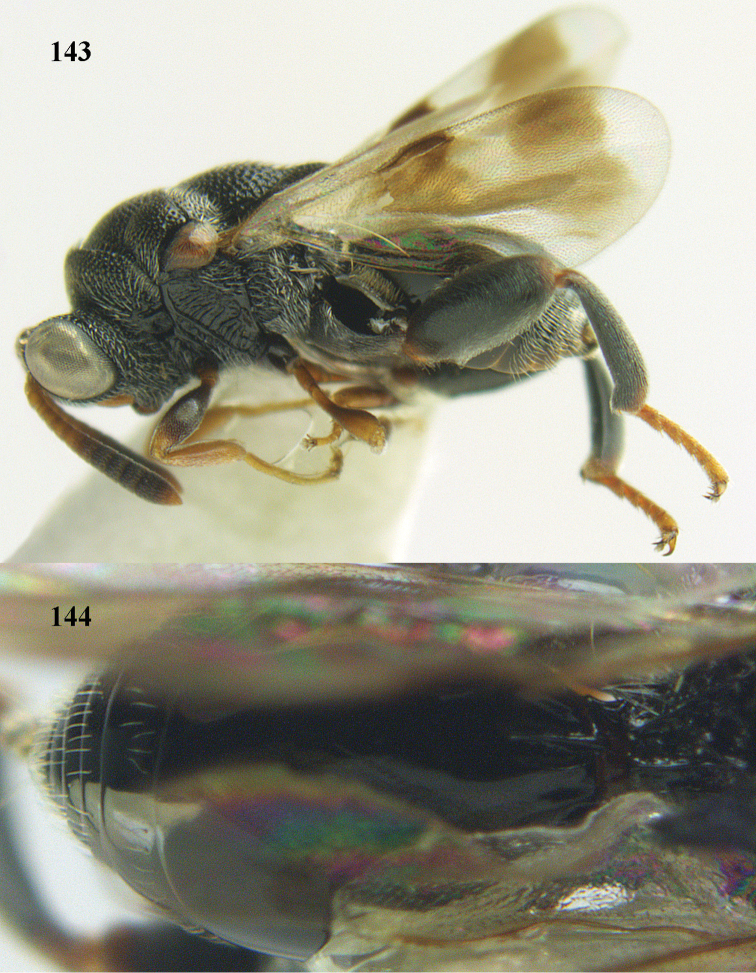
*Heydoniella vietnamensis* sp. n., ♀, holotype. **143** habitus lateral **144** metasoma dorsal.

**Figures 145–146.**
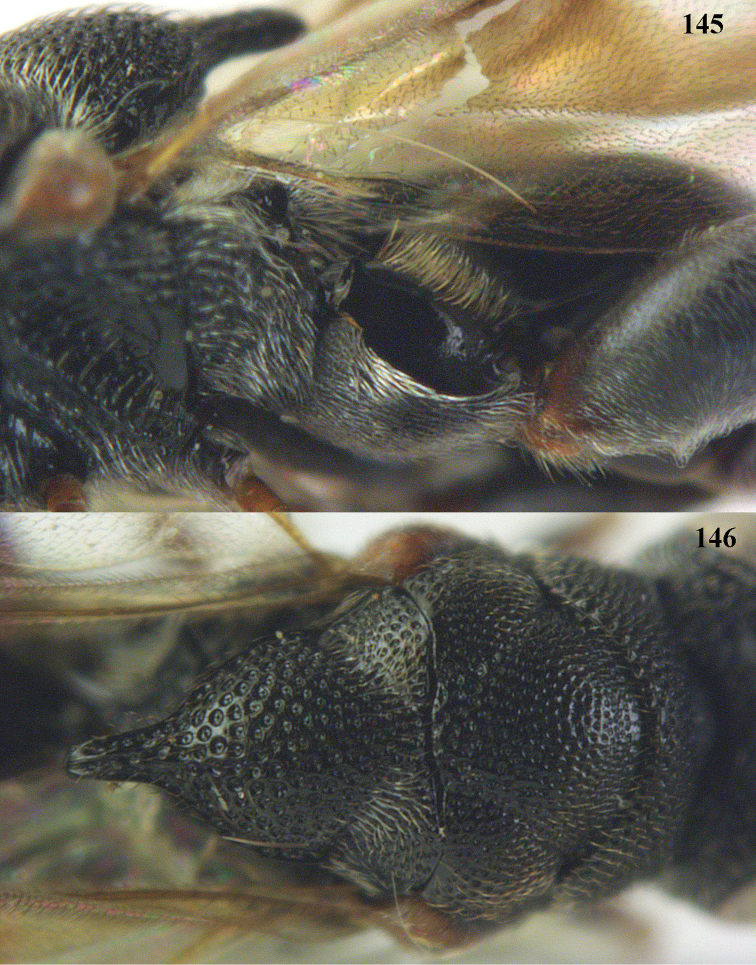
*Heydoniella vietnamensis* sp. n., ♀, holotype. **145** hind coxa lateral **146** mesosoma dorsal.

Figs 143–144 , 145–146

Heydoniella Narendran, 2003: 85. Type-species: Heydoniella sarawakensis Narendran, by monotypy.

##### Diagnosis.

This genus comes near *Oxycoryphe* Kriechbaumer in having scutellum prolonged posteriorly; hind tibia with an additional carina and T1 with basal carinae and fovea. *Heydoniella* differs from *Oxycoryphe* in having: 1) pronotum without a median tooth or tubercle or triangle (in *Oxycoryphe* pronotum with a median tooth or tubercle or at least a median triangle); 2) hind coxa with a characteristic tuft of white dorsal setae (Fig. 94; in *Oxycoryphe* hind coxa without such characteristic white tuft of setae dorsally); 3) T1 unusually large, exceeding well middle of metasoma (in *Oxycoryphe* T1 not unusually large and not exceeding middle of metasoma), and 4) fore wing with two infumate bands (in *Oxycoryphe* fore wing without such bands). Shape of T1 of *Heydoniella* resembles that of *Nearretocera* Girault, which belongs to the tribe Hybothoracini, whereas *Heydoniella* belongs to the tribe Haltichellini.

##### Description.

Pronotum without a median tooth or tubercle or triangular area; scutellum at most slightly exceeding base of metasoma; fore wing with two large infumate bands; hind coxa dorsally with a bunch of white pubescence and a tooth; hind tibia with an additional carina on outer side; metasoma with basal carinae; T1 exceeding middle of metasoma, its posterior margin convex.

##### Host.

Unknown.

##### Distribution.

Malaysia (Sarawak; Narendran 2003), Vietnam (new record).

#### Heydoniella vietnamensis sp. n.

Taxon classificationAnimaliaHymenopteraChalcididae

http://zoobank.org/DCEF5A95-1D5F-4EDC-ACEE-016CEEB699A3

Figs 143–144 , 145–146

##### Type material.

Holotype, ♀ (RMNH), “S. **Vietnam**: Dak Lak, Chu Yang Sin N. P., Krong K’Mar, Mal. traps, 740–900 m, 2–10.v.2007, C. v. Achterberg & R. de Vries, RMNH’07”. Paratype (IEBR): 1 ♀, same data as holotype.

##### Diagnosis.

This new species differs from the only other known species, viz. *Heydoniella sarawakensis* Narendran in having: 1) head with transverse carina anterior to scrobe below anterior ocellus absent (in *Heydoniella sarawakensis* with a transverse carina below anterior ocellus); 2) POL 7.5 × OOL (in *Heydoniella sarawakensis* POL about 3.7 × OOL); 3) clava 1.5 × as long as its width (in *Heydoniella sarawakensis* clava 1.8 × its width); 4) length of scutellar prolongation 0.5 × width of scutellum (in *Heydoniella sarawakensis* length of scutellar prolongation 0.3 × width of scutellum); 5) T1 1.1 × its width (in *Heydoniella sarawakensis* T1 2.1 × as long as wide), and 6) CC 4 × as long as MV (in *Heydoniella sarawakensis* CC 5.3 × as long as MV).

##### Description.

Holotype, ♀, length of body 3.2 mm.

*Colour*. Black; eyes and ocelli reflecting gray; scape, pedicel and anellus pale brownish yellow; F1 to F3 pale reddish brown; remaining segments of antenna dark with apex of clava brownish. All coxae black; all trochanters pale brown; fore and mid femora pale brownish yellow except median part externally black; fore tibia and tarsi pale yellow; mid tibia dark brown with base and apex pale yellow; mid tarsi pale yellow; hind femur completely black; hind tibia black with apex and base pale brown; tarsi pale brown; wings hyaline with two infumate bands on fore wing; veins dark brown; pubescence on body silvery.

*Head*. Width of head in anterior view 1.4 × its height; width in dorsal view 2.4 × its length, almost equal to width of mesosoma (including tegulae); face concave, scrobe deep, cross reticulate-striate, margins ecarinate, weak thin transverse carina below anterior ocellus absent; parascrobal area with deep setigerous close pits, eyes with sparse short pubescence; MS weak but distinct, irregular; maximum diameter of eye in lateral view 2.6 × malar space; pre-orbital carina running upwards and crossing to posterior part of vertex through OOL and meeting post-orbital carina; temples narrow; vertex narrow; occiput moderately excavate, deeply declining from ocelli, deeply punctate; POL 7.5 × OOL; AOL 3 × OOL, shorter than LOL; shortest distance between eyes in dorsal view = twice POL. Antennal segments shorter towards apical part; antennal formula 11173; scape not reaching anterior ocellus; relative L:W of antennal segments:scape = 22:5; pedicel = 5:3; anellus = 3:3; F1 = 3:4; F2 = 4:4; F3 = 5:4; F4 = 4:5; F5 = 4:5; F6 = 4:6; F7 = 4:6; clava = 9:6.

*Mesosoma*. Mesosoma convex, with distinct, deep, umbilicate, setigerous pits, interstices between pits ecarinate, weakly reticulate except strongly reticulate anterior part; posterior margin of pronotum widely concave; notauli shallow, with pits; tegulae pubescent; axillae with stronger and relatively longer pubescence than those of scutellum; scutellum length including apical prolongation 1.6 × its width, apex prolonged posteriorly, slightly exceeding base of metasoma, length of apical prolongation 0.5 × width of scutellum; apical prolongation with a median raised ridge. Propodeum with a pair of distinct submedian carinae; plical carina strongly developed, interstices distinctly alveolate and with deep pits; lateral teeth indistinct.

*Wings*. Fore wing 2.3 × as long as its width; CC 4 × as long as MV; PMV shorter than STV, with 2 infumate bands connected medially, disc densely pilose; speculum absent.

*Legs*. Hind coxa with an anterior dorsal tooth and a bunch of characteristic dense white setae dorsally (Fig. 145); hind femur with a row of teeth on ventral margin, inner side without tooth or protuberance; hind tibia with an extra outer carina.

*Metasoma*. Metasoma as long as mesosoma, subovate, T1 1.1 × its width; exceeding middle of metasoma with two submedian carinae separated by deep fovea basally, outer pair of carinae longer than inner pair.

*Male*. Unknown.

##### Host.

Unknown.

##### Etymology.

Named after Vietnam, the country where the holotype was collected.

#### Hockeria

Taxon classificationAnimaliaHymenopteraChalcididae

Walker, 1834

**Figures 147–148.**
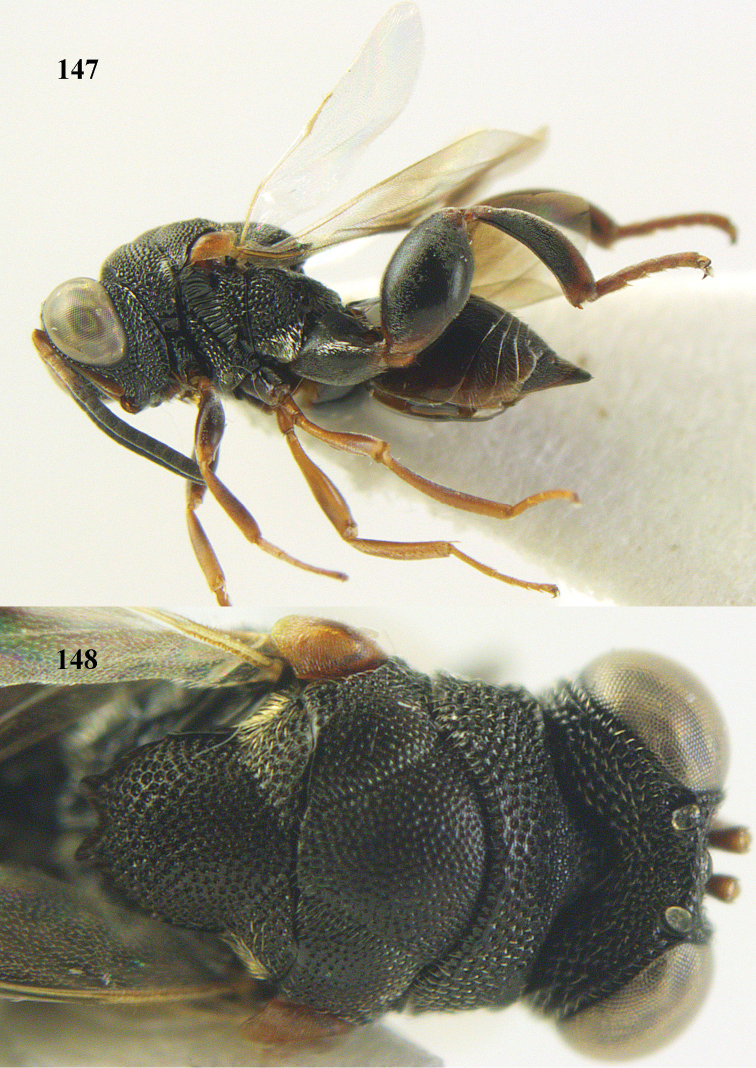
*Hockeria bangalorica* Narendran, ♀, Hua Phong Dien N. R. **147** habitus lateral **148** mesosoma dorsal.

**Figure 149.**
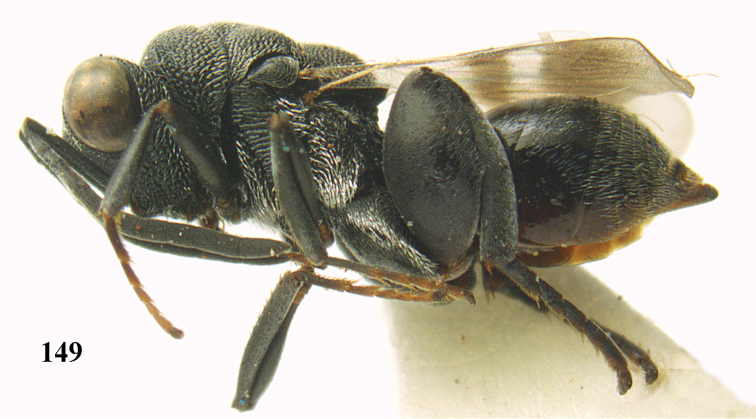
*Hockeria guptai* Narendran, ♀, Vu Quang N. P., habitus lateral.

**Figures 150–151.**
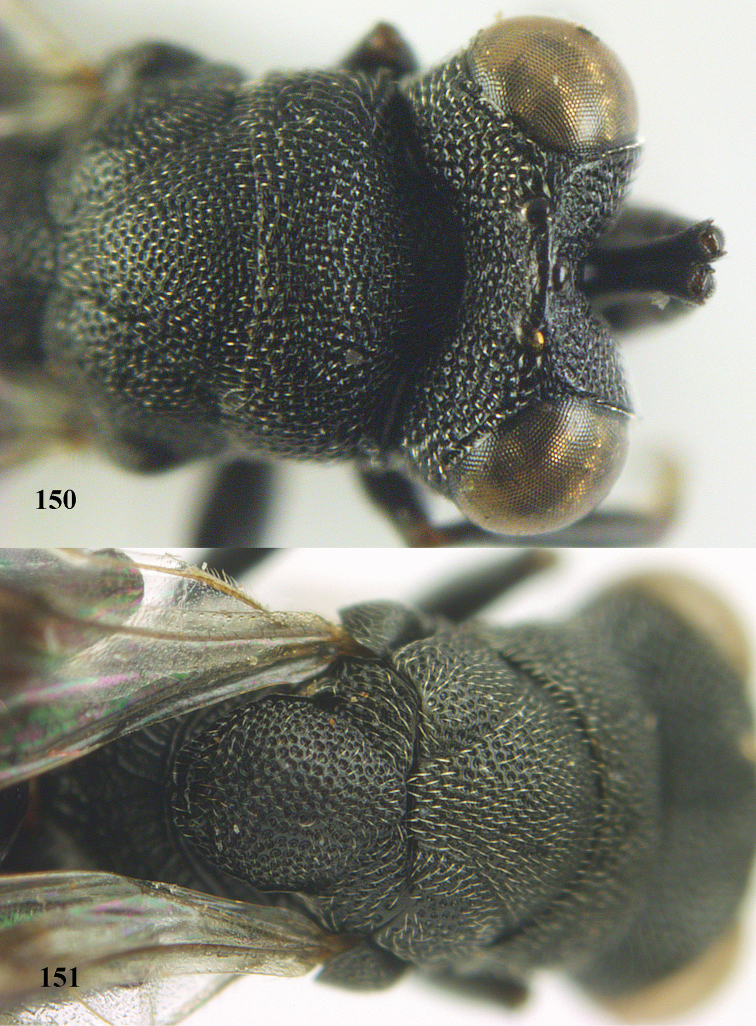
*Hockeria guptai* Narendran, ♀, Vu Quang N. P. **150** head dorsal **151** scutellum dorsal.

Figs 147–148 , 149 , 150–151

Hockeria Walker, 1834: 21, 34. Type species: Hockeria bifasciata Walker; indirectly proposed by Kerrich and Menon 1949.
Stomatoceras Kirby, 1883: 54, 62. Type species: Halticella liberator Walker, by original designation. (Synonymised with Hockeria Walker by Bouček 1988b).
Temnata Cameron, 1897: 42. Type species: Temnata maculipennis Cameron, by monotypy. (Synonymised with Hockeria Walker by Narendran 1989).
Centrochalcis Cameron, 1905: 230. Type species: Centrochalcis octodentata Cameron, by monotypy. (Synonymised with Hockeria Walker by Bouček 1988b).
Hypochalcis Girault, 1915b: 325. Type species: Chalcis modesta Masi, by original designation and monotypy. (Synonymised with Hockeria Walker by Bouček 1952).
Afrochalcis Schmitz, 1946: 115. Type species: Afrochalcis exiguus Schmitz, by monotypy. (Synonymised with Hockeria by Bouček 1988b).
Hockerella Girault, 1930: 4. Type species: Hockerella dioculata Girault, by original designation. (Synonymised with Hockeria by Bouček 1988b).
Afrhockeria Steffan, 1955: 381–382. Type species: Afrhockeria basilewskyi Steffan, by original designation and monotypy. (Synonymised with Hockeria by Bouček 1988b).
Nipponohockeria Habu, 1960: 234. Type species: Nipponohockeria ishiii Habu, by original designation and monotypy. (Synonymised with Hockeria by Narendran 1989).

##### Diagnosis.

This genus closely resembles *Antrocephalus* Kirby in general appearance, but differs from it in having the face without distinct pre-orbital carinae running behind anterior ocellus, not concave as in *Antrocephalus* and the pronotum without anterior carinae or tubercles. Only with experience in the taxonomy of this group one can differentiate these two genera.

##### Description.

Face usually without distinct pre-orbital carina, if pre-orbital carina present then the carina does not turn mesad behind anterior ocellus to join the pre-orbital carina of opposite side; scrobe usually not deep but shallow; pronotum never with anterior carinae and tubercles, metasoma similar to that of *Antrocephalus* and *Kriechbaumerella*; T1 with or without basal carinae.

##### Hosts.

Parasitic on pupae of Lepidoptera. One species was reared from Strepsiptera (Bouček 1988). Two New World species are parasitoids of Neuroptera and one species was reared from Hymenoptera (Diprionidae) (Bouček (in Bouček & Delvare) 1992).

##### Distribution.

Cosmopolitan.

##### Key to Vietnamese species of *Hockeria* Walker

**Table:** 

1	Two basal carinae of T1 present; PMV more than twice as long as STV; propodeum without dense micro-sculpture between carinae; hind femur black with base and apex yellowish brown	***Hockeria bangalorica* Narendran**
–	Two basal carinae of T1 absent; PMV at the most twice as long as STV; propodeum with dense micro-sculpture between carinae; hind femur completely black	***Hockeria guptai* Narendran**

#### Hockeria bangalorica

Taxon classificationAnimaliaHymenopteraChalcididae

Narendran, 1989

Figs 147–148

Hockeria bangalorica Narendran, 1989: 92 (♀, Bangalore, India (transferred from DZCU to QMB)).

##### Material

(RMNH, IEBR). 1 ♀, “C. **Vietnam**: Thu Thien Hué, Phong Dién N. R., n[e]ar base camp, 15 km W. Phong My, c. 150 m, 23.iii.–6.iv.2001, Malaise trap 2, C. v. Achterberg & R. de Vries, RMNH’01”; 1 ♀, “N. Vietnam: Ninh Binh, Cuc Phuong N. P., nr. entrance, c 225 m, 15.iv.–1.v.2000, Malaise trap 2, Mai Phu Quy, RMNH’00”; 1 ♂, “S. Vietnam: Dak Lak, Chu Yang Sin N. P., Kong K’Mar, Malaise traps 1–6, 550–610 m, 21–26.x.2005, C. v. Achterberg & R. de Vries, RMNH’05”.

##### Diagnosis.

In the key to Oriental species of *Hockeria* (Narendran 1989) *Hockeria bangalorica* comes near *Hockeria lankana* Narendran, but differs from it in having: 1) metasoma longer than mesosoma (in *Hockeria lankana* metasoma shorter than mesosoma); 2) pre- and post-orbital carinae present (in *Hockeria lankana* pre- and post-orbital carinae absent); and 3) apex of scutellum broadly emarginate and bi-dentate (in *Hockeria lankana* apex of scutellum less broadly emarginate and bi-lobed).

##### Description.

♀, length of body 2.9–5.5 mm.

*Colour*. Black; scape, pedicel, ring segment and F1 yellowish brown; fore and middle legs and tegula yellowish brown; hind femur yellowish brown basally and apically, middle part blackish brown or black; metasoma brown ventrally; fore wing with two brown infuscations with a medial hyaline spot.

*Head*. Head wider than mesosoma, scrobe not reaching front ocellus; pre-orbital carina distinct; post-orbital carina running upwards behind posterior margin of eye; malar ridge 0.4 × height of eye in profile; eye length in profile 0.8 × its height; geno-temporal furrow absent. POL 9.5 × OOL. Antenna (fig. 79 in Narendran 1989) with scape almost reaching anterior ocellus.

*Mesosoma*. Mesosoma with close pits, interstices narrower than diameter of a pit, rugose; apex of scutellum bi-lobed; scutellum somewhat flat.

*Wings*. Fore wing with PMV more than 2–3 × longer than STV, longer than MV.

*Legs*. Hind coxa with a week dorsal raised carina; hind femur weakly bi-lobed ventrally.

*Metasoma*. Metasoma longer than mesosoma, T1 with two basal carinae and a median fovea in between; carinae shorter than width between them.

*Male*. Resembles female, but with longer antennal segments and the fore wing has no infuscation.

##### Host.

Unknown.

##### Distribution.

Vietnam (new record), Malaysia, India (Narendran 1989).

##### Variation.

The Vietnamese specimens show the following variation from the type. The yellowish brown colour of scape, pedicel, ring segment and F1 tends to become brownish yellow; fore and middle coxae are black; hind tibia and tarsi brownish; POL 5 × OOL; scutellum moderately convex; dorsal carina of hind coxa stronger; basal carinae of T1 longer than space between them.

#### Hockeria guptai

Taxon classificationAnimaliaHymenopteraChalcididae

Narendran, 1989

Figs 149 , 150–151

Hockeria guptai Narendran, 1989: 109 (♀, holotype, India (CNC)).

##### Material.

1 ♀ (RMNH), “C. **Vietnam**: Ha Tinh, Vu Quang N. P., by hand[net], 1.x.2009, R. de Vries, RMNH’09”.

##### Diagnosis.

This species comes very near *Hockeria tristis* (Strand) because of the colour of the body and wings and in having micro-sculpture on the propodeum, but differs from *Hockeria tristis* in having: 1) T1 smooth and shiny (in *Hockeria tristis* T1 densely micro-sculptured); 2) mesopleuron not densely micro-sculptured as in *Hockeria tristis* and 3) apex of scutellum not as deeply emarginated as in *Hockeria tristis*.

##### Description

(based on specimen from Vietnam). ♀, length of body 4.8 mm.

*Colour*. Black with following parts as follows: eyes dull yellowish brown with dull brownish patches; ocelli pale reflecting yellow; fore and mid tarsi blackish brown; fore wing hyaline with 2 brown infuscations and a white patch adjacent to STV.

*Head*. Width of head in anterior view 1.2 × its height, distinctly wider than mesosoma (27:23); vertex and temples not narrow; POL 2.7 × OOL; AOL subequal to OOL; width between eyes 2.5 × POL; pre-orbital carina distinct, not distinctly joining malar sulcus, vertex with a carina -like ridge behind anterior ocellus (this ridge not clearly joining pre-orbital carinae to form a distinct horse-shoe like pre-orbital carina); height of malar sulcus 0.7 × height of eye in profile; eye height 1.3 × eye length in profile; post-orbital carina absent; geno-temporal furrow shallow; scrobe reaching anterior ocellus, cross reticulate-striate. Antennal scape reaching anterior ocellus. Relative lengths of antennal segments:scape = 57, pedicel = 15; ring segment = 7; F1 = 14; F2 = 15; F3 = 14; F4 = 13; F5 = 12.5; F6 = 11.5; F7 = 9; clava = 23.4.

*Mesosoma*. Mesosoma with close, umbilicate, setigerous pits, interstices carinate and rugose; posterior margin of pronotum arched posteriorly; length of middle lobe of mesoscutum a little shorter than scutellum (8:10); scutellum as long as wide, high in profile, apex bi-lobed. Propodeum with dense characteristic micro-sculpture on spaces between longitudinal carinae on sides; interstices densely micro-sculptured; postspiracular teeth well developed; callus with dense setae.

*Wings*. Fore wing 2.9 × longer than wide; relative length of SMV = 37; MV = 10; PMV = 4; STV = 2.

*Legs*. Hind coxa striate dorsally, densely pubescent ventrally; hind femur bi-lobed, 1.8 × as long as broad.

*Metasoma*. Metasoma subequal in length to mesosoma, 1.8 × as long as its width in dorsal view; T1 smooth and shiny; T2 smooth and shiny on dorso-median area, pubescent on dorso-lateral side; T6 densely rugose and weakly punctate; epipygium a little longer than ovipositor sheath in dorsal view.

*Male*. Unknown.

##### Host.

Unknown.

##### Distribution.

Vietnam (new record), India, Malaysia (Narendran 1989).

##### Variation.

Length of ♀ varies from 4.0–4.8 mm; height of malar sulcus distinctly longer than height of eye in Vietnamese specimen (according to the original description MS is shorter than height of eye in profile); POL 2.7–2.8 × OOL; median carina of propodeum not indicated in Vietnamese specimen; basal pit of T1 indistinct in Vietnamese specimen.

#### Kriechbaumerella

Taxon classificationAnimaliaHymenopteraChalcididae

Dalla Torre, 1897

Figs 152 , 153–154 , 155 , 156–157 , 158–159 , 160–161 , 162 , 163–164

Coleops Kriechbaumer, 1894b: 316–317. Type species (by monotypy): Coleops palpebratus Kriechbaumer, 1894. Preoccupied by Coleops Blyth, 1849.
Kriechbaumerella Dalla Torre, 1897: 84. Replacement name for Coleops Kriechbaumer.
Eucepsis Steffan, 1953: 8, 12. Type species (by original designation): Stomatoceras magrettii Kirby, 1886. Synonymised with Kriechbaumerella Dalla Torre by Narendran (1984).

##### Diagnosis.

This genus is similar to the genera *Antrocephalus* and *Hockeria*, but differs mainly in having the ventral margin of the teeth of the hind femur three-lobed or typically wavy and in *Hockeria* the frontal carina is not horseshoe-shaped.

##### Description.

Face with prominent horseshoe-shaped carina (as in *Antrocephalus*) running behind anterior ocellus; pronotum with carinae restricted to lateral third, medially not turning backwards or projecting as tubercles; apex of scutellum usually bi-dentate; ventral margin of wide hind femur with ventral margin of teeth three lobed; PMV always longer than STV; metasoma sessile.

##### Hosts.

Parasitoids of pupae of Lepidoptera.

##### Distribution.

Asia, Europe and Africa.

##### Key to Vietnamese species of the genus *Kriechbaumerella* Dalla Torre (based on females)

**Table:** 

1	Scutellum distinctly bi-dentate posteriorly (Fig. 164) and V-shaped incised medio-posteriorly; antenna long (Fig. 163)	***Kriechbaumerella nepalensis* Narendran**
–	Scutellum truncate posteriorly (Figs 157, 161) or slightly incised; antenna shorter (Figs 155, 158)	**2**
2	Postero-laterally T1 punctate and medio-dorsally granulate; T2–6 coarsely punctate (Fig. 159); with strong smooth carina parallel to dorsal margin of clypeus (Fig. 160)	***Kriechbaumerella destructor* (Waterston)**
–	Postero-laterally and medio-dorsally T1 smooth or only posterior fifth finely punctate; T2–6 finely punctate (Fig. 155); with weak sculptured carina parallel to dorsal margin of clypeus	**3**
3	Posterior fifth of T1 superficially finely punctate; epipygium longer than median length of T6; posterior lamella of scutellum subtruncate, only slightly emarginate and with pair of lateral carinae (Fig. 154)	***Kriechbaumerella ayyari* (Gahan)**
–	Posterior fifth of T1 smooth; epipygium shorter than median length of T6; posterior lamella of scutellum evenly convex and without pair of carinae (Fig. 157)	***Kriechbaumerella cordigaster* Roy & Farooqi**

**Figure 152.**
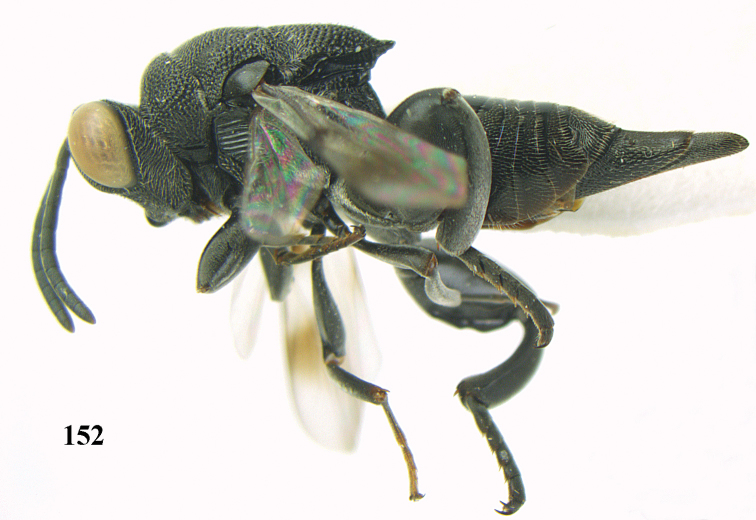
*Kriechbaumerella ayyari* (Gahan), ♀, Núi Chúa N. P., habitus lateral.

**Figures 153–154.**
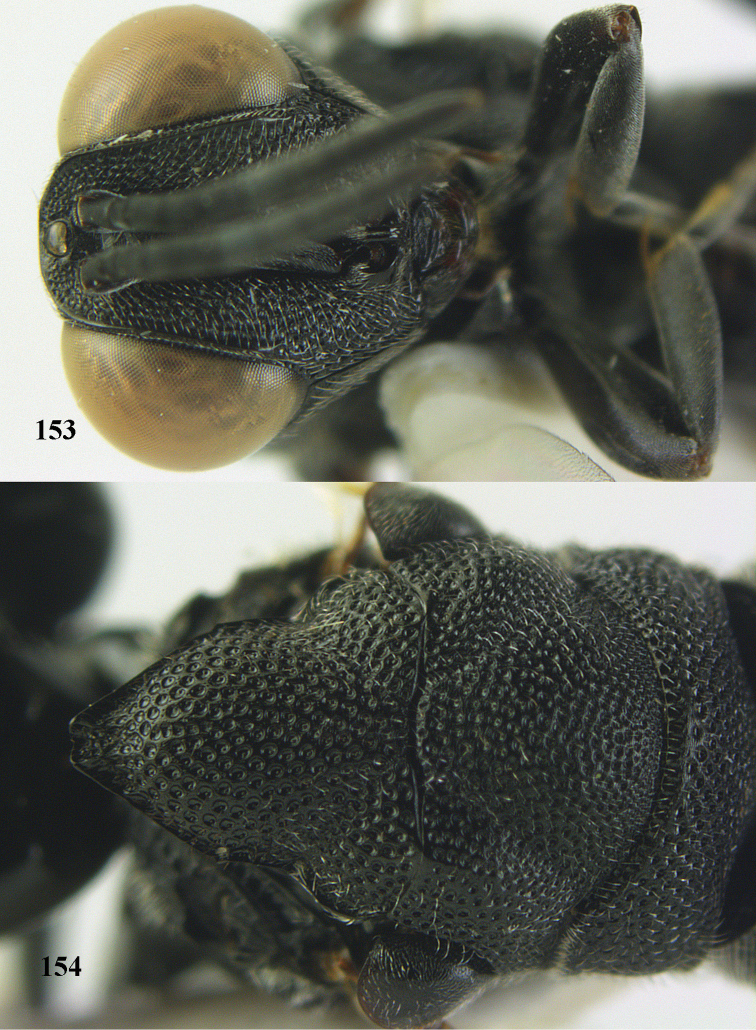
*Kriechbaumerella ayyari* (Gahan), ♀, Núi Chúa N. P. **153** head anterior **154** mesosoma dorsal.

**Figures 155.**
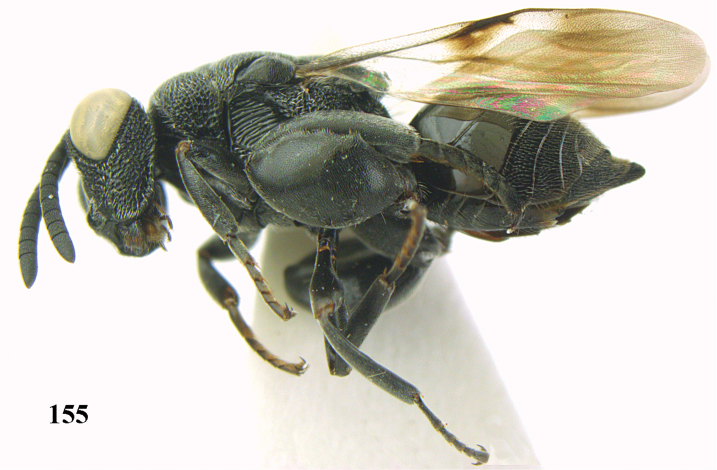
*Kriechbaumerella cordigaster* (Roy & Farooqi), ♀, Chu Yang Sin N. P., habitus lateral.

**Figures 156–157.**
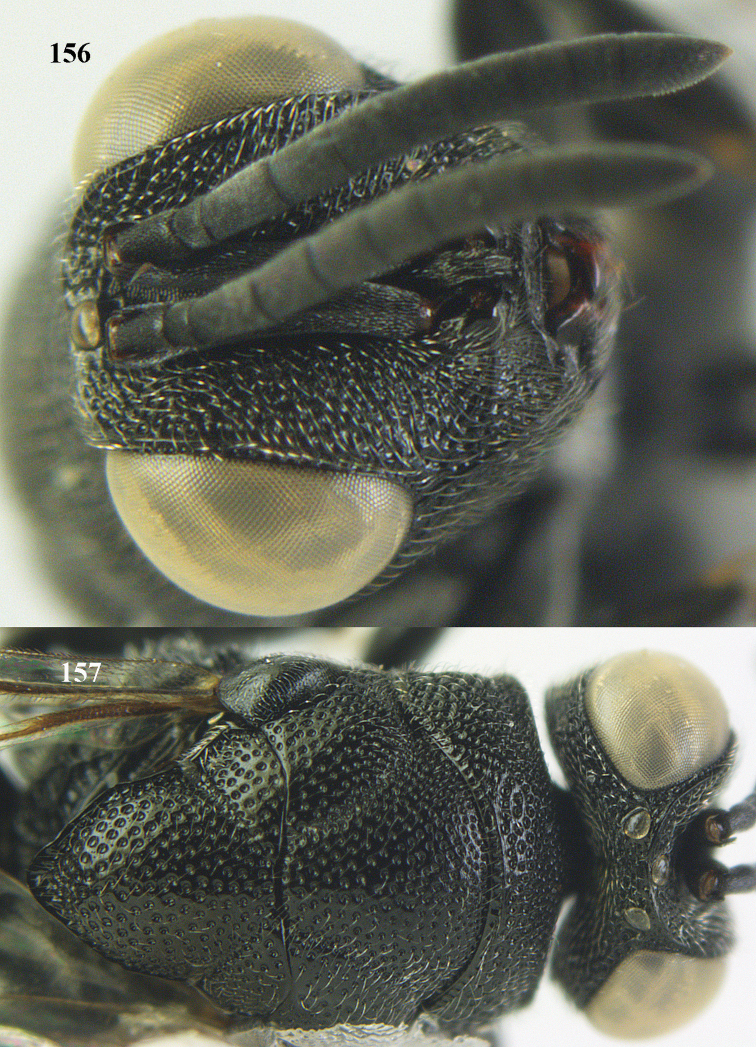
*Kriechbaumerella cordigaster* (Roy & Farooqi), ♀, Chu Yang Sin N. P. **156** head anterior **157** head and mesosoma dorsal.

**Figures 158–159.**
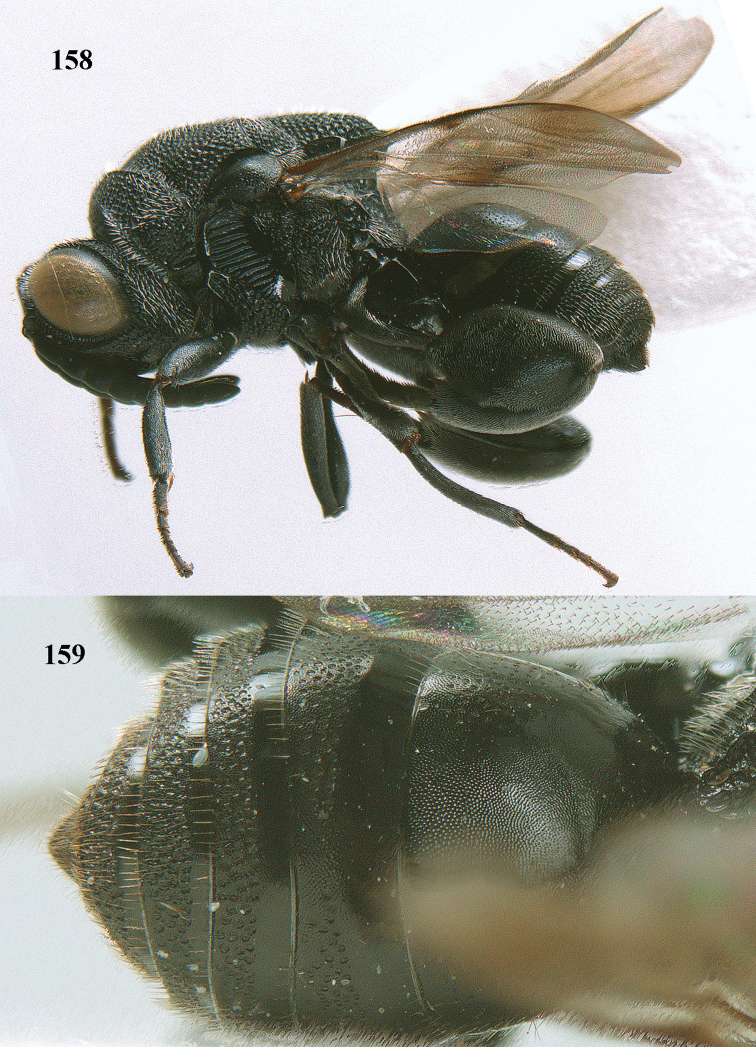
*Kriechbaumerella destructor* Waterston, ♀, Cát Tiên N. P. **158** habitus lateral **159** metasoma dorsal.

**Figures 160–161.**
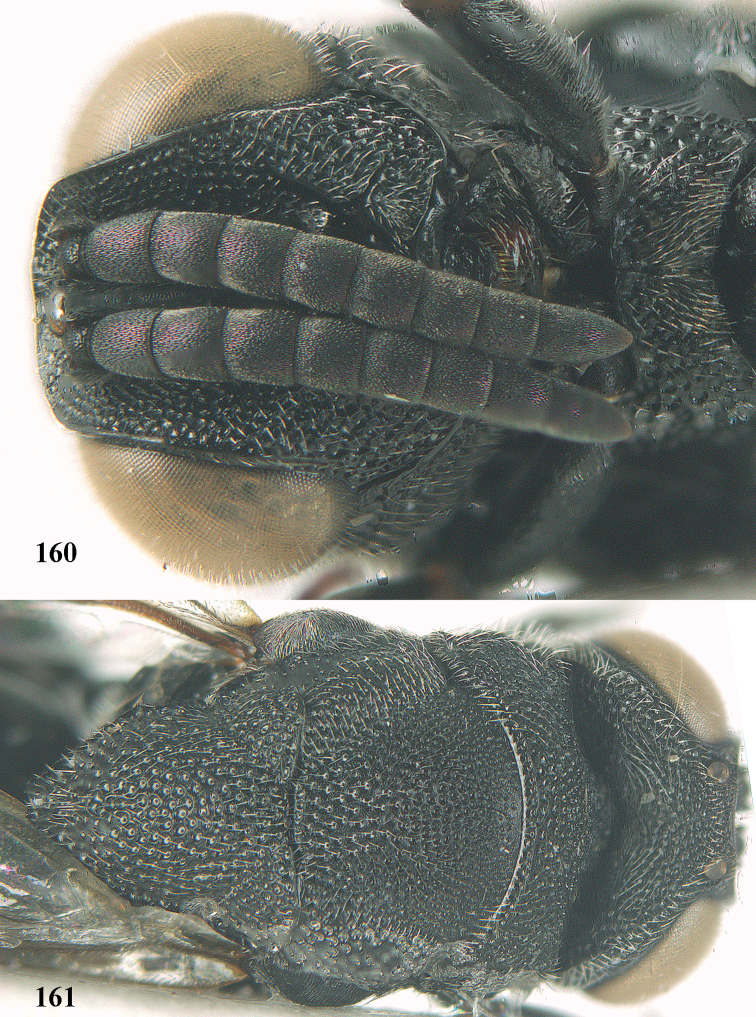
*Kriechbaumerella destructor* Waterston, ♀, Cát Tiên N. P. **160** head anterior **161** head and mesosoma dorsal.

**Figures 162.**
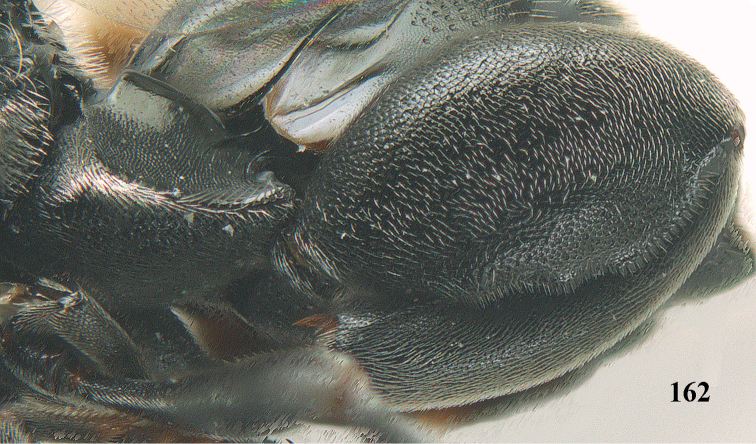
*Kriechbaumerella destructor* Waterston.

**Figures 163–164.**
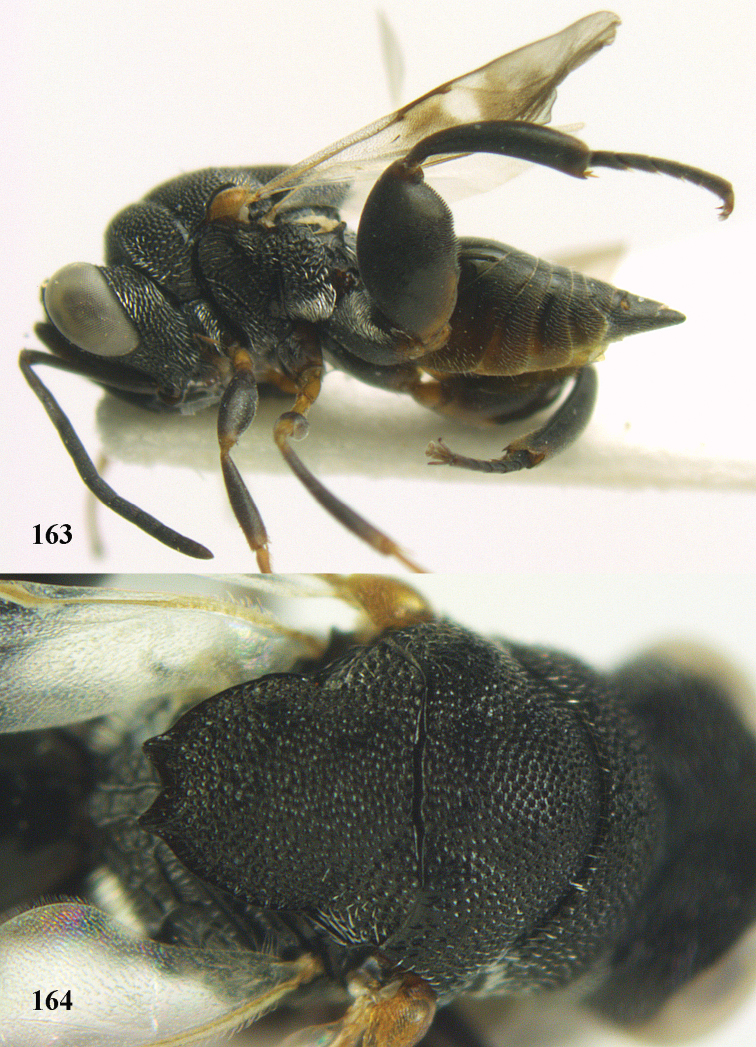
*Kriechbaumerella nepalensis* Narendran, ♀, Núi Chúa N. P. **163** habitus lateral **164** scutellum dorsal.

#### Kriechbaumerella ayyari

Taxon classificationAnimaliaHymenopteraChalcididae

(Gahan, 1919)

Figs 152 , 153–154

Stomatoceras ayyari Gahan, 1919: 518 (♀, lectotype India (Coimbatore), USNM; examined).
Hockeria ayyari ; Narendran 1986: 86.
Kriechbaumerella ayyari ; Cock et al. 1987: 166.
Antrocephalus indicatus Hussain & Agarwal, 1982a: 333 (♂, holotype India (Madhyapradesh), ZDAMU; examined; synonymised with Kriechbaumerella ayyari (Gahan) by Narendran (1989)).

##### Material.

1 ♀ (RMNH), “**Vietnam**: Ninh Thuân, Núi Chúa N. P., northeast part, Mal[aise] traps, 90–150 m, 23–30.v.2007, C. v. Achterberg & R. de Vries, RMNH’07”.

##### Diagnosis.

This species comes near *Kriechbaumerella mansues* (Nikol’skaya) in the key to species of Oriental *Kriechbaumerella* by Narendran (1989), but differs from *Kriechbaumerella mansues* in having: 1) interstices of pits on mesonotum narrower than diameter of pits and nearly rugose (in *Kriechbaumerella mansues* interstices of pits of mesonotum broader, smooth and shiny); 2) hind femur black (in *Kriechbaumerella mansues* hind femur mostly or entirely red), and 3) T6 shallowly pitted and pits rugoso-punctate (in *Kriechbaumerella mansues* T6 with pits deeper and not rugose).

##### Description.

♀, length of body 8.0–8.5 mm.

*Colour*. Black; tip of antennae, fore tarsi, fore femur and mid tarsi black with liver brown tinge; fore wing with deep blackish brown infuscations adjoining MV; POL 3.5–5.0 × OOL.

*Head*. Antenna with scape reaching front ocellus; pre-orbital carinae distinct; post-orbital carina weakly represented, running upwards; geno-temporal furrow absent; scrobe reaching anterior ocellus.

*Mesosoma*. Apex of scutellum well produced posteriorly with a median emargination.

*Wings*. Fore wing with PMV 1.2–1.5 × length of MV. Metasoma with T1 micro-sculptured dorsally (at least partly); posterior margin of T1 straight; T2 to T6 with larger pits on dorso-lateral parts; T6 rugoso-punctate; epipygium carinate at middle, longer than median length of T6; ovipositor sheath subequal in length to epipygium or distinctly shorter than epipygium in dorsal view.

*Male*. Unknown.

##### Host.

*Latoia lepida* (Cramer) (Lepidoptera: Limacodidae).

##### Distribution.

India, Vietnam (new record for Vietnam).

##### Variation.

In the Vietnamese specimen the antenna, fore femur and middle tarsus are without liver brown tinge and the fore wing has two large dark brown patches, one adjoining MV and the other beyond PMV.

#### Kriechbaumerella cordigaster

Taxon classificationAnimaliaHymenopteraChalcididae

(Roy & Farooqi, 1984)

Figs 155 , 156–157

Eucepsis cordigaster Roy & Farooqi, 1984: 19 (♀, holotype India (Shillong), (INPC); examined).
Kriechbaumerella cordigaster ; Narendran 1989: 70.

##### Material.

1 ♀ (RMNH), “S. **Vietnam**: Dak Lak, Chu Yang Sin N. P., n[ea]r river, c. 740 m, Malaise traps, 1–10.vi.2007, C. v. Achterberg & R. de Vries, RMNH’07.

##### Diagnosis.

*Kriechbaumerella cordigaster* comes near *Kriechbaumerella gibsoni* Narendran in the key to Oriental species by Narendran (1989), but differs from *Kriechbaumerella gibsoni* in having: 1) fore wing with two brown infuscations (in *Kriechbaumerella gibsoni* fore wing without infuscations); 2) T1 not micro-sculptured dorsally (in *Kriechbaumerella gibsoni* T1 faintly micro-sculptured on posterior half), and 3) metasoma not subglobose (in *Kriechbaumerella gibsoni* metasoma subglobose).

##### Description.

♀, length of body 5.8–6.8 mm.

*Colour*. Black; fore wing with brown veins and brown infuscations near MV and beyond PMV.

*Head*. Pre-orbital carinae present; post-orbital carinae weakly represented; geno-temporal furrow absent.

*Mesosoma*. Apex of scutellum bi-dentate, teeth turned upwards (visible when viewed from posterior side).

*Metasoma*. Metasoma a little longer or subequal in length to or shorter than mesosoma; T1 smooth with a pit at base; epipygium shorter than T6.

*Male*. Similar to ♀, but scape swollen with a deep groove ventrally.

##### Host.

Unidentified pupa of Lepidoptera.

##### Distribution.

India, Vietnam (new record).

##### Variation.

The metasoma is shorter than the mesosoma in the Vietnamese female, whereas in Indian females the metasoma is longer than the mesosoma, and the antenna and the metasoma have a slight reddish tinge.

#### Kriechbaumerella destructor

Taxon classificationAnimaliaHymenopteraChalcididae

(Waterston, 1922)

Figs 158–159 , 160–161 , 162

Antrocephalus destructor Waterston, 1922: 14 (♀, lectotype, India, (BMNH) examined).
Kriechbaumerella destructor ; Narendran 1989: 66.
Eucepsis longigaster Roy & Farooqi, 1984: 21 (♀, holotype, India (IARI) examined). Synonymised by Narendran (1989).

##### Material.

1 ♀ (RMNH), “S. **Vietnam**: Dóng Nai, Cát Tiên N. P., Dong trail, Mal. traps, c. 100 m, 19–25.iv.2007, Mai Phu Quy & Nguyen Tanh Manh, RMNH’07”.

##### Host.

Parasitoid of *Hypsipyla robusta* (Moore) (Pyralidae) on *Cedrela toona* Roxb.

##### Distribution.

Vietnam (new record), India.

#### Kriechbaumerella nepalensis

Taxon classificationAnimaliaHymenopteraChalcididae

Narendran, 1989

Figs 163–164

Kriechbaumerella nepalensis Narendran, 1989: 72–73 (♀, lectotype Nepal, (CNC) examined).

##### Material.

1 ♀ (RMNH), “**Vietnam**: Ninh Thuân, Núi Chúa N. P., dry south part, Mal[aise] traps, 100–180 m. 22–29.v.2007, C. v. Achterberg & R. de Vries, RMNH’07”.

##### Diagnosis.

Easily recognizable by the combination of the long antenna of ♀ (Fig. 163) and the medio-posteriorly distinctly V-shaped incised scutellum (Fig. 164).

##### Description.

♀, length of body 7.4 mm.

*Colour*. Black; trochanters, fore and middle tarsi, fore and middle femora (but basally and apically brownish yellow) black; fore wing infuscate near MV but with hyaline patch antero-medially (Fig. 163).

*Head*. POL 5 × OOL; antenna with scape reaching level of front ocellus; pre-orbital carinae distinct; post-orbital carina absent; geno-temporal furrow absent; scrobe including anterior ocellus.

*Mesosoma*. Apex of scutellum well produced posteriorly with a medial V-shaped emargination.

*Wings*. Fore wing with sclerotised part of PMV 0.3 × length of MV.

*Metasoma*. T1 smooth and strongly shiny, medio-basally impressed, but without pair of dorsal carinae; posterior margin of T1 straight; T2–6 largely finely granulate; T6 with superficial rugulosity; epipygium keeled medially, about as long as median length of T6; ovipositor sheath subequal in length to epipygium.

*Male*. Unknown.

##### Host.

Unknown.

##### Distribution.

India, Nepal, Vietnam (new record for Vietnam).

#### Megachalcis

Taxon classificationAnimaliaHymenopteraChalcididae

Cameron, 1903

Figs 165–167 , 168

Megachalcis Cameron, 1903: 96–97. Type-species: Megachalcis fumipennis Cameron, 1903, by monotypy.
Allocentrus Cameron, 1911: 12. Type species: Allocentrus hirticeps Cameron, 1911, by monotypy. Synonymised with Megachalcis Cameron by Narendran (1984).
Macrochalcis Masi, 1944: 137. Type species: Macrochalcis bischoffi Masi, 1944, by original designation. Synonymised with Megachalcis Cameron by Narendran (1984).

##### Diagnosis.

This genus resembles the genus *Cratocentrus* Cameron in having PMV much longer than MV, T2 to T4 strongly reduced and mostly hidden under the large T1 and in having a long ovipositor sheath. However, it differs from *Cratocentrus* in having: 1) scapulae and axillae prominently convex (in *Cratocentrus* scapulae and axillae less convex), and 2) vertex with median raised teeth absent (in *Cratocentrus* vertex with a pair of raised teeth).

##### Description.

Body longer than 6 mm; MV much longer than PMV (twice or more); mesonotum with often rasp-like structure; scapulae and axillae prominently convex; propodeal spiracle elongate in oblique subhorizontal direction; body with some silvery patches of setae; petiole very short, mostly concealed in dorsal view; T2 to T4 strongly reduced and for most part hidden under the large T1. Metasoma of ♀ with long ovipositor sheath.

##### Hosts.

Apparently parasitoid of wood-boring beetles (Bouček 1988b).

##### Distribution.

Oriental region.

##### Key to Vietnamese species of *Megachalcis* Cameron (based on females)

**Table:** 

1	Hind femur with an inner basal tooth present; wings with a little golden tinge, not infuscated basally; head without small humps in POL area	***Megachalcis vietnamensis* sp. n.**
–	Hind femur without inner basal tooth; wings without golden tinge, basally more infuscated than apically; head with two small humps in POL area	***Megachalcis carinata* Steffan**

**Figures 165–167.**
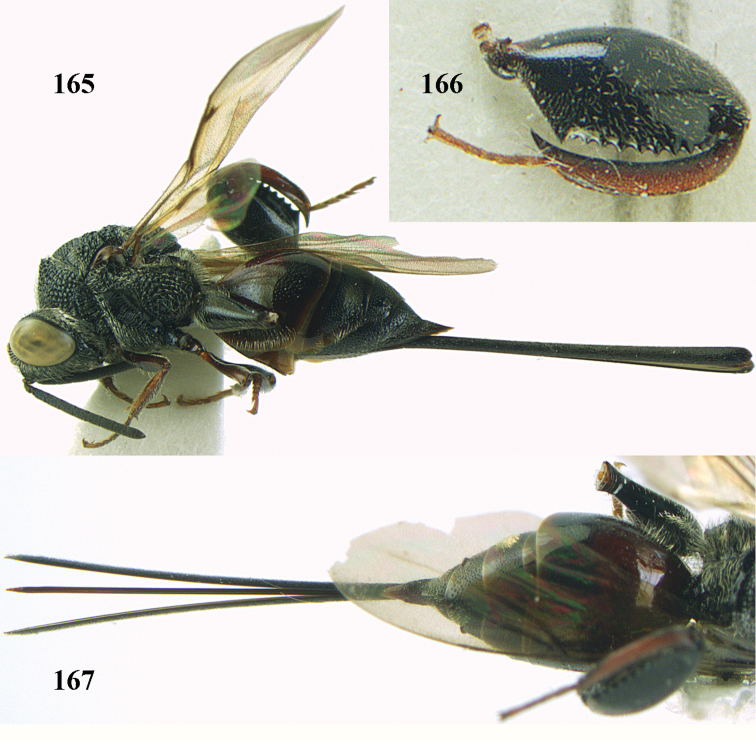
*Megachalcis vietnamensis* sp. n., ♀, holotype. **165** habitus lateral **166** hind femur and tarsus lateral **167** metasoma dorsal.

**Figure 168.**
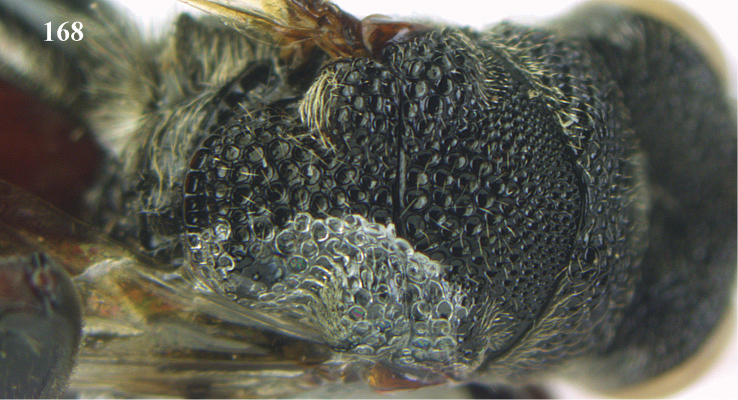
*Megachalcis vietnamensis* sp. n., ♀, holotype, scutellum dorsal.

#### Megachalcis carinata

Taxon classificationAnimaliaHymenopteraChalcididae

Steffan, 1951

Megachalcis carinata Steffan, 1951c: 599 (♀, Vietnam (MNHN)).

##### Diagnosis.

This species comes near *Megachalcis hirticeps* Cameron in the key to species by Narendran (1989), but differs from it in having: 1) fore wing more infuscate at base than at apex (in *Megachalcis hirticeps* fore wing uniformly infuscate); 2) pronotal humps well developed (in *Megachalcis hirticeps* pronotal humps not well developed); 3) distal tooth of propodeum more robust than proximal one (in *Megachalcis hirticeps* proximal tooth of propodeum more prominent than distal dent); 4) hind tibia shiny and polished (in *Megachalcis hirticeps* hind tibia matt), and 5) hollow depression of hind tibia with sparse minute separate pits (in *Megachalcis hirticeps* hollow depression of hind tibia with contiguous pits).

##### Description.

(based on the description by Steffan, 1951c).

*Head*. Head with two relatively small humps in POL; eye height in profile 2.4 × height of malar space in profile, eye length in profile about 0.7 × its height (7:12); antennal length 2.5 × eye height; clava shorter than scape (5:7).

*Mesosoma*. Pronotum with two well developed humps; mesosoma with apex of scutellum projecting angularly; basal areoles of propodeum not of uniform size; distal tooth of propodeum stronger than proximal tooth.

*Wings*. Fore wing more infuscated at base than at apex; fore wing 2.8 × as long as wide.

*Legs*. Hind coxa shorter than hind femur (23:24) without a tooth; hind femur twice as long as wide, with a row of 12–13 irregular teeth on outer ventral margin; hind tibia shiny with hollow depression containing sparse minute pits.

*Metasoma*. Metasoma (excluding ovipositor sheath) longer than mesosoma (50:31); exserted part of ovipositor sheath 1.3 × length of metasoma, and 2.4 × length of hind femur.

*Male*. Unknown.

##### Host.

Unknown.

##### Distribution.

Vietnam.

#### Megachalcis vietnamensis sp. n.

Taxon classificationAnimaliaHymenopteraChalcididae

http://zoobank.org/D1A20450-06E4-482C-B4FD-348A8EAA37C1

Figs 165–167 , 168

##### Type material.

Holotype (RMNH), ♀, “S. **Vietnam**: Dóng Nai, Cát Tiên N. P., *Ficus* trail, Mal. traps, c 100 m, 9–30.iv.2007, M.P. Quy & N.T. Manh, RMNH’07”.

##### Diagnosis.

In the key to Oriental species of *Megachalcis* (Narendran 1989) this species comes near *Megachalcis malabarica* Narendran in having an inner basal tooth at the hind femur. However, the new species differs from *Megachalcis malabarica* in having: 1) distance between apex of epipygium to tip of ovipositor sheath (in dorsal view) longer than metasoma (56:44) (in *Megachalcis malabarica* distance between apex of epipygium to tip of ovipositor sheath shorter than metasoma); 2) pronotum with two submedian humps (in *Megachalcis malabarica* pronotum without humps); 3) hind coxa with a dorsal tooth (in *Megachalcis malabarica* hind coxa without a dorsal tooth), and 4) between apex of scrobe and anterior ocellus without a triangular pubescent area (in *Megachalcis malabrica* with a triangular pubescent area between anterior ocellus and apex of scrobe).

##### Description.

Holotype, ♀, length of body 14.5 mm (including 5.1 mm length of ovipositor sheath).

*Colour*. Black with following parts as follows: eyes grayish yellow; tarsi liver brown; mid coxa with a reddish brown tint on some parts; mid tibia basally and apically slightly yellowish brown; hind tibia reddish brown with margins black; apical margin of T1 and sternites pale brown; wings hyaline with a little golden brown tinge; veins dark brown; pubescence silvery.

*Head*. Head densely pubescent; width in anterior view 1.4 × its height; width in dorsal view 2.4 × its length; POL 1.6 × OOL; AOL equal to OOL; LOL equal to AOL; width between eyes in dorsal view 3 × POL; occiput concave; head completely pitted with large close pits, interstices carinate; scrobe reaching anterior ocellus; MS carinate; malar space 0.4 × eye height in profile; eye length 0.7 × eye height in profile; gena densely pubescent; eyes bare; pre-orbital carina absent; post-orbital carina starting from MS running upwards, not reaching geno-temporal margin; antenna inserted at level of ventral margin of eyes; scape not reaching anterior ocellus. Relative L:W of antennal segments:scape 18:4; pedicel = 4:3; anellus = 1:3; F1 to F6 equal length and width (5:4); F7 = 4:4; clava = 8:4; scape densely setose ventrally.

*Mesosoma*. Pronotum with close, deep pits; interstices carinate, two distinct humps present submedially on posterior marginal area; posterior margin of pronotum a little emarginated medially; mesoscutum and scutellum closely pitted, interstices carinate; apex of scutellum straight or entire. Propodeum with large foveolae, plicae raised, carinate; spiracle bean-shaped; a distinct tooth on either side projecting to lateral side; propodeum with dense silvery large setae on either side, spread over to median part.

*Wings*. Fore wing 3.3 × as long as wide; relative length of CC = 24; SMV = 21; MV = 7; PMV = 14; STV = 3.

*Legs*. Hind coxa with a dorso-basal tooth; hind femur twice as long as broad, ventral margin with a row of 11 teeth of different size; inner side of hind femur with a ventro-basal tooth; hind tibia with a deep smooth lengthy fovea from middle to apex on inner side.

*Metasoma*. Metasoma (excluding ovipositor sheath) distinctly longer than mesosoma (44:31); ovipositor sheath distinctly longer than metasoma (56:44).

*Male*. Unknown.

##### Host.

Unknown.

##### Distribution.

Vietnam.

#### Notaspidium

Taxon classificationAnimaliaHymenopteraChalcididae

Dalla Torre, 1897

**Figures 169–170.**
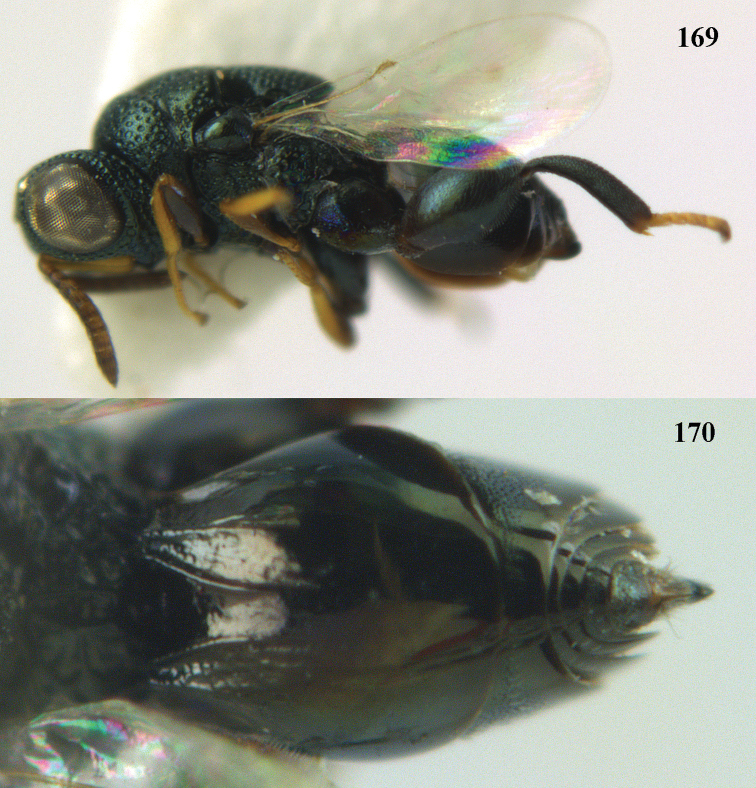
*Notaspidium vietnamicum* sp. n., ♀, holotype. **169** habitus lateral **170** metasoma dorsal.

**Figures 171–172.**
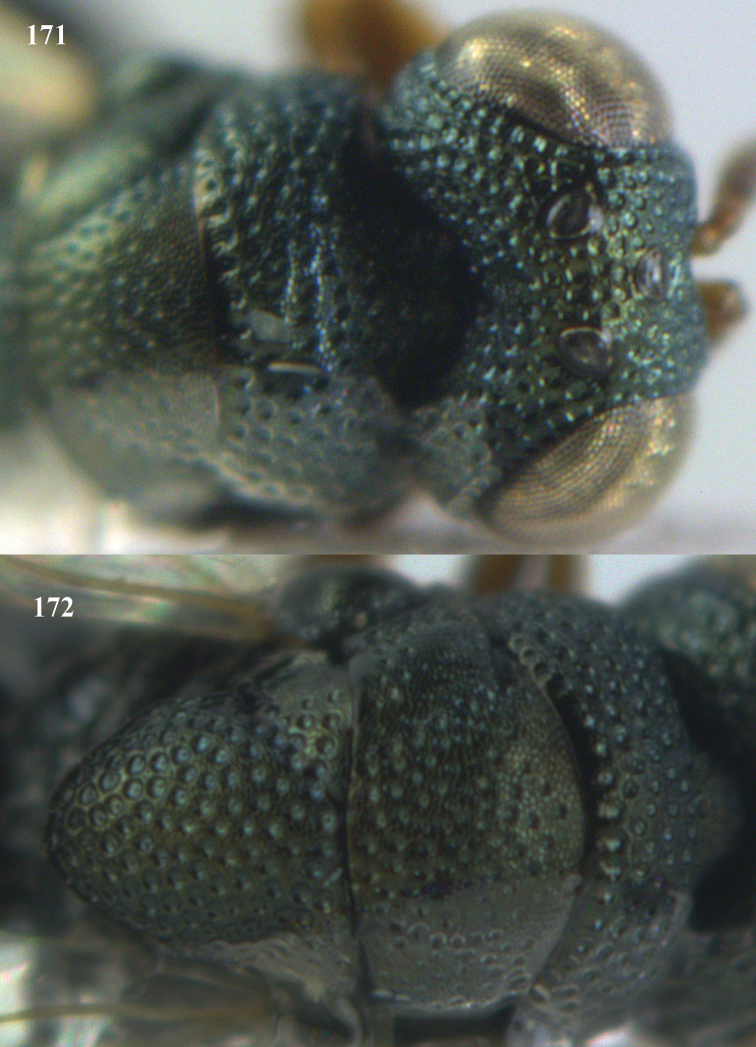
*Notaspidium vietnamicum* sp. n., ♀, holotype. **171** head dorsal **172** mesosoma dorsal.

Figs 169–170 , 171–172

Notaspis Walker, 1834: 21, 37; 1871: 37. Type species: Notaspis formiciformis Walker, by monotypy. Preoccupied by Notaspis Herman, 1804.
Notaspidium Dalla Torre, 1897: 87. Replacement name for Notaspis Walker.

##### Diagnosis.

Belongs to the group with MV short and distinctly removed from anterior wing margin (Fig. 169), PMV absent and STV usually rudimentary. *Notaspidium* differs from *Psilochalcis* by having at least two anteriorly united carinae on T1 (absent in *Psilochalcis*) and the scutellum arcuate, angulate or protruding apically (broadly rounded or truncated in *Psilochalcis*).

##### Description.

Both sexes, length of body 1.5–2.7 mm. Body with bright to dull metallic refringence; flagellum tapering proximally, with two or three segments transverse; metasoma sessile; T1 dorsally more or less flattened and with at least some longitudinal carinae anteriorly united by a cross elevation or carina and mostly with angulate corners. Scutellum often posteriorly produced (rarely hardly so), sometimes into an angulate spine or horn, at least slightly protruding over metanotum; hind femur swollen with a basal large tooth followed by a comb of ventral teeth on outer margin.

##### Hosts.

*Apanteles* spp. (Braconidae) through *Erisimus* spp. (Olethreutidae); *Pheidole titanis* (Wheeler) and Scolytidae (Coleoptera) (Bouček 1992).

##### Distribution.

India, Thailand, Vietnam (new report), Philippines, New Guinea and South America.

##### Key to Oriental species of *Notaspidium* Dalla Torre

**Table:** 

1	T1 with 2 long submedian basal carinae (carinae reaching 0.6 length of T1) with 2 or 3 parallel carinulae at distal part of submedian carinae; [apex of scutellum angulate, somewhat pointed (fig. 12 of Narendran 1987); antennae, fore and mid legs pale brownish; India]	***Notaspidium grisselli* Narendran**
–	T1 with more than one pair of basal carinae	**2**
2	Apex of scutellum angulate (fig. 170 of Narendran 1989) and pointed; scape 0.5 × length of pedicel plus flagellum; [T1 with 4 submedian basal carinae and a few distal broken carinulae beyond the tips of basal carinae; Philippines]	***Notaspidium bakeri* Narendran**
–	Apex of scutellum broadly arcuate posteriorly; scape 0.6 × pedicel plus flagellum or longer	**3**
3	Body without metallic refringence; metasoma a little longer than mesosoma; inner pair of submedian carinae slightly diverging on T1; posterior corners of propodeum tooth-like; [Thailand]	***Notaspidium thailandicum*** Narendran & Konishy
–	Body dull metallic green (Fig. 169); metasoma a little shorter than mesosoma; inner pair of submedian carinae of T1 converging a little posteriorly (Fig. 170); posterior corners of propodeum not with tooth-like projection (Fig. 170); [Vietnam]	***Notaspidium vietnamicum* sp. n.**

#### Notaspidium vietnamicum sp. n.

Taxon classificationAnimaliaHymenopteraChalcididae

http://zoobank.org/B1C6A18B-A7C0-44AB-87BF-34B6989B1ABA

Figs 169–170 , 171–172

##### Type material.

Holotype (RMNH), ♀, “**Vietnam**: Ninh Thuân, Núi Chúa N. P., dry south part, Mal. traps, 100–180 m, 22–29.v.2007, C. v. Achterberg & R. de Vries, RMNH’07”.

##### Diagnosis.

This new species comes near *Notaspidium thailandicum* Narendran & Konishy (Konishy et al. 2004) because of the arcuate apex of scutellum, and scape, fore and mid tibiae pale yellow or pale brownish yellow. However, this new species differs from *Notaspidium thailandicum* in having: 1) body dull metallic green (in *Notaspidium thailandicum* body black without metallic refringence); 2) posterior corners of propodeum without tooth-like projection (in *Notaspidium thailandicum* posterior corners of propodeum with tooth-like projection); 3) metasoma shorter than mesosoma (in *Notaspidium thailandicum* metasoma a little longer than mesosoma); 4) T1 with 3 pairs of strong carinae from base (in *Notaspidium thailandicum* T1 with 2 pairs of carinae from base), and 5) inner pair of submedian carinae converging posteriorly (in *Notaspidium thailandicum* inner pair of submedian carinae slightly diverging posteriorly).

##### Description.

Holotype, ♀, length of body 2.0 mm.

*Colour*. Dull metallic green with following parts as follows: scape pale yellow; pedicel and annellus pale brownish yellow; remaining segments of antenna dark brown; all coxae concolourous with metasoma; fore and mid femora dull brownish with slight green refringence on outer side; inner side pale yellow; fore and mid tibiae and all tarsi pale yellow; hind femur and tibia black with dull green refringence; wings hyaline with SMV pale hyaline yellow; MV and STV pale hyaline yellowish brown.

*Head*. Head densely punctate with micro-sculptured median area on occiput; pre- and post-orbital carinae present; post-orbital carina running upwards towards vertex and weakly joining the pre-orbital carina through ocell-ocular space; scrobe cross reticulate, its margins not carinate, separated from anterior ocellus by almost diameter of anterior ocellus; malar space with deep, close pits, malar ridge or malar sulcus absent; relative measurements: width of head (in anterior view) = 35; height = 30; width of fronto-vertex = 16; height of malar space (in lateral view) = 8; eye height in lateral view = 20; eye length in lateral view = 15; POL = 5; OOL = 2; AOL = 4; scape = 16; flagellum plus pedicel = 26; antenna short, clava acuminate; funicular segments transverse.

*Mesosoma*. Pronotum with closely set pits, interstices as broad as diameter of a pit in many places and half as broad as diameter of a pit in remaining places; interstices reticulate. Mesoscutum distinctly reticulate with scattered shallow pits on area adjoining anterior margin, remaining parts with close pits with interstices as broad as or half as broad as diameter of a pit; on scapula interstices more than diameter of a pit in many regions. Scutellum subequal in length to mesoscutum, with close pits, interstices narrower than diameter of a pit, reticulate; apex of scutellum broadly arcuate and angulate posteriorly, slightly produced over base of propodeum in dorsal view. Propodeum with submedian carinae enclosing smooth areola; other regions of propodeum areolate contain irregular sculpture or pits; posterior corners of propodeum not sharp; postspiracular tooth weakly indicated in the form of a short knob. Lateral panel of pronotum micro-reticulate and with a median raised cross ridge; mesosternum closely pitted; mesepimeron cross carinate; metapleuron closely pitted.

*Wings*. Fore wing 2.4 × as long as broad, pilosity almost absent except a few scattered ones at distal admarginal area.

*Legs*. Hind coxa smooth and shiny on dorsal part with scattered sparse pits on ventral side; hind femur twice as long as broad, ventrally with a broad tooth just before middle (0.42), outer surface finely reticulate, sparse setigerous punctures hardly traceable; hind tibia with a longitudinal band of closely spaced small pits dorsally.

*Metasoma*. Metasoma slightly shorter than mesosoma (19:21), 0.6 × as broad as its length; T1 longest, exceeding middle of metasoma, with 3 pairs of submedian carinae and a lateral carina on either side of outer submedian carina; lateral carina separated from outer submedian carina by a deep fovea; outer submedian carina and inner submedian carina separated by fovea; inner submedian carinae converging towards posterior side but not meeting each other; T1 with a few broken carinulae on posterior half of space between submedian and sublateral carinae and between outer submedian and inner submedian carinae (careful observation under proper illumination is necessary to observe these weak carinulae); T1 mostly smooth and shiny; remaining tergites each with a row of small setigerous pits which are denser on sides. Ovipositor sheath a little protruding; hypopygium exceeding middle of metasoma.

*Male*. Unknown.

##### Host.

Unknown.

##### Distribution.

Vietnam.

##### Etymology.

Named after its country of origin, Vietnam.

#### Oxycoryphe

Taxon classificationAnimaliaHymenopteraChalcididae

Kriechbaumer, 1894

Figs 173–174 , 175–176 , 177 , 178–179 , 180–181 , 182

Oxycoryphe Kriechbaumer, 1894a: 67. Type species: Oxycoryphe subaenea Kriechbaumer; by monotypy.
Paraspirhina Cameron, 1911: 14. Type species: Parasirhina nitida Cameron; by monotypy. Synonymised with Oxycoryphe Kriechbaumer by Bouček 1988b.
Hoozania Masi, 1932a: 40. Type species: Hoozania maculipennis Masi, by original designation. Synonymised with Oxycoryphe Kriechbaumer by Bouček 1988b.

##### Diagnosis.

This genus resembles *Sthulapada* Narendran in having an external extra carina on the hind tibia and a median tubercle on the pronotum. However, it differs from *Sthulapada* in having normal hind tarsal segments (in *Sthulapada* the hind tarsal segments (especially distal one) are unusually and greatly swollen) and the ovipositor sheath without a tooth (in *Sthulapada* ovipositor sheath with a tooth ventrally).

##### Description.

Pronotal collar in middle with a short median keel or tooth or a raised triangle (rarely weakly developed but distinctly indicated); apex of scutellum in most species distinctly produced posteriorly, ending in a lobe (in a few species emarginate); hind tibia with an additional external carina; mesosoma quite flat in some species; epipygium in typical form reaches near subapical part of ovipositor sheath whereas in some other species not at all reaching apex.

##### Hosts.

Parasitoids of Lepidoptera.

##### Distribution.

Africa (South Africa, Uganda) and Asia (India, Philippines, Malaysia, Singapore, Indonesia (Sumatra, Kalimantan), China (Taiwan) (Narendran 1989; Noyes 2011), Vietnam (new record).

##### Key to Vietnamese species of *Oxycoryphe* Kriechbaumer (based on females)

**Table:** 

1	Projected part of apex of scutellum long, 0.2 × length of remaining part of scutellum and distinctly bi-lobed (Fig. 182); width of head twice its length in dorsal view; POL 3–4 × OOL; clava at most twice as long as preceding segment (Fig. 180); hind leg completely black (Fig. 178)	***Oxycoryphe scutellatus* Narendran**
–	Projected part of scutellum shorter than above and at most slightly bi-lobed (Fig. 177); head width 2.9 × its length in dorsal view; POL 6–8 × OOL (Fig. 175); clava a little longer than twice preceding segment (Fig. 175); hind leg reddish or yellowish brown (Fig. 173)	***Oxycoryphe neotenax* sp. n.**

**Figures 173–174.**
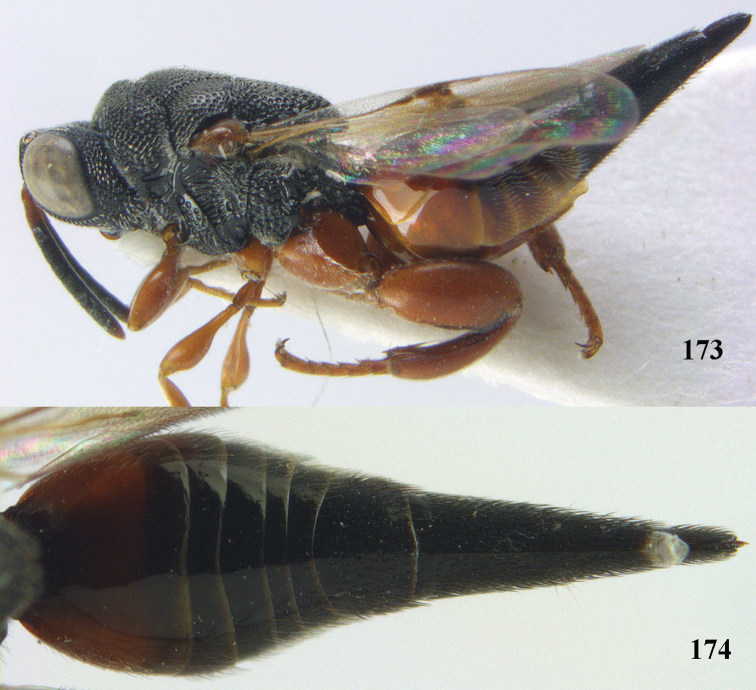
*Oxycoryphe neotenax* sp. n., ♀, holotype. **173** habitus lateral **174** metasoma dorsal.

**Figures 175–176.**
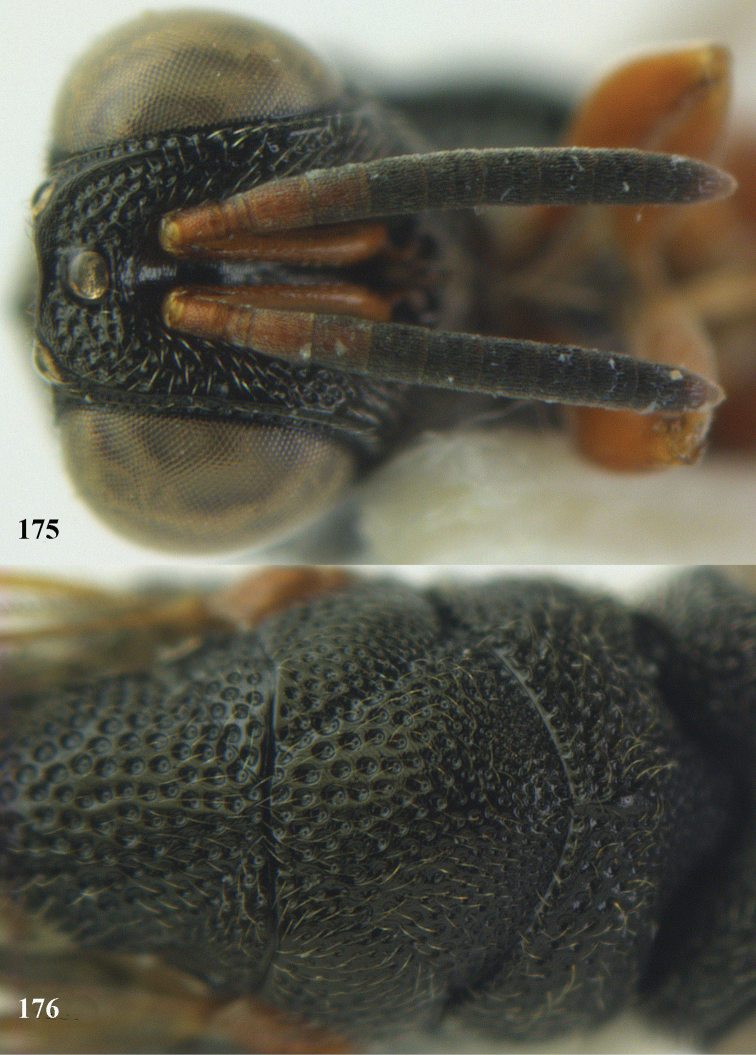
*Oxycoryphe neotenax* sp. n., ♀, holotype. **175** head anterior **176** mesoscutum dorsal.

**Figure 177.**
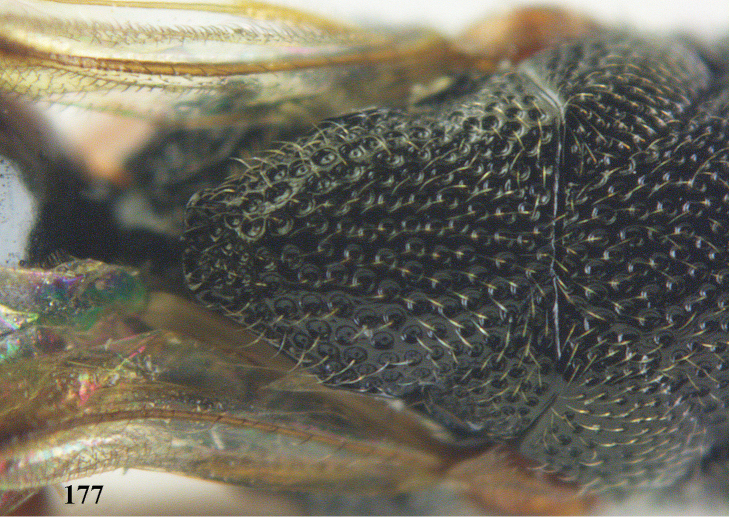
*Oxycoryphe neotenax* sp. n., ♀, holotype, scutellum dorsal.

**Figures 178–179.**
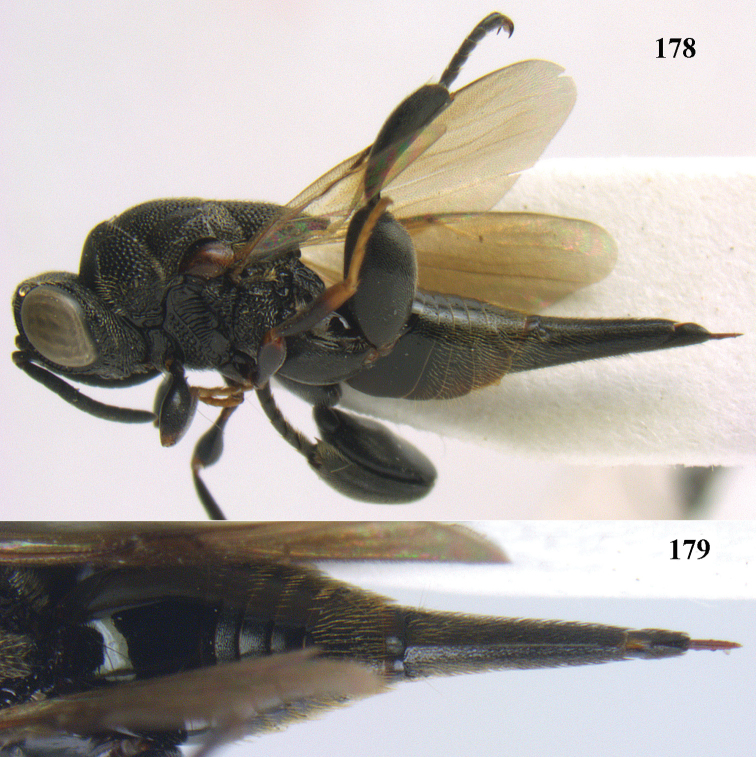
*Oxycoryphe scutellatus* Narendran, ♀, Cát Tiên N.P. **178** habitus lateral **179** metasoma dorsal.

**Figures 180–181.**
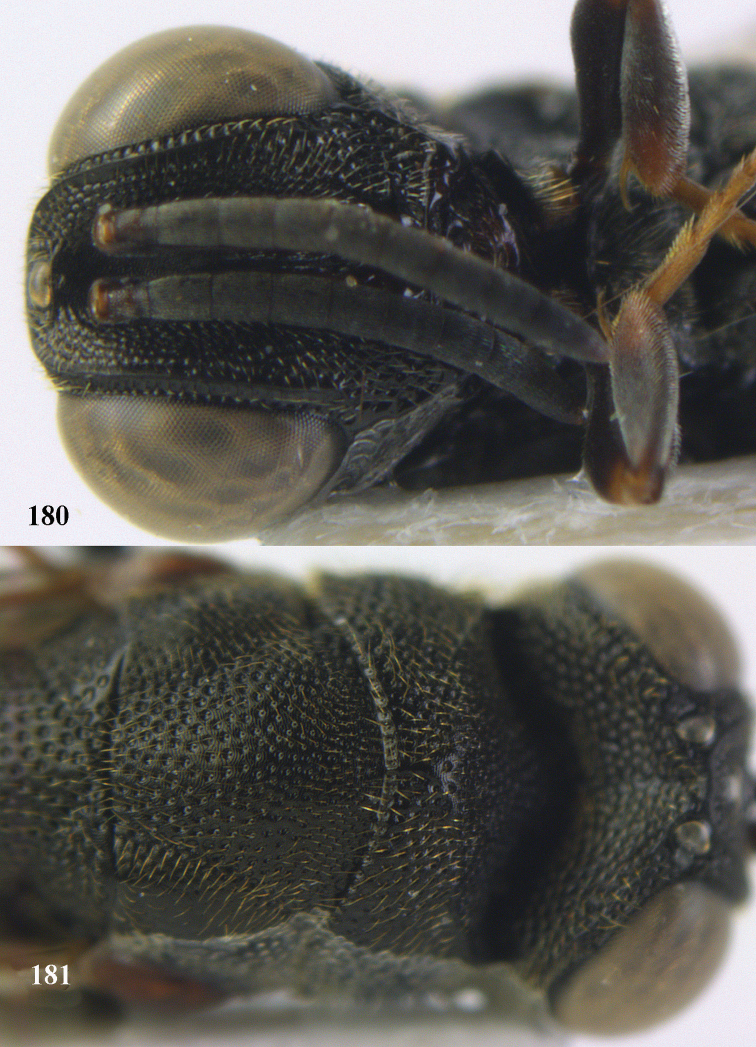
*Oxycoryphe scutellatus* Narendran, ♀, Cát Tiên N.P. **180** head anterior **181** mesoscutum dorsal.

**Figure 182.**
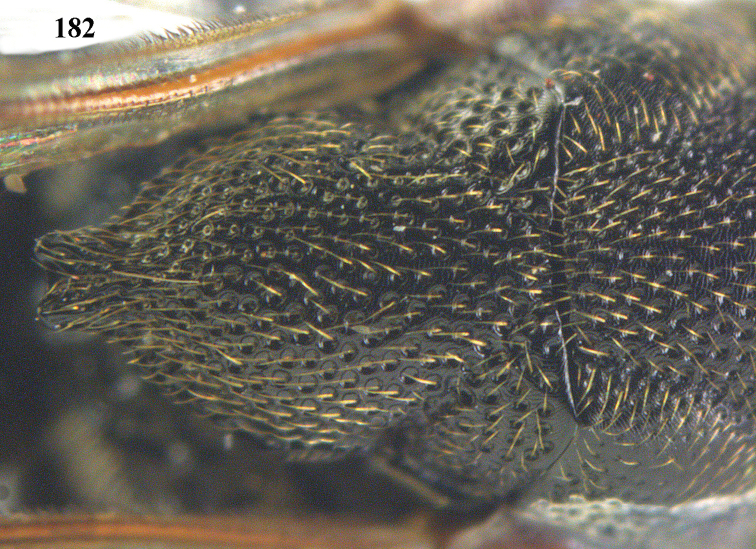
*Oxycoryphe scutellatus* Narendran, ♀, Cát Tiên N.P., scutellum dorsal.

#### Oxycoryphe neotenax sp. n.

Taxon classificationAnimaliaHymenopteraChalcididae

http://zoobank.org/8BA8E5A9-6415-4207-B7FC-C9B0A711E46C

Figs 173–174 , 175–176 , 177

##### Type material.

Holotype, ♀ (RMNH) “**Vietnam**: Ninh Thuân, Núi Chúa N. P., northwest part, Mal. trap 17, c. 150 m, 24–30.v.2007, C. v. Achterberg & R. de Vries, RMNH’07”. Paratypes: 2 ♀ (RMNH, IEBR), “Vietnam: Ninh Thuân, Núi Chúa N. P., northeast part, Mal. traps, 90–150 m, 23–30.v.2007, C. v. Achterberg & R. de Vries, RMNH’07”; 1 ♀ (RMNH), id., but dry south part, 100–180 m, 22–29.v.2007, C. v. Achterberg & R. de Vries, RMNH’07”; 2 ♀ (RMNH, IEBR), “S. Vietnam: Dóng Nai, Cát Tiên N. P., Bird trail, Mal. traps 30–35, c. 100 m, 15–20.v.2007, C. v. Achterberg & R. de Vries, RMNH’07”.

##### Diagnosis.

Very similar to *Oxycoryphe tenax* Narendran, 1989, from Malaysia, but *Oxycoryphe neotenax* has the lateral ocelli close to the eyes (POL 6–8 × OOL; 3.3–4.3 × in *Oxycoryphe tenax*), PMV distinctly longer than SV (about as long as SV in *Oxycoryphe tenax*), the hind femur and tibia reddish to yellowish brown (brownish yellow in *Oxycoryphe tenax*), the post-orbital carina not well developed (distinct in *Oxycoryphe tenax*) and PMV as long as MV or longer (less developed in *Oxycoryphe tenax*).

##### Description.

Holotype, ♀, length of body 6.0 mm.

*Colour*. Black with following parts as follows: eyes and ocelli pale gray; scape and pedicel pale brownish yellow; annellus, F1, F2 and F3 brownish yellow; remaining segments of antenna dark brown; apex of clava, fore coxa pale brown, remaining segments of fore and mid legs yellow; hind leg reddish pale brown; tegula pale brownish yellow; metasoma black with yellowish brown on sides from T1 to T6; epipygium and ovipositor sheath black. Pubescence pale white with slight yellowish tinge; fore wing hyaline with a brownish tinge; veins dark brown; pilosity of wing disc blackish brown.

*Head*. Width 1.1 × its height in anterior view; width in dorsal view 2.9 × its length, subequal to width of mesosoma; pre-orbital carina distinct meeting each other behind anterior ocellus; post-orbital carina absent; geno-temporal furrow present; scrobe cross striate-reticulate, reaching anterior ocellus; posterior ocellus close to eye (fig. 175), POL 6.2 × OOL; AOL twice OOL; LOL a little longer than AOL (9:8); shortest width between eyes in dorsal view 2.2 × POL; face and vertex with close, deep umbilicate, setigerous pits; MS indistinct, malar space 0.4 × eye height in profile; eyes with a few scattered minute pilosity; antenna inserted well below level of ventral margin of eyes, scape not quite reaching anterior ocellus; antennal formula 11273; relative L:W of antennal segments:scape = 29:3; pedicel = 4:4; F1 = 7:5; F2 = 7:5; F3 = 6:6; F4 = 6:6; F5 = 6:6; F6 = 6:6; F7 = 6:6; clava = 13:6.

*Mesosoma*. Pronotum with a tooth-like raised structure medially (but not distinctly forming a tooth as in the type species), with close setigerous pits, interstices narrower than diameter of a pit, somewhat carinate, reticulate; posterior margin of pronotum concave; mesoscutum and scutellum with close setigerous pits; interstices rugulose, narrower than diameter of a pit; apex of scutellum a little produced posteriorly; propodeum with submedian and sublateral carinae distinct, areolate, lateral teeth indistinct.

*Wings*. Fore wing length 3.1 × its width, densely pilose; CC about 7 × as long as MV; PMV subequal to MV, longer than STV.

*Legs*. Hind coxa with a weak tooth at dorso-basal side; hind femur 2.8 × as long as wide; hind tibia with an extra external carina.

*Metasoma*. Metasoma 1.6 × as long as mesosoma, length of pre-epipygial part of metasoma a little longer than mesosoma (27:21), but subequal to length of mesosoma in holotype; T1 with a pair of basal longitudinal carinae, each carina as long as width between each other; posterior margin of T1 to T5 smooth and shiny, T6 with shallow setigerous, micro-punctate; epipygium with close, shallow setigerous pits, with a median carina, fully pubescent.

*Male*. Unknown.

##### Host.

Unknown.

##### Distribution.

Vietnam.

##### Variation.

Length of female varies from 6.0–6.5 mm; POL 6–8 × OOL; fore wing with a small infuscation below SMV in some specimens; 4–6 basal segments of antenna yellowish brown.

#### Oxycoryphe scutellatus

Taxon classificationAnimaliaHymenopteraChalcididae

Narendran, 1989

Figs 178–179 , 180–181 , 182

Oxycoryphe scutellatus Narendran, 1989: 131 (♀, holotype, E. Malaysia, SW. Sabah) (RMNH, examined)).

##### Material.

2 ♀ (RMNH, IEBR), “S. **Vietnam**: Dóng Nai, Cát Tiên N. P., Bird trail, Mal. traps 30–35, c. 100 m, 15–20.v.2007, C. v. Achterberg & R. de Vries, RMNH’07”.

##### Diagnosis.

This species comes near *Oxycoryphe thresiae* Narendran, 1989, in the key to species by Narendran (1989), but differs from it in having: 1) scrobe cross reticulate (in *Oxycoryphe thresiae* scrobe striate); 2) apex of scutellum projecting 0.17–0.24 × length of remaining part of scutellum (in *Oxycoryphe thresiae* apex of scutellum projecting less than 0.17 × length of remaining part of scutellum); 3) PMV longer than MV (in *Oxycoryphe thresiae* PMV equal to MV); 4) T1 with 2 basal carinae (in *Oxycoryphe thresiae* T1 without a pair of basal carinae, but with a subrectangular pit), and 5) POL 3 × OOL (in *Oxycoryphe thresiae* POL much more than 3 × OOL).

##### Description

(specimen from Cát Tiên N. P.). ♀, length of body 7.5 mm.

*Colour*. Black; eyes grayish yellow; ocelli pale reflecting yellow; bases and apices of fore and mid femora and tibiae slightly yellowish brown; fore and mid tarsi pale brownish yellow; hind leg completely black; wings hyaline with slight yellowish brown infuscation with a weak brownish yellow arch connecting parastigma and STV.

*Head*. Width of head 1.1 × its height (excluding mandibles) in anterior view; width in dorsal view twice its length, a little wider than mesosoma (20:17); height of eye in profile 2.6 × height of malar space, length of eye in profile 1.7 × height of malar space; scrobe almost reaching anterior ocellus, separated from it by a sharp cross ridge; surface of scrobe cross reticulate; pre-orbital carinae horse-shoe-like, ventrally touching malar ridge; pre-orbital carina running upwards towards temple; auricular carina almost reaching malar ridge, not clearly touching it; geno-temporal furrow very narrow, narrower than diameter of adjacent pit, 0.3 × length of fore femur. POL 3.5 × OOL; AOL 1.8 × OOL; face and vertex with deep, close, umbilicate, setigerous pits; interstices carinate and rugose; antenna with scape not reaching anterior ocellus; relative L:W of antennal segments:scape = 28:4; pedicel = 3:4; F1 to F7 equal length and width (= 6:5); clava = 10:5.

*Mesosoma*. Pronotum anteriorly with wavy carinae joining at middle to form a median carina running to posterior margin; middle lobe of mesoscutum with close umbilicate pits, interstices as broad as or half as broad as diameter of a pit in most areas, distinctly rugulose; scutellum with slightly larger and deeper pits than those of middle lobe of mesoscutum, scutellum slightly longer than mesoscutum (26:24) (including projecting apex), 1.2 × as long as wide; apex of scutellum projected posteriorly, bi-lobed, projected apex 0.2 × as long as remaining part of scutellum (4:22). Propodeum declining 60° to the vertical axis of scutellum; submedian and sublateral carinae well developed; post spiracular tooth on each side distinct.

*Wings*. Fore wing 3 × as long as broad; relative length of CC = 25; SMV = 22; parastigma = 3; MV = 5; PMV = 6; STV = 2.

*Legs*. Hind coxa with a distinct dorso-basal tooth; hind femur without an inner basal tooth.

*Metasoma*. Metasoma 1.5 × as long as mesosoma; T1 with basal carinae as long as space between them, smooth and shiny; T1 0.3 × as long as metasoma, its posterior margin a little concave medially; T2 to T5 micro-sculptured, moderately pubescent laterally; posterior margins of T2 to T5 a little concave; T6 with 9–10 cross rows of shallow pits, interstices weakly micro-sculptured without a distinct smooth basal marginal strip; T6 twice as long as T5, 0.4 × as long as epipygium; visible part of ovipositor sheath 0.3 × length of epipygium.

*Male*. See Narendran (1989) for description.

##### Host.

Unknown.

##### Distribution.

Vietnam (new record), Malaysia, Indonesia (Sumatra), Philippines (Narendran 1989).

##### Variation.

Length body of ♀ 5.3–7.8 mm. Hind coxa with basal admarginal area pale brownish, yellow in one specimen; metasoma 1.5–1.6 × as long as mesosoma; epipygium 2–4 × as long as T6; projected apex of scutellum 0.17–0.24 × as long as remaining length of scutellum; postspiracular tooth weak to strong. In some specimens hind tarsi black, while in one specimen first 3 hind tarsal segments are dark brown.

#### Psilochalcis

Taxon classificationAnimaliaHymenopteraChalcididae

Kieffer, 1905

Figs 183–184

Psilochalcis Kieffer, 1905: 250–251. Type species: Psilochalcis longigena Kieffer, by monotypy. Redescribed by Steffan (1951b).
Leptochalcis Kieffer, 1905: 251–252. Type species: Leptochalcis filicornis Kieffer, by monotypy. Synonymised with Psilochalcis by Steffan (1951b).
Invreia Masi, 1929b: 210–211. Type species: Invreia subaenea Masi, by original designation. Synonymised with Psilochalcis by Bouček (1992).
Euchalcidia Masi, 1929b: 220–222. Type species: Euchalcidia elegantula Masi, designated by Masi, 1929. Synonymised with Psilochalcis by Bouček (1992).
Chalcidiopsis Masi, 1933: 4. Type species: Chalcidiopsis odontomera Masi, by original designation. Synonymised with Psilochalcis by Narendran and Sudheer (2005).
Peltochalcidia Steffan, 1949: 121. Type species: Peltochalcidia benoisti Steffan, by original designation. Synonymised with Psilochalcis by Bouček (1992).
Hyperchalcidia Steffan; 1951b: 67. Type species: Hyperchalcidia soudanensis Steffan, by original designation. Synonymised with Psilochalcis by Narendran (1989).
Parinvreia Steffan, 1951a: 7 (as subgenus of Invreia). Type species: Invreia frequens Masi, designated by Bouček (1984). Synonymised with Psilochalcis by Bouček (1992).

##### Diagnosis.

See under *Notaspidium*.

##### Description.

Horizontal ventral part of mesopleuron anteriorly with a carina defining margin of area for reception of fore coxae; body usually with dense pubescence. MV short and distinctly removed from anterior wing margin; hind tibia with 2 apical spurs. Antenna 13 segmented; in ♀ antenna inserted at clypeus; in ♂ antenna inserted a little above it; scape not reaching anterior ocellus; head in front view roundly triangular or subrectangular as in *Psilochalcis soudanensis* group; outer surface of clypeus gradually turns downwards and its upper edge projects slightly to strongly in different species; body densely setose in some species; mesosoma sturdy, in some species propodeum almost horizontal and with very distinct carinae (submediae, accessoriae, sublaterales and costae lateralis); scutellum flatly arched, posteriorly broadly rounded or truncated; hind femur with a ventral row of teeth, basal tooth massive in “*Chalcidiopsis*” and typical *Psilochalcis*; metasoma with large, tongue-shaped, posteriorly rounded T1.

##### Hosts.

Parasitoids of lepidopterous pupae.

##### Distribution.

North and Central America, Europe, Africa, Madagascar, Oriental region and Japan.

**Figures 183–184.**
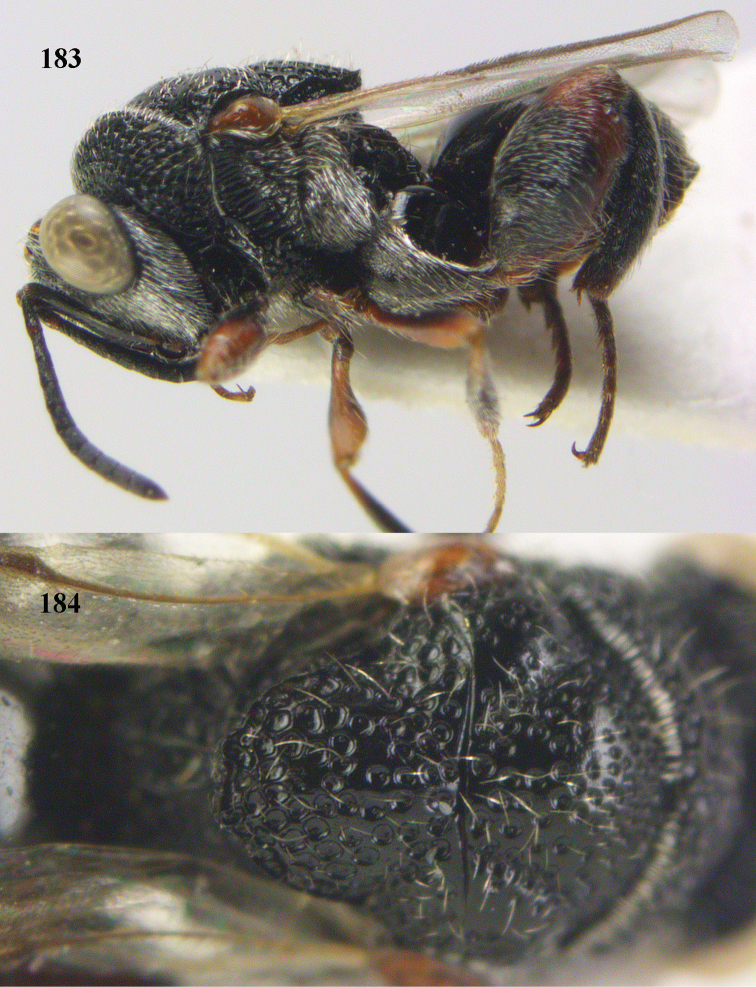
*Psilochalcis carinigena* (Cameron), ♀, Cát Tiên N. P. **183**. habitus lateral **184** mesosoma dorsal.

#### Psilochalcis carinigena

Taxon classificationAnimaliaHymenopteraChalcididae

(Cameron, 1907)

Figs 183–184

Coelochalcis carinigena Cameron, 1907: 579 (lectotype (designated by Narendran 1989), India (BMNH) (examined)).
Psilochalcis carinigena ; Narendran 1987b: 438–439.
Chalcidiopsis odontomera Masi, 1933: 5 (♂, lectotype, Taiwan (SDEI) examined, designated by Bouček 1988); Narendran and Sudheer 2005: 89–98 (synonymy with Psilochalcis carinigena (Cameron)).
Invreia opisinae Narendran, 1985: 87 (♂, holotype, India (CNC), synonymised with Psilochalcis carinigena (Cameron) by Narendran 1989).

##### Material

(RMNH, IEBR). 3 ♂, “**Vietnam**: Ninh Thuân, Núi Chúa N. P., Northeast part, Malaise traps, 90–190 m, 19–25.iv.2007, Mai Phu Quy & Nguyen Tanh Manh, RMNH’07”; 1 ♀, “S. Vietnam: Dóng Nai, Cát Tiên N. P., Dong trail, Mal. traps, c. 100 m, 1–8.iv.2007, Mai Phu Quy & Nguyen Tanh Manh, RMNH’07”, 1 ♀, id., but Botanical Garden, 13–20.v.2007, C. v. Achterberg, R. de Vries & E. Gassó Miracle.

##### Diagnosis.

This species comes very near *Psilochalcis keralensis* Narendran in general colour, but *Psilochalcis keralensis* differs in having: 1) lateral part of fore coxa almost smooth with few weak incomplete rugae on lower part (in *Psilochalcis carinigena* lateral part of fore coxa with several distinct rugae); 2) malar ridge incomplete, not reaching eye (in *Psilochalcis carinigena* malar ridge complete, reaching ventral margin of eye); 3) apex of scutellum not emarginate (in *Psilochalcis carinigena* apex of scutellum at least slightly emarginate); 4) clava as long as preceding two segments combined (in *Psilochalcis carinigena* clava 1.3 × as long as preceding segments combined) and 5) T3 0.3 × as long as T2 in dorsal view (in *Psilochalcis carinigena* T3 0.5 × as long as T2).

##### Description.

♀, length of body 2.9–4.6 mm.

*Colour*. Black; femora and tibiae brownish red, hind tibia and tarsus brownish black; tegula pale brown or brown.

*Head*. Scrobe cross rugulose, not reaching anterior ocellus; scape 4 × as long as pedicel; apex of scutellum slightly to moderately incised or bi-lobed; propodeum horizontal basally, subvertical apically.

*Legs*. Hind coxa without a tooth on dorsal or ventral side; hind femur with a large basal tooth.

*Metasoma*. Metasoma without basal carinae; T1 shagreened on disc; head and body densely pubescent.

*Male*. Similar to female except in having stouter antenna with a dent on scape; reddish colour of hind femur and tibia more blackish; body more densely pubescent than that of female.

##### Variation.

In some Vietnamese specimens hind femur black or red with a black patch (Fig. 183); tibiae more blackish in some specimens.

##### Hosts.

Lepidoptera: *Opisina arenosella* Walker (Oecophoridae) and *Hyblaea puera* Cramer (Hyblaeidae) (Narendran 1989).

##### Distribution.

India, China (Taiwan), Vietnam (new record).

#### Smicromorpha

Taxon classificationAnimaliaHymenopteraChalcididae

Girault, 1913

Figs 185–188

Smicromorpha Girault, 1913b: 89. Type species: Smicromorpha doddi Girault, by original designation and monotypy.
Smicromorphella Girault, 1930: 2. Type species: Smicromorphella minera Girault, by monotypy (synonymised with Smicromorpha Girault by Naumann 1986).

##### Diagnosis.

**Figures 185–188.**
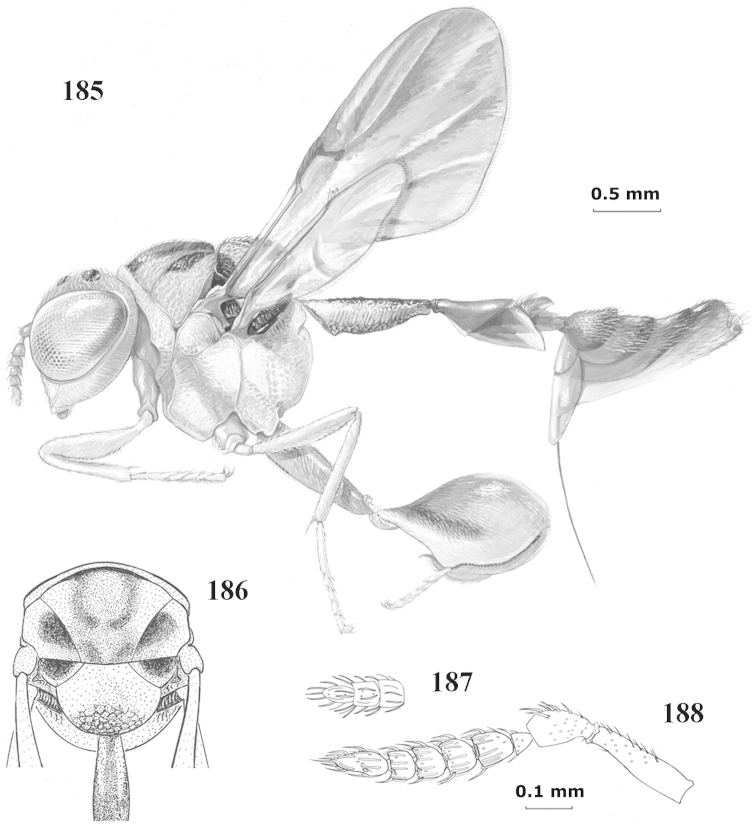
*Smicromorpha masneri* Darling, ♀, Bach Ma N. P. **185** habitus lateral **186** mesosoma dorsal **187** apical antennal segments **188** antenna. From Darling 2009.

Unique among the Chalcididae because of shortened antennal flagellum (usually much shorter than length of eye) with less than 11 distinguishable segments (Figs 185, 188) and usually tail-like metasoma attached at upper margin of propodeum (Fig. 185).

##### Description.

Integument of body predominantly translucent, yellow to orange in colour, occasionally with brown or black markings, non-metallic. Eyes moderate sized to very large, inner margins entire; occipital carina absent; occipital suture dorsally distinct; antennal scrobe deep, margins usually carinate; toruli slightly above or slightly below level of ventral margin of eye, never near anterior tentorial pits; anterior margin of clypeus very weakly emarginated, not produced; mandibles asymmetrical; antenna relatively short, 9 segmented; scape 3.6–4.8 × as long as wide; pedicel swollen; flagellum fusiform or filiform; clava unsegmented. Pronotum without transverse carina; mesoscutum with notauli distinct, percurrent; scutellum with axilla differentiated; frenum not differentiated; prepectus minute, scale-like; mesopleuron with deep ventral depression; dorsellum absent; propodeum rugose-punctate, without carinae or plicae; petiolar foramen situated anteriorly, separated from metanotum by very narrow rim. Hind coxa subequal in length to hind femur; hind femur greatly enlarged, with a comb of teeth on outer ventral margin, without an inner tooth; hind tibia ventro-apically produced into a spine with a small spur near apex of spine, without additional external carinae; tarsi 5 segmented. Wings densely pilose, sparse on basal part; SMV very long (longer than twice or more length of MV) parallel to costal margin; MV shorter than or as long as STV. Metasoma elongate, in ♀ T1 and S1 fused to form a petiole; segments 2–4 depressed, subcylindrical; segments 5–8 laterally compressed; anterior segment of petiole with strong dorsal condyle providing articulation with propodeum; posterior segment of petiole defined antero-laterally and antero-ventrally by transverse lamina, posteriorly slightly overlapping T2 and S2. In ♂ T6-T8 elongate, differentiated into an anterior part concealed by preceding tergite and an exposed posterior setose part; T8 undivided.

##### Host.

*Oecophylla smaragdina* (Fabricius) (Hymenoptera: Formicidae).

##### Distribution.

Australia, Vietnam, New Guinea, Africa (Narendran 1979, 1989; Naumann 1986; Bouček 1988b and Darling 2009).

#### Smicromorpha masneri

Taxon classificationAnimaliaHymenopteraChalcididae

Darling, 2009

Figs 185–188

Smicromorpha masneri Darling, 2009: 157 (♀, holotype, Vietnam, Bach Ma N.P., (ROMT)).

##### Diagnosis.

*Smicromorpha masneri* comes near *Smicromorpha lagynos* Naumann, 1986, in the key to species by Naumann (1986), but differs from it having: 1) paired dorsal carinae at base of first tergite absent (in *Smicromorpha lagynos* strong paired dorsal carinae present at base of first tergite); and 2) anterior tentorial pits distinct (in *Smicromorpha lagynos* anterior tentorial pits absent).

##### Description

(based on Darling 2009). ♀, length of body 4.2–5.0 mm; fore wing length 2.3–2.6 mm.

*Colour*. Predominantly pale yellow to white, darker dorsad; mesoscutum with light brown areas, a diffuse circle on middle lobe of mesoscutum and along transscutal articulation, and dark brown along notauli; axilla and lateral panel of axilla dark brown or black; apex of scutellum light brown; lateral panel of metanotum dark brown; propodeum white; fore and middle legs white; hind legs dark yellow dorsally, white below; femoral teeth black, tibia yellow; tarsus white; metasoma brown, darker above; petiole dark brown; but yellowish white basad; antenna yellow.

*Head*. Head in anterior view wider than high, in dorsal view wider than long; malar sulcus indistinct. Pronotal collar laterally carinate. Propodeum posteriorly convex; spiracular sulcus indistinct.

*Wings*. Fore wing with stump of basal vein (1-SR) present; STV shorter than MV.

*Legs*. Hind coxa 3.5–4.1 × as long as high; hind femur 1.7–2.0 × as long as high, with a weak ventral process and well developed ventral teeth.

*Metasoma*. First metasomal tergite subequal in length to hind coxa, 3.5 × as long as wide, dorsally minutely reticulate-punctate, without paired dorsal carinae; transverse lamina indistinct, not extending postero-ventrally to middle length of first tergite.

*Male*. Unknown.

##### Host.

*Oecophylla smaragdina* (Fabricius) (Formicidae).

##### Distribution.

Vietnam.

#### Sthulapada

Taxon classificationAnimaliaHymenopteraChalcididae

Narendran, 1989

**Figure 189.**
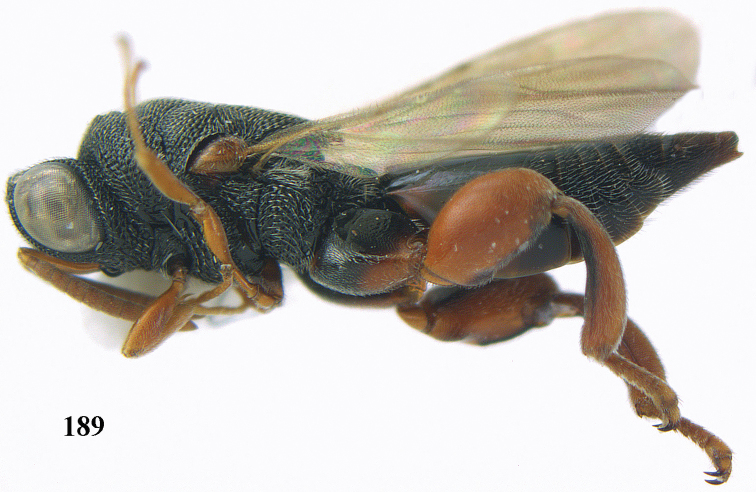
*Sthulapada neopadata* sp. n., ♀, holotype, habitus lateral.

**Figures 190–191.**
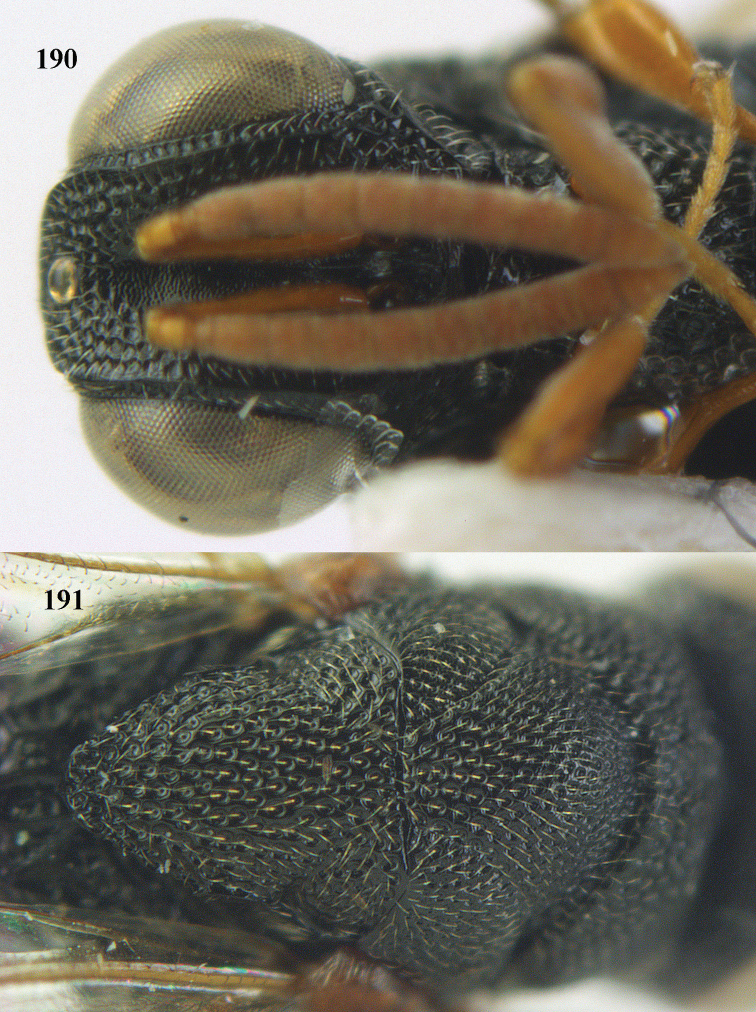
*Sthulapada neopadata* sp. n., ♀, holotype. **190** head, anterior **191** mesosoma dorsal.

**Figure 192.**
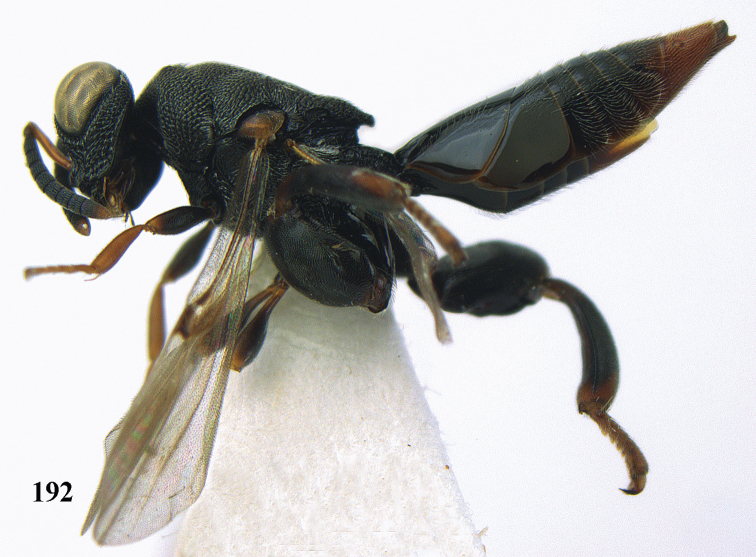
*Sthulapada vietnamensis* sp. n., ♀, holotype, habitus lateral.

**Figures 193–194.**
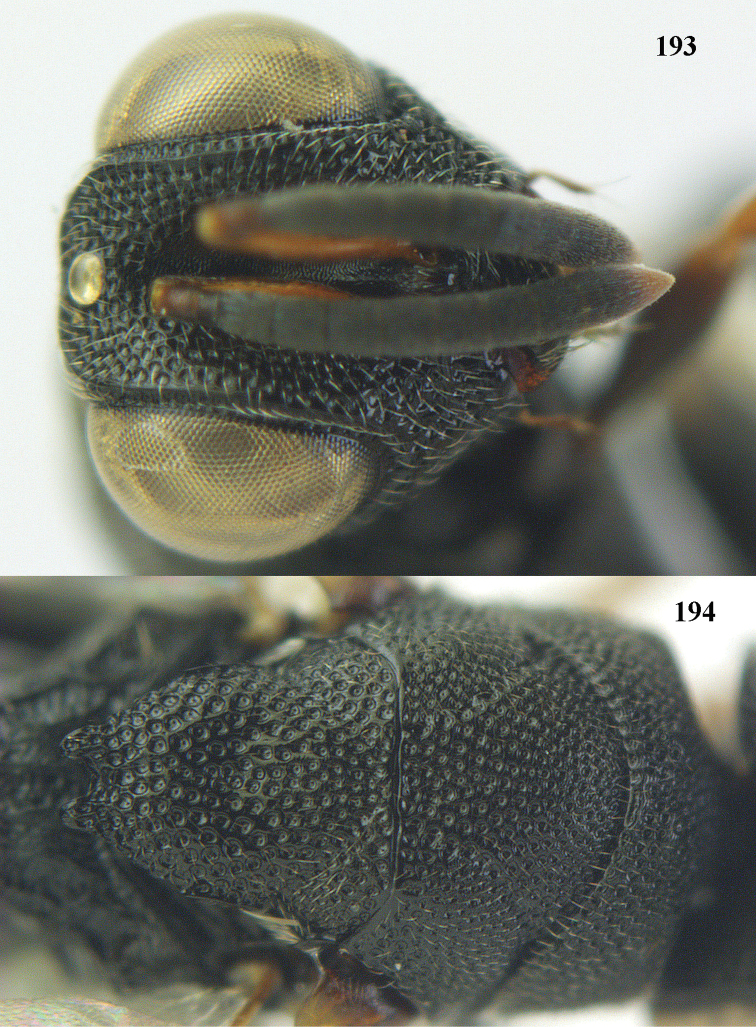
*Sthulapada vietnamensis* sp. n., ♀, holotype. **193** head anterior **194** mesosoma dorsal.

Figs 189 , 190–191 , 192 , 193–194

Sthulapada Narendran, 1989: 145. Type-species: Sthulapada padata Narendran, 1989, by monotypy.

##### Diagnosis.

This genus comes near *Thresiaella* Narendran, 1989, in the key to genera of Chalcididae by Narendran (1989), but differs from it in having: 1) apex of hind tibia and hind tarsus swollen (in *Thresiaella* hind tibia and hind tarsus not swollen), and 2) ovipositor sheath with a tooth at apex (in *Thresiaella* ovipositor sheath without tooth). *Sthulapada* also comes near *Oxycoryphe* in general appearance, but differs from it in having peculiarly developed hind tarsal segments and ovipositor sheath with a tooth at apex.

##### Description.

Head wider than mesosoma; scrobe deep, not quite reaching anterior ocellus; clava pointed; pre-orbital carinae joining each other behind anterior ocellus; pronotum with a median tubercle or with two humps; pronotum and mesonotum convex; hind femur greatly swollen; hind tibia greatly clavate; hind tarsal segments (especially telotarsus) unusually swollen; epipygium with a tooth on ventral side below ovipositor sheath apically.

##### Host.

Unknown.

##### Distribution.

Malaysia, Vietnam (new record).

##### Key to species of *Sthulapada* Narendran

**Table:** 

1	Fore wing without a brownish infuscation adjoining MV; distal end of SMV not clavate; hind coxa with a weak dorso-basal tooth; F1 shorter than F2; clava 2.3 × as long as F7; hind coxa black with a brownish yellow apex; [not yet found in Vietnam]	***Sthulapada padata* Narendran**
–	Fore wing with a brownish infuscation adjoining MV present; distal end of SMV clavate; hind coxa without a dorso-basal tooth; F1 as long as F2; clava different from above; colour of hind coxa partly or completely different	**2**
2	Antenna mostly black but scape and pedicel pale brownish yellow and pale brown, respectively; legs mostly black; metasoma black with epipygium reddish brown; apex of scutellum bi-lobed; propodeum without a median carina	***Sthulapada vietnamensis* sp. n.**
–	Antenna entirely brownish yellow; legs mainly brownish yellow but hind coxa black basally; metasoma brownish basally and ventrally; apex of scutellum uni-lobed; propodeum with a short median carina	***Sthulapada neopadata* sp. n.**

#### Sthulapada neopadata sp. n.

Taxon classificationAnimaliaHymenopteraChalcididae

http://zoobank.org/6B8C3645-8F2A-477B-8875-D8CFFF07210D

Figs 189 , 190–191

##### Material.

Holotype (RMNH), ♀, “**Vietnam**: Dak Lak, Chu Yang Sin N. P., n[ea]r dam, 600–1000 m, 2–10.vi.2007, Mal. traps 9–11, C. v. Achterberg & R. de Vries, RMNH’07”.

##### Diagnosis.

This new species differs from the type species *Sthulapada padata* Narendran in having: 1) pits on mesoscutum and scutellum close, with interstices narrower than half a diameter of a pit and rugulose (in *Sthulapada padata* interstices of mesoscutum and scutellum wider, smooth, shiny and not carinate); 2) fore wing without brownish infuscation adjoining MV (in *Sthulapada padata* with brownish infuscation adjoining MV); 3) distal end of SMV not clavate (in *Sthulapada padata* distal end of SMV clavate); 4) hind coxa with a weak dorso-basal tooth (in *Sthulapada padata* hind coxa without a dorso-basal tooth); 5) hind femur 0.6 × as broad as long (in *Sthulapada padata* hind femur 0.7 × as broad as long); 6) hind tibia width 0.2 times its length (in *Sthulapada padata* hind tibia width 0.3 times its length); 7) clava 2.3 × as long as F7 (in *Sthulapada padata* clava 2.6 × as long as F7); 8) F1 shorter than F2 (in *Sthulapada padata* F1 as long as F2); 9) metasoma mainly black (in *Sthulapada padata* metasoma mostly yellowish brown); 10) hind coxa black with a brownish yellow patch apically (brownish yellow more extensive on ventral apical part than dorsal apical part; in *Sthulapada padata* hind coxa mostly yellow with a black or reddish black patch dorsally from base to slightly beyond middle).

##### Description.

Holotype, ♀, length of body 4.3 mm.

*Colour*. Black, but eyes and ocelli pale grayish yellow; antenna orange yellow; fore leg pale brownish yellow except black coxa; mid leg similar to fore leg except coxa black with yellowish brown tinge, especially towards its distal end; hind coxa black with apical part brownish yellow; hind femur orange yellow; hind tibia orange brown with ventral part medially black; tarsi orange brown; telotarsi black; wings hyaline, veins dark brown; pubescence on body white.

*Head*. Width of head in anterior view a little more than its height (32:29); width in dorsal view about twice its length, a little wider than mesosoma (excluding tegulae) (31:28); occiput concave; surface of face, vertex and occiput with close umbilicate pits, interstices carinate; POL 4 × OOL; AOL 2.3 × OOL; distance between eye in dorsal view 2.4 × POL; pre-orbital carina starting from malar ridge running upwards towards vertex and meeting each other behind anterior ocellus; malar sulcus forming malar ridge; geno-temporal furrow deep; scrobe reticulate, not reaching anterior ocellus, margins not carinate; malar space 0.4 times height of eye in profile; eye length in profile 0.7 × eye height; eyes bare; antenna inserted well below level of ventral margin of eyes; scape not at all reaching anterior ocellus; relative L:W of antennal segments:scape = 16:3; pedicel = 4:3; F1 = 5:3; F2–F7 = 4:4; clava = 9:4.

*Mesosoma*. Mesosoma with close umbilicate pits, interstices narrower than half diameter of a pit, rugulose; pronotum with two humps in the submedian part, posterior margin concave medially; apex of scutellum entire; propodeum horizontal, with distinct submedian and sublateral carinae; area between submedian carinae with 12–15 cross carinulae, spaces between carinulae smooth and shiny; area between plical carina and submedian carina alveolate; lateral paraspiracular tooth on each side weakly represented, spiracle bean-shaped.

*Wings*. Fore wing 2.9 × as long as its width; relative length of CC = 44; SMV = 43; MV = 6; PMV = 1; STV = 4. MV separated from SMV by a distinct break; distal end of SMV not clavate as in *Sthulapada padata*.

*Legs*. Hind coxa without a dorso-basal tooth; hind femur 0.6 × as broad as long with a strong inner basal tooth, outer ventral margin with a row of teeth extending from apex to posterior 0.6, ventral margin not distinctly bi-lobed; hind tibia with an additional extra external carina; hind tarsal segments relatively larger, wider than long; fifth tarsal segment 1.5 × as broad as fourth tarsal segment, about 3 × as long as fourth tarsal segment; claws uni-dentate.

*Metasoma*. Metasoma 1.3 × longer than mesosoma; T1 with 3 basal carinae; median carina shorter than lateral carinae; length of median carina 0.2 × length of T1; lateral carina 0.3 × length of T1 in dorsal view; length of T1 a little shorter than half length of metasoma; T1 smooth and shiny, its posterior margin a little convex; T2 smooth with 2 cross rows of minute setigerous pits, pits discontinues at middle in anterior row; T3-T5 rugulose with scattered pits on sides and a single row posteriorly; posterior margin of T2-T4 almost straight; posterior margin of T5 weakly concave; T6 with 7–8 cross rows of setigerous pits; epipygium carinate at middle, its median length a little longer than median length of T6 (10:9); epipygium with a distinct posteriorly directed tooth just below ovipositor sheath; ovipositor sheath about half length of epipygium medially.

*Male*. Unknown.

##### Host.

Unknown.

##### Distribution.

Vietnam.

#### Sthulapada vietnamensis sp. n.

Taxon classificationAnimaliaHymenopteraChalcididae

http://zoobank.org/F6397F36-B1E4-46A9-842D-20B8232F4648

Figs 192 , 193–194

##### Type material.

Holotype, ♀ (RMNH), “**Vietnam**: Dak Lak, Chu Yang Sin N. P., Krong K’Mar, Mal. traps 12–13, 590 m, 22–26.x.2005, C. v. Achterberg & R. de Vries, RMNH’05”.

##### Diagnosis.

This new species differs from *Sthulapada padata* Narendran in having: 1) antenna mostly black with scape and pedicel pale brownish yellow and pale brown, respectively (in *Sthulapada padata* antenna is immaculate yellow); 2) legs mostly black (in *Sthulapada padata* legs yellow, but hind coxa slightly dark brown basally); 3) metasoma black with epipygium reddish brown (in *Sthulapada padata* metasoma pale brownish yellow basally and latero-ventrally); 4) apex of scutellum distinctly bi-lobed (in *Sthulapada padata* apex of scutellum not bi-lobed), and 5) propodeum without a median carina (in *Sthulapada padata* propodeum with a short median carina).

##### Description.

Holotype, ♀, length of body 6.6 mm.

*Colour*. Black except the following: scape pale brownish yellow; pedicel pale brown; apex of clava pale pink; eyes and ocelli pale reflecting yellow; tegula pale brownish yellow; apex of fore femur, fore tibia and tarsi pale brownish yellow; base and apex of mid femur pale brownish yellow; base and apex of hind tibia pale brown; hind tarsi pale brown; epipygium and caudal tooth reddish brown. Wings hyaline with veins dark brown with a patch of infuscation behind STV.

*Head*. Head closely pitted, interstices narrower than diameter of a pit; width of head in anterior view 1.1x its height; width in dorsal view 2.1 × its length; occiput concave; POL 5.2 × OOL; AOL 2.4 × OOL; distance between eyes in dorsal view 2.2 × POL; pre-orbital carina starting from malar ridge running upwards towards vertex and meeting each other behind anterior ocellus; geno-temporal furrow distinct but not deep; scrobe cross reticulate, not reaching anterior ocellus, margins ecarinate; height of malar space 0.5 × eye height in profile; eye length in profile 0.7 × eye height; eyes bare; antennae inserted well below level of ventral margin of eyes; scape not at all reaching anterior ocellus; relative L:W of antennal segments:scape = 25:4; pedicel = 5:4; F1–F7 = 5:6; clava = 10:6.

*Mesosoma*. Mesosoma closely pitted on pronotum, mesoscutum and scutellum; interstices narrower than half diameter of a pit, posterior margin concave medially; apex of scutellum bi-lobed; propodeum horizontal, with distinct submedian and sublateral carinae; area between plical carina and submedian carina coarsely sculptured with a median micro-reticulate part; area between plical carina and sublateral carina transversely carinate; lateral prespiracular part with a tooth on either side of propodeum; spiracle somewhat bean-shaped.

*Wings*. Fore wing 2. 7x as long as wide; relative length of CC = 53; SMV = 51; MV = 7; PMV = 3; STV = 4; MV separated from SMV by a distinct break; distal end of SMV not clavate as *Sthulapada padata*.

*Legs*. Hind coxa without a dorsal tooth; hind femur greatly swollen, 0.6 × as broad as its length, its ventral margin weakly bi-lobed with a row of teeth; inner basal part of hind femur with a tooth; hind tibia with an extra external carina; hind tarsal segments relatively larger, much wider than long; fifth tarsal segment of hind leg unusually larger than other tarsal segments; each claw unidentate.

*Metasoma*. Metasoma 1.4 × as long as mesosoma; T1 with 3 basal carinae; length of lateral carina 0.3 × length of T1 in dorsal view; length of median carina 0.2 × length of T1; length of T1 in dorsal view a little less than half length of metasoma; T1 smooth and shiny, posterior margin a little convex; T2 smooth and shiny with 2 rows of setigerous pits; T3 weakly shagreened with 1–2 transverse rows of minute setigerous pits; T3 to T5 more distinctly shagreened with 1–2 rows of setigerous pits; posterior margin of T2 to T5 slightly to distinctly concave progressively; T6 distinctly pitted and pubescent; epipygium carinate at middle, its median length subequal to median length of T6; epipygium with a distinct posteriorly directed ventral tooth just below ovipositor sheath and closely attached to it.

*Male*. Unknown.

##### Host.

Unknown.

##### Distribution.

Vietnam.

#### Tanycoryphus

Taxon classificationAnimaliaHymenopteraChalcididae

Cameron, 1905

**Figure 195.**
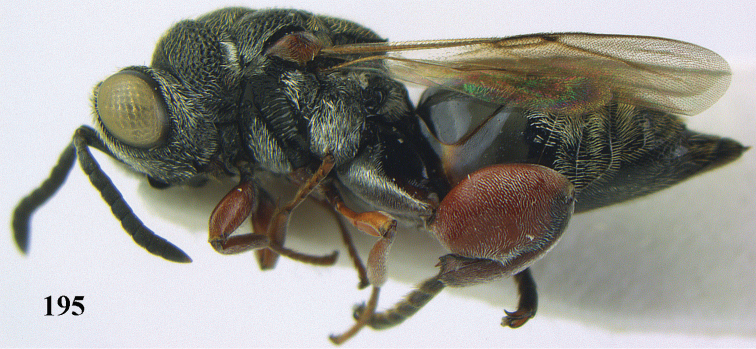
*Tanycoryphus masii* sp. n., ♀, holotype, habitus lateral.

**Figure 196.**
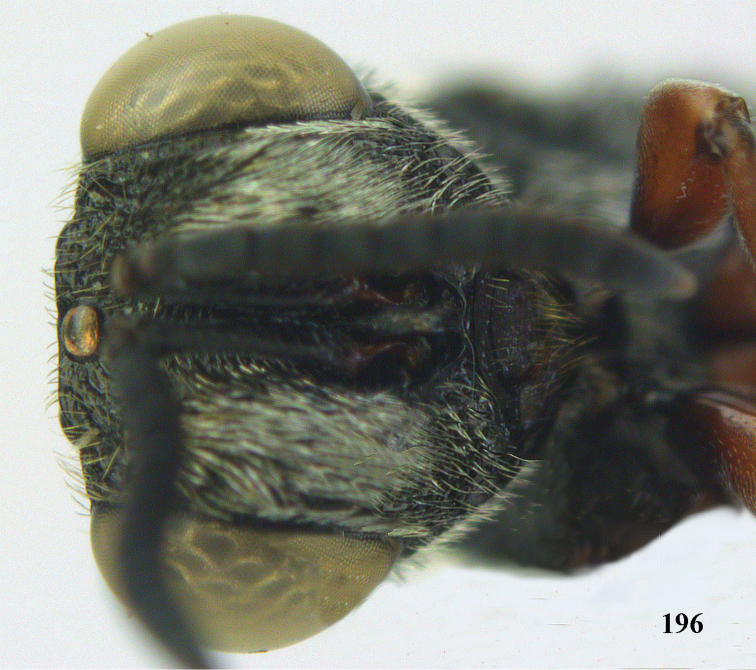
*Tanycoryphus masii* sp. n., ♀, holotype, head anterior.

**Figures 197–199.**
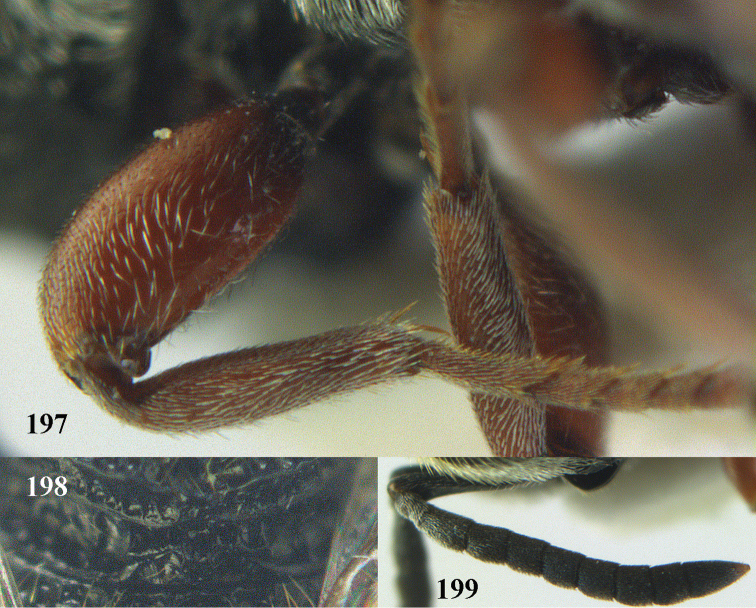
*Tanycoryphus masii* sp. n., ♀, holotype. **197** fore leg posterior **198** metanotum (postscutellum) dorsal **199** antenna lateral.

Figs 195 , 196 , 197–199

Tanycoryphus Cameron, 1905: 313. Type species: Tanycoryphus sulcifrons Cameron, by monotypy.
Sabatius Masi, 1929a: 163. Type species: Sabatius ater Masi, by monotypy. Synonymised with Tanycoryphus by Steffan (1957).
Malambrunia Masi, 1929a: 169. Type species: Malambrunia merisicornis Masi, by monotypy. Synonymised with Tanycoryphus by Steffan (1950).

##### Diagnosis.

The genus resembles the extralimital genera *Chirocera* Latreille, 1825, and *Tanyotorthus* Steffan, 1955, in general appearance, but differs from them in having PMV relatively shorter (in *Chirocera* and *Tanyotorthus* PMV 4–5 × longer) and in having pre- and post-orbital carinae present (pre- and post-orbital carinae indistinct in *Chirocera* and *Tanyotorthus*).

##### Description.

Clava narrowed towards apex; fore tibia swollen; tip of hind tibia with a characteristic outer spur and outer carina; postscutellum with characteristic rugae. MV at the wing margin; PMV and STV present.

##### Hosts.

Unknown.

##### Distribution.

Asia and Africa.

#### Tanycoryphus masii sp. n.

Taxon classificationAnimaliaHymenopteraChalcididae

http://zoobank.org/C1397CA3-B004-4688-B689-C989BC80127C

Figs 195 , 196 , 197–199

##### Type material.

Holotype, ♀ (RMNH), “**Vietnam**: Ninh Thuân, Núi Chúa N. P., Northeast part, Mal. traps, 90–150 m, 23–30.v.2007, C. v. Achterberg & R. de Vries, RMNH’07”.

##### Diagnosis.

In the key to species by Narendran (1989) this new species comes to *Tanycoryphus merisicornis* (Masi, 1929), but differs from it in having: 1) pronotal carina absent medially (in *Tanycoryphus merisicornis* pronotal carina prominent medially); 2) width of fore tibia half width of fore femur (in *Tanycoryphus merisicornis* width of fore tibia 0.7 × width of fore femur); 3) F1 longer than F2 (in *Tanycoryphus merisicornis* F1 not longer than F2), and 4) each lateral ocellus connected to eye margin by a cross carina (no such carina in *Tanycoryphus merisicornis*).

##### Description.

Holotype, ♀, length of body 8.5 mm.

*Colour*. Black but eyes pale yellow with reflecting yellow spots, ocelli pale reflecting yellow, tegula yellowish brown and legs reddish brown except black fore and hind coxae; wings hyaline with slight brownish tinge; pubescence on face and gena silvery; pubescence on vertex, pronotum and mesoscutum slightly yellowish except silvery bunch on posterior corners of pronotum and anterior outer corners of scapulae; pubescence on metasoma golden yellow.

*Head*. Width of head 1.2 × its height in anterior view, a little wider than mesosoma (24:21), 2.6 × its length in dorsal view; pre-orbital carina running upwards weakly joining each other behind anterior ocellus; post-orbital carina running upwards to vertex; MS represented as a malar ridge, height malar space 0.4 eye height in profile; eyes bare, eye length in profile 0.7 × eye height; geno-temporal furrow indistinct due to dense pilosity on gena; scrobe not quite reaching anterior ocellus, cross striate; POL 2.6 × OOL; AOL as long as OOL; LOL as long as AOL; minimum width between eyes in dorsal view 2.4 × POL; each lateral ocellus and vertex with deep, close setigerous pits; face and gena densely pubescent; antenna inserted close to clypeus; radicula 0.3 × length of scape, scape almost reaching anterior ocellus; antennal formula 11171; relative L:W of antennal segments:scape = 25:4; pedicel = 3:4; anellus = 2:4; F1 = 8:6; F2–F4 = 6:6; F5–F7 = 5:6; clava = 5:9.

*Mesosoma*. Pronotum with anterior carina obsolescent in median part (visible on sides only), with deep, close setigerous pits, interstices narrower than diameter of a pit, rugulose; mesoscutum with relatively smaller sized close pits on anterior part, pits becoming slightly larger and more widely spaced towards posterior part; notauli distinct and pitted; area surrounding pronotal spiracle with dense white pubescence; tegula pubescent; scutellum with close, deep, setigerous pits, interstices narrower than diameter of a pit; apex of scutellum widely emarginated with two teeth; axillula densely pubescent just behind tegula; propodeum with two submedian carinae enclosing areola and deep pits; other areas with areolae and pits; sublateral carinae distinct; lateral teeth absent.

*Wings*. Fore wing 1.7 × as long as wide; relative length of fore wing CC and veins: CC = 22; SMV = 19; MV = 5; PMV = 7; STV = 2; CC 11 × as long as wide.

*Legs*. Hind coxa without a dorso-basal tooth; hind femur without an inner basal tooth, with a row of teeth on ventral margin in two lobes, distal lobe larger than proximal lobe; hind tibia densely pubescent on distal half, with characteristic broad apex and spur, with outer carina resent.

*Metasoma*. Metasoma a little longer than mesosoma (40:36); T1 smooth and shiny, without basal carinae, posterior margin convex, a little shorter than half length of metasoma; T2 to T5 densely pubescent and micro-sculptured; T6 with distinct close setigerous pits; epipygium carinate at middle, a little shorter than T6 (9:10); ovipositor sheath 0.44 × length of epipygium.

*Male*. Unknown.

##### Host.

Unknown.

##### Etymology.

Named after Luigi Masi (1879–1961), for his great contributions to the taxonomy of Chalcidoidea.

#### Trigonura

Taxon classificationAnimaliaHymenopteraChalcididae

Sichel, 1865

Figs 200 , 201

Trigonura Sichel, 1865: 358, 376–377 (as subgenus of Phasganophora Sichel, 1865). Type species: Phasganophora crassicauda Sichel, by monotypy.
Trigonura ; Kirby 1883: 54, 59–60 (upgraded to genus level).
Bactrochalcis Kieffer, 1912: 463. Type species: Bactrochalcis reticulata Kieffer, by monotypy. Synonymised with Trigonura Sichel by Steffan (1951b).
Centrochalcis Cameron, 1913: 92. Type species: Centrochalcis ruficaudis Cameron, by monotypy. Synonymised with Trigonura Sichel by Waterston (1922).
Centrochalcidea Gahan & Fagan, 1923: 28. Replacement name for Centrochalcis Cameron, 1913, not 1905.
Chalcidellia Girault, 1924a: 1–3. Type species: Chalcis euthyrrhini Dodd, by original designation. Synonymised with Trigonura Sichel by Bouček (1988b).
Urochalcis Nikol’skaya, 1952: 91. Type species: Urochalcis ninae Nikol’skaya, by original designation. Synonymised with Trigonura Sichel by Nikol’skaya (1960).

##### Diagnosis.

This genus comes very close to *Trigonurella* Bouček, but *Trigonurella* differs from *Trigonura* in having: 1) lower face with conspicuous raised, X-shaped structure (fig. 251 of Narendran 1989); 2) face flat; 3) eye margin delimited by pre-orbital carina and hind femur with a large basal tooth. In *Trigonura* the lower face is regularly punctate; the face is convex without strong pre-orbital carina and hind femur without broad tooth.

##### Description.

Face convex, without strong pre-orbital carinae; malar space with coarse punctation but without malar sulcus or carina; antenna inserted about at level with lowest margin of eyes; interstices between pits on dorsum of mesosoma raised, rasp-like; T1 normally convex, dorsally shiny, with fine punctures, rarely dull with rugose substriate sculpture; T1 much larger than T2.

##### Variation.

Some characters show intrageneric variation. The typical distinct flat and punctured interantennal space varies to narrow upwardly produced (into scrobal space) interantennal space in some species groups. The apex of the metasoma of female varies in length. The apex of scutellum is mostly rounded but in some species medially truncate. In some species T1 has a dull rugose area (Bouček 1992, 1988b; Steffan 1951b; Burks 1959).

##### Hosts.

Parasitoids of coleopterous larvae (Buprestidae, Curculionidae, Scolytidae).

##### Distribution.

New World, Africa, Asia, Australia and New Guinea (Bouček 1988b).

**Figure 200.**
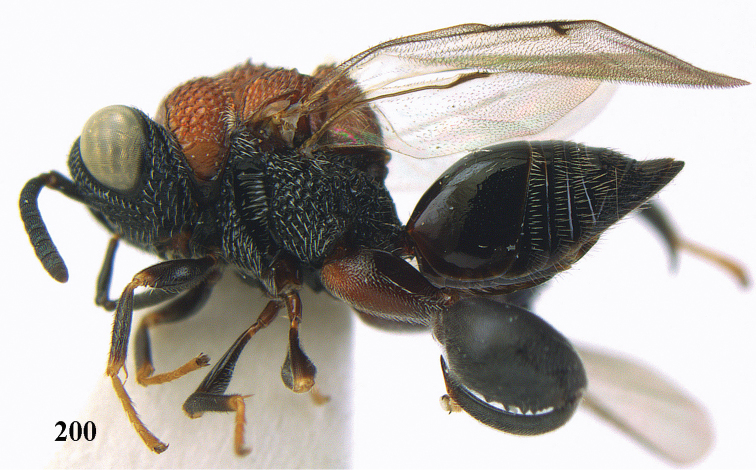
*Trigonura luzonensis* Narendran, ♀, Cát Tiên N. P., habitus lateral.

**Figure 201.**
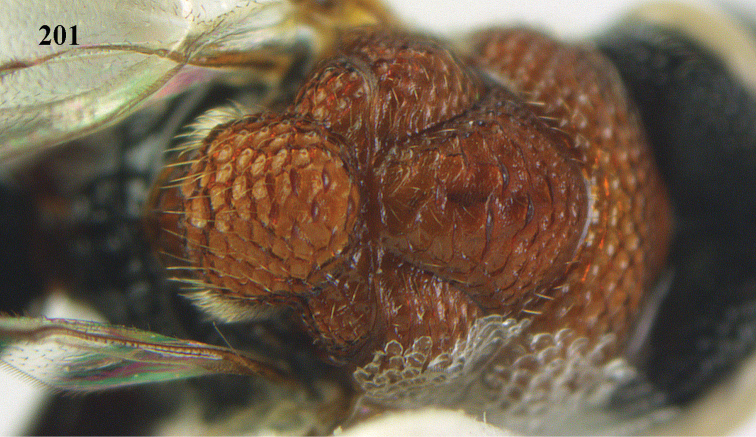
*Trigonura luzonensis* Narendran, ♀, Cát Tiên N. P., mesosoma dorsal.

#### Trigonura luzonensis

Taxon classificationAnimaliaHymenopteraChalcididae

Narendran, 1987

Figs 200 , 201

Trigonura luzonensis Narendran, 1987a: 288 (♀, Luzon, Philippines (USNM)), 1989: 216.

##### Material.

1 ♀ (RMNH), “S. **Vietnam**: Dóng Nai, Cát Tiên N. P., c. 100 m, 1–9.x.2005, *Ficus* trail, Malaise traps 1–8, C. v. Achterberg & R. de Vries, RMNH’05.

##### Diagnosis.

This species resembles *Trigonura indica* Narendran in having the metasoma acuminate and oval and scutellum highly vaulted. However, *Trigonura luzonensis* differs from *Trigonura indica* in many features. In *Trigonura luzonensis* the outer disc of hind femur is densely and minutely punctate and blackish, without any yellow spot, whereas in *Trigonura indica* the punctures are less close on the hind femur and it is yellowish-red with a distinct characteristic yellow spot at the apex. The hind tibia is reddish-brown with its base black in *Trigonura indica*, whereas in *Trigonura luzonensis* the hind tibia is completely black. In addition, the punctation on T6 differs in both species.

##### Description.

♀, length of body 6.2–7.0 mm.

*Colour*. Head black; eye and ocellus reflecting pale yellow; mandible rusty red except its black tips; mesosoma dorsally rusty-red and laterally and ventrally black; legs black with following parts rusty-red: tip of fore femur, base and tip of fore tibia, base and tip of mid tibia, hind coxa and all tarsi; fore wing slightly smoky.

*Head*. Head with large close, umbilicate pits, interstices shorter than half diameter of a pit, rugose, almost carinate in most areas; POL 2.5–3.1 × OOL; interantennal projection in Vietnamese specimen reach just below anterior ocellus as a continuation of a carina from distal part of inter-antennal projection.

*Mesosoma*. Pronotum with deep, close pits, interstices rugose-carinate; anterior margin of pronotum carinate laterally, posterior margin concave in middle; mesoscutum highly convex and vaulted with interstices between pits projecting as humps; scutellum highly convex and similarly sculptured.

*Wings*. Fore wing with PMV slightly longer than STV, 0.3 × as long as MV.

*Legs*. Hind coxa densely punctate and pubescent on ventral side; hind femur with close minute setigerous pits, outer ventral margin with a row of irregular teeth, basal tooth much larger than others.

*Metasoma*. Metasoma with a small T1 (shorter than its width); metasoma a little longer than mesosoma.

*Male*. Similar to ♀ except for stouter antenna and shorter metasoma.

##### Host.

Unknown.

##### Distribution.

Philippines, India, Vietnam (new record).

##### Variation.

The Vietnamese specimen has the dorsal part and distal ventral half of the hind coxa black and remaining baso-ventral part rusty red (Fig. 200).

## Supplementary Material

XML Treatment for Antrocephalus

XML Treatment for Antrocephalus decipiens

XML Treatment for Antrocephalus lugubris

XML Treatment for Antrocephalus maculipennis

XML Treatment for Antrocephalus nasutus

XML Treatment for Antrocephalus neogalleriae

XML Treatment for Antrocephalus sepyra

XML Treatment for Antrocephalus validicornis

XML Treatment for Brachymeria

XML Treatment for Brachymeria alternipes

XML Treatment for Brachymeria aurea

XML Treatment for Brachymeria bengalensis

XML Treatment for Brachymeria carinata

XML Treatment for Brachymeria coxodentata

XML Treatment for Brachymeria euploeae

XML Treatment for Brachymeria excarinata

XML Treatment for Brachymeria hime

XML Treatment for Brachymeria jambolana

XML Treatment for Brachymeria kamijoi

XML Treatment for Brachymeria lasus

XML Treatment for Brachymeria longiscaposa

XML Treatment for Brachymeria lugubris

XML Treatment for Brachymeria margaroniae

XML Treatment for Brachymeria marmonti

XML Treatment for Brachymeria megaspila

XML Treatment for Brachymeria minuta

XML Treatment for Brachymeria neowiebesina

XML Treatment for Brachymeria olethria

XML Treatment for Brachymeria podagrica

XML Treatment for Brachymeria scutellocarinata

XML Treatment for Brachymeria semirusula

XML Treatment for Brachymeria shansiensis

XML Treatment for Brachymeria taiwana

XML Treatment for Brachymeria wiebesina

XML Treatment for Dirhinus

XML Treatment for Dirhinus anthracia

XML Treatment for Dirhinus auratus

XML Treatment for Dirhinus claviger

XML Treatment for Dirhinus neoclaviger

XML Treatment for Dirhinus secundarius

XML Treatment for Epitranus

XML Treatment for Epitranus albipennis

XML Treatment for Epitranus ater

XML Treatment for Epitranus crassicornis

XML Treatment for Epitranus erythrogaster

XML Treatment for Epitranus gauldi

XML Treatment for Epitranus narendrani

XML Treatment for Epitranus neonigriceps

XML Treatment for Epitranus nigriceps

XML Treatment for Epitranus parvidens

XML Treatment for Epitranus oxytelus

XML Treatment for Epitranus ramnathi

XML Treatment for Haltichella

XML Treatment for Haltichella clavicornis

XML Treatment for Haltichella delhensis

XML Treatment for Haltichella nipponensis

XML Treatment for Heydoniella

XML Treatment for Heydoniella vietnamensis

XML Treatment for Hockeria

XML Treatment for Hockeria bangalorica

XML Treatment for Hockeria guptai

XML Treatment for Kriechbaumerella

XML Treatment for Kriechbaumerella ayyari

XML Treatment for Kriechbaumerella cordigaster

XML Treatment for Kriechbaumerella destructor

XML Treatment for Kriechbaumerella nepalensis

XML Treatment for Megachalcis

XML Treatment for Megachalcis carinata

XML Treatment for Megachalcis vietnamensis

XML Treatment for Notaspidium

XML Treatment for Notaspidium vietnamicum

XML Treatment for Oxycoryphe

XML Treatment for Oxycoryphe neotenax

XML Treatment for Oxycoryphe scutellatus

XML Treatment for Psilochalcis

XML Treatment for Psilochalcis carinigena

XML Treatment for Smicromorpha

XML Treatment for Smicromorpha masneri

XML Treatment for Sthulapada

XML Treatment for Sthulapada neopadata

XML Treatment for Sthulapada vietnamensis

XML Treatment for Tanycoryphus

XML Treatment for Tanycoryphus masii

XML Treatment for Trigonura

XML Treatment for Trigonura luzonensis
